# Synthesis and vectorial functionalisation of pyrazolo[3,4-*c*]pyridines†

**DOI:** 10.1039/d3ra07458g

**Published:** 2023-11-23

**Authors:** Elizabeth V. Bedwell, Flavio da Silva Emery, Giuliano C. Clososki, Patrick G. Steel

**Affiliations:** a Department of Chemistry, University of Durham South Road Durham DH1 3LE UK p.g.steel@durham.ac.uk; b Departamento de Ciências Farmacêuticas, Faculdade de Ciências Farmacêuticas de Ribeirão Preto, Universidade de São Paulo Ribeirão Preto SP Brazil; c Departamento de Ciências Biomoleculares, Faculdade de Ciências Farmacêuticas de Ribeirão Preto, Universidade de São Paulo Ribeirão Preto SP Brazil

## Abstract

Heterocycles are a cornerstone of fragment-based drug discovery (FBDD) due to their prevalence in biologically active compounds. However, novel heterocyclic fragments are only valuable if they can be suitably elaborated to compliment a chosen target protein. Here we describe the synthesis of 5-halo-1*H*-pyrazolo[3,4-*c*]pyridine scaffolds and demonstrate how these compounds can be selectively elaborated along multiple growth-vectors. Specifically, N-1 and N-2 are accessed through protection-group and *N*-alkylation reactions; C-3 through tandem borylation and Suzuki–Miyaura cross-coupling reactions; C-5 through Pd-catalysed Buchwald–Hartwig amination; and C-7 through selective metalation with TMPMgCl^.^LiCl followed by reaction with electrophiles or transmetalation to ZnCl_2_ and Negishi cross-coupling. Linking multiple functionalisation strategies emulates a hit-to-lead pathway and demonstrates the utility of pyrazolo[3,4-*c*]pyridines to FBDD.

## Abstract 2

Vectorial functionalisation of heterocyclic cores provides a simple route to diverse building blocks for fragment-based drug discovery application.

## Graphical abstract

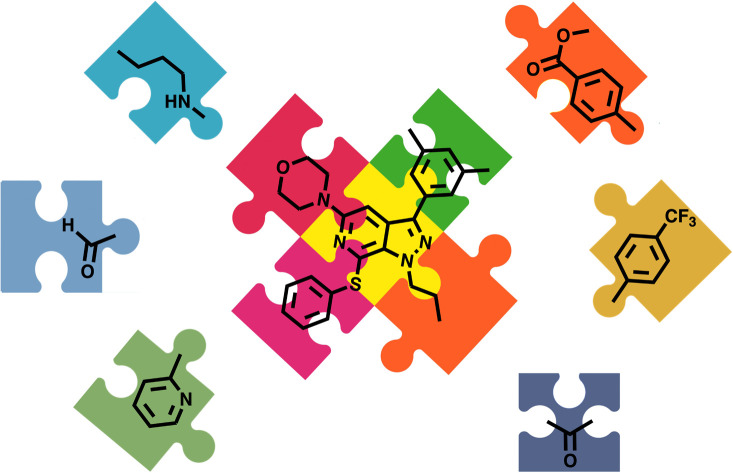

## Introduction

Through the identification of weakly binding small-molecules (fragments) and their subsequent elaboration into larger more potent lead compounds, fragment-based drug discovery (FBDD) has become a major strategy for the development of new drug leads.^1,2^ Heterocyclic compounds represent attractive starting points for FBDD due to an ability to engage with the target protein through a wide variety of intermolecular interactions, coupled with the potential to optimise drug-like properties (lipophilicity, hydrogen bonding capacity, and polarity) through modification of substitution patterns. Given the requirements to compliment a protein's 3D-structure, fragment elaboration requires multiple positions, also called growth-vectors, to be decorated with different motifs to introduce new, or improve existing, binding interactions with the target. It is therefore important to be able to functionalise a given fragment along the available growth-vectors in a chemically diverse yet directionally controlled manner.

Whilst FBDD has been successfully applied to several studies, this has largely been achieved using a relatively limited set of heteroaromatic scaffolds.^3^ A computational enumeration of the underutilized areas of chemical space was conducted to address this deficiency, introducing a set of novel heterocyclic motifs, the “heteroaromatic rings of the future”, as valuable inputs to inspire new drug candidates.^4^ This stimulated a number of reports describing initial access to these structures.^5^ However, further functionalisation or introduction of alternative substituent patterns often requires developing challenging *de novo* syntheses. The complexity of this synthesis limits the structural diversity of compounds that can be rapidly generated for testing and so creates a bottle-neck in FBDD programs.^3^ Consequently, promising fragment hits may be down-prioritised due to supposed synthetic intractability.^6^

**Fig. 1:**
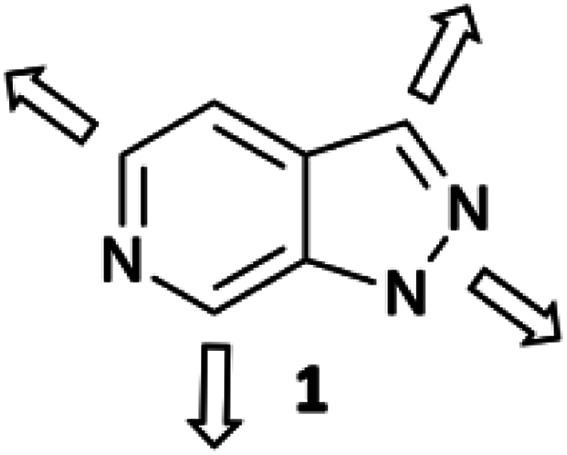
1*H*-Pyrazolo[3,4-*c*]pyridine 1; the heterocyclic scaffold chosen for elaboration by vectorial functionalisation along the 4 major growth vectors.

To address these issues and increase efficiency of hit-to-lead elaboration of heterocyclic fragments, we have initiated a programme to explore late-stage functionalisation of heterocyclic cores that can be applied in a sequential and vectorially diverse manner. In this report we describe the application of complementary C–H activation methods to the vectorial elaboration of a 1*H*-pyrazolo[3,4-*c*]pyridine 1 scaffold (Fig. 1) as a representative example of this fragment set.

## Results

**Scheme 1:**
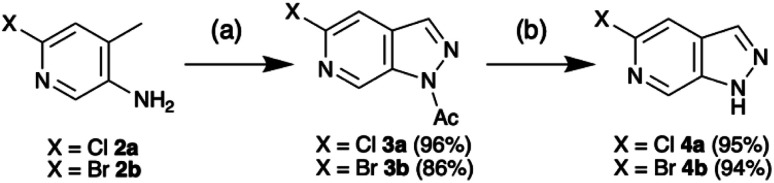
Synthesis of 5-chloro-1*H*-pyrazolo[3,4-*c*]pyridine 4a and 5-bromo-1*H*-pyrazolo[3,4-*c*]pyridine 4b. Conditions: (a) NaNO_2_, Ac_2_O, DCE, rt – 90 °C, 20 h, (b) MeOH, NaOMe, rt, 1 h.

Access to the 5-methoxy-pyrazolo[3,4-*c*]pyridine was recently reported by Silva Júnior *et al.* in an adaptation of the procedure first reported by Chapman and Hurst based on the classical Huisgen indazole synthesis.^7,8^ In further optimisation, introduction of dichloroethane (DCE) as a co-solvent enhanced scalability and enabled isolation of the 1-(5-halo-pyrazolo[3,4-*c*]pyridine-1-yl)ethan-1-ones 3 without need for purification (Scheme 1). Following simple deacetylation of 3 with NaOMe/MeOH, the desired 5-halo-1*H*-pyrazolo[3,4-*c*]pyridines 4 were isolated in excellent overall yield. With the scaffold in hand, the initial objective was to develop selective N-protection sequences. Using precedents based on standard indazole chemistry and tailoring reaction conditions enabled functionalisation of either N-1 or N-2.^9^

Mesylation (Ms) selectively afforded the N-1 protected product 5a in 92% yield (Table 1, row 1). However, this group proved less useful than hoped due to the tendency for migration to C-3 as exploited by Silva Júnior *et al.*^10–12^ Tetrahydropyran (THP) protection of the bromo-scaffold 4b was similarly straightforward as the N-1 isomer 6a and N-2 isomer 6b could be produced selectively based on reaction time (Table 1, rows 2–3) with longer reaction times favouring the thermodynamically more stable N-1 protected product.^9^ Surprisingly, the chloro-analogues 7 could not be produced selectively without significant impact on the yield. Fortunately, the N-1 and N-2 protected products, 7a and 7b, could be readily separated by chromatography (Table 1, row 4). Complete selectivity in the introduction of a trimethylsilylethoxymethyl (SEM) group was also challenging. However, by careful choice of base, either NaH or *N*,*N*-dicyclohexylmethylamine, predominant formation of either the N-1 or N-2 isomer could be achieved with the organic base favouring the formation of the N-2 products, 8b and 9b (Table 1, rows 5–8).

**Table 1:** Selective N-1 and N-2 functionalisation of 5-halopyrazolo[3,4-*c*]pyridines

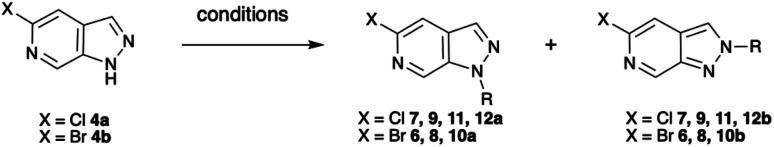					
Entry	X =	Reaction conditions	R =	PG_1_ (yield)	PG_2_ (yield)
1	Cl	MsCl, NaH, THF, 0 °C-rt, 2 h	-Ms	5a (92%)	5b (0%)
2	Br	DHP, *p*TsOH, DCM, rt, 2 h	-THP	6a (6%)	6b (75%)
3	Br	DHP, *p*TsOH, DCM, rt, 22 h	-THP	6a (82%)	6b (0%)
4	Cl	DHP, *p*TsOH, DCM, rt, 4 h	-THP	7a (14%)	7b (66%)
5	Br	SEMCl, Cy_2_MeN, THF, 0 °C-rt, 18 h	-SEM	8a (18%)	8b (32%)
6	Br	SEMCl, NaH, THF, 0 °C-rt, 6 h	-SEM	8a (47%)	8b (26%)
7	Cl	SEMCl, NaH, THF, 0 °C-rt, 6 h	-SEM	9a (45%)	9b (29%)
8	Cl	SEMCl, Cy_2_MeN, THF, 0 °C-rt, 18 h	-SEM	9a (21%)	9b (44%)
9	Br	MeI, NaH, 0 °C-rt, 1 h	-Me	10a (36%)	10b (50%)
10	Cl	MeI, NaH, 0 °C-rt, 1 h	-Me	11a (31%)	11b (42%)
11	Cl	PrI, NaH, 0 °C-rt, 24 h	-Pr	12a (27%)	12b (35%)

Finally, simple alkylation afforded the corresponding *N*-alkylated species as mixtures that could be separated by column chromatography (Table 1, rows 9–11).

**Scheme 2:**
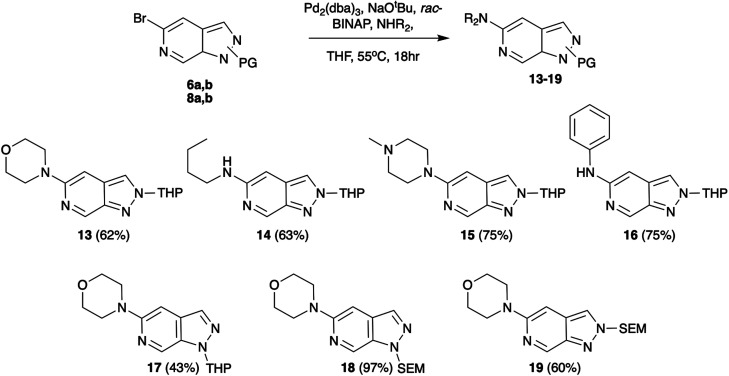
Pd-catalysed Buchwald–Hartwig amination examples.

As a first vector of functionalisation, we opted to explore Buchwald–Hartwig amination at C-5 (Scheme 2). A vast array of conditions exists in the literature varying base, ligand, solvent, and reaction time depending on the substrate.^13^ Following precedents established by Slade,^9^ the combination of Pd_2_(dba)_3_, *rac*-BINAP, and NaO^*t*^Bu in THF was successfully applied to reactions of 6b with primary, secondary, and aromatic amines generating products in yields of 62–75%. These conditions were also explored for various protection group strategies which revealed the reaction of 8a as the most successful affording 18 in 97% yield.

**Scheme 3:**
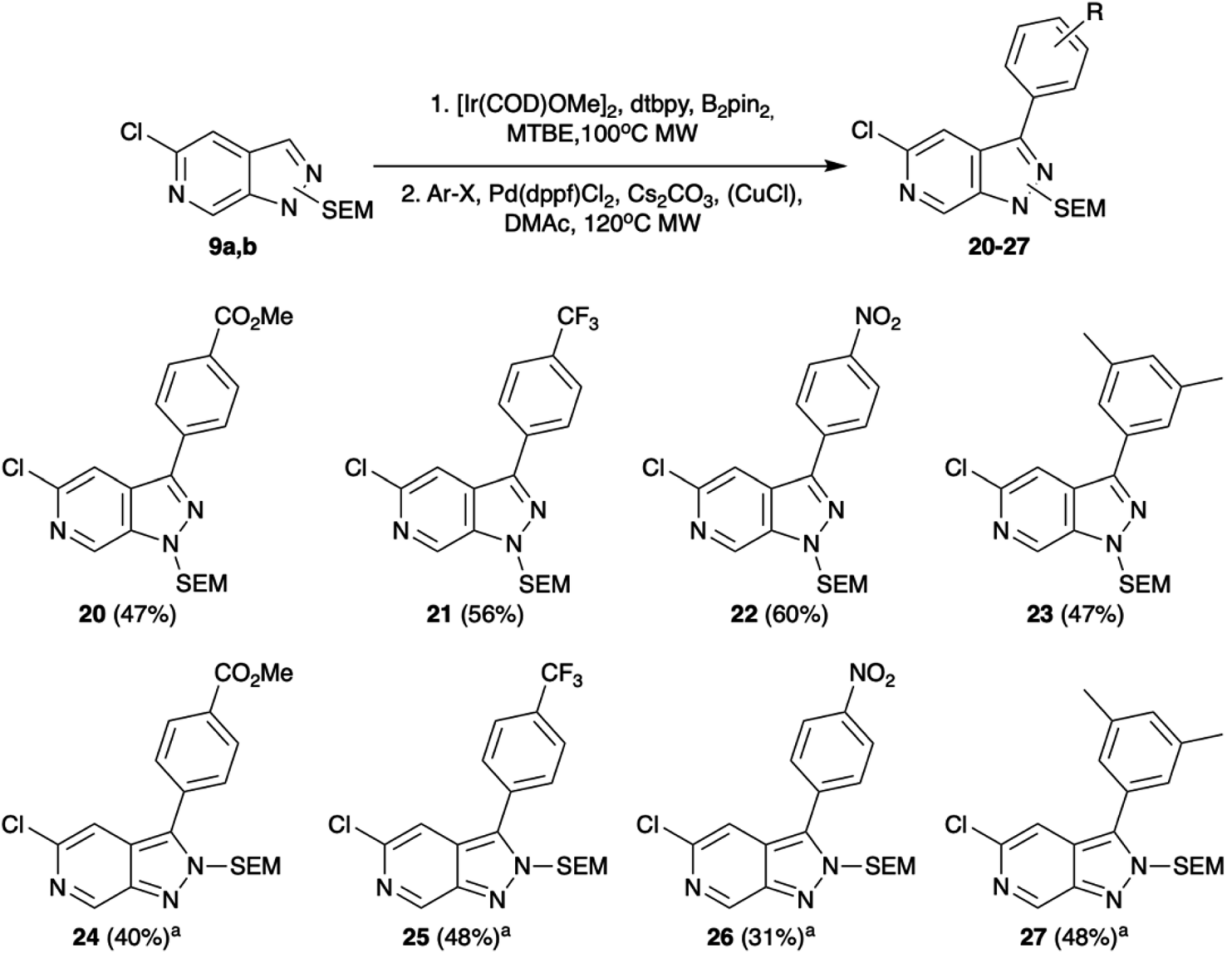
Tandem C–H borylation and Suzuki–Miyaura cross-coupling of 9. Conditions: (1) [Ir(COD)OMe]_2_, dtbpy, B_2_pin_2_, MTBE, 100 °C, MW, (2). Ar-X, Pd(dppf)Cl_2_, Cs_2_CO_3_, DMAc, 120 °C, MW. ^*a*^Additional CuCl.

Selecting C-3 as the next target vector, we again took precedent from indazole chemistry in which iridium catalysed C–H borylation has been shown to occur regioselectively in this position. Consistent with the literature reports, using conditions based on those optimised by Sadler *et al.*^14,15^ both 9a and 9b underwent efficient borylation at the C-3 position. The utility of these boronate esters was demonstrated through *in situ* Suzuki–Miyaura cross-coupling. Whilst Suzuki–Miyaura cross-coupling of the 9a substrate proceeded readily under standard conditions of Cs_2_CO_3_ and Pd(dppf)Cl_2_ in DMAc with yields of 47–60% (Scheme 3), rapid protodeborylation was a barrier to product generation for reaction of 9b. This was addressed by use of a CuCl additive to increase the rate of transmetalation *via* formation of an intermediary Cu species.^16^ Under these modified conditions, the desired C-3 functionalised products could be obtained in moderate to good overall yields (31–48%, Scheme 3).

Having established methodologies to functionalise N-1, C-3, and C-5, we then turned to the remaining vector of C-7. Initial attempts to exploit the azinyl nature of C-7 and couple the heterocycle with ^*n*^butyl-lithium led to nucleophilic addition at the C-7 position, albeit in very low yields. Consequently, we turned to more selective metalation chemistries with the mixed magnesium-lithium TMP (2,2,4,4-tetramethylpiperidine) bases.

**Scheme 4:**
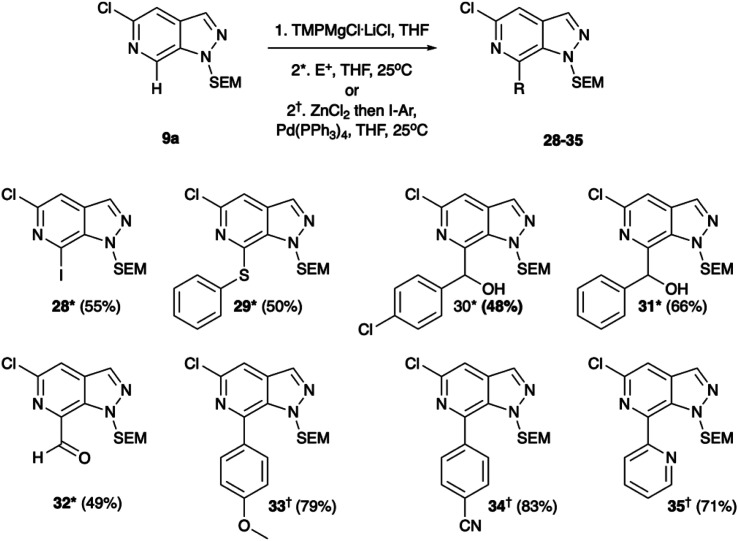
Metalation with TMPMgCl^.^LiCl reaction with electrophiles and transmetalation with ZnCl_2_ for Negishi cross-coupling. Conditions*: (1) TMPMgCl·LiCl, THF, −40 °C, (2) E^+^, THF, 25 °C or conditions^†^: (1). TMPMgCl^.^LiCl, THF, −40 °C, (2) ZnCl_2_ then I–Ar, Pd(PPh_3_)_4_, THF, 25 °C.

**Scheme 5:**
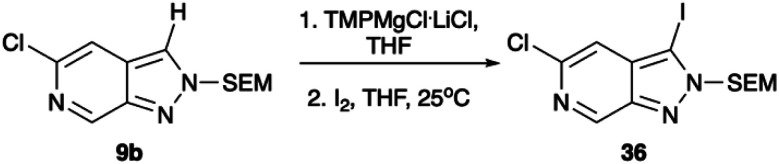
Metalation with TMPMgCl^.^LiCl reaction with iodine. Conditions: (1) TMPMgCl^.^LiCl, THF, −78 °C, 30 min, (2) I_2_, THF, 25 °C, 1 h.

Regioselective metalation by TMP-metal bases has been previously reported for a broad range of aromatic and heteroaromatic compounds.^17–19^ When applied to the N-1 SEM protected analogue 9a this afforded the desired metalation at C-7. The efficiency of the magnesiation step proved to be highly temperature dependent with optimal conversion, following trapping with iodine, being obtained at −40 °C (Table S1, ESI†). To demonstrate the broad scope of this chemistry, the intermediate organomagnesium species were treated with a variety of electrophiles including aldehydes, diphenyl disulfide, and DMF for yields of 48–66%. Alternatively, transmetalation with ZnCl_2_ afforded a range of arylated products *via* Negishi cross-coupling chemistry with yields of 71–83% (Scheme 4). Interestingly, TMPMgCl^.^LiCl treatment of the N-2 SEM-protected compound 13b led to metalation at C-3 instead of C-7 (Scheme 5). However, this reaction was inefficient and produced a complex mixture of products.

As the C-3 vector had already been thoroughly explored through Ir-catalysed borylation, this route was not pursued further.

While individual elaboration along each growth vector is a valuable tool to explore fragment space, it is likely that an FBDD optimisation may require multiple vectors of growth. As a final aspect we, therefore, sought to demonstrate how these functionalisation strategies could be combined to emulate a hit-to-lead pathway common to medicinal chemistry development.

**Scheme 6:**
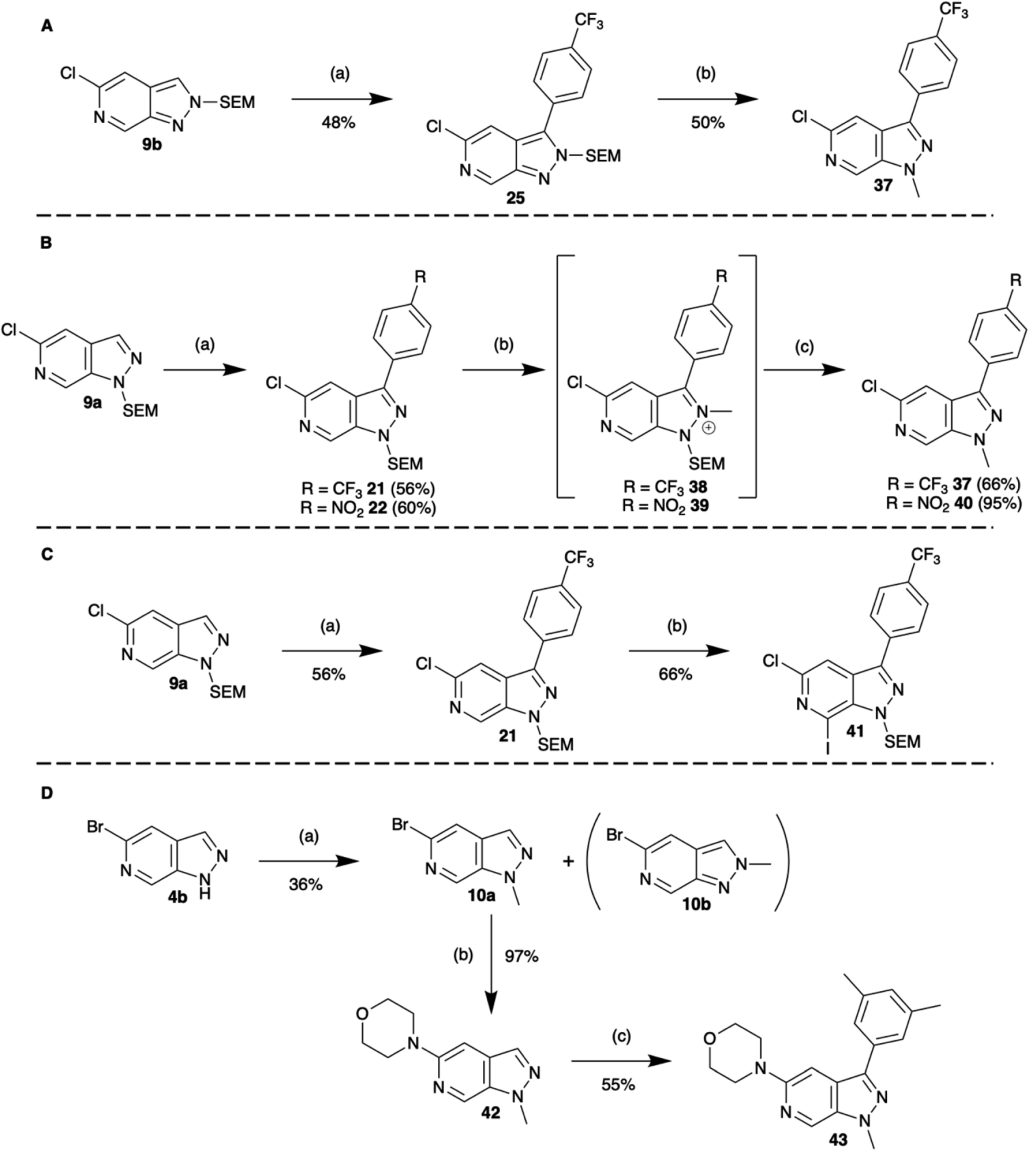
Multiple-vector elaboration sequences. (A) C-3 borylation, Suzuki–Miyaura cross-coupling with a tandem *N*-methylation/SEM-deprotection, conditions: (a) (1) [Ir(COD)OMe]_2_, dtbpy, B_2_pin_2,_ MTBE, 100 °C MW, 2. C_7_H_4_F_3_Br, Pd(dppf)Cl_2_, Cs_2_CO_3_, CuCl, DMAc, 120 °C MW, (b) [Me_3_O][BF_4_], EtOAc, rt, 18 h. (B) C-3 borylation, Suzuki–Miyaura cross-coupling with a tandem *N*-methylation/SEM-deprotection, conditions: (a) (1) [Ir(COD)OMe]_2_, dtbpy, B_2_pin_2,_ MTBE, 100 °C MW, 2. ArX, Pd(dppf)Cl_2_, Cs_2_CO_3_, DMAc, 120 °C MW, (b) [Me_3_O][BF_4_], EtOAc, rt, 6 h, (c) TFA, DCM, rt, 20 h. (C) C-3 borylation, Suzuki–Miyaura cross-coupling with C-7 TMPMgCl^.^LiCl metalation and electrophilic trapping with iodine, conditions: (a) (1) [Ir(COD)OMe]_2_, dtbpy, B_2_pin_2,_ MTBE, 100 °C MW, (2) C_7_H_4_F_3_Br, Pd(dppf)Cl_2_, Cs_2_CO_3_, DMAc, 120 °C MW, (b) (1) TMPMgCl^.^LiCl, THF, −40 °C, 30 min (2) I_2_, THF, rt, 1 h. (D) *N*-Methylation, Buchwald–Hartwig amination, and C-3 borylation, Suzuki–Miyaura cross-coupling, conditions: (a) MeI, NaH, THF, rt, 45 min, (b) Pd_2_(dba)_3_, NHR_2_, NaO^*t*^Bu, *rac*-BINAP, THF, 55 °C, 18 h, (c) (1) [Ir(COD)OMe]_2_, dtbpy, B_2_pin_2,_ MTBE, 100 °C MW 30 min (2) C_8_H_9_I, Pd(dppf)Cl_2_, Cs_2_CO_3_, DMAc, 120 °C MW 2 h.

In the first sequence, a combination of C-3 borylation, Suzuki–Miyaura cross-coupling and a tandem *N*-methylation/SEM-deprotection was explored (Scheme 6A). In the case of N-2 SEM-protected compound 25, reaction with [Me_3_O][BF_4_] (trimethyloxonium tetrafluoroborate) saw efficient formation of the N1-methylated product 37. In the case of the N-1 SEM-protected isomers, methylation of N-2 occurred readily with [Me_3_O][BF_4_] to afford the methyl salts 38 and 39.

However, surprisingly, acid catalysed SEM-deprotection with TFA did not give the expected conversion to N2-methylated compounds, instead, ipso methylation at N-1 was observed and 37 and 40 were isolated (Scheme 6B). It is likely that these observations reflect the greater thermodynamic stability of the 1*H*-pyrazolo[3,4-*c*]pyridine core.

A second two stage sequence paired C-3 borylation Suzuki–Miyaura cross-coupling functionalisation with C-7 TMPMgCl^.^LiCl metalation and electrophilic trapping with iodine to give 41 (Scheme 6C).

Finally, extensions to more elaborate three-vector functionalisation sequences are also possible. Exemplified in Scheme 6D showing a three-step sequence from fragment 4b to compound 43. Importantly, despite the increasing complexity, in each of these processes the yield of each step was comparable to that obtained earlier. Other sequences of elaboration can be endlessly imagined combining the chemistry explored herein to realise an expansive library of heterocyclic compounds.

## Conclusion

In summary, we have developed efficient synthetic routes to 5-halo-1*H*-pyrazolo[3,4-*c*]pyridine scaffolds and demonstrated how these compounds can be selectively elaborated along multiple reaction vectors. The pyrazolo[3,4-*c*]pyridine core has begun to attract attention as a therapeutic agent due to its structural similarity to purine.^20–23^ Purine derivatives have a broad range of applications, as anti-inflammatory, anti-viral, and anti-cancer agents, arising from the prevalence of purine-based biological compounds and, subsequently, the variety of cellular proteins that contain purine-binding pockets.^20^ Whilst routes have been reported for related products with different substituent patterns around the pyrazolo[3,4-*c*]pyridine core, most of these require discrete synthetic endeavour.^23–25^ The chemistry described here allows introduction of functionality at C-3, C-5 and C-7 and as such is complimentary to the recent related report by Silva Júnior *et al.* which describes alternative C-4 functionalisation of a 5-methoxy analogue.^10^

Importantly, through careful control of reaction sequence it is possible to selectively explore growth in a combination of vectors with no loss in synthetic efficiency.

Overall, the late-stage functionalisation sequences described here provide practical advantages, particularly for the introduction of structural diversity required for fragment-elaboration in an FBDD hit-to-lead evolution pipeline. Extensions of these ideas to other heterocyclic cores are in progress and the results of this will be reported in due course.

## Experimental section

All solvents and reagents were purchased from commercial suppliers and used without further purification unless otherwise stated below. Final compound purification by flash column chromatography was performed on a CombiFlash® System from Teledyne Isco equipped with an UV-light detector using prepacked silica RediSep Rf cartridges with the stated solvent gradient. Crude mixtures to be purified were dry loaded onto silica (normal phase) or Celite® 545 (reverse phase) prior to running the column. NMR spectra were recorded on the following instruments: Bruker Neo 700 MHz spectrometer with operating frequencies of 700 MHz for ^1^H and 175 MHz for ^13^C; Varian VNMRS-600 with operating frequencies of 600 MHz for ^1^H and 150 MHz for ^13^C NMR; and Varian VNMRS-400 with operating frequencies of 400 MHz for ^1^H and 376 MHz for ^19^F NMR. Spectra were referenced relative to CDCl_3_ (*δ*_H_ 7.26 ppm, *δ*_C_ 77.16 ppm), CD_3_OD (*δ*_H_ 4.87 ppm, *δ*_C_ 49.00 ppm), or DMSO (*δ*_H_ 2.50 ppm, *δ*_C_ 39.52 ppm). Chemical shifts are reported in parts per million (ppm), coupling constants (*J*) in hertz (Hz) and multiplicity as singlet (s), doublet (d), triplet (t), quartet (q), pentet (p), sextet (s), multiplet (m) or a combination thereof. All *J* values are *J*_H–H_ unless otherwise stated. All ^1^H NMR and ^13^C NMR spectral assignments were made with the aid of ^1^H^1^H COSY, ^1^H^1^H NOESY, ^1^H^13^C HSQC and ^1^H^13^C HMBC NMR experiments. Infra-red spectra were recorded on a PerkinElmer Paragon 1000 FT-IR spectrometer or a PerkinElmer RX FT-IR spectrometer with Golden Gate Diamond ATR apparatus. IR assignments are reported in wavenumbers (cm^−1^).

Melting points were recorded on Thermo Scientific Electrothermal IA9100 Digital Melting Point apparatus. Thin layer chromatography was performed using Merck F254 silica gel 60 aluminium sheets pre-coated with silica gel. High resolution mass spectrometry (HRMS) and liquid chromatography mass spectrometry (LC-MS) were recorded on a Waters TQD mass spectrometer ESI-LC water (0.1% formic acid): MeCN/MeOH, flow rate 0.6 mL min^−1^ with a UPLC BEH C18 1.7 μm (2.1 mm × 50 mm) column. Gas chromatography mass spectrometry (GCMS) was carried out on a Shimadzu QP2010-Ultra with a temperature gradient 50–300 °C and a hold time of 5 min, using a Rxi-17Sil MS (0.15 μm × 10 m × 0.15 mm) column.

### Substrate synthesis

#### 1′-{5-Chloro-1*H*-pyrazolo[3,4-*c*]pyridin-1-yl}ethan-1′-one 3a

Ac_2_O (33 mL, 0.35 mol, 10.0 eq.) was added to a solution of 6-chloro-4-methylpyridin-3-amine 2a (5.00 g, 35 mmol, 1.00 eq.) in DCE (140 mL) and stirred at room temperature for 90 minutes under nitrogen. NaNO_2_ (9.68 g, 0.14 mol, 4.00 eq.) was added and the reaction mixture stirred at room temperature for 3 hours, then heated overnight at 90 °C. The reaction mixture was concentrated under reduced pressure then diluted with NaHCO_3_ (150 mL). The product was extracted into EtOAc (5 × 100 mL) then washed with H_2_O (4 × 100 mL) and brine (2 × 100 mL), dried over MgSO_4_, filtered, and concentrated under reduced pressure to give 1′-{5-chloro-1*H*-pyrazolo[3,4-*c*]pyridin-1-yl}ethan-1′-one 3a as a white solid (6.57 g, 34 mmol, 96%) with mp 138–139 °C. *δ*_H_ (400 MHz, chloroform-*d*) 9.56 (1*H*, s, 7-*H*), 8.15 (1*H*, s, 3-*H*), 7.69 (1*H*, s, 4-*H*), 2.81 (3H, s, C(O)C*H*_3_); *δ*_C_ (101 MHz, chloroform-*d*) 170.3 (*C*=O), 144.7 (*C*-5), 138.0 (*C*-7), 137.8 (*C*-3), 134.7 (*C*-7a), 133.8 (*C*-3a), 114.8 (*C*-4), 22.6 (C(O)*C*H_3_); *V*_max_ (ATR) 1729 (C=O), 1390, 1353, 634 cm^−1^; LC-MS (ES+) [M (^35^Cl) + H] 196.069, [M (^37^Cl) + H] 198.007, [M (^35^Cl) + H − COCH_3_] 154.035, [M (^37^Cl) + H − COCH_3_] 156.011; HRMS (ES+) found [M + H]^+^ 196.0290, C_8_H_7_N_3_O^35^Cl requires *M* 196.0278. The analytical data were consistent with the literature.^7^

#### 5-Chloro-1*H*-pyrazolo[3,4-*c*]pyridine 4a

NaOMe (0.150 g, 2.8 mmol, 0.25 eq.) was added to a solution of 1′-{5-chloro-1*H*-pyrazolo[3,4-*c*]pyridin-1-yl}ethan-1′-one 3a (2.0 g, 10 mmol, 1.00 eq.) in anhydrous MeOH (50 mL) and stirred at room temperature for 15 minutes. The reaction was quenched by addition of HCl : MeOH 1 : 100 (8 mL) until acidic pH and concentrated under reduced pressure. The crude product was taken up in H_2_O (75 mL) then adjusted to pH 10 by addition of aqueous NaOH then extracted with EtOAc (3 × 100 mL). The combined organic layers were washed with brine (75 mL), dried over MgSO_4_, filtered and concentrated to afford 5-chloro-1*H*-pyrazolo[3,4-*c*]pyridine 4a as a white solid (1.5 g, 10 mmol, 95%) with mp 225–226 °C. *δ*_H_ (400 MHz, methanol-*d*_4_) 8.80 (1H, s, 7-H), 8.15 (1H, d, *J* = 1.2 Hz, 3-H), 7.82 (1H, d, *J* = 1.2 Hz, 4-H); *δ*_C_ (101 MHz, methanol-*d*_4_) 141.0 (*C*-5), 137.6 (*C*-7a), 135.1 (*C*-7), 134.2 (*C*-3), 131.2 (*C*-3a), 115.6 (*C*-4); *V*_max_ (ATR) 909, 736, 652 cm^−1^; LC-MS (ES+) [M (^35^Cl) + H] 154.113, [M (^37^Cl) + H] 156.128; HRMS (ES+) found [M + H]^+^ 154.0167, C_6_H_5_N_3_^35^Cl requires *M* 154.0172.

#### General procedure A – for Buchwald–Hartwig amination

Pd_2_dba_3_ (0.05 eq.), *rac*-BINAP (0.12 eq.), NaO^*t*^Bu (3.00 eq.), and the stated protected pyrazolo[3,4-*c*]pyridine (1.00 eq.) were sealed under a nitrogen atmosphere. The stated amine (1.10 eq.) was added under nitrogen, followed by dry THF (0.1 M). The deep red solution was stirred overnight at 55 °C until LCMS analysis confirmed complete conversion of the starting substrate. The reaction was cooled to room temperature, diluted with EtOAc and filtered through Celite, washing the cake with additional EtOAc. This solution was concentrated under reduced pressure. The product was purified by silica gel flash column chromatography using the stated solvent system.

#### 4-[2′-(Oxan-2′′-yl)-2*H*-pyrazolo[3,4-*c*]pyridin-5′-yl]morpholine 13

General procedure A was applied to 5-bromo-2-(oxan-2′-yl)-2*H*-pyrazolo[3,4-*c*]pyridine 6b (0.080 g, 0.28 mmol, 1.00 eq.) with morpholine (0.03 mL, 0.31 mmol, 1.10 eq.). After purification by silica gel flash column chromatography (EtOAc:Pet ether 40–60 0–100%), 4-[2′-(oxan-2′′-yl)-2*H*-pyrazolo[3,4-*c*]pyridin-5′-yl]morpholine 13 was isolated as a dark green oil (0.051 g, 0.18 mmol, 62%). *δ*_H_ (600 MHz, chloroform-*d*) 9.05 (1H, t, *J* = 1.2 Hz, 7′-*H*), 7.99 (1H, d, *J* = 1.2 Hz, 3′-*H*), 6.58 (1H, d, *J* = 1.2 Hz, 4′-*H*), 5.66 (1H, dd, *J* = 8.2, 4.1 Hz, 2′′-*H*), 4.13–4.07 (1H, m, 6′′-*H*), 3.91–3.87 (4H, m, 2,6-*H*), 3.77 (1H, ddd, *J* = 11.7, 10.4, 3.0 Hz, 6′′-*H*), 3.40–3.35 (4H, m, 3,5-H), 2.20 (2H, ddd, *J* = 10.0, 8.2, 4.1 Hz, 3′′-H), 2.07–2.01 (1H, m, 4′′-*H*), 1.79–1.63 (1H, m, 4′′-*H*), 1.79–1.63 (2H, m, 5′′-*H*); *δ*_C_ (151 MHz, chloroform-*d*) 154.1 (*C*-5′), 143.3 (*C*-7′), 142.7 (*C*-7′a), 126.1 (*C*-3′a), 119.3 (*C*-3′), 91.8 (*C*-4), 89.2 (*C*-2′′), 67.8 (*C*-6′′), 66.9 (*C*-2,6), 48.1 (*C*-3,5), 31.2 (*C*-3′′), 24.9 (*C*-5′′), 21.9 (*C*-4′′); *V*_max_ (ATR) 1495, 1200, 0998, 729 cm^−1^; LC-MS (ES+) [M + H] 289.294; HRMS (ES+) found [M + H]^+^ 289.1670, C_15_H_21_N_4_O_2_ requires M 298.1665.

### General procedure B – for tandem borylation and Suzuki–Miyaura cross-coupling

[Ir(COD)OMe]_2_ (0.025 eq.), B_2_pin_2_ (1.10 eq.), and dtbpy (0.05 eq.) were sealed in an oven-dried microwave reaction vial and degassed with N_2_/vacuum cycling. A solution of the SEM-protected pyrazolo[3,4-*c*]pyridine in anhydrous MTBE (0.4 M) was added under nitrogen. The reaction mixture was heated in a microwave reactor at 100 °C until GCMS analysis showed complete borylation had occurred, then concentrated under reduced pressure to afford the crude boronate ester. To the crude boronate ester was added Cs_2_CO_3_(2.00 eq.), Pd(dppf)Cl_2_(0.025 eq.), the aryl halide (1.10 eq.) and anhydrous DMAc (1 M) under nitrogen. The reaction mixture was heated in a microwave reactor at 120 °C until GCMS analysis showed no boronate ester remained. The reaction mixture was filtered through Celite® and the residue washed with EtOAc. The combined filtrates were concentrated under reduced pressure, and the residue was dissolved in H_2_O then extracted with EtOAc. The combined organic layers were washed with brine, dried over MgSO_4_, filtered, and concentrated. The product was purified by silica gel flash column chromatography using the stated solvent system.

#### 5-Chloro-3-(4′-nitrophenyl)-1-{[2′′-(trimethylsilyl)ethoxy] methyl}-1*H*-pyrazolo[3,4-*c*]pyridine 22

General procedure B was applied to 5-chloro-1-{[2′-(trimethylsilyl)ethoxy]methyl}-1*H*-pyrazolo[3,4-*c*]pyridine 9a (0.150 g, 0.53 mmol, 1.00 eq.) with 1-iodo-4-nitrobenzene (0.145 g, 0.58 mmol, 1.10 eq.). After purification by silica gel flash column chromatography (EtOAc:hexanes 0–20%), 5-chloro-3-(4′-nitrophenyl)-1-{[2′′-(trimethylsilyl)ethoxy]methyl}-1*H*-pyrazolo[3,4-*c*]pyridine 22 was isolated as a white solid (0.129 g, 0.32 mmol, 60%) with mp 111–113 °C. *δ*_H_ (600 MHz, chloroform-*d*) 8.96 (1H, d, J = 1.2 Hz, 7-*H*), 8.41–8.35 (2H, m, 3′,5′-*H*), 8.15–8.09 (2H, m, 2′,6′-*H*), 7.94 (1H, d, *J* = 1.2 Hz, 4-*H*), 5.86 (2H, s, NC*H*_2_O), 3.64–3.59 (2H, m, OC*H*_2_CH_2_), 0.95–0.88 (2H, m, CH_2_C*H*_2_SiMe_3_), −0.06 (9H, s, SiC*H*_3_); *δ*_C_ (151 MHz, Chloroform-*d*) 147.7 (*C*-4′), 143.0 (*C*-5), 141.4 (*C*-3), 138.1 (*C*-1′), 137.1 (*C*-7a), 134.4 (*C*-7), 129.2 (*C*-3a), 127.6 (*C*-3′, 5′), 124.4 (*C*-2′, 6′), 114.3 (*C*-4), 79.1 (N*C*H_2_O), 67.4 (O*C*H_2_CH_2_), 17.7 (CH_2_*C*H_2_Si), −1.5 (Si(*C*H_3_)_3_); *V*_max_ (ATR) 1516 (N=O asymmetric), 1349 (N=O symmetric), 1074, 857, 834, 821 cm^−1^; LC-MS (ES+) [M (^35^Cl) + H] 405.282 [M (^37^Cl) + H] 407.297; HRMS (ES+) found [M + H]^+^ 405.1153, C_18_H_22_N_4_O_3_Si^35^Cl requires *M* 405.1150.

### General procedure C – for tandem borylation and Suzuki–Miyaura cross-coupling with CuCl

[Ir(COD)OMe]_2_ (0.025 eq.), B_2_pin_2_ (1.10 eq.), and dtbpy (0.05 eq.) were sealed in an oven-dried microwave reaction vial and degassed with N_2_/vacuum cycling. A solution of the SEM-protected pyrazolo[3,4-*c*]pyridine in anhydrous MTBE (0.4 M) was added under nitrogen. The reaction mixture was heated in a microwave reactor at 100 °C until GCMS analysis showed complete borylation had occurred, then concentrated under reduced pressure to afford the crude boronate ester. To the crude boronate ester was added Cs_2_CO_3_(1.00 eq.), Pd(OAc)_2_(0.025 eq.), 1,1′-bis(diphenylphosphino)ferrocene (dppf) (0.050 eq.), CuCl (1.00 eq.), the aryl halide (1.10 eq.) and anhydrous DMAc (1 M) under nitrogen. The reaction mixture was heated in a microwave reactor at 120 °C until GCMS analysis showed no boronate ester remained. The reaction mixture was filtered through Celite® and the residue washed with EtOAc. The combined filtrates were concentrated under reduced pressure, and the residue was dissolved in H_2_O then extracted with EtOAc. The combined organic layers were washed with brine, dried over MgSO_4_, filtered, and concentrated. The product was purified by silica gel flash column chromatography using the stated solvent system.

#### 5-Chloro-3-(3′,5′-dimethylphenyl)-2-{[2′′-(trimethylsilyl)ethoxy] methyl}-2*H*-pyrazolo[3,4-*c*]pyridine 27

General procedure C was applied to 5-chloro-2-{[2′-(trimethylsilyl)ethoxy]methyl}-2*H*-pyrazolo[3,4-*c*]pyridine 9b (0.100 g, 0.35 mmol, 1.00 eq.) with 1-iodo-3,5-dimethylbenzene (0.06 mL, 0.39 mmol, 1.10 eq.). Purification by silica gel flash column chromatography (EtOAc:Pet ether 40–60 0–20%) afforded 5-chloro-3-(3′,5′-dimethylphenyl)-2-{[2′′-(trimethylsilyl)ethoxy]methyl}-2*H*-pyrazolo[3,4-*c*]pyridine 27 as a yellow oil (0.065 g, 0.17 mmol, 48%). *δ*_H_ (600 MHz, chloroform-*d*) 9.11 (1H, d, *J* = 1.2 Hz, 7-*H*), 7.57 (1H, d, *J* = 1.2 Hz, 4-*H*), 7.30–7.27 (2H, m, 2′,6′-*H*), 7.17–7.14 (1H, m, 4′-*H*), 5.72 (2H, s, NC*H*_2_O), 3.86–3.80 (2H, m, OC*H*_2_CH_2_), 2.43 (6H, q, *J* = 0.7 Hz, Ar-C*H*_3_), 0.98–0.93 (2H, m, CH_2_C*H*_2_Si), −0.01 (9H, s, Si(C*H*_3_)_3_); *δ*_C_ (151 MHz, chloroform-*d*) 144.6 (*C*-7), 143.9 (*C*-5), 140.6 (*C*-7a), 139.0 (*C*-3′,5′), 137.2 (*C*-3), 131.3 (*C*-4′), 127.8 (*C*-1′), 127.3 (*C*-2′,6′), 125.4 (*C*-3a), 113.5 (*C*-4), 79.8 (N*C*H2O), 68.1 (O*C*H_2_CH_2_), 21.4 (Ar-*C*H_3_), 17.9 (CH_2_*C*H_2_Si), −1.4 (Si(*C*H_3_)_3_); *V*_max_ (ATR) 1465, 1106, 1085, 1061, 858, 838 cm^−1^; LC-MS (ES+) [M (^35^Cl) + H] 388.333 [M (^37^Cl) + H] 390.347; HRMS (ES+) found [M + H]^+^ 388.1598, C_20_H_27_^35^ClN_3_OSi requires *M* 388.1612.

### General procedure D – for deproto-metalation by TMPMgCl^.^LiCl and trapping with an electrophile

An oven dried RBF was charged with a solution of the stated substrate (1.00 eq.) in dry THF (0.5 M) and cooled to −40 °C under a nitrogen atmosphere. TMPMgCl^.^LiCl in THF (2.00 eq.) was added dropwise and the reaction was stirred for 30 min at −40 °C. The corresponding electrophile was added at −40 °C, then the reaction was stirred at room temperature for the time stated. The reaction was quenched with NaHSO_3_ sat. solution and the crude product extracted with EtOAc, then the combined organic layers were washed with brine, dried over MgSO_4_, filtered, and concentrated under reduced pressure. The product was purified by silica gel flash column chromatography using the stated solvent system.

#### 5-Chloro-7-(phenylsulfanyl)-1-{[2′-(trimethylsilyl)ethoxy] methyl}-1*H*-pyrazolo[3,4-*c*]pyridine 29

General procedure D was applied to 5-chloro-1-{[2′-(trimethylsilyl)ethoxy]methyl}-1*H*-pyrazolo[3,4-*c*]pyridine 9a (0.150 g, 0.53 mmol, 1.00 eq.) with electrophile S_2_Ph_2_ (0.173 g, 0.79 mmol, 1.50 eq.) for 18 hours. Purification by reverse phase column chromatography (MeCN:H_2_O 0–100%) afforded 5-chloro-7-(phenylsulfanyl)-1-{[2′-(trimethylsilyl)ethoxy] methyl}-1*H*-pyrazolo[3,4-*c*]pyridine 29 as a yellow oil (0.104 g, 0.27 mmol, 50%). *δ*_H_ (700 MHz, chloroform-*d*) 7.98 (1H, s, 3-*H*), 7.58 (2H, dd, *J* = 7.5, 2.1 Hz, 2′′,6′′-*H*), 7.43 – 7.40 (2H, m, 3′′,5′′-*H*), 7.43–7.40 (1H, m, 4′′-*H*), 7.39 (1H, s, 4-*H*), 6.09 (2H, s, NC*H*_2_O), 3.64–3.59 (2H, m, OC*H*_2_CH_2_), 0.95–0.90 (2H, m, OCH_2_C*H*_2_Si), −0.04 (9H, s, Si(C*H*_3_)_3_); *δ*_C_ (176 MHz, chloroform-*d*) 143.1 (*C*-7), 140.9 (*C*-5), 134.4 (*C*-2′′,6′′), 134.3 (*C*-7a), 133.1 (*C*-3), 132.3 (*C*-3a), 129.4 (*C*-1′′), 129.3 (*C*-3′′,5′′), 129.1 (*C*-4′′), 111.6 (*C*-4), 79.9 (N*C*H_2_O), 66.7 (O*C*H_2_CH_2_Si), 17.9 (OCH_2_*C*H_2_Si), −1.3 (Si(*C*H_3_)_3_); *V*_max_ (ATR) 1078, 856, 833, 796, 689 cm^−1^; LC-MS (ES+) [M (^35^Cl) + H] 392.209 [M (^37^Cl) + H] 394.223; HRMS found [M + H]^+^ 392.1020, C_18_H_23_^35^ClN_3_OSSi requires *M* 392.1020.

### General procedure E − for deproto-metalation by TMPMgCl^.^LiCl and transmetalation to Zn for Negishi cross-coupling

An oven-dried reaction vessel was charged with a solution of the substrate (1.00 eq.) in dry THF (0.5 M), the atmosphere was exchange to nitrogen, and the solution cooled to −40 °C. TMPMgCl^.^LiCl in THF (2.00 eq.) was added dropwise and the reaction was stirred for 30 minutes at −40 °C. A solution of ZnCl_2_ (1.00 eq.) in THF (1 M) was added and the reaction stirred for 30 minutes at −40 °C. In a separate oven-dried reaction vessel, a solution of the corresponding (hetero)aryl halide (1.50 eq.) and Pd(PPh_3_)_4_ (0.05 eq.) in THF (0.5 M) were stirred at room temperature for 30 minutes. This solution was added to the main reaction mixture at −40 °C, then the reaction was stirred at room temperature overnight. The reaction was quenched with NH_4_Cl sat. solution and the crude product extracted with EtOAc, then the combined organic layers were washed with brine, dried over MgSO_4_, filtered, and concentrated under reduced pressure. The product was purified by silica gel flash column chromatography using the stated solvent system.

#### 4-(5′-Chloro-1′-((2′′-(trimethylsilyl)ethoxy)methyl)-1*H*-pyrazolo[3,4-*c*]pyridin-7′-yl)benzonitrile 34

General procedure E was applied to 5-chloro-1-{[2′-(trimethylsilyl)ethoxy]methyl}-1*H*-pyrazolo[3,4-*c*]pyridine 9a (0.100 g, 0.35 mmol, 1.00 eq.) with 4-iodobenzonitrile (0.121 g, 0.55 mmol, 1.50 eq.). Purification by reverse phase column chromatography (MeCN:H_2_O 50–100%) afforded 4-(5′-chloro-1′-((2′′-(trimethylsilyl)ethoxy)methyl)-1*H*-pyrazolo[3,4-*c*]pyridin-7′-yl)benzonitrile 34 as a cream solid (0.113 g, 0.29 mmol, 83%) with mp 90–94 °C.

*δ*_H_ (600 MHz, CDCl_3_) 8.13 (1H, s, 3′-*H*), 7.85 (2H, d, *J* = 7.9 Hz, 2,6-*H*), 7.81 (2H, d, *J* = 7.9 Hz, 3,5-*H*), 7.71 (1H, s, 4′H), 5.38 (2H, s, NC*H*_2_O), 3.40 (2H, m, OC*H*_2_CH_2_), 0.77 (2H, m, OCH_2_C*H*_2_Si), −0.07 (9H, s, Si(C*H*_3_)_3_); *δ*_C_ (151 MHz, CDCl_3_) 143.0 (*C*-7′), 141.4 (*C*-4), 140.9 (*C*-5′), 133.7 (*C*-3′a), 133.6 (*C*-7′a), 133.4 (*C*-3′), 132.1 (*C*-2,6), 130.2 (*C*-3,5), 118.3 (C≡N), 114.3 (*C*-4′), 113.4 (*C*-1), 78.4 (N*C*H_2_O), 66.8 (O*C*H_2_CH_2_), 17.7 (OCH_2_*C*H_2_Si), −1.5 (Si(*C*H_3_)_3_); *V*_max_ (ATR) 2230 (C≡N), 1070, 856, 837, 815 cm^−1^; LC-MS (ES+) [M (^35^Cl) + H] 385.330 [M (^37^Cl) + H] 387.307; HRMS found [M + H]^+^ 385.1250, C_19_H_22_^35^ClN_4_OSi requires M 385.1251.

## Author contributions

EVB designed and conducted experiments; EVB and PGS drafted the manuscripts; FE, GCC and PGS conceived the project, designed experiments, and supervised the work; PGS secured the funding. All authors reviewed and edited the manuscript and approved the final submitted version.

## Conflicts of interest

There are no conflicts to declare.

## Supplementary Material

RA-013-D3RA07458G-s001
