# Supplementary material for: Synthesis and vectorial functionalisation of pyrazolo[3,4-c]pyridines
Source: RSC Adv. 2023 Nov 23;13(49):34391–9. doi: 10.1039/d3ra07458g (PMC10667592; doi:10.1039/d3ra07458g)

Electronic Supplementary Material (ESI) for RSC Advances.
This journal is © The Royal Society of Chemistry 2023

Supporting Information

Synthesis and Vectorial Functionalisation of Pyrazolo[3,4-c]pyridines

Elizabeth V Bedwell, Flavio da Silva Emery, Giuliano C Clososki, Patrick G Steel

Table of Contents

Table 1: Optimisation of metalation of 9a with TMPMgCl.LiCl reaction with iodine 2

Experimental Procedures 3

NMR Spectra of all new compounds.. 27

Table 1: Optimisation of metalation of 9a with TMPMgCl.LiCl reaction with iodine

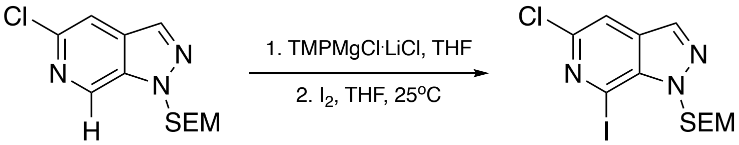

Entry	base eq.	Temperature with base	Time with base / minutes	Time with I_2 / minutes	Yield
1	2	0°C	120	60	0%
2	2	0°C	120	1080	0%
3	2	25°C	300	60	0%[a]
4	2	25°C	1080	60	0%[a]
5	1.2	-78°C	15	120	16%
6	1.5	-78°C	30	120	35%
7	2	-40°C	60	180	59%[a]
8	2	-40°C	15	60	77%[a]
9	2	-40°C	30	60	70%[a]
10	2	-40°C	60	60	81%[a]

[a]=conversion calculated by GCMS analysis of crude reaction mixture

Experimental Procedures

Materials and Methods

All solvents and reagents were purchased from commercial suppliers and used without further purification unless otherwise stated below. Final compound purification by flash column chromatography was performed on a CombiFlash® System from Teledyne Isco equipped with an UV-light detector using prepacked silica RediSep Rf cartridges with the stated solvent gradient. Crude mixtures to be purified were dry loaded onto silica (normal phase) or Celite® 545 (reverse phase) prior to running the column. NMR spectra were recorded on the following instruments: Bruker Neo 700 MHz spectrometer with operating frequencies of 700 MHz for ^{1}H and 175 MHz for ^{13}C; Varian VNMRS-600 with operating frequencies of 600 MHz for ^{1}H and 150 MHz for ^{13}C NMR; and Varian VNMRS-400 with operating frequencies of 400 MHz for ^{1}H and 376 MHz for ^{19}F NMR. Spectra were referenced relative to $CDCl_3$ (δ_H 7.26 ppm, δ_C 77.16 ppm), CD3OD (δ_H 4.87 ppm, δ_C 49.00 ppm), or DMSO (δ_H 2.50 ppm, δ_C 39.52 ppm). Chemical shifts are reported in parts per million (ppm), coupling constants (*J*) in hertz (Hz) and multiplicity as singlet (s), doublet (d), triplet (t), quartet (q), pentet (p), sextet (s), multiplet (m) or a combination thereof. All *J* values are $J_{H\text{-}H}$ unless otherwise stated. All ^{1}H NMR and ^{13}C NMR spectral assignments were made with the aid of ^{1}H^{1}H COSY, ^{1}H^{1}H NOESY, ^{1}H^{13}C HSQC and ^{1}H^{13}C HMBC NMR experiments. Infra-red spectra were recorded on a Perkin Elmer Paragon 1000 FT-IR spectrometer or a Perkin Elmer RX FT-IR spectrometer with Golden Gate Diamond ATR apparatus. IR assignments are reported in wavenumbers (cm^{-1}).
Melting points were recorded on Thermo Scientific Electrothermal IA9100 Digital Melting Point apparatus. Thin layer chromatography was performed using Merck F254 silica gel 60 aluminium sheets pre-coated with silica gel. High resolution mass spectrometry (HRMS) and liquid chromatography mass spectrometry (LC-MS) were recorded on a Waters TQD mass spectrometer ESI-LC water (0.1 % formic acid): MeCN/MeOH, flow rate 0.6 mL min^{-1} with a UPLC BEH C18 1.7 µm (2.1 mm x 50 mm) column. Gas chromatography mass spectrometry (GCMS) was carried out on a Shimadzu QP2010- Ultra with a temperature gradient 50 ˚C – 300 ˚C and a hold time of 5 mins, using a Rxi-17Sil MS (0.15 µm x 10 m x 0.15 mm) column.

Substrate synthesis

*Compound **3a***
1'-{5-chloro-*1H*-pyrazolo[3,4-c]pyridin-1-yl}ethan-1'-one

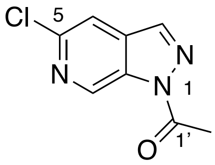

Ac_2O (33 mL, 0.35 mol, 10.0 eq) was added to a solution of 6-chloro-4-methylpyridin-3-amine **2a** (5.00 g, 35 mmol, 1.00 eq) in DCE (140 mL) and stirred at room temperature for 90 minutes under a nitrogen atmosphere. $NaNO_2$ (9.68 g, 0.14 mol, 4.00 eq) was added and the reaction mixture stirred at room temperature for 3 hours, then heated overnight at 90 ˚C. The reaction mixture was concentrated under reduced pressure then diluted with $NaHCO_3$ (150 mL). The product was extracted into EtOAc (5 x 100 mL) then washed with H_2O (4 x 100 mL) and brine (2 x 100 mL), dried over $MgSO_4$, filtered, and concentrated under reduced pressure to give 1'-{5-chloro-*1H*-pyrazolo[3,4-c]pyridin-1-yl}ethan-1'-one **3a** as a white solid (6.57 g, 34 mmol, 96%) with mp 138-139 ˚C. No further purification was necessary.

δ_H (400 MHz, Chloroform-*d*) 9.56 (1H, s, 7-*H*), 8.15 (1H, s, 3-*H*), 7.69 (1H, s, 4-*H*), 2.81 (3H, s, C(O)C*H₃*); δ_C (101 MHz, Chloroform-*d*) 170.3 (*C*=O), 144.7 (*C*-5), 138.0 (*C*-7), 137.8 (*C*-3), 134.7 (*C*-7a), 133.8 (*C*-3a), 114.8 (*C*-4), 22.6 (C(O)*C*H_3); V_{max} (ATR) 1729 (C=O), 1390, 1353, 634 cm^{-1}; LC-MS (ESI) [M (^{35}Cl) + H] 196.069, [M (^{37}Cl)+ H]

198.007, [M (^{35}Cl) + H – $COCH_3$] 154.035, [M (^{37}Cl) + H – $COCH_3$] 156.011; HRMS (ESI) found $[M+H]^+$ 196.0290, $C_8H_7N_3O^{35}Cl$ requires *M* 196.0278. The analytical data were consistent with the literature.[1]

Compound ***4a***
5-chloro-1H-pyrazolo[3,4-c]pyridine

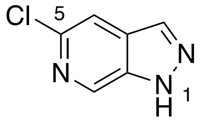

NaOMe (0.15 g, 2.8 mmol, 0.25 eq) was added to a solution of 1′-{5-chloro-1H-pyrazolo[3,4-c]pyridin-1-yl}ethan-1′-one **3a** (2.0 g, 10 mmol, 1.00 eq) in anhydrous MeOH (0.2 M, 50 mL) and stirred at room temperature for 15 minutes. The reaction was quenched by addition of HCl:MeOH 1:100 (8 mL) until acidic pH and concentrated under reduced pressure. The crude product was taken up in H_2O then adjusted to pH 10 by addition of aqueous NaOH then extracted with EtOAc. The combined organic layers were washed with brine, dried over $MgSO_4$, filtered and concentrated to afford 5-chloro-*1H*-pyrazolo[3,4-c]pyridine **4a** as a white solid (1.5 g, 10 mmol, 95%) with mp 225-226 ˚C.

δ_H (400 MHz, Methanol-d_4) 8.80 (1H, s, 7-H), 8.15 (1H, d, *J* = 1.2 Hz, 3-H), 7.82 (1H, d, *J* = 1.2 Hz, 4-H); δ_C (101 MHz, Methanol-d_4) 141.0 (*C*-5), 137.6 (*C*-7a), 135.1 (*C*-7), 134.2 (*C*-3), 131.2 (*C*-3a), 115.6 (*C*-4); V_{max} (ATR) 909, 736, 652 cm^{-1}; LC-MS (ESI) [M (^{35}Cl) + H] 154.113, [M (^{37}Cl)+ H] 156.128; HRMS (ESI) found $[M+H]^+$ 154.0167, $C_6H_5N_3{}^{35}Cl$ requires *M* 154.0172.

Compound ***3b***
1′-{5-bromo-*1H*-pyrazolo[3,4-c]pyridin-1-yl}ethan-1′-one

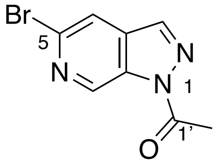

Ac_2O (2.5 mL, 27 mmol, 10.0 eq) was added to a solution of 6-bromo-4-methylpyridin-3-amine **2b** (0.500 g, 2.7 mmol) in DCE (10 mL) and stirred at room temperature for 45 minutes under a nitrogen atmosphere. $NaNO_2$ (0.738 g, 11 mmol, 4.00 eq) was added and the reaction mixture stirred at room temperature for 3 hours, then heated overnight at 90 ˚C. The reaction mixture was concentrated under reduced pressure then diluted with $NaHCO_3$ (40 mL). The product was extracted into EtOAc (4 x 25 mL) then washed with H_2O (2 x 25 mL) and brine (2 x 25 mL), dried over $MgSO_4$, filtered, and concentrated under reduced pressure to give 1′-{5-bromo-*1H*-pyrazolo[3,4-c]pyridin-1-yl}ethan-1′-one **3b** as a yellow solid (0.553 g, 2.3 mmol, 86%) mp 129-149 ˚C. No further purification was necessary.

δ_H (600 MHz, Chloroform-*d*) 9.54 (1H, t, *J* = 1.0 Hz, 7-*H*), 8.13 (1H, d, *J* = 1.0 Hz, 3-*H*), 7.85 (1H, d, *J* = 1.0 Hz, 4-*H*), 2.79 (3H, s, C(O)CH_3); δ_C (151 MHz, Chloroform-*d*) 170.2 (*C*=O), 138.5 (*C*-7), 137.5 (*C*-3), 135.0 (*C*-7a), 134.1 (*C*-1), 133.8 (*C*-3a), 118.7 (*C*-4), 22.5 (CH_3); V_{max} (ATR) 1711 (C=O), 1383, 1349, 889, 740 cm^{-1}; LC-MS (ESI) [M (^{79}Br) + H] 240.079, [M (^{81}Br)+ H] 242.093; HRMS (ESI) found $[M+H]^+$ 239.9796, $C_8H_7{}^{79}BrN_3O$ requires *M* 239.9772.

Compound ***4b***
5-bromo-*1H*-pyrazolo[3,4-c]pyridine

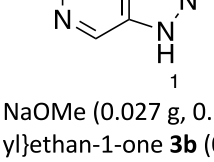

NaOMe (0.027 g, 0.50 mmol, 0.25 eq) was added to a solution of 1-{5-bromo-*1H*-pyrazolo[3,4-c]pyridin-1-yl}ethan-1-one **3b** (0.480 g, 2.0 mmol, 1.00 eq) in dry MeOH (8 mL) and stirred at room temperature for 30 minutes. The reaction was quenched by addition of HCl in MeOH (1:100) (5 mL), then concentrated under reduced pressure. The crude product was taken up in H_2O (25 mL) and adjusted to pH 10 by addition of

[1] D. Chapman and J. Hurst, *Journal of the Chemical Society, Perkin Transactions 1*, 1980, 2398-2404

aqueous NaOH. The product was extracted into EtOAc (3 x 25 mL) then washed with brine (25 mL), dried over MgSO4, filtered, and concentrated under reduced pressure. 5-bromo-*1H*-pyrazolo[3,4-c]pyridine **4b** (0.372 g, 1.9 mmol, 94%) was collected as a pale yellow solid with mp 238-239 ˚C without further purification.

δ_H (600 MHz, Methanol-*d*) 8.78 (1H, s, 7-*H*), 8.13 (1H, s, 3-*H*), 7.97 (1H, s 4-*H*); δ_C (151 MHz, Methanol-*d*) 136.5 (*C*-5), 134.2 (*C*-7), 132.6 (*C*-3), 130.0 (*C*-7a), 128.6 (*C*-3a), 118.2 (*C*-4); V_{max} (ATR) 1462, 952, 873, 785, 753 cm^{-1}; LC-MS (ESI) [M (^{79}Br) + H]198.083, [M (^{81}Br)+ H] 200.059; HRMS (ESI) found $[M+H]^+$ 197.9680, $C_6H_5{}^{79}BrN_3$ require *M* 197.9667.

Protecting Groups and Alkylations

Mesylate protection

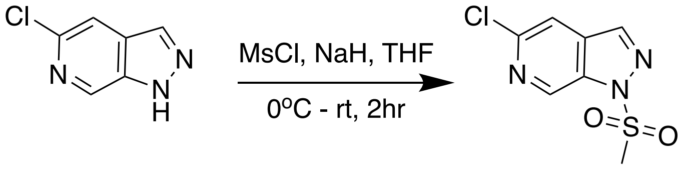

Compound ***5a***
5-chloro-1-methanesulfonyl-1H-pyrazolo[3,4-c]pyridine

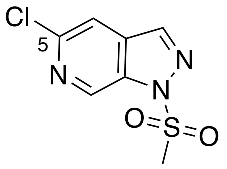

A solution of 5-chloro-*1H*-pyrazolo[3,4-c]pyridine **4a** (0.500 g, 5.3 mmol, 1.00 eq) in anhydrous THF (10 mL) was added slowly to a suspension of NaH (0.195 g, 8.1 mmol, 2.50 eq) in anhydrous THF (16 mL) at 0 ˚C under a nitrogen atmosphere. MsCl was added over 5 minutes then the reaction allowed to return to room temperature and stirred for 2 hours. The reaction was quenched with H_2O:propan-2-ol 1:1 (20 mL) then concentrated under reduced pressure. The reaction mixture was diluted with H_2O (20 mL) and extracted into DCM (4 x 20 mL). The combined organic layers were washed with brine (20 mL), dried over $MgSO_4$, filtered, and concentrated to afford 5-chloro-1-methanesulfonyl-*1H*-pyrazolo[3,4-c]pyridine **5a** as a yellow solid (0.695 g, 3.0 mmol, 92%) with mp 157-158 ˚C.

δ_H (600 MHz, Chloroform-*d*) 9.26 (1H, t, *J* = 1.0 Hz, 7-*H*), 8.31 (1H, d, *J* = 1.0 Hz, 3-*H*), 7.73 (1H, d, *J* = 1.0 Hz, 4-*H*), 3.39 (3H, s, SCH_3); δ_C (151 MHz, Chloroform-*d*) 144.6 (*C*-5), 139.0 (*C*-3), 135.9 (*C*-7), 135.8 (*C*-7a), 132.8 (*C*-3a), 114.9 (*C*-4), 41.5 (SCH_3); V_{max} (ATR) 1364, 557, 515 cm^{-1}; LC-MS (ESI) [M (^{35}Cl) + H] 232.097, [M (^{37}Cl)+ H] 234.150; HRMS (ESI) found $[M+H]^+$ 231.9941, $C_7H_7N_3SO_2{}^{35}Cl$ requires *M* 231.9948.

THP Protection

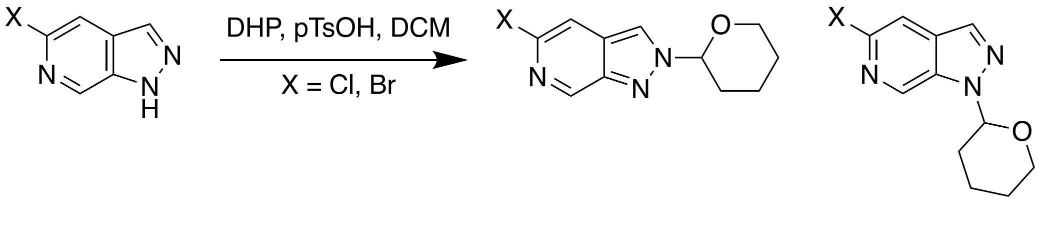

*Compound **6a***

5-bromo-1-(tetrahydropyran-2'-yl)-*1H*-pyrazolo[3,4-c]pyridine

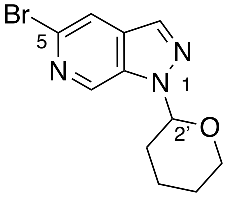

DHP (0.28 mL, 3.0 mmol, 3.00 eq) then pTsOH·H_2O (0.019 g, 0.10 mmol, 0.100 eq) were added slowly to a solution of 5-bromo-*1H*-pyrazolo[3,4-c]pyridine **4b** (0.200 g, 1.0 mmol) in DCM (7 mL) over ice. After 5 minutes the reaction mixture was allowed to return to room temperature, then stirred for 2 hours. The reaction was diluted with DCM (20 mL) then washed with sat. $NaHCO_3$ (3 x 25 mL) and brine (20 mL), dried over $MgSO_4$, filtered, and concentrated under reduced pressure to a yellow oil. Purification by silica gel flash column chromatography (EtOAc:Pet Ether 40-60 0-25%) afforded 5-bromo-1-(tetrahydropyran-2'-yl)-*1H*-pyrazolo[3,4-c]pyridine **6a** as a white solid (0.233 g, 0.83 mmol, 82%) with mp 125-127 ˚C.

δ_H (600 MHz, Chloroform-*d*) 8.91 (1H, t, *J* = 1.0 Hz, 7-*H*), 8.00 (1H, d, *J* = 1.0 Hz, 3-*H*), 7.80 (1H, d, *J* = 1.0 Hz, 4-*H*), 5.80 (1H, dd, *J* = 9.2, 2.9 Hz, 2'-*H*), 3.97 (1H, ddt, *J* = 9.2, 5.4, 2.5 Hz, 6'-*H*), 3.82-3.73 (1H, m, 6'-*H*), 2.54 – 2.40 (1H, m, 3'-*H*), 2.20 – 2.08 (1H, m, 3'-*H*), 2.20 – 2.08 (1H, m, 5'-*H*), 1.85 – 1.46 (2H, m, 4'-*H*), 1.85 – 1.46 (1H, m, 5'-*H*); δ_C (151 MHz, Chloroform-*d*) 135.8 (*C*-5), 134.9 (*C*-7), 132.2 (*C*-3), 131.8 (*C*-7a), 130.6 (*C*-3a), 118.4 (*C*-74), 86.6 (*C*-2'), 67.3 (*C*-6'), 29.6 (*C*-3'), 25.0 (*C*-4'), 22.0 (*C*-5'); V_{max} (ATR) 1465, 1047, 911, 733 cm^{-1}; LC-MS (ESI) [M (^{79}Br) + H] 282.150, [M (^{81}Br)+ H] 283.963, [M (^{79}Br) + H – C_5H_9O] 198.121, [M (^{81}Br) + H – C_5H_9O] 200.097; HRMS (ESI) found $[M+H]^+$ 282.0249, $C_{11}H_{13}{}^{79}BrN_3O$ requires *M* 282.0242.

*Compound **6b***

5-bromo-2-(tetrahydropyran-2'-yl)-*2H*-pyrazolo[3,4-c]pyridine

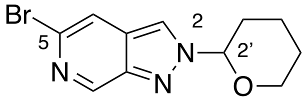

DHP (0.55 mL, 6.1 mmol, 3.00 eq) then pTsOH·H_2O (0.038 g, 0.20 mmol, 0.10 eq) were added slowly to a solution of 5-bromo-*1H*-pyrazolo[3,4-c]pyridine **4b** (0.400 g, 2.0 mmol, 1.00 eq) in DCM (15 mL) over ice. After 5 minutes the reaction mixture was allowed to return to room temperature, then stirred for 2 hours. The reaction was diluted with DCM (20 mL) then washed with sat. $NaHCO_3$ (3 x 25 mL) and brine (20 mL), dried over $MgSO_4$, filtered, and concentrated under reduced pressure to a yellow oil. Purification by silica gel flash column chromatography (EtOAc:Pet Ether 40-60 0-40%) afforded 5-bromo-2-(tetrahydropyran-2'-yl)-*2H*-pyrazolo[3,4-c]pyridine **6b** as a white solid (0.426 g, 1.5 mmol, 75%) with mp 58-60 ˚C. 5-bromo-1-(tetrahydropyran-2'-yl)-*1H*-pyrazolo[3,4-c]pyridine **6a** (0.035 g, 0.13 mmol, 6.2%) was also collected. $R_{f,\mathbf{6a}}$ = 0.61 and $R_{f,\mathbf{6b}}$ = 0.37 in EtOAc:Hexanes 1:2.

δ_H (600 MHz, Chloroform-*d*) 9.05 (1H, t, *J* = 1.0 Hz, 7-*H*), 8.19 (1H, d, *J* = 1.0 Hz, 3-*H*), 7.76 (1H, d, *J* = 1.0 Hz, 4-*H*), 5.73 (1H, dd, *J* = 9.2, 2.9 Hz, 2'-*H*), 4.12 (1H, ddt, *J* = 9.2, 5.4, 2.5 Hz, 6'-*H*), 3.80 (1H, ddd, *J* = 11.7, 9.9, 3.3 Hz, 6'-*H*), 2.31 – 2.25 (1H, m, 3'-*H*), 2.18 – 2.08 (1H, m, 3'-*H*), 2.07 – 2.01 (1H, m, 5'-*H*), 1.83 – 1.66 (2H, m, 4'-*H*), 1.83 – 1.66 (1H, m, 5'-*H*); δ_C (151 MHz, Chloroform-*d*) 144.9 (*C*-7), 144.4 (*C*-5), 130.0 (*C*-7a), 126.3 (*C*-3a), 120.4 (*C*-3), 117.3 (*C*-4), 89.7 (*C*-2'), 68.0 (*C*-6'), 31.6 (*C*-3'), 24.9 (*C*-4'), 21.8 (*C*-5'); V_{max} (ATR) 1471, 1147, 1092, 1044, 918 cm^{-1}; LC-MS (ESI) [M (^{79}Br) + H] 282.112, [M (^{81}Br)+ H] 283.898, [M (^{79}Br) + H – C_5H_9O] 198.083, [M (^{81}Br) + H – C_5H_9O] 200.097; HRMS (ESI) found $[M+H]^+$ 282.0250, $C_{11}H_{13}{}^{79}BrN_3O$ requires *M* 282.0242.

*Compound **7***

DHP (0.36 mL, 3.9 mmol, 3.00 eq) then *p*TsOH (0.025 g, 0.13 mmol, 0.10 eq) in DCM (5 mL) were added slowly to a solution of 5-chloro-*1H*-pyrazolo[3,4-c]pyridine **XX** (0.200 g, 1.3 mmol, 1.00 eq) in DCM (3 mL) over ice. The reaction mixture was stirred for 5 minutes then allowed to return to room temperature and stirred for 3 hours. The reaction mixture was diluted with DCM (25 mL) then washed with sat. $NaHCO_3$ (3 x 20 mL), dried over

$MgSO_4$, filtered, and concentrated. Purification by silica gel flash column chromatography (EtOAc:Pet Ether 40-60 0-50%) afforded 5-chloro-2-(tetrahydropyran-2'-yl)-*2H*-pyrazolo[3,4-c]pyridine **7b** (0.206 g, 0.87 mmol, 66%) as a yellow oil and 5-chloro-1-(tetrahydropyran-2'-yl)-*1H*-pyrazolo[3,4-c]pyridine **7a** as a white solid (0.0434 g, 0.18 mmol, 14%) with mp 89-93 ˚C. . $R_{f,\mathbf{7a}}$ = 0.76 and $R_{f,\mathbf{7b}}$ = 0.57 in EtOAc:Hexanes 1:1.

*Compound **7a***
5-chloro-1-(tetrahydropyran-2'-yl)-*1H*-pyrazolo[3,4-c]pyridine

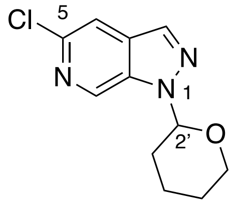

δ_H (600 MHz, Chloroform-*d*) 8.91 (1H, t, *J* = 1.0 Hz, 7-*H*), 7.99 (1H, d, *J* = 0.8 Hz, 3-*H*), 7.62 (1H, d, *J* = 1.1 Hz, 4-*H*), 5.79 (1H, dd, *J* = 8.7, 2.7 Hz, 2'-*H*), 3.97 (1H, dtd, *J* = 11.7, 4.0, 1.5 Hz, 6'-*H*), 3.76 (1H, ddd, *J* = 11.7, 9.3, 3.4 Hz, 6'-*H*), 2.49 – 2.41 (1H, m, 3'-*H*), 2.16 – 2.08 (1H, m, 3'-*H*), 2.16 – 2.08 (1H, m, 4'-*H*), 1.81 – 1.74 (1H, m, 4'-*H*), 1.76 – 1.69 (1H, m, 5'-*H*), 1.72 – 1.67 (1H, m, 5'-*H*); δ_C (151 MHz, Chloroform-*d*) 141.3 (*C*-5), 135.5 (*C*-7a), 134.4 (*C*-7), 132.4 (*C*-3), 131.6 (*C*-3a), 114.4 (*C*-4), 86.6 (*C*-2'), 67.3 (*C*-6'), 29.6 (*C*-3'), 25.0 (*C*-5'), 22.0 (*C*-4'); V_{max} (ATR) 1466, 1418, 1063, 871 cm^{-1}; LC-MS (ESI) [M (^{35}Cl) + H] 238.178, [M (^{37}Cl)+ H] 240.193, [M (^{35}Cl) + H – C_5H_9O] 154.073, [M (^{37}Cl) + H – C_5H_9O] 156.087; HRMS (ESI) found $[M+H]^+$ 238.0759, $C_{11}H_{13}{}^{35}ClN_3O$ requires *M* 238.0747.

*Compound **7b***
5-chloro-2-(tetrahydropyran-2'-yl)-*2H*-pyrazolo[3,4-c]pyridine

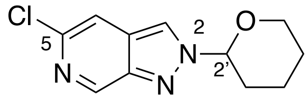

δ_H (600 MHz, Chloroform-*d*) 9.07 (1H, t, *J* = 1.1 Hz, 7-*H*), 8.19 (1H, d, *J* = 1.1 Hz, 3-*H*), 7.57 (1H, d, *J* = 1.1 Hz, 4-*H*), 5.73 (1H, dd, *J* = 9.1, 2.9 Hz, 2'-*H*), 4.12 (1H, dtd, *J* = 11.7, 3.8, 1.6 Hz, 6'-*H*), 3.80 (1H, ddd, *J* = 11.7, 9.9, 3.3 Hz, 6'-*H*), 2.31 – 2.26 (1H, m, 3'-*H*), 2.14 (1H, dddd, *J* = 13.2, 11.0, 9.1, 4.2 Hz, 3'-*H*), 2.07 – 2.02 (1H, m, 4'-*H*), 1.82 – 1.72 (1H, m, 4'-*H*/5'-*H*), 1.82 – 1.72 (1H, m, 4'-*H*/5'-*H*), 1.72 – 1.70 (1H, m, 6'-*H*); δ_C (151 MHz, Chloroform-*d*) 144.7 (*C*-7), 144.3 (*C*-5), 140.7(*C*-7a), 125.9 (*C*-3a), 120.6 (*C*-3), 113.2 (*C*-4), 89.7 (*C*-2'), 68.0 (*C*-6'), 31.6 (*C*-3'), 24.9 (*C*-5'), 21.8 (*C*-4'); V_{max} (ATR) 1475, 1053 (C-O), 724 cm^{-1}; LC-MS (ESI) [M (^{35}Cl) + H] 238.221, [M (^{37}Cl)+ H] 240.273, [M (^{35}Cl) + H – C_5H_9O] 154.113, [M (^{37}Cl) + H – C_5H_9O] 156.116; HRMS (ESI) found $[M+H]^+$ 238.0736, $C_{11}H_{13}{}^{35}ClN_3O$ requires *M* 238.0747.

SEM Protection Route 1

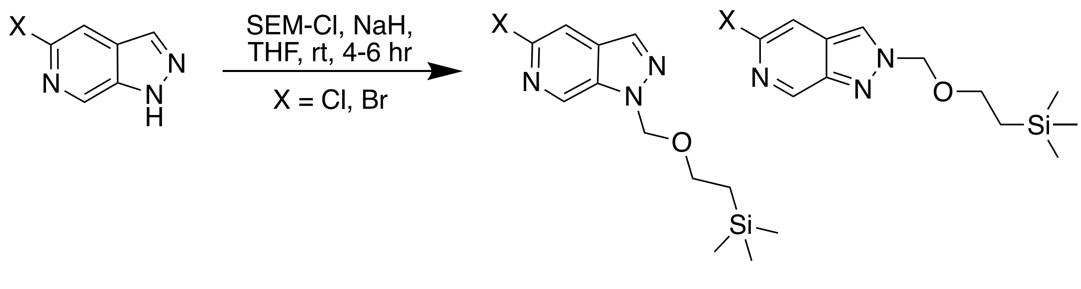

A solution of the starting *1H*-pyrazolo[3,4-c]pyridine **4a** (1.00 eq) in anhydrous THF was added slowly to a suspension of NaH (1.50 eq) in anhydrous THF at 0 ˚C under a nitrogen atmosphere. The mixture was stirred at 0 ˚C for 30 minutes then SEM-Cl (1.50 eq) was added dropwise. The reaction mixture was allowed to return to room temperature then stirred until no starting material remained. The reaction was quenched by addition of H_2O:propan-2-ol 1:1 then concentrated under reduced pressure. The crude product was diluted with H_2O,

extracted with DCM, washed with brine, dried over $MgSO_4$, filtered, and concentrated. Purification by silica gel flash column chromatography with the stated solvent system afforded the two product isomers.

SEM Protection Route 2

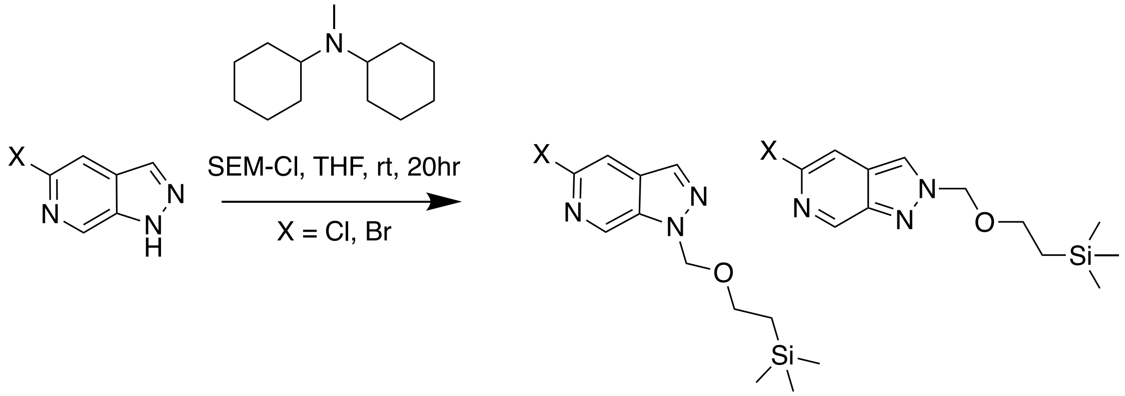

Dicyclohexylmethylamine (1.20 eq) then SEM-Cl (1.20 eq) were added to a solution of the starting *1H*-pyrazolo[3,4-c]pyridine **4a** (1.00 eq) in anhydrous THF nitrogen and the reaction mixture was stirred at room temperature overnight. The reaction was quenched by addition of NaOH solution then concentrated under reduced pressure to remove the THF. The crude product was extracted with EtOAc, washed with brine, dried over $MgSO_4$, filtered, and concentrated. The purification by silica gel flash column chromatography with the stated solvent system afforded the two product isomers.

*Compound **8***

SEM protection route 1 was applied to 5-bromo-*1H*-pyrazolo[3,4-c]pyridine **4b** (1.00 g, 5.1 mmol, 1.00 eq). Purification by silica gel flash column chromatography (EtOAc:Pet Ether 40-60 0-50%) afforded compound **8a** (0.785 g, 2.4 mmol, 47%) as a colourless oil and compound **8b** (0.435 g, 1.3 mmol, 26%) as a yellow solid with mp 61-62 ˚C. $R_{f,\mathbf{8a}}$ = 0.78, $R_{f,\mathbf{8b}}$ = 0.43 in EtOAc:Pet Ether 40-60 1:4.

SEM protection route 2 was applied to 5-bromo-*1H*-pyrazolo[3,4-c]pyridine **4b** (0.200 g, 1.0 mmol, 1.00 eq) then purification by silica gel flash column chromatography (EtOAc:Pet Ether 40-60 0-50%) afforded compound **8a** (0.060 g, 0.18 mmol, 18%) as a colourless oil and compound **8b** (0.105 g, 0.32 mmol, 32%) as a yellow solid with mp 61-62 ˚C. $R_{f,\mathbf{8a}}$ = 0.78, $R_{f,\mathbf{8b}}$ = 0.43 in EtOAc:Pet Ether 40-60 1:4.

*Compound **8a***
5-bromo-1-{[2-(trimethylsilyl)ethoxy]methyl}-*1H*-pyrazolo[3,4-c]pyridine

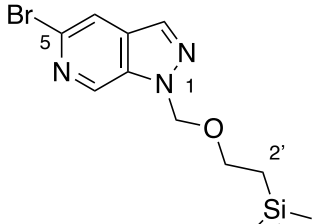

δ_H (600 MHz, Chloroform-*d*) 8.89 (1H, t, *J* = 1.0 Hz, 7-*H*), 8.01 (1H, d, *J* = 1.0 Hz, 3-*H*), 7.84 (1H, d, *J* = 1.0 Hz, 4-*H*), 5.79 (2H, s, NCH_2O), 3.56 – 3.50 (2H, m, OCH_2CH_2), 0.91 – 0.85 (2H, m, OCH_2CH_2Si), -0.07 (9H, s, $Si(CH_3)_3$); δ_C (151 MHz, Chloroform-*d*) 135.9 (*C*-5), 134.0 (*C*-7), 132.5 (*C*-3), 131.9 (*C*-7a), 131.9 (*C*-3a), 118.4 (*C*-4), 78.6 (NCH_2O), 67.0 (OCH_2CH_2), 17.7 (CH_2CH_2Si), -1.5 (SiCH3); V_{max} (ATR) 1467, 1088, 865, 839, 779 cm^{-1}; LC-MS (ESI) [M(^{79}Br) +H] 328.211, [M(^{81}Br)+H] 329.959; HRMS (ESI) found $[M+H]^+$ 328.0478, $C_{12}H_{19}{}^{79}BrN_3OSi$ requires *M* 328.0481.

*Compound **8b***
5-bromo-2-{[2'-(trimethylsilyl)ethoxy]methyl}-*2H*-pyrazolo[3,4-c]pyridine

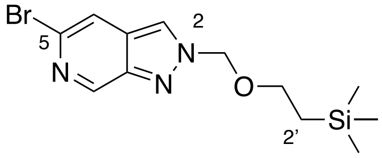

δ_H (600 MHz, Chloroform-*d*) 9.08 (1H, t, *J* = 1.1 Hz, 7-*H*), 8.13 (1H, d, *J* = 1.1 Hz, 3-*H*), 7.78 (1H, d, *J* = 1.1 Hz, 4-*H*), 5.77 (2H, s, NCH_2O), 3.66 – 3.61 (2H, m, OCH_2CH_2), 0.98 – 0.91 (2H, m, OCH_2CH_2Si), -0.03 (9H, s, Si(CH_3)$_3$); δ_C (151 MHz, Chloroform-*d*) 144.9 (*C*-7), 144.5 (*C*-5), 130.2 (*C*-7a), 126.8 (*C*-3a), 121.6 (*C*-3), 117.1 (*C*-4), 82.6 (NCH_2O), 68.2 (OCH_2CH_2), 17.8 (CH_2CH_2Si), -1.5 (SiCH3); V_{max} (ATR) 1105, 1041, 914, 840, 735 cm^{-1}; LC-MS (ESI) [M(^{79}Br) +H] 328.211, [M(^{81}Br)+H] 329.997; HRMS (ESI) found [M+H]$^+$ 328.0485, $C_{12}H_{19}{}^{79}BrN_3OSi$ requires *M* 328.0481.

*Compound **9***

SEM protection route 1 was applied to 5-chloro-*1H*-pyrazolo[3,4-c]pyridine **4a** (0.200 g, 1.3 mmol, 1.00 eq) then purification by silica gel flash column chromatography (EtOAc:Hexanes 0-25%) afforded compound **9a** (0.167 g, 0.59 mmol, 45%) as a colourless oil and compound **9b** (0.108 g, 0.38 mmol, 29%) as a yellow solid with mp 66.8-67.2 ˚C. $R_{f,\mathbf{9a}}$ = 0.49, $R_{f,\mathbf{9b}}$ = 0.32 in EtOAc:Hexane 1:4.

SEM protection route 2 was applied to 5-chloro-*1H*-pyrazolo[3,4-c]pyridine **4a** (0.330 g, 2.1 mmol, 1.00 eq) then purification by silica gel flash column chromatography (EtOAc:Hexanes 0-25%) afforded compound **9a** (0.127 g, 0.45 mmol, 21%) as a colourless oil and compound **9b** (0.270 g, 0.95 mmol, 44%) as a yellow solid with mp 66.8-67.2 ˚C. $R_{f,\mathbf{9a}}$ = 0.49, $R_{f,\mathbf{9b}}$ = 0.32 in EtOAc:Hexane 1:4.

*Compound **9a***
5-chloro-1-{[2'-(trimethylsilyl)ethoxy]methyl}-1H-pyrazolo[3,4-c]pyridine

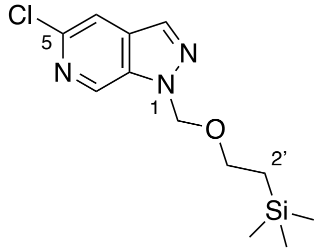

δ_H (400 MHz, Chloroform-*d*) 8.92 (1H, t, *J* = 1.0 Hz, 7-H), 8.04 (1H d, *J* = 1.0 Hz, 3-H), 7.70 (1H, d, *J* = 1.0 Hz, 4-H), 5.82 (2H, s, NCH_2O), 3.60 – 3.51 (2H, m, OCH_2C), 0.96 – 0.86 (2H, m, CH_2Si), -0.05 (9H, s, Si(CH_3)$_3$); δ_C (101 MHz, Chloroform-*d*) 141.7 (*C*-5), 135.7 (*C*-7), 133.7 (*C*-7a), 132.8 (*C*-3), 131.8 (*C*-3a), 114.6 (*C*-4), 78.7 (NCH_2O), 67.2 (OCH_2CH_2), 17.8 (CH_2Si(CH_3)$_3$), -1.4 (Si(CH_3)$_3$); V_{max} (ATR) 1469, 1079, 1061, 834, 801, 785 cm^{-1}; LC-MS (ESI) [M (^{35}Cl) + H] 284.245, [M (^{37}Cl)+ H] 286.260; HRMS (ESI) found [M+H]$^+$ 284.0992, $C_{12}H_{19}N_3OSi^{35}Cl$ requires *M* 284.0986.

*Compound **9b***
5-chloro-2-{[2'-(trimethylsilyl)ethoxy]methyl}-2H-pyrazolo[3,4-c]pyridine

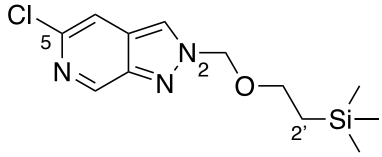

δ_H (400 MHz, Chloroform-*d*) 9.10 (1H, t, *J* = 1.1 Hz, 7-H), 8.14 (1H, d, *J* = 1.1 Hz, 3-H), 7.60 (1H, d, *J* = 1.1 Hz, 4-H), 5.77 (2H, s, NCH_2O), 3.68 – 3.59 (2H, m, OCH_2CH_2), 1.00 – 0.90 (2H, m, CH_2Si), -0.03 (9H, s, Si(CH_3)$_3$); δ_C (101 MHz, Methanol-d4) 145.7 (*C*-5), 145.3 (*C*-7), 141.0 (*C*-7a), 127.8 (*C*-3a), 125.4 (*C*-3), 115.1 (*C*-4), 83.6 (NCH_2O), 68.9 (OCH_2CH_2), 18.6 (CH_2Si), -1.5 (Si(CH_3)$_3$); V_{max} (ATR) 1249, 1088, 1054, 854, 832 cm^{-1}; LCMS (ESI) [M (^{35}Cl) + H] 284.245, [M (^{37}Cl)+ H] 286.260; HRMS (ESI) found [M+H]$^+$ 284.0989, $C_{12}H_{19}N_3OSi^{35}Cl$ requires *M* 284.0986.

Methylation

*Compound **10***

A cooled solution of 5-bromo-*1H*-pyrazolo[3,4-c]pyridine **4b** (0.500 g, 2.5 mmol, 1.00 eq) and NaH (0.121 g, 3.0 mmol, 1.20 eq) in THF (13 mL) was stirred for 30 minutes on ice under a nitrogen atmosphere. MeI (0.24 mL, 3.8 mmol, 1.50 eq) was added and the reaction mixture was allowed to return to room temperature. After 45 minutes the reaction was quenched with H_2O:propan-2-ol 1:1 (30 mL) then concentrated under reduced pressure. The product was extracted with DCM (4 x 25 mL) and the combined organic layers were washed with brine (20 mL), dried over $MgSO_4$, filtered, and concentrated under reduced pressure to form a yellow solid. Purification by silica gel flash column chromatography (EtOAc:Pet Ether 40-60 0-75%) gave 5-bromo-1-methyl-*1H*-pyrazolo[3,4-c]pyridine **10a** (0.191 g, 0.90 mmol, 36%) as a white solid with mp 109-110 ˚C, and 5-bromo-2-methyl-*2H*-pyrazolo[3,4-c]pyridine **10b** (0.268 g, 1.3 mmol, 50%) as a white solid with mp 175-178 ˚C. $R_{f,\mathbf{10a}}$ = 0.17 and $R_{f,\mathbf{10b}}$ = 0.58 in EtOAc:Pet Ether 40-60 1:1.

*Compound **10a***
5-bromo-1-methyl-*1H*-pyrazolo[3,4-c]pyridine

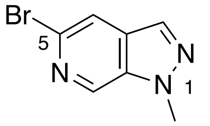

δ_H (700 MHz, Chloroform-*d*) 8.65 (1H, d, *J* = 1.0 Hz, 7-*H*), 7.91 (1H, d, *J* = 1.0 Hz, 3-*H*), 7.74 (1H, t, *J* = 1.0 Hz, 4-*H*), 4.11 (3H, s, NC*H*3); δ_C (176 MHz, Chloroform-*d*) 136.3 (*C*-5), 133.2 (*C*-7), 131.5 (*C*-3), 130.8 (*C*-7a), 130.0 (*C*-3a), 118.2 (*C*-4), 36.4 (*C*H3); V_{max} (ATR) 1473, 1050, 765, 640, 428 cm^{-1}; LC-MS (ESI) [M(^{79}Br) +H] 212.031, [M(^{81}Br)+H] 214.045; HRMS (ESI) found $[M+H]^+$ 211.9835, $C_7H_7{}^{79}BrN_3$ requires *M* 211.9823.

*Compound **10b***
5-bromo-2-methyl-*2H*-pyrazolo[3,4-c]pyridine

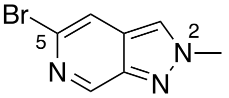

δ_H (700 MHz, Chloroform-*d*) 8.93 (1H, s, 7-*H*), 7.85 (1H, s, 3-*H*), 7.64 (1H, s, 4-*H*), 4.22 (3H, s, NC*H*3); δ_C (176 MHz, Chloroform-*d*) 144.8 (*C*-5), 143.9 (*C*-7), 129.9 (*C*-7a), 127.0 (*C*-3a), 122.8 (*C*-3), 116.7 (*C*-4), 41.2 (N*C*H3); V_{max} (ATR) 1154, 1043, 902, 489, 441 cm^{-1}; LC-MS (ESI) [M(^{79}Br) +H] 212.039, [M(^{81}Br)+H] 214.025; HRMS (ESI) found $[M+H]^+$ 211.9837, $C_7H_7{}^{79}BrN_3$ requires *M* 211.9823.

*Compound **11***

A cooled solution of 5-chloro-*1H*-pyrazolo[3,4-c]pyridine **4a** (0.100 g, 0.65 mmol, 1.00 eq) and NaH (0.031 g, 0.78 mmol, 1.20 eq) in THF (3 mL) was stirred for 30 minutes on ice under a nitrogen atmosphere. MeI (0.06 mL, 0.98 mmol, 1.50 eq) was added and the reaction mixture was allowed to return to room temperature. After 45 minutes the reaction was quenched with H_2O:IPA 1:1 (10 mL) then concentrated under reduced pressure. The product was extracted with DCM (3 x 15 mL) and the combined organic layers were washed with brine (20 mL), dried over $MgSO_4$, filtered, and concentrated under reduced pressure to form a yellow solid. Purification by silica gel flash column chromatography (EtOAc:Pet Ether 40-60 0-60%) gave 5-chloro-1-methyl-*1H*-pyrazolo[3,4-c]pyridine **11a** (0.033 g, 0.20 mmol, 31%) as a white solid with mp 102-104 ˚C, and 5-chloro-2-methyl-*2H*-pyrazolo[3,4-c]pyridine **11b** as a white solid (0.046 g, 0.28 mmol, 42%) with mp 134-146°C. $R_{f,\mathbf{11a}}$ = 0.50 and $R_{f,\mathbf{11b}}$ = 0.18 in EtOAc:Pet Ether 1:2.

Compound ***11a***
5-chloro-1-methyl-*1H*-pyrazolo[3,4-c]pyridine

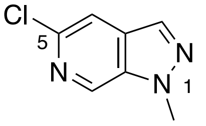

δ_H (600 MHz, Chloroform-*d*) 8.70 (1H, d, *J* = 1.0 Hz, 7-*H*), 7.95 (1H, d, *J* = 1.0 Hz, 3-*H*), 7.61 (1H, t, *J* = 1.0 Hz, 4-*H*), 4.16 (3H, s, NC*H*3); δ_C (151 MHz, Chloroform-*d*) 140.7 (*C*-5), 136.0 (*C*-7a), 132.7 (*C*-7), 131.6 (*C*-3), 130.6 (*C*-3a), 114.2 (*C*-4), 36.3 (*C*H3); V_{max} (ATR) 1545, 1539, 1346, 713 cm^{-1}; LC-MS (ESI) [M(^{35}Cl) +H] 168.059, [M(^{37}Cl)+H] 170.073; MS (ESI) found $[M+H]^+$ 168.0338 $C_7H_8{}^{35}ClN_3$ requires *M* 168.0323. The analytical data were consistent with the literature .[2]

Compound ***11b***
5-chloro-2-methyl-*2H*-pyrazolo[3,4-c]pyridine

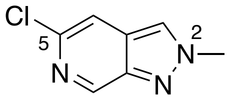

δ_H (600 MHz, Chloroform-*d*) 9.01 (1H, t, *J* = 1.0 Hz, 7-*H*), 7.90 (1H, d, *J* = 1.0 Hz 3-*H*), 7.53 (1H, d, *J* = 1.0 Hz, 4-*H*), 4.27 (3H, s, NC*H*3); δ_C (151 MHz, Chloroform-*d*) 144.7 (*C*-5), 143.6 (*C*-7), 140.5 (*C*-7a), 126.6 (*C*-3a), 122.9 (*C*-3), 112.6 (*C*-4), 41.1 (N*C*H3); V_{max} (ATR) 1545, 1539, 1346, 713 cm^{-1}; LC-MS (ESI) [M(^{35}Cl) +H] 168.059, [M(^{37}Cl)+H] 170.111; HRMS (ESI) found $[M+H]^+$ 168.0334 $C_7H_8{}^{35}ClN_3$ requires *M* 168.0323. The analytical data were consistent with the literature.[3]

Propylation

Compound ***12***

A cooled solution of 5-chloro-*1H*-pyrazolo[3,4-c]pyridine **4a** (0.200 g, 1.3 mmol, 1.00 eq) and NaH (0.047 g, 1.6 mmol, 1.20 eq) in THF (6.5 mL) was stirred for 30 minutes on ice under a nitrogen atmosphere. 1-iodopropane (0.19 mL, 2.0 mmol, 1.50 eq) was added and the reaction mixture was allowed to return to room temperature. After stirring overnight, the reaction was quenched with NH_4Cl (25 mL) then volatiles were removed under reduced pressure. The product was extracted with EtOAc (3 x 30 mL), and the combined organic layers washed with brine (30 mL), dried over $MgSO_4$, filtered, then concentrated under reduced pressure to give the crude product as an orange solid. The final products were purified by silica gel column chromatography (EtOAc:Hexanes 0-35%) affording compound **12a** as a white solid (0.068g, 0.35 mmol, 27%) with mp 53-55 ˚C and compound **12b** as a yellow solid (0.089g, 0.46 mmol, 35%) mp 56-60 ˚C. $R_{f,\mathbf{12a}}$ = 0.48 and $R_{f,\,\mathbf{12b}}$ = 0.31 in EtOAc:Hexanes 1:3.

Compound ***12a***
5-chloro-1-propyl-*1H*-pyrazolo[3,4-c]pyridine

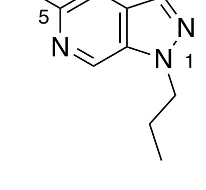

δ_H (400 MHz, Chloroform-*d*) 8.73 (1H, s, 7-*H*), 7.99 (1H, s, 3-*H*), 7.63 (1H, s, 4-*H*), 4.42 (2H, t, *J* = 7.3 Hz, NC*H*2CH2), 1.98 (2H, s, *J* = 7.3 Hz, C*H*2CH3), 0.92 (3H, t, *J* = 7.3 Hz, CH2C*H*3); δ_C (101 MHz, Chloroform-*d*) 140.6 (*C*-

[2] A. Papastathopoulos *et al*, *European Journal of Medicinal Chemistry*, **2021** (218), 113387
[3]

5), 135.7 (*C*-7a), 132.8 (*C*-7), 131.6 (*C*-3), 130.5 (*C*-3a), 114.3 (*C*-4), 51.5 (N*C*H_2CH_2), 23.4 (*C*H_2CH_3), 11.3 (CH_2*C*H_3); LC-MS (ESI) [M(^{35}Cl)+H] 196.145, [M(^{37}Cl)+H] 198.121; HRMS found $[M+H]^+$ 196.0643, $C_9H_{11}N_3{}^{35}Cl$ requires *M* 196.0642.

*Compound **12b***
5-chloro-2-propyl-*2H*-pyrazolo[3,4-c]pyridine

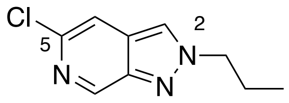

δ_H (400 MHz, Chloroform-*d*) 9.03 (1H, t, *J* = 1.1 Hz 7-*H*), 7.92 (1H, d, *J* = 1.1 Hz, 3-*H*), 7.54 (1H, d, *J* = 1.1 Hz, 4-*H*), 4.43 (2H, t, *J* = 7.3 Hz, NC*H_2*CH_2), 2.06 (2H, s, *J* = 7.3 Hz, C*H_2*CH_3), 0.95 (3H, t, *J* = 7.3 Hz, CH_2C*H_3*); δ_C (101 MHz, Chloroform-*d*) 144.6 (*C*-5), 143.8 (*C*-7), 140.3 (*C*-7a), 126.3 (*C*-3a), 122.1 (*C*-3), 112.7 (*C*-4), 56.2 (N*C*H_2CH_2), 23.9 (*C*H_2CH_3), 11.1 (CH_2*C*H_3); LC-MS (ESI) [M(^{35}Cl)+H] 196.145, [M(^{37}Cl)+H] 198.121; HRMS found $[M+H]^+$ 196.0644, $C_9H_{11}N_3{}^{35}Cl$ requires *M* 196.0642.

Buchwald-Hartwig Amination

General Procedure A
For Buchwald-Hartwig Amination

Pd_2dba_3 (0.05 eq), *rac*-BINAP (0.12 eq), NaO^{t}Bu (3.00 eq), and the stated protected pyrazolo[3,4-c]pyridine (1.00 eq) were sealed under a nitrogen atmosphere. The stated amine (1.10 eq) was added under nitrogen, followed by dry THF (0.1M). The deep red solution was stirred overnight at 55 ˚C until LCMS analysis confirmed complete conversion of the starting substrate. The reaction was cooled to room temperature, diluted with EtOAc and filtered through Celite®, washing the cake with additional EtOAc. This solution was concentrated under reduced pressure.
The product was purified by silica gel flash column chromatography using the stated solvent system.

*Compound **13***
4-[2’-(oxan-2’’-yl)-*2H*-pyrazolo[3,4-c]pyridin-5’-yl]morpholine

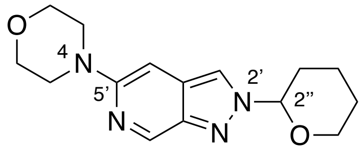

General procedure A was applied to 5-bromo-2-(oxan-2’-yl)-*2H*-pyrazolo[3,4-c]pyridine **6b** (0.080 g, 0.28 mmol, 1.00 eq) with morpholine (0.03 mL, 0.31 mmol, 1.10 eq). After purification by silica gel flash column chromatography (EtOAc:Pet Ether 40-60 0-100%), 4-[2’-(oxan-2’’-yl)-*2H*-pyrazolo[3,4-c]pyridin-5’-yl]morpholine **13** was isolated as a dark green oil (0.051 g, 0.18 mmol, 62%).

δ_H (600 MHz, Chloroform-*d*) 9.05 (1H, t, *J* = 1.2 Hz, 7’-*H*), 7.99 (1H, d, *J* = 1.2 Hz, 3’-*H*), 6.58 (1H, d, *J* = 1.2 Hz, 4’-*H*), 5.66 (1H, dd, *J* = 8.2, 4.1 Hz, 2’’-*H*), 4.13 – 4.07 (1H, m, 6’’-*H*), 3.91 – 3.87 (4H, m, 2,6-*H_2*), 3.77 (1H, ddd, *J* = 11.7, 10.4, 3.0 Hz, 6’’-*H*), 3.40 – 3.35 (4H, m, 3,5-*H_2*), 2.20 (2H, ddd, *J* = 10.0, 8.2, 4.1 Hz, 3’’-H), 2.07 – 2.01 (1H, m, 4’’-*H*), 1.79 – 1.63 (1H, m, 4’’-*H*), 1.79 – 1.63 (2H, m, 5’’-*H*); δ_C (151 MHz, Chloroform-*d*) 154.1 (*C*-5’), 143.3 (*C*-7’), 142.7 (*C*-7’a), 126.1 (*C*-3’a), 119.3 (*C*-3’), 91.8 (*C*-4), 89.2 (*C*- 2’’), 67.8 (*C*-6’’), 66.9 (*C*-2,6), 48.1 (*C*-3,5), 31.2 (*C*-3’’), 24.9 (*C*-5’’), 21.9 (*C*-4’’); V_{max} (ATR) 1495, 1200, 0998, 729 cm^{-1}; LC-MS (ESI) [M + H] 289.294; HRMS (ESI) found $[M+H]^+$ 289.1670, $C_{15}H_{21}N_4O_2$ requires *M* 298.1665.

Compound ***14***

N-butyl-2-(oxan-2'-yl)-*2H*-pyrazolo[3,4-c]pyridin-5-amine

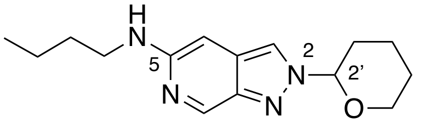

General procedure A was applied to 5-bromo-2-(oxan-2'-yl)-*2H*-pyrazolo[3,4-c]pyridine **6b** (0.080 g, 0.28 mmol, 1.00 eq) with *N*-butylamine (0.03 mL, 0.31 mmol, 1.10 eq). After purification by silica gel flash column chromatography (EtOAc:Pet Ether 40-60 0-80%), *N*-butyl-2-(oxan-2'-yl)-*2H*-pyrazolo[3,4-c]pyridine-5-amine **14** was isolated as a brown solid (0.049 g, 0.18 mmol, 63%) with mp 105-109 ˚C.

δ_H (600 MHz, Chloroform-*d*) 8.91 (1H, dd, *J* = 1.4, 0.9 Hz, 7-*H*), 7.89 (1H, d, *J* = 0.9 Hz, 3-*H*), 6.25 (1H, d, *J* = 1.4 Hz, 4-*H*), 5.65 – 5.62 (1H, m, 2'-*H*), 4.13 – 4.06 (1H, m, 6'-*H*), 3.76 (1H, ddd, *J* = 11.6, 10.3, 3.0 Hz, 6'-*H*), 3.13 (2H, t, *J* = 7.1 Hz, NHCH_2CH$_2$), 2.24 – 2.17 (2H, m, 3'-*H*), 2.08 – 2.02 (1H, m, 4'-*H*), 1.78 – 1.68 (1H, m, 4'-*H*), 1.78 – 1.68 (1H, m, 5'-*H*), 1.68 – 1.65 (2H, m, NHCH$_2$CH_2CH$_2$), 1.68 – 1.65 (1H, m, 5'-*H*), 1.53 – 1.42 (2H, m, CH$_2$CH_2CH$_3$), 0.96 (3H, t, *J* = 7.4 Hz, CH$_2$CH$_2$CH_3); δ_C (151 MHz, Chloroform-*d*) 152.5 (*C*-5), 143.7 (*C*-7), 142.4 (*C*-7a), 126.7 (*C*-3a), 118.2 (*C*-3), 89.1 (*C*-2'), 86.3 (*C*-4), 67.8 (*C*-6'), 43.4 (NH*C*H$_2$CH$_2$), 31.3 (NHCH$_2$*C*H$_2$), 31.1 (*C*-3'), 24.9 (*C*-5'), 21.9 (*C*-4'), 20.4 (CH$_2$*C*H$_2$CH$_3$), 13.9 (CH$_2$CH$_2$*C*H$_3$); V_{max} (ATR) 1635, 1507, 1089, 1048 cm^{-1}; LC-MS (ESI) [M + H] 275.309; HRMS (ESI) found [M+H]$^+$ 275.1874, $C_{15}H_{22}N_4O$ requires *M* 275.1872.

Compound ***15***

1-methyl-4-[2'-(oxan-2''-yl)-*2H*-pyrazolo[3,4-c]pyridin-5'-yl]piperazine

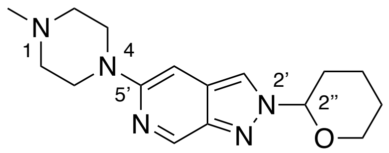

General procedure A was applied to 5-bromo-2-(oxan-2'-yl)-*2H*-pyrazolo[3,4-c]pyridine **6b** (0.080 g, 0.28 mmol, 1.00 eq) with *N*-methylpiperazine (0.04 mL, 0.31 mmol, 1.10 eq). After purification by silica gel flash column chromatography (EtOAc:Pet Ether 40-60 0-100%, MeOH:EtOAc 0-30%), 1-methyl-4-[2'-(oxan-2''-yl)-*2H*-pyrazolo[3,4-c]pyridin-5'-yl]piperazine **15** was isolated as a green oil (0.064 g, 0.21 mmol, 75%).

δ_H (600 MHz, Chloroform-*d*) 9.04 (1H, t, *J* = 1.3 Hz, 7'-*H*), 7.97 (1H, t, *J* = 1.3 Hz, 3'-*H*), 6.59 (1H, d, *J* = 1.3 Hz, 4'-*H*), 5.65 (1H, ddd, *J* = 8.0, 4.4, 2.3 Hz, 2''-*H*), 4.09 (1H, ddt, *J* = 11.8, 3.6, 2.3 Hz, 6''-*H*), 3.80 – 3.73 (1H, m, 6''-*H*), 3.47 – 3.41 (4H, m, 3,5-H_2), 2.63 (4H, td, *J* = 5.1, 2.3 Hz, 2,6-H_2), 2.37 (3H, d, *J* = 2.3 Hz, NCH_3), 2.19 (2H, dd, *J* = 4.4, 2.3 Hz, 3''-*H*), 2.03 (1H, dtq, *J* = 10.9, 4.4, 2.3 Hz, 5''-*H*), 1.80 – 1.68 (1H, m, 4''-*H*), 1.80 – 1.68 (1H, m, 5''-*H*), 1.65 (1H, dt, *J* = 12.2, 2.9 Hz, 4''-*H*); δ_C (151 MHz, Chloroform-*d*) 154.2 (*C*-5'), 143.3 (*C*-7'), 142.5 (*C*-7'a), 126.2 (*C*-3'a), 119.2 (*C*-3'), 91.9 (*C*-4'), 89.2 (*C*-2''), 67.8 (*C*-6''), 54.9 (*C*-2,6), 47.6 (*C*-3,5), 46.0 (N*C*H$_3$), 31.2 (*C*-3''), 24.9 (*C*-4''), 21.9 (*C*-5''); V_{max} (ATR) 1632, 1496, 1215, 1203 cm^{-1}; LC-MS (ESI) [M + H] 302.368; HRMS (ESI) found [M+H]$^+$ 302.1983, $C_{16}H_{24}N_5O$ requires *M* 302.1981.

Compound ***16***

2-(oxan-2'-yl)-*N*-phenyl-*2H*-pyrazolo[3,4-c]pyridin-5-amine

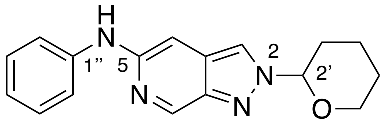

General procedure A was applied to 5-bromo-2-(oxan-2'-yl)-*2H*-pyrazolo[3,4-c]pyridine **6b** (0.080 g, 0.28 mmol, 1.00 eq) with aniline (0.03 mL, 0.31 mmol, 1.10 eq). After purification by silica gel flash column chromatography (EtOAc:Pet Ether 40-60 0-60%), 2-(oxan-2'-yl)-*N*-phenyl-*2H*-pyrazolo[3,4-c]pyridin-5-amine **16** was isolated as a green solid (0.063 g, 0.21 mmol, 75%) with mp 130-133 ˚C.

δ_H (600 MHz, Chloroform-*d*) 9.02 (1H, t, *J* = 1.2 Hz, 7-*H*), 7.97 (1H, d, *J* = 1.2 Hz, 3-*H*), 7.34 – 7.28 (2H, m, 3'',5''-*H*), 7.24 – 7.21 (2H, m, 2'',6''-*H*), 7.08 (1H, d, *J* = 1.2 Hz, 4-*H*), 7.01 – 6.95 (1H, m, 4''-*H*), 6.62 (1H, s, N-*H*), 5.67

(1H, dd, *J* = 8.7, 3.4 Hz, 2'-*H*), 4.10 (1H, dtd, *J* = 11.8, 3.7, 1.6 Hz, 6'-*H*), 3.77 (1H, ddd, *J* = 11.6, 10.2, 3.1 Hz, 6'-*H*), 2.24 – 2.14 (2H, m, 3'-*H*), 2.07 – 2.01 (1H, m, 5'-*H*), 1.79 – 1.64 (2H, m, 4'-*H*), 1.79 – 1.64 (1H, m, 5'-*H*); δ_C (151 MHz, Chloroform-*d*) 147.8 (*C*-5), 143.7 (*C*-7), 142.9 (*C*-7a), 142.0 (*C*-1''), 129.5 (*C*-3'',5''), 126.4 (*C*-3a), 121.9 (*C*-4''), 119.3 (*C*-3), 119.1 (*C*-2'',6''), 92.4 (*C*-4), 89.4 (*C*-2'), 68.0 (*C*-6'), 31.4 (*C*-3'), 25.0 (*C*-4'), 22.0 (*C*-5'); V_{max} (ATR) 1633, 1603, 1493, 1089, 1047cm^{-1}; LC-MS (ESI) [M + H] 295.299; HRMS (ESI) found $[M+H]^+$ 295.1578, $C_{17}H_{19}N_4O$ requires *M* 295.1559.

*Compound **17***
4-[1'-(oxan-2''-yl)-*1H*-pyrazolo[3,4-c]pyridin-5'-yl]morpholine

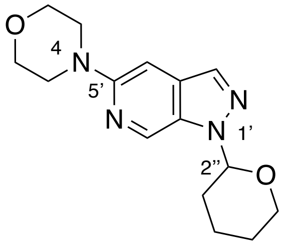

General procedure A was applied to 5-bromo-1-(oxan-2-yl)-1H-pyrazolo[3,4-c]pyridine **6a** (0.080 g, 0.28 mmol, 1.00 eq) with morpholine (0.03 mL, 0.31 mmol, 1.10 eq). After purification by silica gel flash column chromatography (EtOAc:Pet Ether 40-60 0-60%) followed by HPLC separation afforded 4-[1'-(oxan-2''-yl)-*1H*-pyrazolo[3,4-c]pyridin-5'-yl]morpholine **17** as a pink solid (0.036 g, 0.12 mmol, 43%) with mp 122-123 ˚C.

δ_H (400 MHz, Chloroform-*d*) 8.84 (1H, t, *J* = 1.0 Hz, 7'-*H*), 7.89 (1H, d, *J* = 1.0 Hz, 3'-*H*), 6.79 (1H, d, *J* = 1.0 Hz, 4'-*H*), 5.74 (1H, dd, *J* = 9.0, 2.5 Hz, 2''-*H*), 3.99 (1H, dtd, *J* = 11.6, 3.8, 1.4 Hz, 6''-*H*), 3.93 – 3.86 (4H, m, 2,6-*H₂*), 3.75 (1H, ddd, *J* = 11.6, 9.6, 3.5 Hz, 6''-*H*), 3.42 (4H, td, *J* = 4.4, 1.7 Hz, 3,5-*H₂*), 2.56 – 2.41 (1H, m, 3''-*H*), 2.16 – 2.11 (1H, m, 3''-*H*), 2.11 – 2.06 (1H, m, 4''-*H*), 1.85 – 1.62 (1H, m, 4''-*H*), 1.85 – 1.62 (2H, m, 5''-*H*); δ_C (101 MHz, Chloroform-*d*) 155.1 (*C*-5'), 132.8 (*C*-7'), 132.6 (*C*-7'a), 132.4 (*C*-3'), 132.1 (*C*-3'a), 95.1 (*C*-2''), 86.3 (*C*-4'), 67.4 (*C*-6''), 67.0 (*C*-2,6), 48.0 (*C*-3,5), 29.6 (*C*-3''), 25.2 (*C*-4''), 22.3 (*C*-5''); V_{max} (ATR) 1485, 1453, 1228, 1124, 1045, 970, 919 cm^{-1}; LC-MS (ESI) [M + H] 289.294; HRMS (ESI) found $[M+H]^+$ 289.1664, $C_{15}H_{21}N_4O_2$ requires *M* 289.1664.

*Compound **18***
4-(1'-{[2''-(trimethylsilyl)ethoxy]methyl}-*1H*-pyrazolo[3,4-c]pyridin-5'-yl)morpholine

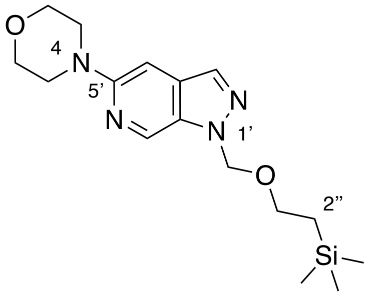

General procedure A was applied to 5-bromo-1-{[2'-(trimethylsilyl)ethoxy]methyl}-*1H*-pyrazolo[3,4-c]pyridine **8a** (0.300 g, 0.91 mmol, 1.00 eq) with morpholine (0.09 mL, 1.0 mmol, 1.10 eq). After purification by silica gel flash column chromatography (EtOAc:Pet Ether 40-60 0-30%), 4-(1'-{[2''-(trimethylsilyl)ethoxy]methyl}-*1H*-pyrazolo[3,4-c]pyridin-5'-yl)morpholine **18** was isolated as a pale green solid (0.297 g, 0.89 mmol, 97%) with mp 86-88 ˚C.

δ_H (600 MHz, Chloroform-*d*) 8.79 (1H, t, *J* = 1.1 Hz, 7'-*H*), 7.88 (1H, d, *J* = 1.1 Hz, 3'-*H*), 6.81 (1H, d, *J* = 1.1 Hz, 4'-*H*), 5.73 (2H, s, NCH_2O), 3.92 – 3.88 (4H, m, 2,6-H_2), 3.55 – 3.49 (2H, m, OCH_2CH_2), 3.46 – 3.42 (4H, m, 3,5-H_2), 0.90 – 0.84 (2H, m, CH2C*H2*Si), -0.07 (9H, s, $Si(CH_3)_3$); δ_C (151 MHz, Chloroform-*d*) 155.1 (*C*-5'), 132.7 (*C*-3'a), 132.5 (*C*-3'), 132.1 (*C*-7'a), 131.9 (*C*-7'), 94.9 (*C*-4'), 78.3 (NCH_2O), 66.9 (*C*-2,6), 66.6 (OCH_2CH_2), 47.8 (*C*-3,5), 17.74 (CH_2CH_2Si), -1.5 ($Si(CH_3)_3$); V_{max} (ATR) 1483, 1081, 907, 840, 735 cm^{-1}; LC-MS (ESI) [M + H] 335.393; HRMS (ESI) found $[M+H]^+$ 335.1931, $C_{16}H_{27}N_4O_2Si$ requires *M* 335.1903.

*Compound **19***
4-(2’-{[2’’-(trimethylsilyl)ethoxy]methyl}-*2H*-pyrazolo[3,4-c]pyridin-5’-yl)morpholin

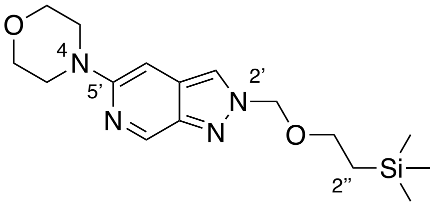

General procedure A was applied to 5-bromo-2-{[2’-(trimethylsilyl)ethoxy]methyl}-*2H*-pyrazolo[3,4-c]pyridine **8b** (0.200 g, 0.61 mmol, 1.00 eq) with morpholine (0.06 mL, 0.67 mmol, 1.10 eq). After purification by silica gel flash column chromatography (EtOAc:Pet Ether 40-60 0-60%), 4-(2’-{[2’’-(trimethylsilyl)ethoxy]methyl}-*2H*-pyrazolo[3,4-c]pyridin-5’-yl)morpholine **19** was isolated as a green oil (0.123 g, 0.37 mmol, 60%).

δ_H (600 MHz, Chloroform-*d*) 9.07 (1H, d, *J* = 1.1 Hz, 7’-*H*), 7.94 (1H, d, *J* = 1.1 Hz, 3’-*H*), 6.60 (1H, s, 4’-*H*), 5.70 (2H, s, NC*H*$_2$O), 3.92 – 3.88 (4H, m, 2,6-*H*$_2$), 3.64 – 3.58 (2H, m, OC*H*$_2$CH$_2$), 3.42 – 3.38 (4H, m, 3,5-*H*$_2$), 0.96 – 0.90 (2H, m, OCH$_2$C*H*$_2$Si), -0.04 (9H, s, Si(CH$_3$)$_3$); δ_C (151 MHz, Chloroform-*d*) 154.2 (*C*-5’), 143.4 (*C*-7’), 142.9 (*C*-7’a), 126.9 (*C*-3’a), 120.7 (*C*-3’), 91.6 (*C*-4’), 82.2 (N*C*H$_2$O), 67.9 (O*C*H$_2$CH$_2$), 66.9 (*C*-2,6), 48.0 (*C*-3,5), 17.8 CH$_2$*C*H$_2$Si), -1.5 (Si(*C*H$_3$)$_3$); V_{max} (ATR) 1498, 1119, 1104, 914, 735 cm^{-1}; LC-MS (ESI) [M + H] 335.355; HRMS (ESI) found [M+H]$^+$ 335.1911, $C_{16}H_{27}N_4O_2Si$ requires *M* 335.1903.

Borylation and Suzuki-Miyaura cross-coupling

General Procedure B
For tandem Borylation and Suzuki-Miyaura cross-coupling

[Ir(COD)OMe]$_2$ (0.025 eq), B$_2$pin$_2$ (1.10 eq), and dtbpy (0.05 eq) were sealed in an oven-dried microwave reaction vial and degassed with N$_2$/vacuum cycling. A solution of the SEM-protected pyrazolo[3,4-c]pyridine in anhydrous MTBE (0.4 M) was added under nitrogen. The reaction mixture was heated in a microwave reactor at 100 ˚C until GCMS analysis showed complete borylation had occurred, then concentrated under reduced pressure to afford the crude boronate ester.
To the crude boronate ester was added Cs_2CO_3 (2.00 eq), Pd(dppf)Cl$_2$ (0.025 eq), the aryl halide (1.10 eq) and anhydrous DMAc (1 M) under nitrogen. The reaction mixture was heated in a microwave reactor at 120 ˚C until GCMS analysis showed no boronate ester remained.
The reaction mixture was filtered through Celite and the residue washed with EtOAc. The combined filtrates were concentrated under reduced pressure, and the residue was dissolved in H_2O then extracted with EtOAc. The combined organic layers were washed with brine, dried over $MgSO_4$, filtered, and concentrated.
The product was purified by silica gel flash column chromatography using the stated solvent system.

General Procedure C
For tandem Borylation and Suzuki-Miyaura cross-coupling with CuCl

[Ir(COD)OMe]$_2$ (0.025 eq), B$_2$pin$_2$ (1.10 eq), and dtbpy (0.05 eq) were sealed in an oven-dried microwave reaction vial and degassed with N$_2$/vacuum cycling. A solution of the SEM-protected pyrazolo[3,4-c]pyridine in anhydrous MTBE (0.4 M) was added under nitrogen.
The reaction mixture was heated in a microwave reactor at 100 ˚C until GCMS analysis showed complete borylation had occurred, then concentrated under reduced pressure to afford the crude boronate ester.
To the crude boronate ester was added Cs_2CO_3 (1.00 eq), Pd(OAc)$_2$ (0.025 eq), 1,1’-Bis(diphenylphosphino) ferrocene (dppf) (0.050 eq), CuCl (1.00 eq), the aryl halide (1.10 eq) and anhydrous DMAc (1 M) under nitrogen. The reaction mixture was heated in a microwave reactor at 120 ˚C until GCMS analysis showed no boronate ester remained.
The reaction mixture was filtered through Celite and the residue washed with EtOAc. The combined filtrates were concentrated under reduced pressure, and the residue was dissolved in H_2O then extracted with EtOAc. The combined organic layers were washed with brine, dried over $MgSO_4$, filtered, and concentrated.
The product was purified by silica gel flash column chromatography using the stated solvent system.

*Compound **20***
methyl 4’-(5-chloro-1-{[2’’-(trimethylsilyl)ethoxy]methyl}-1H-pyrazolo[3,4-c]pyridin-3-yl)benzoate

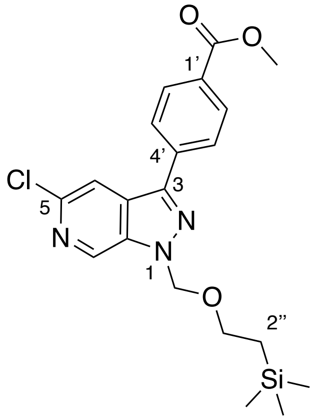

General procedure B was applied to 5-chloro-1-{[2’-(trimethylsilyl)ethoxy]methyl}-1H-pyrazolo[3,4-c]pyridine **9a** (0.050 g, 0.009 mmol, 1.00 eq) with methyl 4-iodobenzoate (0.069 g, 0.26 mmol, 1.50 eq). After purification by silica gel flash column chromatography (EtOAc:Hexanes 0-15%) methyl 4’-(5-chloro-1-{[2’’-(trimethylsilyl)ethoxy]methyl}-1H-pyrazolo[3,4-c]pyridin-3-yl)benzoate **20** was isolated as an off-white solid (0.035g, 0.083 mmol, 47%) with mp 98-103°C.

δ_H (600 MHz, Chloroform-*d*) 8.92 (1H, d, *J* = 1.1 Hz, 7-H), 8.20 – 8.15 (2H, m, 3’, 5’-H), 8.02 – 7.97 (2H, m, 2’, 6’-H), 7.93 (1H, d, *J* = 1.1 Hz, 4-*H*), 5.84 (2H, s, NC*H₂*O), 3.95 (3H, s, OC*H₃*), 3.65 – 3.58 (2H, m, OC*H₂*CH₂), 0.94 – 0.87 (2H, m, C*H₂*Si), -0.07 (9H, s, Si(C*H₃*)₃); δ_C (151 MHz, Chloroform-*d*) 166.6 (*C*=O), 142.7 (*C*-3), 142.5 (*C*-5), 137.1 (*C*-7a), 136.1 (*C*-1’), 134.1 (*C*-7), 130.3 (*C*-2’, 6’), 130.2 (*C*-4’), 129.3 (*C*-3a), 126.9 (*C*-3’, 5’), 114.6 (*C*-4), 78.9 (N*C*H₂O), 67.2 (O*C*H₂CH₂), 52.3 (O*C*H₃), 17.7 (*C*H₂Si), -1.5 (Si(*C*H₃)₃); V_{max} (ATR) 1714 (C=O), 1089, 833, 818, 701 cm^{-1}; LC-MS (ESI) [M (^{35}Cl) + H] 418.287, [M (^{37}Cl)+ H] 42.301; HRMS (ESI) found $[M+H]^+$ 418.1347, $C_{20}H_{25}N_3O_3Si^{35}Cl$ requires *M* 418.1332.

*Compound **21***
5-chloro-3-[4’-(trifluoromethyl)phenyl]-1-{[2”-(trimethylsilyl)ethoxy]methyl}-1H-pyrazolo[3,4-c]pyridine

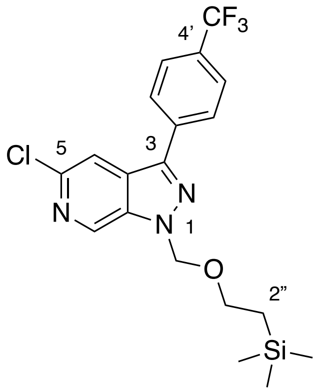

General procedure B was applied to 5-chloro-1-{[2’-(trimethylsilyl)ethoxy]methyl}-1H-pyrazolo[3,4-c]pyridine **9a** (0.150 g, 0.53 mmol, 1.00 eq) with 4-bromobenzotrifluoride (0.08 mL, 0.58 mmol, 1.10 eq). After purification by silica gel flash column chromatography (EtOAc:Hexanes 0-15%), 5-chloro-3-[4’-(trifluoromethyl)phenyl]-1-{[2”-(trimethylsilyl)ethoxy]methyl}-1H-pyrazolo[3,4-c]pyridine **21** was isolated as a white solid (0.127 g, 0.30 mmol, 56%) with mp 87-90 ˚C.

δ_H (700 MHz, Chloroform-*d*) 8.98 (1H, d, *J* = 1.2 Hz, 7-*H*), 8.08 (2H, d, *J* = 8.1 Hz, 2’, 6’-*H*), 7.96 (1H, d, *J* = 1.2 Hz, 4-*H*), 7.82 (2H, d, *J* = 8.1 Hz, 3’, 5’-*H*), 5.89 (2H, s, NC*H₂*O), 3.67 – 3.62 (2H, m, OC*H₂*CH₂), 0.98 – 0.93 (2H, m, C*H₂*SiMe₃), -0.03 (9H, s, Si(C*H₃*)₃); δ_C (176 MHz, Chloroform-*d*) 142.8 (*C*-3), 142.5 (*C*-5), 137.3 (*C*-7a), 135.5 (m, *C*-1’), 134.3 (*C*-7), 130.9 (q, J_{CF} = 32.5 Hz, *C*-4’), 129.4 (*C*-3a), 127.5 (*C*-2’, 6’), 126.2 (q, J_{CF} = 3.8 Hz, *C*-3’, 5’), 124.2 (q, J_{CF} = 272.8 Hz, *C*F₃), 114.6 (*C*-4), 79.1 (N*C*H₂O), 67.4 (O*C*H₂CH₂), 17.9 (*C*H₂SiMe₃), -1.3 (Si(*C*H₃)₃); δ_F (376 MHz,

Chloroform-*d*) -62.66; V_{max} (ATR) 1324, 1164, 1066, 860, 825 cm^{-1}; LC-MS (ESI) [M (^{35}Cl) + H] 428.282 [M (^{37}Cl)+ H] 430.296; HRMS (ESI) found [M+H]$^+$ 428.1165, *M* $C_{19}H_{22}N_3OSi^{35}ClF_3$ requires 428.1173.

Compound ***22***

5-chloro-3-(4'-nitrophenyl)-1-{[2''-(trimethylsilyl)ethoxy]methyl}-1H-pyrazolo[3,4-c]pyridine

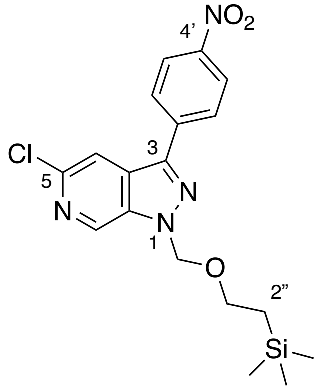

General procedure B was applied to 5-chloro-1-{[2'-(trimethylsilyl)ethoxy]methyl}-*1H*-pyrazolo[3,4-c]pyridine **9a** (0.150g, 0.53 mmol, 1.00 eq) with 1-iodo-4-nitrobenzene (0.145 g, 0.58 mmol, 1.10 eq). After purification by silica gel flash column chromatography (EtOAc:Hexanes 0-20%), 5-chloro-3-(4'-nitrophenyl)-1-{[2''-(trimethylsilyl)ethoxy]methyl}-*1H*-pyrazolo[3,4-c]pyridine **22** was isolated as a white solid (0.129 g, 0.32 mmol, 60 %) with mp 111-113 ˚C.

δ_H (600 MHz, Chloroform-*d*) 8.96 (1H, d, J = 1.2 Hz, 7-*H*), 8.41 – 8.35 (2H, m, 3',5'-*H*), 8.15 – 8.09 (2H, m, 2',6'-*H*), 7.94 (1H, d, J = 1.2 Hz, 4-*H*), 5.86 (2H, s, NC*H*$_2$O), 3.64 – 3.59 (2H, m, OC*H*$_2$CH$_2$), 0.95 – 0.88 (2H, m, CH$_2$C*H*$_2$SiMe$_3$), -0.06 (9H, s, SiC*H*$_3$); δ_C (151 MHz, Chloroform-*d*) 147.7 (*C*-4'), 143.0 (*C*-5), 141.4 (*C*-3), 138.1 (*C*-1'), 137.1 (*C*-7a), 134.4 (*C*-7), 129.2 (*C*-3a), 127.6 (*C*-3', 5'), 124.4 (*C*-2', 6'), 114.3 (*C*-4), 79.1 (N*C*H$_2$O), 67.4 (O*C*H$_2$CH$_2$), 17.7 (CH$_2$*C*H$_2$Si), -1.5 (Si(*C*H$_3$)$_3$); V_{max} (ATR) 1516 (N=O asymmetric), 1349 (N=O symmetric), 1074, 857, 834, 821 cm^{-1}; LC-MS (ESI) [M (^{35}Cl) + H] 405.282, [M (^{37}Cl) + H] 407.297; HRMS (ESI) found [M+H]$^+$ 405.1153, $C_{18}H_{22}N_4O_3Si^{35}Cl$ requires *M* 405.1150.

Compound ***23***

5-chloro-3-(3',5'-dimethylphenyl)-1-{[2''-(trimethylsilyl)ethoxy]methyl}-*1H*-pyrazolo[3,4-c]pyridine

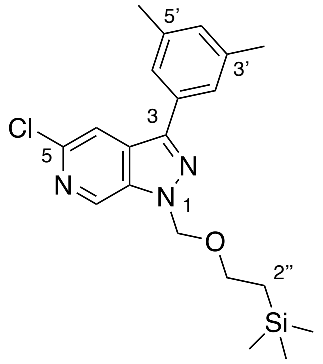

General procedure B was applied to 5-chloro-1-{[2'-(trimethylsilyl)ethoxy]methyl}-*1H*-pyrazolo[3,4-c]pyridine **9a** (0.200 g, 0.71 mmol, 1.00 eq) with 1-iodo-3,5-dimethylbenzene (0.11 mL, 0.78 mmol, 1.10 eq). After purification by silica gel flash column chromatography (EtOAc:Hexanes 0-20%), 5-chloro-3-(3',5'-dimethylphenyl)-1-{[2''-(trimethylsilyl)ethoxy]methyl}-*1H*-pyrazolo[3,4-c]pyridine **23** was isolated as a colourless oil (0.127 g, 0.33 mmol, 47%).

δ_H (600 MHz, Chloroform-*d*) 8.90 (1H, d, *J* = 1.1 Hz, 7-*H*), 7.92 (1H, d, *J* = 1.1 Hz, 4-*H*), 7.52 – 7.49 (2H, m, 2',6'-*H*), 7.12 – 7.09 (1H, m, 4'-*H*), 5.83 (2H, s, NC*H*$_2$O), 3.64 – 3.58 (2H, m, OC*H*$_2$CH$_2$), 2.43 (6H, q, *J* = 0.7 Hz, Ar-C*H*$_3$), 0.94 – 0.89 (2H, m, CH$_2$C*H*$_2$Si), -0.06 (9H, s, Si(C*H*$_3$)$_3$); δ_C (151 MHz, Chloroform-*d*) 144.3 (*C*-3), 141.9 (*C*-5), 138.8 (*C*-3',5'), 137.0 (*C*-7a), 133.7 (*C*-7), 131.5 (*C*-1'), 130.7 (*C*-4'), 129.5 (*C*-3a), 125.0 (*C*-2',6'), 115.0 (*C*-4), 78.7 (N*C*H$_2$O), 67.0 (O*C*H$_2$CH$_2$), 21.4 (Ar-*C*H$_3$), 17.7 (CH$_2$*C*H$_2$Si), -1.47 (Si(*C*H$_3$)$_3$); V_{max} (ATR) 1089, 863, 839 cm^{-1}; LC-MS (ESI) [M

(^{35}Cl) + H] 388.371, [M (^{37}Cl) + H] 390.385; HRMS (ESI) found $[M+H]^+$ 388.1616, $C_{20}H_{27}{}^{35}ClN_3OSi$ requires *M* 388.1612.

*Compound **24***
methyl 4’-(5-chloro-2-{[2’’-(trimethylsilyl)ethoxy]methyl}-2H-pyrazolo[3,4-c]pyridin-3-yl)benzoate

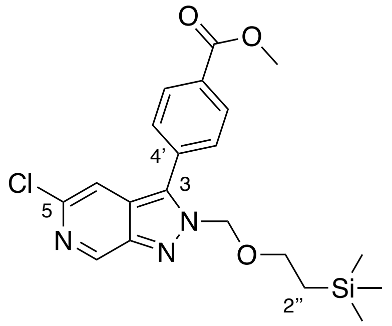

General procedure C was applied to 5-chloro-2-{[2’-(trimethylsilyl)ethoxy]methyl}-2H-pyrazolo[3,4-c]pyridine **9b** (0.150 g, 0.53 mmol, 1.00 eq) with methyl 4-iodobenzoate (0.152 g, 0.58 mmol, 1.10 eq). Purification by C_{18}-silica gel reverse phase flash column chromatography (MeOH:H_2O 0-100%) afforded methyl 4’-(5-chloro-2-{[2’’-(trimethylsilyl)ethoxy]methyl}-*2H*-pyrazolo[3,4-c]pyridine-3-yl)benzoate **24** as a yellow solid (0.088 g, 0.21 mmol, 40%) with mp 99 – 103 ˚C.

δ_H (600 MHz, Chloroform-*d*) 9.11 (1H, d, *J* = 1.3 Hz, 7-*H*), 8.23 (2H, d, *J* = 8.1 Hz, 2’,6’-*H*), 7.81 (2H, d, *J* = 8.2 Hz, 3’,5’-*H*), 7.61 – 7.56 (1H, d, *J* = 1.3 Hz, 4-*H*), 5.73 (2H, s, NC*H*$_2$O), 3.97 (3H, s, OC*H*$_3$), 3.90 – 3.83 (2H, m, OC*H*$_2$CH$_2$), 0.99 – 0.94 (2H, m, OCH$_2$C*H*$_2$), -0.01 (9H, s, Si(C*H*$_3$)$_3$); δ_C (151 MHz, Chloroform-*d*) 166.3 (*C*(=O)OCH$_3$), 144.9 (*C*-7), 143.8 (*C*-7a), 141.5 (*C*-5), 135.5 (*C*-3), 132.3 (*C*-4’), 130.8 (*C*-1’), 130.5 (*C*-2’,6’), 129.4 (*C*-3’,5’), 125.6 (*C*-3a), 112.9 (*C*-4), 80.1 (N*C*H$_2$O), 68.3 (O*C*H$_2$CH$_2$), 52.4 (O*C*H$_3$), 17.9 (OCH$_2$*C*H$_2$Si), -1.4 (Si(*C*H$_3$)$_3$); V_{max} (ATR) 1727 (C=O), 1279, 837, 741 cm^{-1}; LC-MS (ESI) [M (^{35}Cl) + H] 418.318, [M (^{37}Cl) + H] 420.332; HRMS found $[M+H]^+$ 418.1361, $C_{20}H_{25}{}^{35}ClN_3O_3Si$ requires *M* 418.1354.

*Compound **25***
5-chloro-3-[4’-(trifluoromethyl)phenyl]-2-{[2’’-(trimethylsilyl)ethoxy]methyl}-2H-pyrazolo[3,4-c]pyridine

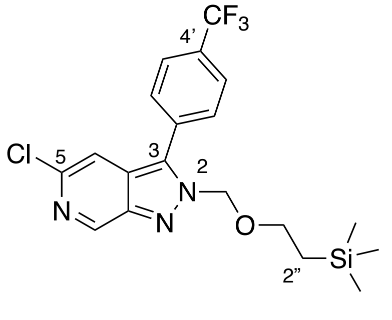

General procedure C was applied to 5-chloro-2-{[2’-(trimethylsilyl)ethoxy]methyl}-*2H*-pyrazolo[3,4-c]pyridine **9b** (0.100 g, 0.35 mmol, 1.00 eq) with 1-bromo-4-(trifluoromethyl)benzene (0.087 g, 0.39 mmol, 1.10 eq). After purification by silica gel flash column chromatography (EtOAc:Hexanes 0-20%), 5-chloro-3-[4’-(trifluoromethyl)phenyl]-2-{[2’’-(trimethylsilyl)ethoxy]methyl}-*2H*-pyrazolo[3,4-c]pyridine **25** was isolated as a yellow solid (0.072 g, 0.17 mmol, 48 %) with mp 104-105 ˚C.

δ_H (600 MHz, Chloroform-*d*) 9.15 (1H, d, *J* = 1.3 Hz, 7-*H*), 7.89 (2H, d, *J* = 8.3 Hz, 2’,6’-*H*), 7.85 (2H, d, *J* = 8.3 Hz, 3’,5’-*H*), 7.59 (1H, d, *J* = 1.3 Hz, 4-*H*), 5.74 (2H, s, NC*H*$_2$O), 3.89 – 3.82 (2H, m, OC*H*$_2$CH$_2$), 1.00 – 0.93 (2H, m, C*H*$_2$SiMe$_3$), -0.01 (9H, s, Si(C*H*$_3$)$_3$); δ_C (151 MHz, Chloroform-*d*) 145.0 (*C*-7), 143.8 (*C*-5), 141.7 (*C*-7a), 134.9 (*C*-3), 131.6 (d, J_{CF} = 1.4 Hz, *C*-1’), 131.4 (q, J_{CF} = 33.0 Hz, *C*-4’), 129.9 (*C*-2’,6’), 126.3 (q, J_{CF} = 3.7 Hz, *C*-3’,5’), 125.6 (*C*-3a), 123.7 (q, J_{CF} = 272.4 Hz, *C*F$_3$), 112.7 (*C*-4), 80.1 (N*C*H$_2$O), 68.4 (O*C*H$_2$CH$_2$), 17.9 (*C*H$_2$SiMe$_3$), -1.5 (Si(*C*H$_3$)$_3$); δ_F (376 MHz, Chloroform-*d*) -62.84; V_{max} (ATR) 1323, 911, 732 cm^{-1}; LC-MS (ESI) [M (^{35}Cl) + H] 428.236, [M (^{37}Cl)+ H] 430.251; HRMS (ESI) found $[M+H]^+$ 428.1177, $C_{19}H_{22}N_3OSiF_3{}^{35}Cl$ requires *M* 428.1173.

*Compound **26***

5-chloro-3-(4'-nitrophenyl)-2-{[2''-(trimethylsilyl)ethoxy]methyl}-*2H*-pyrazolo[3,4-c]pyridine

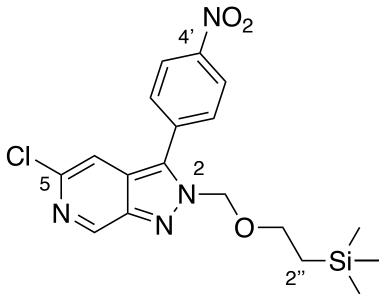

General procedure C was applied to 5-chloro-2-{[2'-(trimethylsilyl)ethoxy]methyl}-*2H*-pyrazolo[3,4-c]pyridine **9b** (0.150g, 0.53 mmol, 1.00 eq) with 1-iodo-4-nitrobenzene (0.145 g, 0.58 mmol, 1.10 eq). Purification by C_{18}-silica gel reverse phase flash column chromatography (MeOH:H_2O 0-100%) afforded 5-chloro-3-(4'-nitrophenyl)-2-{[2''-(trimethylsilyl)ethoxy]methyl}-*2H*-pyrazolo[3,4-c]pyridine **26** as a yellow oil (0.066 g, 0.16 mmol, 31%).

δ_H NMR (600 MHz, Chloroform-*d*) 9.16 (1H, d, *J* = 1.3 Hz, 7-*H*), 8.46 – 8.43 (2H, m, 3',5'-*H*), 7.99 – 7.96 (2H, m, 2',6'-*H*), 7.60 (1H, d, *J* = 1.3 Hz, 4-*H*), 5.76 (2H, s, NC*H*$_2$O), 3.92 – 3.87 (2H, m, OC*H*$_2$CH$_2$), 1.02 – 0.97 (2H, m, C*H*$_2$SiMe$_3$), 0.01 (9H, s, Si(C*H*$_3$)$_3$); δ_C NMR (151 MHz, Chloroform-*d*) 148.0 (*C*-4'), 145.2 (*C*-7), 143.8 (*C*-7a), 142.3 (*C*-5), 134.3 (*C*-1'), 134.0 (*C*-3), 130.3 (*C*-2',6'), 125.8 (*C*-3a), 124.6 (*C*-3',5'), 112.5 (*C*-4), 80.4 (N*C*H$_2$O), 68.6 (O*C*H$_2$CH$_2$), 18.0 (OCH$_2$*C*H$_2$Si), -1.4 (Si(*C*H$_3$)$_3$); V_{max} (ATR) 1523 (N-O, asymmetric), 1349 (N-O, symmetric), 858, 838, 734, 698 cm^{-1}; [M(^{35}Cl)+H] 405.320, [M(^{37}Cl)+H] 407.297; HRMS found [M+H]$^+$ 405.1146, $C_{18}H_{22}{}^{35}ClN_4O_3Si$ requires *M* 405.1150.

*Compound **27***

5-chloro-3-(3',5'-dimethylphenyl)-2-{[2''-(trimethylsilyl)ethoxy]methyl}-*2H*-pyrazolo[3,4-c]pyridine

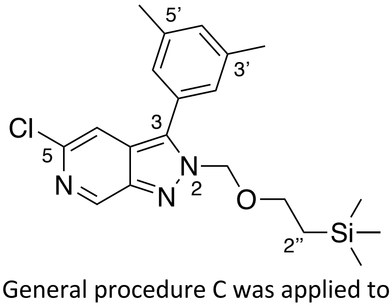

General procedure C was applied to 5-chloro-2-{[2'-(trimethylsilyl)ethoxy]methyl}-*2H*-pyrazolo[3,4-c]pyridine **9b** (0.100g, 0.35 mmol, 1.00 eq) with 1-iodo-3,5-dimethylbenzene (0.06 mL, 0.39 mmol, 1.10 eq). Purification by silica gel flash column chromatography (EtOAc:Pet Ether 40-60 0-20%) afforded 5-chloro-3-(3',5'-dimethylphenyl)-2-{[2''-(trimethylsilyl)ethoxy]methyl}-*2H*-pyrazolo[3,4-c]pyridine **27** as a yellow oil (0.065 g, 0.17 mmol, 48%).

δ_H (600 MHz, Chloroform-*d*) 9.11 (1H, d, *J* = 1.2 Hz, 7-*H*), 7.57 (1H, d, *J* = 1.2 Hz, 4-*H*), 7.30 – 7.27 (2H, m, 2',6'-*H*), 7.17 – 7.14 (1H, m, 4'-*H*), 5.72 (2H, s, NC*H*$_2$O), 3.86 – 3.80 (2H, m, OC*H*$_2$CH$_2$), 2.43 (6H, q, *J* = 0.7 Hz, Ar-C*H*$_3$), 0.98 – 0.93 (2H, m, CH$_2$C*H*$_2$Si), -0.01 (9H, s, Si(C*H*$_3$)$_3$); δ_C (151 MHz, Chloroform-*d*) 144.6 (*C*-7), 143.9 (*C*-5), 140.6 (*C*-7a), 139.0 (*C*-3',5'), 137.2 (*C*-3), 131.3 (*C*-4'), 127.8 (*C*-1'), 127.3 (*C*-2',6'), 125.4 (*C*-3a), 113.5 (*C*-4), 79.8 (N*C*H2O), 68.1 (O*C*H$_2$CH$_2$), 21.4 (Ar-*C*H$_3$), 17.9 (CH$_2$*C*H$_2$Si), -1.4 (Si(*C*H$_3$)$_3$); V_{max} (ATR) 1465, 1106, 1085, 1061, 858, 838 cm^{-1}; LC-MS (ESI) [M (^{35}Cl) + H] 388.333, [M (^{37}Cl) + H] 390.347; HRMS (ESI) found [M+H]$^+$ 388.1598, $C_{20}H_{27}{}^{35}ClN_3OSi$ requires *M* 388.1612.

Metal-base Chemistry

Preparation of TMPMgCl•LiCl

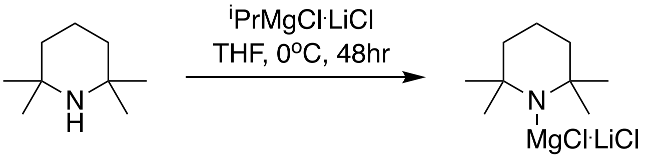

An oven dried RBF wrapped in foil to exclude light was charged with *turbo*-Grignard (isopropyl magnesium chloride lithium chloride complex 1.3 M in THF) under a nitrogen atmosphere in an ice + salt bath. Freshly distilled 2,2,6,6-tetramethylpiperidine (TMPH) (1.05 eq.) was added dropwise and the reaction stirred for 48 hours generating a dark grey solution. The concentration of the metal-base was calculated via titration against benzoic acid in THF conducted under a nitrogen atmosphere in an ice bath. The prepared TMPMgCl·LiCl solution was stored at -18 ˚C under a dry nitrogen atmosphere.

General Procedure D
For deproto-metalation by TMPMgCl·LiCl and trapping with an electrophile

An oven dried RBF was charged with a solution of the stated substrate (1.00 eq.) in dry THF (0.5 M) and cooled to -40 ˚C under a nitrogen atmosphere. TMPMgCl·LiCl in THF (2.00 eq.) was added dropwise and the reaction was stirred for 30 minutes at -40 ˚C. The corresponding electrophile was added at -40 ˚C, then the reaction was stirred at room temperature for the time stated. The reaction was quenched with $NaHSO_3$ sat. solution and the crude product extracted with EtOAc, then the combined organic layers were washed with brine, dried over $MgSO_4$, filtered, and concentrated under reduced pressure. The product was purified by silica gel flash column chromatography using the stated solvent system.

General Procedure E
For deproto-metalation by TMPMgCl·LiCl and transmetalation to Zn for Negishi cross-coupling

An oven-dried reaction vessel was charged with a solution of the substrate (1.00 eq.) in dry THF (0.5 M), the atmosphere was exchange to nitrogen, and the solution cooled to -40 ˚C. TMPMgCl·LiCl in THF (2.00 eq.) was added dropwise and the reaction was stirred for 30 minutes at -40 ˚C. A solution of $ZnCl_2$ (1.00 eq) in THF (1 M) was added and the reaction stirred for 30 minutes at -40 ˚C. In a separate oven-dried reaction vessel, a solution of the corresponding (hetero)aryl halide (1.50 eq) and $Pd(PPh_3)_4$ (0.05 eq) in THF (0.5 M) were stirred at room temperature for 30 minutes. This solution was added to the main reaction mixture at -40 ˚C, then the reaction was stirred at room temperature overnight. The reaction was quenched with NH_4Cl sat. solution and the crude product extracted with EtOAc, then the combined organic layers were washed with brine, dried over $MgSO_4$, filtered, and concentrated under reduced pressure. The product was purified by silica gel flash column chromatography using the stated solvent system.

*Compound **28***
5-chloro-7-iodo-1-{[2'-(trimethylsilyl)ethoxy]methyl}-*1H*-pyrazolo[3,4-c]pyridine

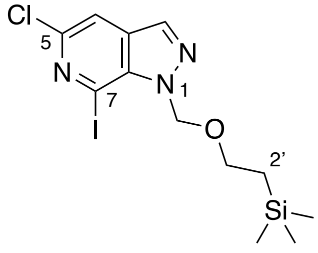

General procedure D was applied to 5-chloro-1-{[2'-(trimethylsilyl)ethoxy]methyl}-1H-pyrazolo[3,4-c]pyridine **9a** (0.100g, 0.35 mmol, 1.00 eq) with electrophile I_2 (0.134 g, 0.53 mmol, 1.50 eq) for 1 hour. Purification by reverse phase column chromatography (MeOH:H_2O 0-100%) afforded 5-chloro-7-iodo-1-{[2'-(trimethylsilyl)ethoxy]methyl}-*1H*-pyrazolo[3,4-c]pyridine **28** as a yellow oil (0.080g, 0.20 mmol, 55%).

δ_H (700 MHz, Chloroform-*d*) 8.06 (1H, s, 3-*H*), 7.66 (1H, s, 4-*H*), 6.14 (2H, s, NCH_2O), 3.66 – 3.61 (2H, m, OCH_2CH$_2$), 0.99 – 0.93 (2H, m, CH$_2$CH_2Si), 0.00 (9H, s, Si(CH_3)$_3$); δ_C (176 MHz, Chloroform-*d*) 140.1 (*C*-5), 138.3 (*C*-7a), 132.5 (*C*-3), 131.9 (*C*-3a), 114.1 (*C*-4), 96.3 (*C*-7), 77.7 (NCH$_2$O), 66.7 (OCH$_2$CH$_2$), 17.9 (CH$_2$CH$_2$Si), -1.3 (Si(*C*H$_3$)$_3$); V_{max} (ATR) 1082, 914, 798, 739 cm^{-1}; LC-MS (ESI) [M (^{35}Cl) + H] 410.147, [M (^{37}Cl) + H] 412.161; HRMS found [M+H]$^+$ 409.9954, $C_{12}H_{18}{}^{35}ClIN_3OSi$ requires *M* 409.9952.

*Compound **29***

5-chloro-7-(phenylsulfanyl)-1-{[2'-(trimethylsilyl)ethoxy]methyl}-*1H*-pyrazolo[3,4-c]pyridine

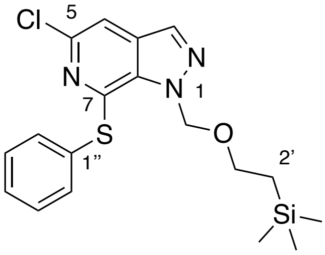

General procedure D was applied to 5-chloro-1-{[2'-(trimethylsilyl)ethoxy]methyl}-1H-pyrazolo[3,4-c]pyridine **9a** (0.150g, 0.53 mmol, 1.00 eq) with electrophile S_2Ph_2 (0.173 g, 0.79 mmol, 1.50 eq) for 18 hours. Purification by reverse phase column chromatography (MeCN:H_2O 0-100%) afforded 5-chloro-7-(phenylsulfanyl)-1-{[2'-(trimethylsilyl)ethoxy]methyl}-*1H*-pyrazolo[3,4-c]pyridine **29** as a yellow oil (0.104 g, 0.27 mmol, 50%).

δ_H (700 MHz, Chloroform-*d*) 7.98 (1H, s, 3-*H*), 7.58 (2H, dd, *J* = 7.5, 2.1 Hz, 2",6"-*H*), 7.43 – 7.40 (2H, m, 3",5"-*H*), 7.43 – 7.40 (1H, m, 4"-*H*), 7.39 (1H, s, 4-*H*), 6.09 (2H, s, NCH_2O), 3.64 – 3.59 (2H, m, OCH_2CH$_2$), 0.95 – 0.90 (2H, m, OCH$_2$CH_2Si), -0.04 (9H, s, Si(CH_3)$_3$); δ_C (176 MHz, Chloroform-*d*) 143.1 (*C*-7), 140.9 (*C*-5), 134.4 (*C*-2",6"), 134.3 (*C*-7a), 133.1 (*C*-3), 132.3 (*C*-3a), 129.4 (*C*-1"), 129.3 (*C*-3",5"), 129.1 (*C*-4"), 111.6 (*C*-4), 79.9 (N*C*H$_2$O), 66.7 (O*C*H$_2$CH$_2$Si), 17.9 (OCH$_2$*C*H$_2$Si), -1.3 (Si(*C*H$_3$)$_3$); V_{max} (ATR) 1078, 856, 833, 796, 689 cm^{-1}; LC-MS (ESI) [M (^{35}Cl) + H] 392.209, [M (^{37}Cl) + H] 394.223; HRMS found [M+H]$^+$ 392.1020, $C_{18}H_{23}{}^{35}ClN_3OSSi$ requires *M* 392.1020.

*Compound **30***

(5-chloro-1-{[2'-(trimethylsilyl)ethoxy]methyl}-*1H*-pyrazolo[3,4-c]pyridin-7-yl)(4''-chlorophenyl)methanol

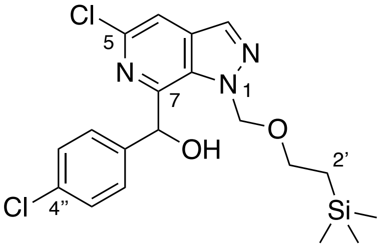

General procedure D was applied to 5-chloro-1-{[2'-(trimethylsilyl)ethoxy]methyl}-1H-pyrazolo[3,4-c]pyridine **9a** (0.100 g, 0.35 mmol, 1.00 eq) with electrophile 4-chlorobenzaldehyde (0.111 g, 0.79 mmol, 2.25 eq) for 4 hours. Purification by reverse phase column chromatography (MeCN:H_2O 0-100%) afforded (5-chloro-1-{[2'-(trimethylsilyl)ethoxy]methyl}-*1H*-pyrazolo[3,4-c]pyridin-7-yl)(4''-chlorophenyl)methanol **30** as a yellow solid (0.072, 0.17 mmol, 48%) with mp 82-84 ˚C.

δ_H (600 MHz, Chloroform-*d*) 8.00 (1H, s, 3-*H*), 7.66 (1H, s, 4-*H*), 7.28 (2H, d, *J* = 8.4 Hz, 3'',5''-*H*), 7.18 (2H, d, *J* = 8.4 Hz, 2'',6''-*H*), 6.43 (1H, s, C(OH)*H*), 5.47 – 5.38 (2H, m, NCH_2O), 3.49 – 3.36 (2H, m, OCH_2CH$_2$), 0.93 – 0.75 (2H, m, CH$_2$CH_2Si), -0.07 (9H, s, Si(CH_3)$_3$); δ_C (151 MHz, Chloroform-*d*) 145.7 (*C*-7), 140.9 (*C* -4''), 139.9 (*C*-5), 134.3 (*C*-1''), 133.7 (*C*-3a), 132.7 (*C*-3), 132.6 (*C*-7a), 129.2 (*C*-3',5'), 129.1 (*C*-2',6'), 114.3 (*C*-4), 79.8 (N*C*H$_2$O), 71.7 (*C*(OH)H), 66.5 (O*C*H$_2$CH$_2$), 17.6 (OCH$_2$*C*H$_2$Si), -1.6 (Si(*C*H$_3$)$_3$); V_{max} (ATR) 3410 (br, O-H), 2961, 1075, 833 cm^{-1}; LC-MS (ESI) [M (^{35}Cl) + H] 424.208, [M (^{37}Cl) + H] 426.222; HRMS found [M+H]$^+$ 424.1013, $C_{19}H_{24}{}^{35}Cl_2N_3O_3Si$ requires *M* 424.1015.

*Compound **31***
(5-chloro-1-{[2'-(trimethylsilyl)ethoxy]methyl}-*1H*-pyrazolo[3,4-c]pyridin-7-yl)(phenyl)methanol

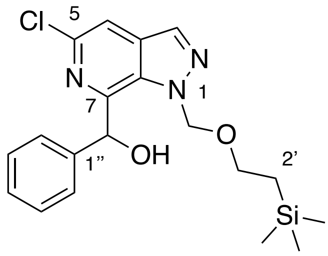

General procedure D was applied to 5-chloro-1-{[2'-(trimethylsilyl)ethoxy]methyl}-1H-pyrazolo[3,4-c]pyridine **9a** (0.100 g, 0.35 mmol, 1.00 eq) with electrophile benzaldehyde (0.05 mL, 0.53 mmol, 1.50 eq) for 4 hours. Purification by reverse phase column chromatography (MeCN:H_2O 0-100%) afforded (5-chloro-1-{[2'-(trimethylsilyl)ethoxy]methyl}-*1H*-pyrazolo[3,4-c]pyridin-7-yl)(phenyl)methanol **31** as a yellow solid (0.090 g, 0.23 mmol, 66%) with mp 63-68 ˚C.

δ_H (600 MHz, Chloroform-*d*) 8.00 (1H, s, 3-*H*), 7.66 (1H, s, 4-*H*), 7.34 – 7.30 (2H, m, 3",5"-*H*), 7.30 – 7.28 (1H, m, 4"-*H*), 7.26 – 7.23 (2H, m, 2",6"-*H*), 6.47 (1H, s, C(OH)*H*), 5.46 – 5.37 (2H, m, NCH_2O), 5.29 (1H, br s, O-*H*),3.51-3.38 (2H, m, OCH_2CH_2), 0.96 – 0.78 (2H, m, OCH_2CH_2Si), -0.05 (9H, s, Si(CH_3)$_3$); δ_C (151 MHz, Chloroform-*d*) 146.3 (*C*-7), 142.4 (*C*-1"), 139.8 (*C*-5), 133.6 (*C*-3a), 132.7 (*C*-7a), 132.7 (*C*-3), 129.1 (*C*-3",5"), 128.5 (*C*-4"), 127.7 (*C*-2",6"), 114.0 (*C*-4), 79.8 (NCH_2O), 72.4 (*C*(OH)H), 66.5 (OCH_2CH_2), 17.6 (OCH_2CH_2Si), -1.5 (Si(CH_3)$_3$); V_{max} (ATR) 3337 (br, O-H), 2958, 1085, 1070, 1063 cm^{-1}; LC-MS (ESI) [M (^{35}Cl) + H] 390.271, [M (^{37}Cl) + H] 392.285; HRMS found $[M+H]^+$ 390.1403, $C_{19}H_{25}{}^{35}ClN_3O_2Si$ requires *M* 390.1405.

*Compound **32***
5-chloro-1-{[2'-(trimethylsilyl)ethoxy]methyl}-*1H*-pyrazolo[3,4-c]pyridine-7-carbaldehyde

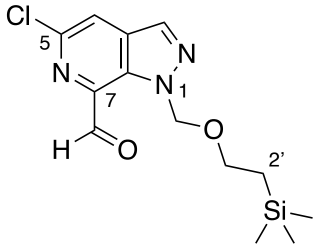

General procedure D was applied to 5-chloro-1-{[2'-(trimethylsilyl)ethoxy]methyl}-*1H*-pyrazolo[3,4-c]pyridine **9a** (0.200 g, 0.70 mmol, 1.00 eq) with neat DMF (0.08 mL, 1.05 mmol, 1.50 eq) for 5 hours. Purification by silica gel flash column chromatography (EtOAc:Hexanes 5-25%) afforded 5-chloro-1-{[2-(trimethylsilyl)ethoxy]methyl}-1H-pyrazolo[3,4-c]pyridine-7-carbaldehyde **32** as a cream solid (0.107 g, 0.34 mmol, 49%) with mp 60-62 ˚C.

δ_H (600 MHz, Chloroform-*d*) 10.16 (1H, s, C(=O)*H*), 8.18 (1H, s, 3-*H*), 7.94 (1H, s, 4-*H*), 6.25 (2H, s, NCH_2O), 3.47 – 3.41 (2H, m, OCH_2CH_2), 0.84 – 0.77 (2H, m, OCH_2CH_2Si), -0.11 (9H, s, Si(CH_3)$_3$); δ_C NMR (151 MHz, Chloroform-*d*) 191.2 (*C*=O), 140.5 (*C*-5), 138.0 (*C*-7), 135.7 (*C*-3a), 133.6 (*C*-3), 132.2 (*C*-7a), 120.0 (*C*-4), 81.7 (NCH_2O), 66.5 (OCH_2CH_2), 17.7 (OCH_2CH_2Si), -1.6 (Si(CH_3)$_3$); V_{max} (ATR) 1717 (C=O), 1085, 1056, 838, 796 cm^{-1}; LC-MS (ESI) [M (^{35}Cl) + H] 312.211, [M (^{37}Cl) + H] 314.187; HRMS found $[M+H]^+$ 312.0938, $C_{13}H_{19}{}^{35}ClN_3O_2Si$ requires *M* 312.0935.

*Compound **33***

5-chloro-7-(4'-methoxyphenyl)-1-{[2''-(trimethylsilyl)ethoxy]methyl}-*1H*-pyrazolo[3,4-c]pyridine

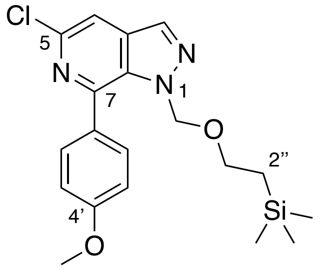

General procedure E was applied to 5-chloro-1-{[2'-(trimethylsilyl)ethoxy]methyl}-*1H*-pyrazolo[3,4-c]pyridine **9a** (0.100 g, 0.35 mmol, 1.00 eq) with 1-iodo-4-methoxybenzene (0.124 g, 0.53 mmol, 1.50 eq). Purification by reverse phase column chromatography (MeCN:H_2O 0-100%) afforded 5-chloro-7-(4'-methoxyphenyl)-1-{[2''-(trimethylsilyl)ethoxy]methyl}-*1H*-pyrazolo[3,4-c]pyridine **32** as a yellow oil (0.108 g, 0.28 mmol, 79%).

δ_H (600 MHz, Chloroform-*d*) 8.10 (1H, s, 3-*H*), 7.68 – 7.62 (2H, m, 2',6'- *H*), 7.61 (1H, s, 4- *H*), 7.05 – 7.00 (2H, m, 3',5'- *H*), 5.45 (2H, s, NCH_2O), 3.88 (3H, s, OCH_3), 3.39 – 3.33 (2H, m, OCH_2CH_2), 0.80 – 0.73 (2H, m, CH_2CH_2Si), -0.08 (9H, s, $Si(CH_3)_3$); δ_C (151 MHz, Chloroform-*d*) 160.7 (*C*-4'), 145.4 (*C*-7), 140.7 (*C*-5), 134.2 (*C*-7a), 133.4 (*C*-3), 133.2 (*C*-3a), 130.7 (*C*-2',6'), 129.5 (*C*-1'), 113.9 (*C*-3',5'), 112.7 (*C*-4), 78.2 NCH_2O), 66.5 (OCH_2CH_2), 55.4 ($O(CH_3)$, 17.7 NCH_2CH_2), -1.5 ($Si(CH_2)$; V_{max} (ATR) 1253 (Ar-O), 1078 (O-Me), 838, 800 cm^{-1}; LC-MS (ESI) [M (^{35}Cl) + H] 390.271, [M (^{37}Cl) + H] 392.285; HRMS found $[M+H]^+$ 390.1416, $C_{19}H_{25}{}^{35}ClN_3O_2Si$ requires M 390.1405.

*Compound **34***

4-(5'-chloro-1'-((2''-(trimethylsilyl)ethoxy)methyl)-*1H*-pyrazolo[3,4-c]pyridin-7'-yl)benzonitrile

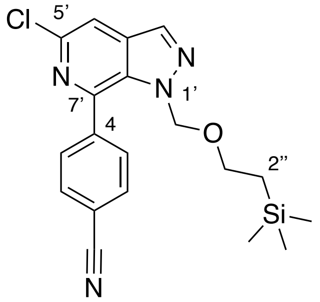

General procedure E was applied to 5-chloro-1-{[2'-(trimethylsilyl)ethoxy]methyl}-*1H*-pyrazolo[3,4-c]pyridine **9a** (0.100 g, 0.35 mmol, 1.00 eq) with 4-iodobenzonitrile (0.121 g, 0.55 mmol, 1.50 eq). Purification by reverse phase column chromatography (MeCN:H_2O 50-100%) afforded 4-(5'-chloro-1'-((2''-(trimethylsilyl)ethoxy)methyl)-*1H*-pyrazolo[3,4-c]pyridin-7'-yl)benzonitrile **34** as a cream solid (0.113 g, 0.29 mmol, 83%) with mp 90-94 ˚C.

δ_H (600 MHz, Chloroform-*d*) 8.13 (1H, s, 3'-*H*), 7.85 (2H, d, *J* = 7.9 Hz, 2,6-*H*), 7.81 (2H, d, *J* = 7.9 Hz, 3,5-*H*), 7.71 (1H, s, 4'-*H*), 5.38 (2H, s, NCH_2O), 3.40 (2H, m, OCH_2CH_2), 0.77 (2H, m, OCH_2CH_2Si), -0.07 (9H, s, $Si(CH_3)_3$); δ_C (151 MHz, Chloroform-*d*) 143.0 (*C*-7'), 141.4 (*C*-4), 140.9 (*C*-5'), 133.7 (*C*-3'a), 133.6 (*C*-7'a), 133.4 (*C*-3'), 132.1 (*C*-2,6), 130.2 (*C*-3,5), 118.3 (*C*≡N), 114.3 (*C*-4'), 113.4 (*C*-1), 78.4 (NCH_2O), 66.8 (OCH_2CH_2), 17.7 (OCH_2CH_2Si), -1.5 ($Si(CH_3)_3$); V_{max} (ATR) 2230 (C≡N), 1070, 856, 837, 815 cm^{-1}; LC-MS (ESI) [M (^{35}Cl) + H] 385.330, [M (^{37}Cl) + H] 387.307; HRMS found $[M+H]^+$ 385.1250, $C_{19}H_{22}{}^{35}ClN_4OSi$ requires *M* 385.1251.

*Compound **35***

5-chloro-7-(pyridin-2′-yl)-1-((2′′-(trimethylsilyl)ethoxy)methyl)-*1H*-pyrazolo[3,4-c]pyridine

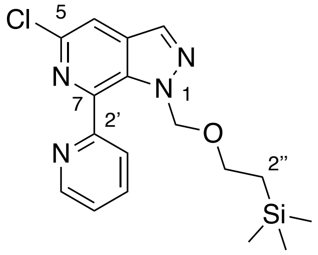

General procedure E was applied to 5-chloro-1-{[2′-(trimethylsilyl)ethoxy]methyl}-*1H*-pyrazolo[3,4-c]pyridine **9a** (0.100 g, 0.35 mmol, 1.00 eq) with 2-iodopyridine (0.06 mL, 0.55 mmol, 1.50 eq). Purification by reverse phase column chromatography (MeCN:H_2O 0-80%) afforded 5-chloro-7-(pyridin-2′-yl)-1-((2′′-(trimethylsilyl) ethoxy)methyl)-*1H*-pyrazolo[3,4-c]pyridine **35** as a yellow oil (0.091 g, 0.25 mmol, 71%).

δ_H (700 MHz, Chloroform-*d*) 8.74 (1H, ddd, *J* = 4.8, 1.8, 0.9 Hz, 6'-*H*), 8.11 (1H, s, 3-*H*), 8.08 (1H, dt, *J* = 7.8, 0.9 Hz, 3'-*H*), 7.91 (1H, td, *J* = 7.8, 1.8 Hz, 4'-*H*), 7.72 (1H, s, 4-*H*), 7.42 (1H, ddd, *J* = 7.8, 4.8, 0.9 Hz, 5'-*H*), 6.02 (2H, s, NC*H*$_2$O), 3.14 – 3.05 (2H, m, OC*H*$_2$CH$_2$Si), 0.59 – 0.49 (2H, m, OCH$_2$C*H*$_2$Si), -0.22 (9H, s, Si(C*H*$_3$)$_3$); δ_C (176 MHz, Chloroform-*d*) 156.0 (*C*-2'), 148.3 (*C*-6'), 143.7 (*C*-7), 140.3 (*C*-5), 137.4 (*C*-4'), 134.7 (*C*-3a), 133.5 (*C*-7a), 133.1 (*C*-3), 125.2 (*C*-3'), 124.1 (*C*-5'), 114.7 (*C*-4), 80.9 (N*C*H$_2$O), 66.0 (O*C*H$_2$CH$_2$Si), 17.6 (OCH$_2$*C*H$_2$Si), -1.5 (Si(*C*H$_3$)$_3$); V_{max} (ATR) 1078, 863, 837, 796 cm^{-1}; LC-MS (ESI) [M (^{35}Cl) + H] 361.274, [M (^{37}Cl) + H] 362.288; HRMS found $[M+H]^+$ 361.1253, $C_{17}H_{22}{}^{35}ClN_4OSi$ requires *M* 361.1251.

*Compound **36***

5-chloro-3-iodo-2-{[2′-(trimethylsilyl)ethoxy]methyl}-*2H*-pyrazolo[3,4-c]pyridine

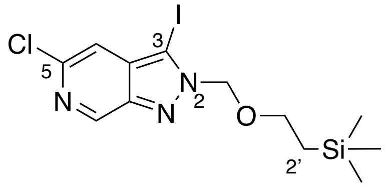

TMPMgCl·LiCl in THF (1.20 eq.) was added dropwise to a cooled solution of 5-chloro-2-{[2′-(trimethylsilyl)ethoxy]methyl}-*2H*-pyrazolo[3,4-c]pyridine **9b** (0.040g, 0.14 mmol, 1.00 eq) and the reaction was stirred for 1 hour at -78 ˚C under a nitrogen atmosphere. A solution of I_2 in THF (1.50 eq, 0.5 M) was added, then the reaction was stirred at room temperature for 1 hour. The reaction was quenched with NH_4Cl sat. solution (10 mL) and the crude product extracted with EtOAc (3 x 10 mL), then the combined organic layers were washed with brine (10 mL), dried over $MgSO_4$, filtered, and concentrated under reduced pressure. Purification by silica gel flash column chromatography (EtOAc:Pet Ether 40-60 0-20%) afforded 5-chloro-3-iodo-2-{[2′-(trimethylsilyl)ethoxy]methyl}-*2H*-pyrazolo[3,4-c]pyridine **36** as a white solid (0.0063 g, 0.015 mmol, 11%) with mp 101-103 ˚C.

δ_H (600 MHz, Chloroform-*d*) 8.85 (1H, d, *J* = 1.1 Hz, 7-*H*), 7.45 (1H, d, *J* = 1.1 Hz, 4-*H*), 5.78 (2H, s, NC*H*$_2$O), 3.59 – 3.53 (2H, m, OC*H*$_2$CH$_2$), 0.92 – 0.85 (2H, m, CH$_2$C*H*$_2$Si), -0.05 (9H, s, Si(C*H*$_3$)$_3$); δ_C (151 MHz, Chloroform-*d*) 142.3 (*C*-5), 136.4 (*C*-3), 136.2 (*C*-7a), 133.9 (*C*-7), 114.9 (*C*-4), 90.3 (*C*-3), 79.0 (N*C*H$_2$O), 67.3 (O*C*H$_2$CH$_2$), 17.7 (CH$_2$*C*H$_2$Si), -1.5 (Si(*C*H$_3$)$_3$); V_{max} (ATR) 1463, 1085, 816, 736 cm^{-1}; LC-MS (ESI)) [M (^{35}Cl) + H] 410.147, [M (^{37}Cl) + H] 412.161; HRMS found $[M+H]^+$ 409.9952, $C_{12}H_{18}{}^{35}ClIN_3OSi$ requires *M* 409.9952.

Multi-vector Elaboration

*Compound **37***
5-chloro-1-methyl-3-[4'-(trifluoromethyl)phenyl]-*1H*-pyrazolo[3,4-c]pyridine

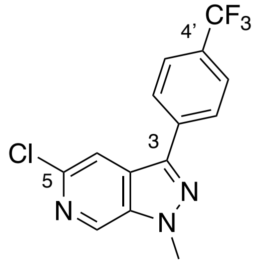

Route 1

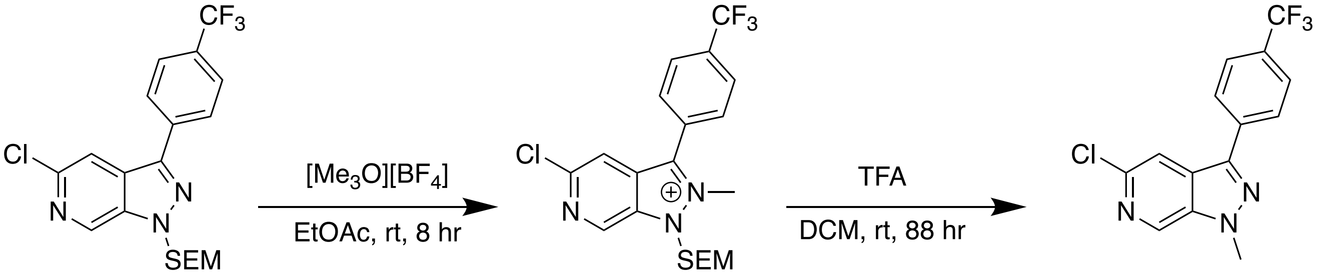

Trimethoxonium tetrafluoroborate (0.036 g, 0.23 mmol, 2.00 eq) and 5-chloro-3-[4'-(trifluoromethyl)phenyl]-1-{[2''-(trimethylsilyl)ethoxy]methyl}-*1H*-pyrazolo[3,4-c]pyridine **21** (0.050 g, 0.12 mmol, 1.0 eq) were sealed under a nitrogen atmosphere. Anhydrous EtOAc (1.5 mL) was added and the reaction stirred at room temperature for 8 hours. The reaction mixture was concentrated under reduced pressure to afford a white solid. The crude salt was taken up in anhydrous DCM (1.0 mL) then TFA (2.0 mL) was added dropwise under a nitrogen atmosphere, and the reaction stirred at room temperature for 3 days. The reaction was diluted with DCM (10 mL) then washed with sat. $NaHCO_3$ (3 x 15 mL) and brine (20 mL), then dried over $MgSO_4$, filtered, and concentrated. Purification by silica gel flash column chromatography (MeOH:DCM 0-30%) afforded 5-chloro-1-methyl-3-[4'-(trifluoromethyl)phenyl]-*1H*-pyrazolo[3,4-c]pyridine **37** as a red semi-solid (0.024 g, 0.075 mmol, 66%).

Route 2

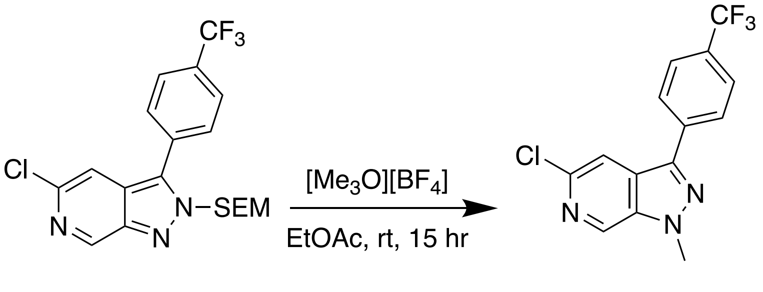

Trimethoxonium tetrafluoroborate (0.026 g, 0.18 mmol, 1.50 eq) and 5-chloro-3-[4'-(trifluoromethyl)phenyl]-2-{[2''-(trimethylsilyl)ethoxy]methyl}-*2H*-pyrazolo[3,4-c]pyridine **25** (0.050 g, 0.12 mmol, 1.00 eq) were sealed under a nitrogen atmosphere. Anhydrous EtOAc (3.0 mL) was added and the reaction stirred at room temperature overnight. The reaction mixture was concentrated under reduced pressure then washed with minimal H_2O. Purification by silica gel flash column chromatography (MeOH:DCM 0-30%) afforded 5-chloro-1-methyl-3-[4'-(trifluoromethyl)phenyl]-*1H*-pyrazolo[3,4-c]pyridine **37** as a red semi-solid (0.018 g, 0.059 mmol, 50%).

δ_H (600 MHz, DMSO) 9.85 (1H, s, 7-*H*), 8.77 (1H, s, 4-*H*), 8.34 (2H, d, *J* = 8.1 Hz, 2',6'-*H*), 7.83 (2H, d, *J* = 8.1 Hz, 3',5'-*H*), 4.35 (3H, s, NC*H*$_3$); δ_C (151 MHz, DMSO) 142.1 (*C*-3)*, 138.3 (*C*-5)*, 137.9 (*C*-7), 135.2 (*C*-1'), 132.24 (*C*-7a), 128.6 (q, $J_{C\text{-}F}$ = 31.8 Hz, *C*-4'), 127.3 (*C*-2',6'), 126.9 (*C*-3a)*, 126.0 (q, $J_{C\text{-}F}$ = 3.9 Hz, *C*-3',5'), 126.3 (CF_3, q, $J_{C\text{-}F}$ = 272.1 Hz), 118.8 (*C*-4), 47.3 (N*C*H$_3$); δ_F (376 MHz, DMSO) -62.91; V_{max} (ATR) 1329, 1071, 1018, 847 cm^{-1}; LC-MS (ESI): [M (^{35}Cl) + H] 312.217, [M (^{37}Cl)+ H] 314.269; HRMS (ESI) found $[M+H]^+$ 312.0504, $C_{14}H_{10}N_3{}^{35}ClF_3$ requires *M* 312.0515.
*resolved in ^{13}C NMR at 90 ˚C.

*Compound **40***
5-chloro-1-methyl-3-(4'-nitrophenyl)-*1H*-pyrazolo[3,4-c]pyridine

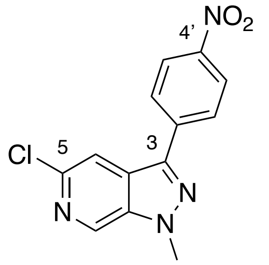

Trimethoxonium tetrafluoroborate (0.027 g, 0.19 mmol, 1.50 eq) and 5-chloro-3-[4'-nitrophenyl]-1-{[2''-(trimethylsilyl)ethoxy]methyl}-*1H*-pyrazolo[3,4-c]pyridine **22** (0.050 g, 0.12 mmol, 1.00 eq) were sealed under nitrogen. Anhydrous EtOAc (3.0 mL) was added and the reaction stirred at room temperature for 6 hours, a pale precipitate was generated. TFA (2.0 mL) was added dropwise and the reaction stirred at room temperature overnight. The reaction mixture was diluted with EtOAc (2.0 mL) then concentrated under reduced pressure. EtOAc (3 x 10 mL) was used as an azeotroping agent to remove the TFA by concentrating the product under reduced pressure 3 times to afford a white solid, the trifluoroacetate salt. Reaction with NH_3 in MeOH (1.0 mL) and washing with minimal H_2O afforded 5-chloro-1-methyl-3-(4'-nitrophenyl)-*1H*-pyrazolo[3,4-c]pyridine **40** as a bright yellow solid (0.034 g, 0.12 mmol, 95%) which is thermally unstable above 170 ˚C.

δ_H (400 MHz, DMSO) 9.73 (1H, s, 7-*H*), 8.65 (1H, s, 4-*H*), 8.42 (2H, d, *J* = 9.0 Hz, 3',5'-*H*), 8.29 (2H, d, *J* = 9.0 Hz, 2',6'-*H*), 4.30 (3H, s, NC*H*$_3$); δ_H (600 MHz, DMSO + TFA) 10.10 (1H, s, 7-*H*), 9.15 (1H, s, 4-*H*), 8.40 (2H, d, *J* = 8.9 Hz, 3',5'-*H*), 8.37 (2H, d, *J* = 8.9 Hz, 2',6'-*H*), 4.46 (3H, s, NC*H*$_3$); δ_C (400 MHz, DMSO + TFA) 147.6 (*C*-4'), 142.2 (*C*-3), 137.7 (*C*-7), 137.7 (*C*-5), 136.4 (*C*-1'), 134.2 (*C*-7a), 128.2 (*C*-3',5'), 127.2 (*C*-3a), 124,4 (*C*-2',6'), 119.7 (*C*-4), 47.9 (N*C*H$_3$); V_{max} (ATR) 1592 (N=O, asymmetric), 1503, 1329 (N=O, symmetric), 856 cm^{-1}; LC-MS (ESI): [M (^{35}Cl) + H] 289.104, [M (^{37}Cl)+ H] 291.157; HRMS (ESI) found $[M+H]^+$ 289.0509, $C_{13}H_{10}N_4O_2{}^{35}Cl$ requires *M* 289.0492.

*Compound **41***
5-chloro-7-iodo-3-(4'-(trifluoromethyl)phenyl)-1-((2''-(trimethylsilyl)ethoxy)methyl)-*1H*-pyrazolo[3,4-c]pyridine

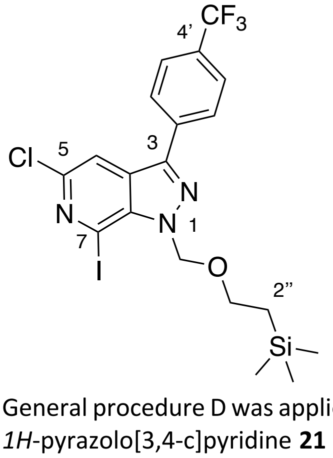

General procedure D was applied to 5-chloro-3-[4-(trifluoromethyl)phenyl]-1-{[2-(trimethylsilyl)ethoxy]methyl}-*1H*-pyrazolo[3,4-c]pyridine **21** (0.140 g, 0.33 mmol, 1.00 eq) with electrophile I_2 (0.125 g, 0.49 mmol, 1.50 eq) for 1 hour. Purification by reverse phase column chromatography (MeOH:H_2O 50-100%) afforded 5-chloro-7-

iodo-3-(4-(trifluoromethyl)phenyl)-1-((2-(trimethylsilyl)ethoxy)methyl)-*1H*-pyrazolo[4,3-d]pyridine **41** as a yellow oil (0.119 g, 0.22 mmol, 66%).

δ_H (700 MHz, Chloroform-*d*) 7.98 (2H, d, *J* = 8.1 Hz, 2',6'-*H*), 7.83 (1H, s, 4-*H*), 7.79 (2H, d, *J* = 8.1 Hz, 3',5'-*H*), 6.16 (2H, s, NCH_2O), 3.70 – 3.65 (2H, m, OCH_2CH_2), 0.94 (2H, m, OCH_2CH_2Si), -0.04 (9H, s, $Si(CH_3)_3$); δ_C (176 MHz, Chloroform-*d*) 142.2 (*C*-3), 141.0 (*C*-5), 139.6 (*C*-7a), 134.8 (q, J_{C-F} = 1.4 Hz, *C*-1'), 131.2 (q, J_{C-F} = 32.7 Hz, *C*-4'), 129.5 (*C*-3a), 127.8 (*C*-2',6'), 126.3 (q, *J* = 3.8 Hz, *C*-3',5'), 124.1 (q, *J* = 272.2 Hz, CF_3), 114.0 (*C*-4), 97.1 (*C*-7), 77.9 (NCH_2O), 66.8 (OCH_2CH_2), 17.9 (CH_2CH_2Si), -1.3 ($Si(CH_3)_3$); δ_F (376 MHz, Chloroform-*d*) -62.72; V_{max} (ATR) 1327, 1078, 840, 826 cm^{-1}; LC-MS (ESI) [M (^{35}Cl) + H] 554.14, [M (^{37}Cl) + H] 556.16, [M (^{35}Cl) + H – I] 428.27, [M (^{37}Cl) + H – I] 430.21; HRMS found $[M+H]^+$ 554.0149, $C_{19}H_{21}{}^{35}ClF_3IN_3OSi$ requires *M* 554.0139.

*Compound **42***
4-{1'-methyl-*1H*-pyrazolo[3,4-c]pyridin-5'-yl}morpholine

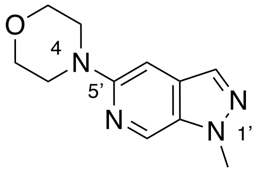

General procedure A was applied to 5-bromo-1-methyl-*1H*-pyrazolo[3,4-c]pyridine **10a** (0.150 g, 0.71 mmol, 1.00 eq) with morpholine (0.07 mL, 0.78 mmol, 1.10 eq). After purification by silica gel flash column chromatography (EtOAc:Pet Ether 40-60 0-70%), 4-{1'-methyl-*1H*-pyrazolo[3,4-c]pyridin-5'-yl}morpholine **42** was isolated as a yellow solid (0.297 g, 0.89 mmol, 97%).

δ_H (600 MHz, Chloroform-*d*) 8.63 (1H, t, *J* = 1.0 Hz, 7'-*H*), 7.83 (1H, d, *J* = 1.0 Hz, 3'-*H*), 6.77 (1H, d, *J* = 1.0 Hz, 4'-*H*), 4.10 (3H, s, N-CH_3), 3.91 – 3.86 (4H, m, 2,6-H_2), 3.42 – 3.38 (4H, m, 3,5-H_2); δ_C (151 MHz, Chloroform-*d*) δ 154.7 (*C*-5'), 133.4 (*C*-7'a), 131.3 (*C*-7'), 131.1 (*C*-3'), 130.9 (*C*-3'a), 94.9 (*C*-4'), 66.9 (*C*-2,6), 48.0 (*C*-3,5), 36.0 (N-CH_3); V_{max} (ATR) 1492, 1225, 1116, 967 cm^{-1}; LC-MS (ESI) [M + H] 219.214; HRMS (ESI) found $[M+H]^+$ 219.1254, $C_{11}H_{15}N_4O$ requires *M* 219.1246.

*Compound **43***
4-[3'-(3'',5''-dimethylphenyl)-1'-methyl-*1H*-pyrazolo[3,4-c]pyridin-5'-yl]morpholine

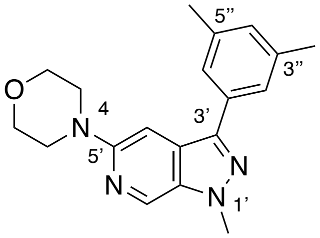

General procedure B was applied to 4-{1'-methyl-*1H*-pyrazolo[3,4-c]pyridin-5'-yl}morpholine **42** (0.130 g, 0.60 mmol, 1.00 eq) with 1-iodo-3,5-dimethylbenzene (0.09 mL, 0.66 mmol, 1.10 eq). Purification by silica gel flash column chromatography (EtOAc:Pet Ether 40-60 0-80%) afforded 4-[3'-(3'',5''-dimethylphenyl)-1'-methyl-*1H*-pyrazolo[3,4-c]pyridin-5'-yl]morpholine **43** as a yellow solid (0.106 g, 0.33 mmol, 55%) with mp 118-120 ˚C.

δ_H (700 MHz, Chloroform-*d*) 8.70 (1H, d, *J* = 1.3 Hz, 7'-*H*), 7.55 (2H, m, 2'',6''-*H*), 7.11 – 7.09 (1H, m, 4''-*H*), 7.08 (1H, d, *J* = 1.3 Hz, 4'-*H*), 4.20 (3H, s, NCH_3), 3.99 – 3.95 (4H, m, 2,6-H_2), 3.52 – 3.48 (4H, m, 3,5-H_2), 2.47 (6H, q, *J* = 0.8 Hz, Ar-CH_3); δ_C (176 MHz, Chloroform-*d*) 155.1 (*C*-5'), 142.7 (*C*-3'), 138.5 (*C*-3'',5''), 134.8 (*C*-7a), 133.0 (*C*-1''), 131.6 (*C*-7), 129.7 (*C*-4''), 128.5 (*C*-3a), 124.8 (*C*-2'',6''), 95.3 (*C*-4'), 66.9 (*C*-2,6), 48.1 (*C*-3,5), 36.1 (NCH_3), 21.5 Ar-CH_3); V_{max} (ATR) 1617, 1488, 1451, 1231, 1122, 963 cm^{-1}; LC-MS (ESI) [M + H] 323.346; HRMS (ESI) found $[M+H]^+$ 323.1876, $C_{19}H_{23}N_4O$ requires *M* 323.1872.

^1H NMR (400 MHz, Chloroform-*d*) and ^{13}C NMR (101 MHz, Chloroform-*d*) of compound **3a**

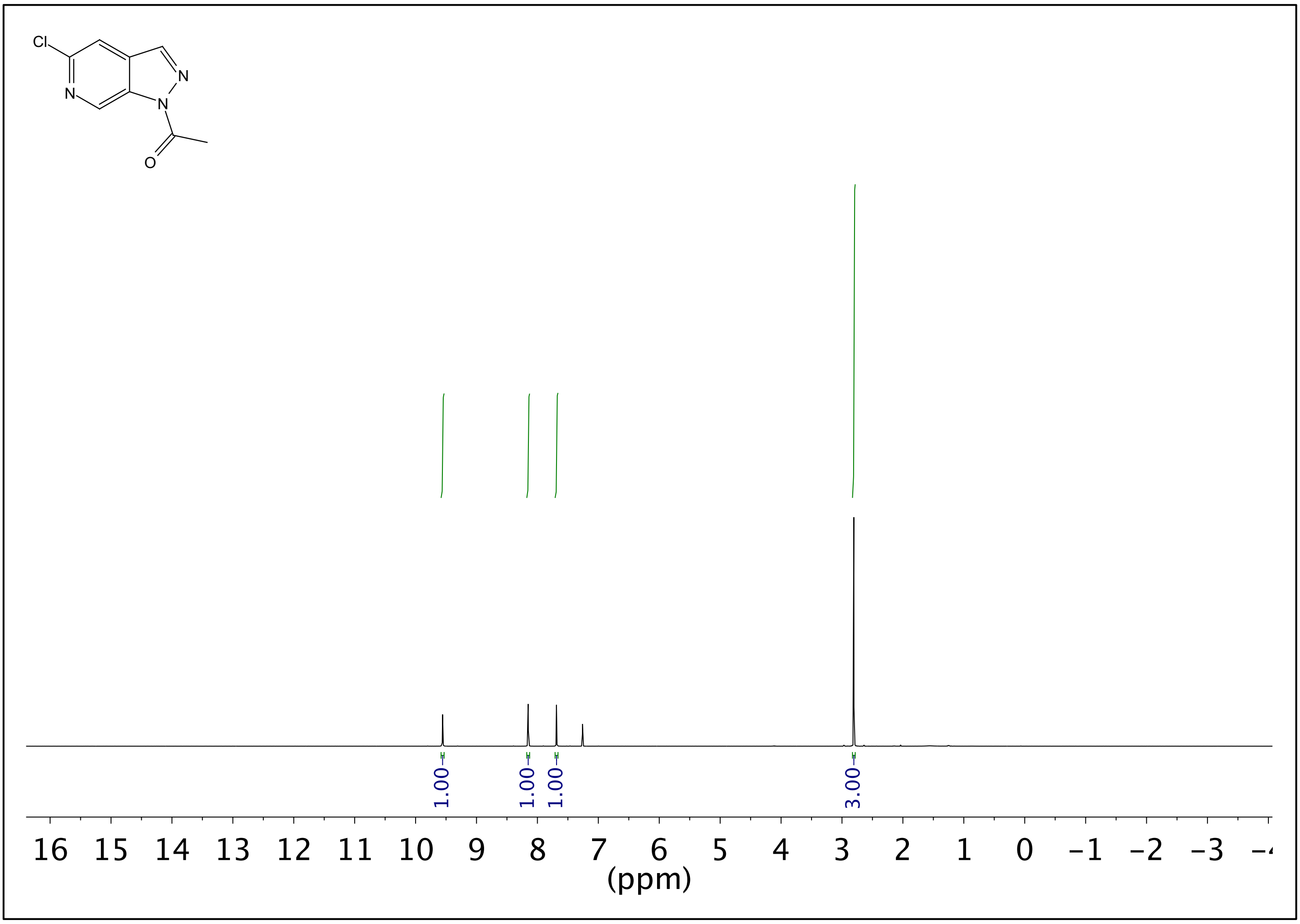

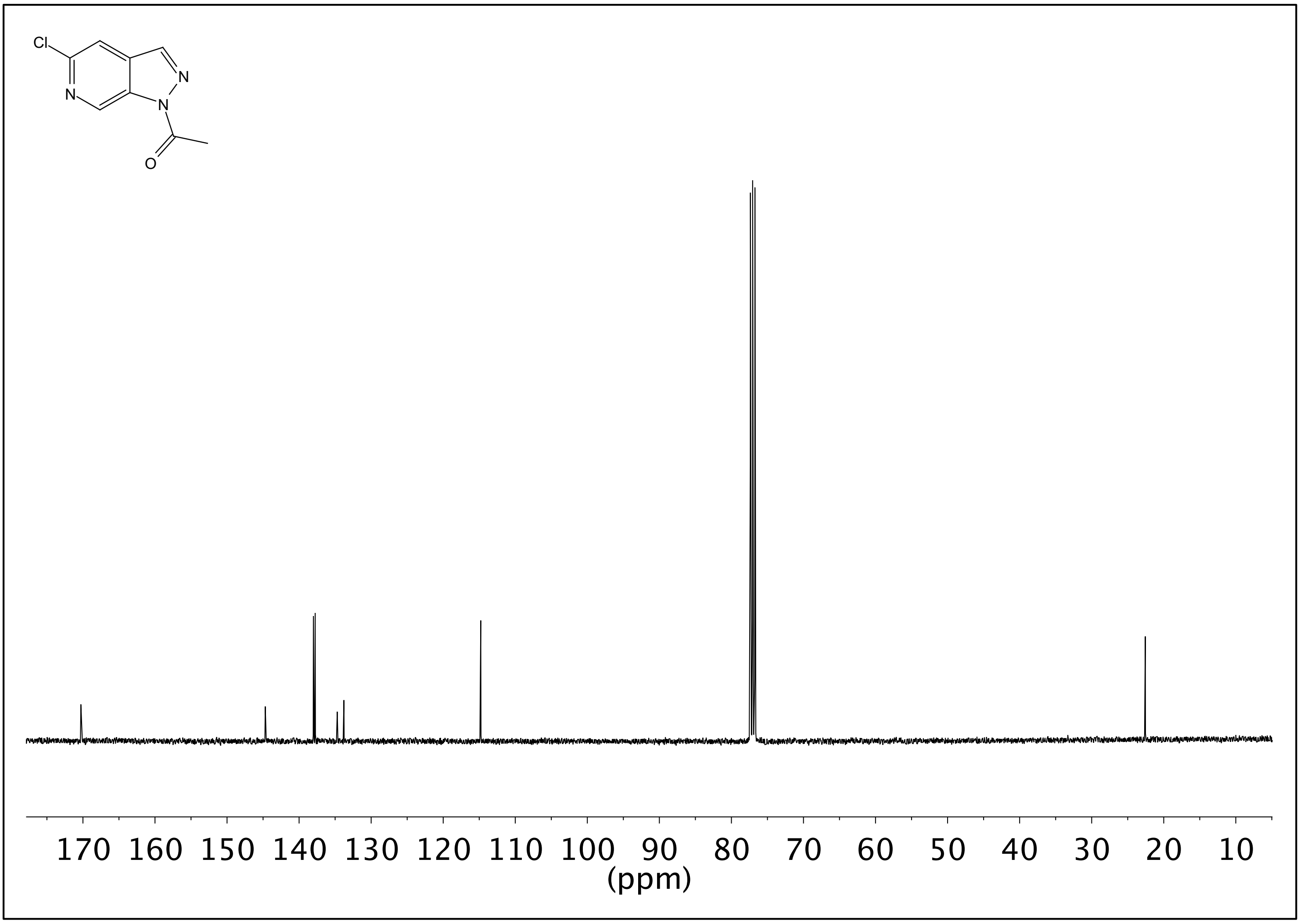

^{1}H NMR (400 MHz, Methanol-*d*) and ^{13}C NMR (101 MHz, Methanol-*d*) of compound **4a**

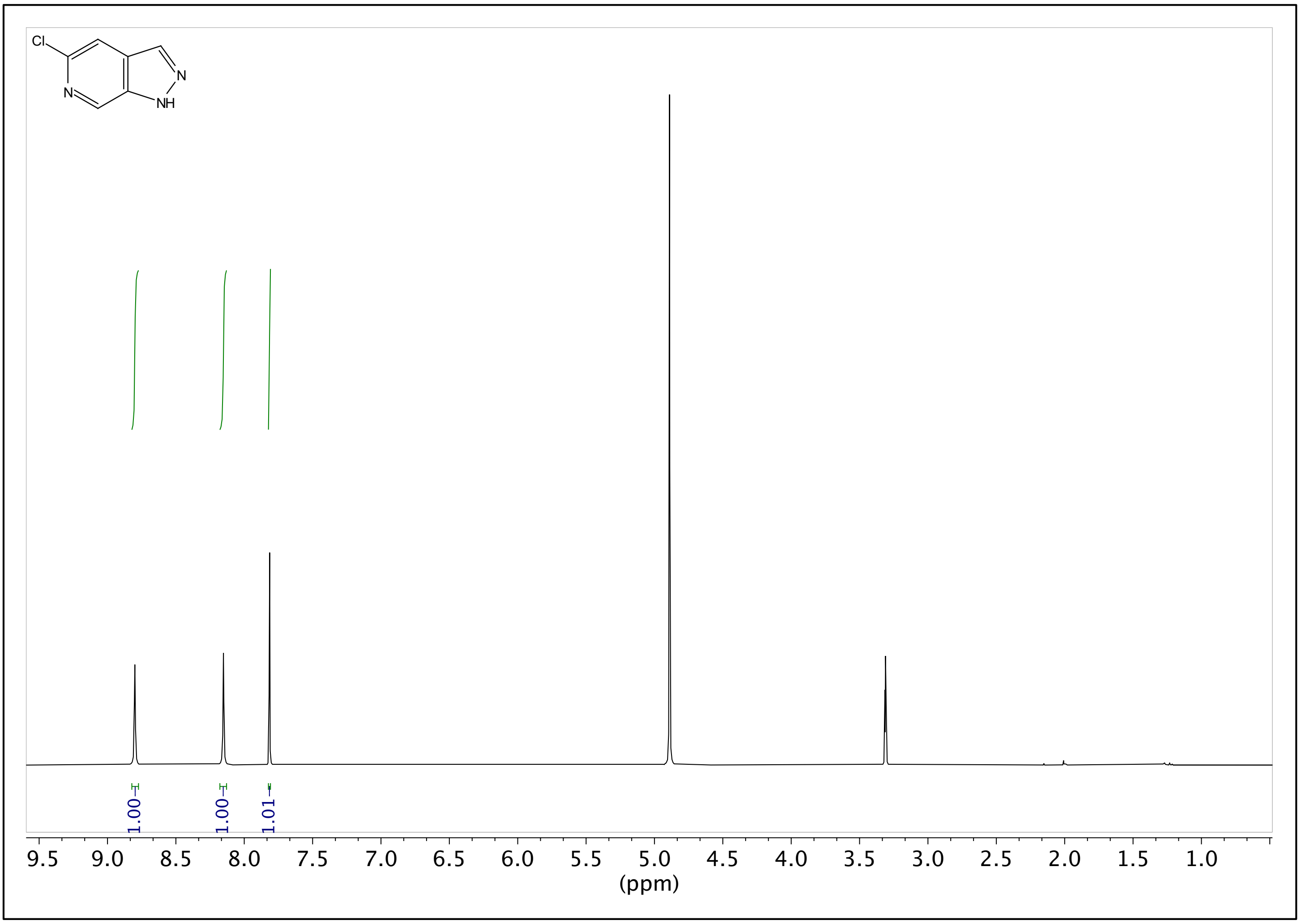

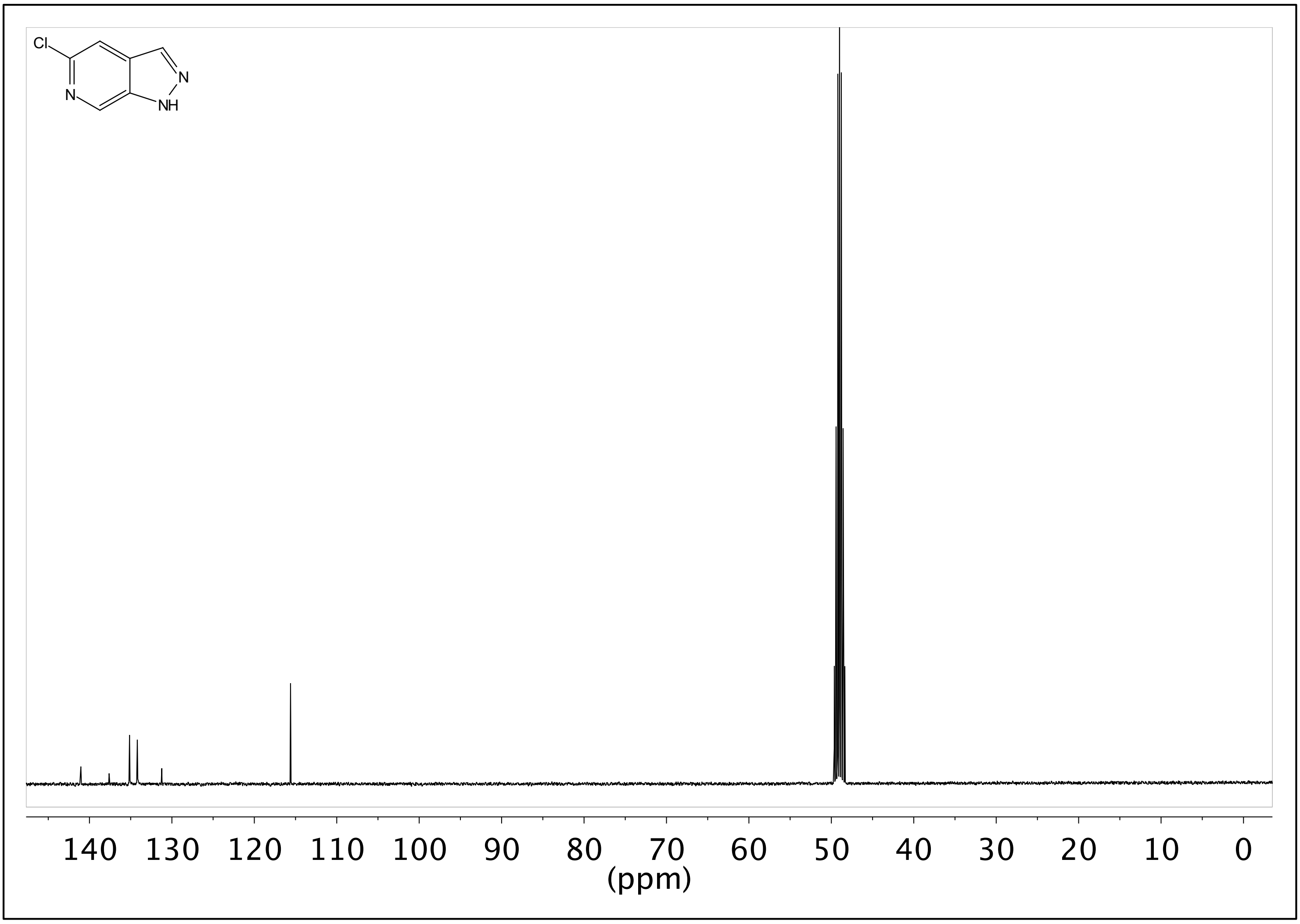

^{1}H NMR (600 MHz, Chloroform-*d*) and ^{13}C NMR (151 MHz, Chloroform-*d*) of compound **3b**

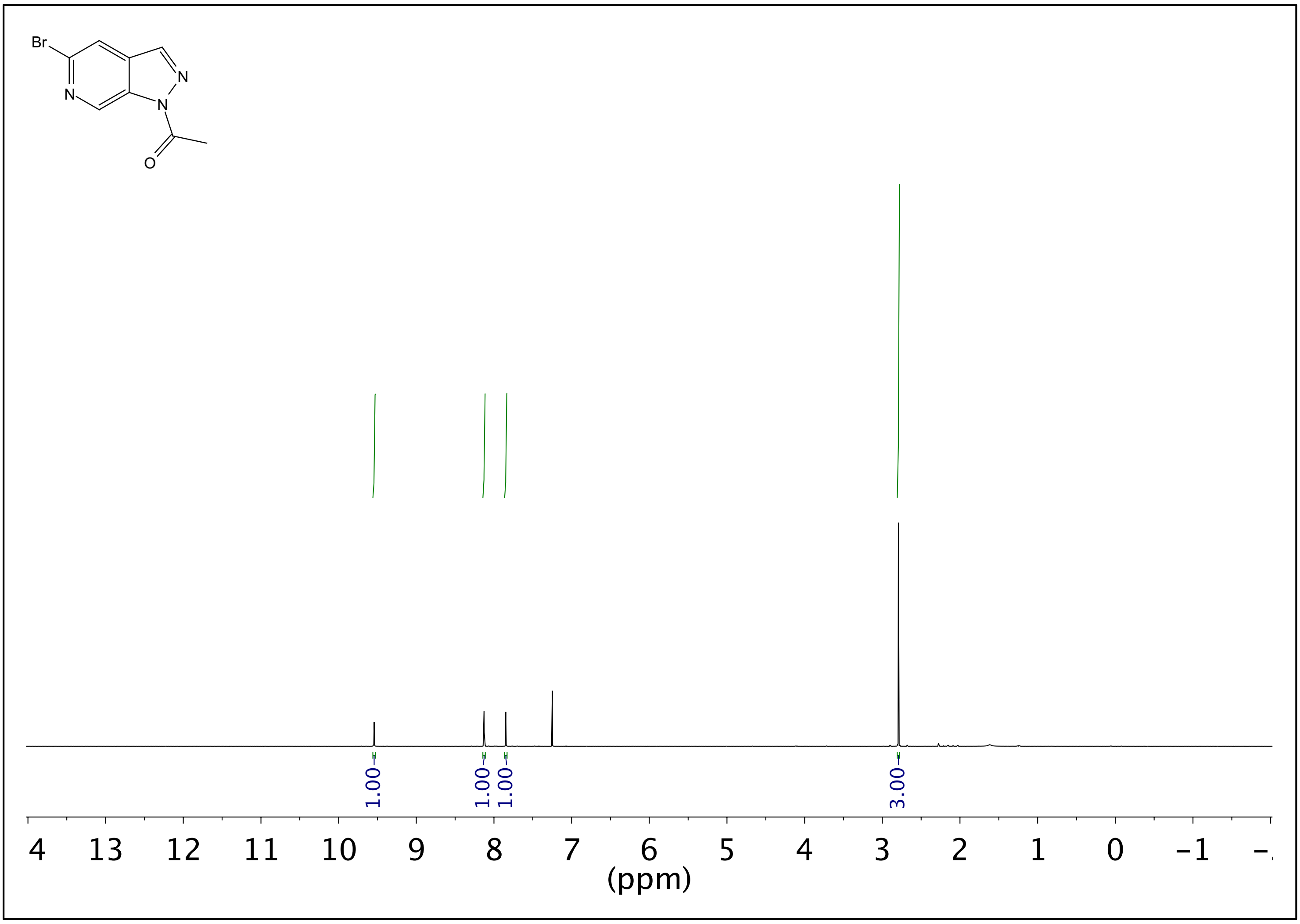

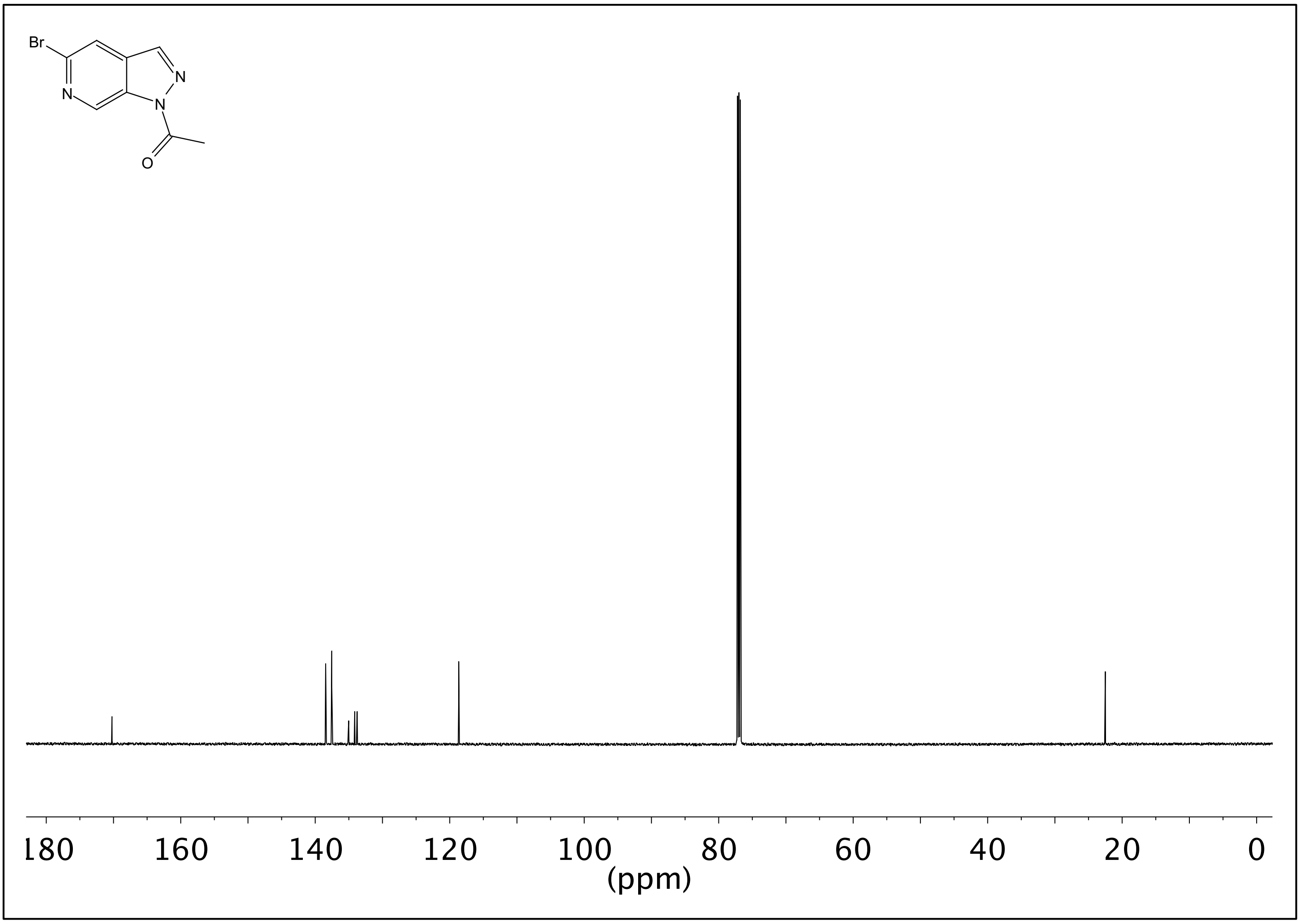

^{1}H NMR (600 MHz, Methanol-*d*) and ^{13}C NMR (151 MHz, Methanol-*d*) of compound **4b**

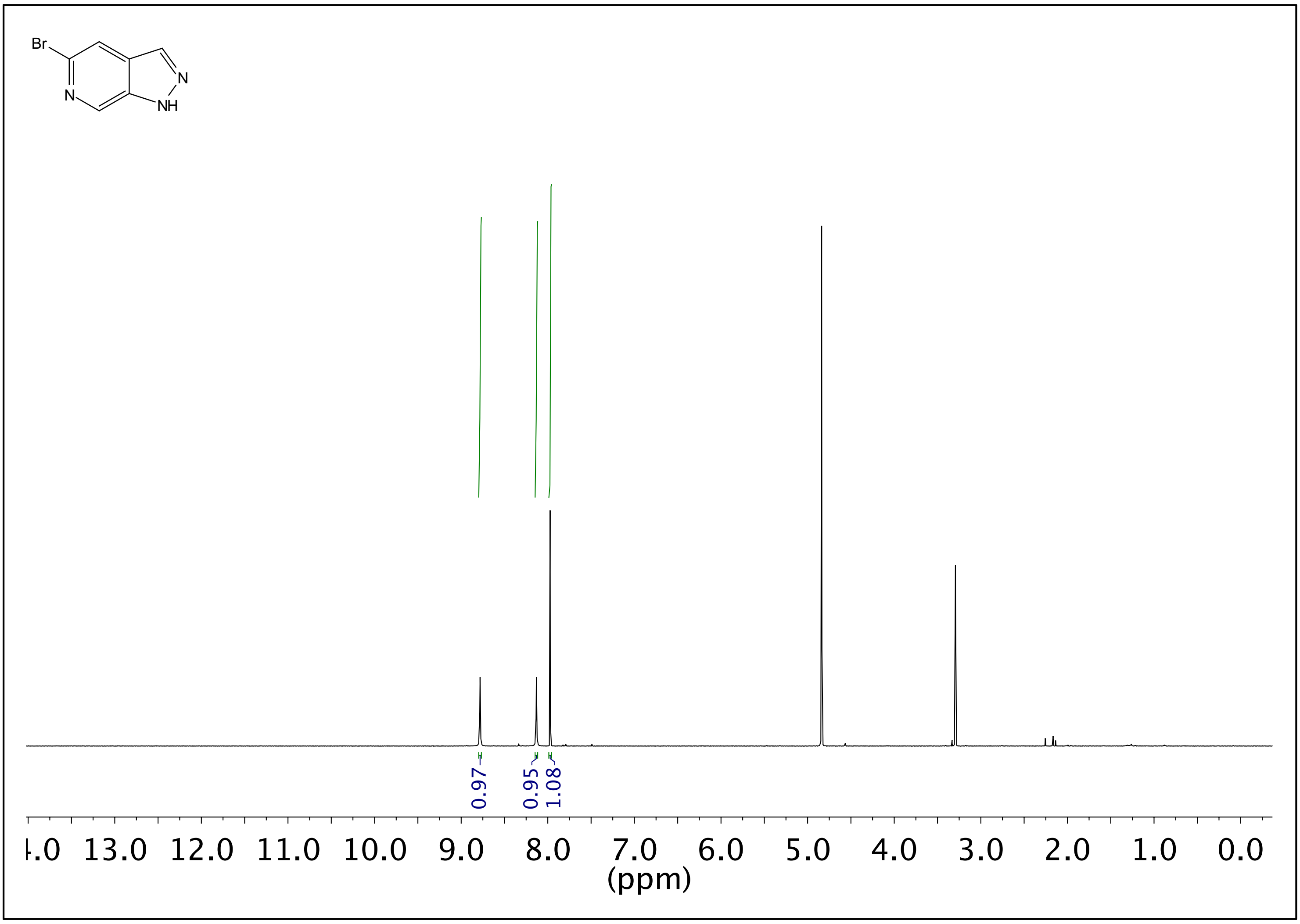

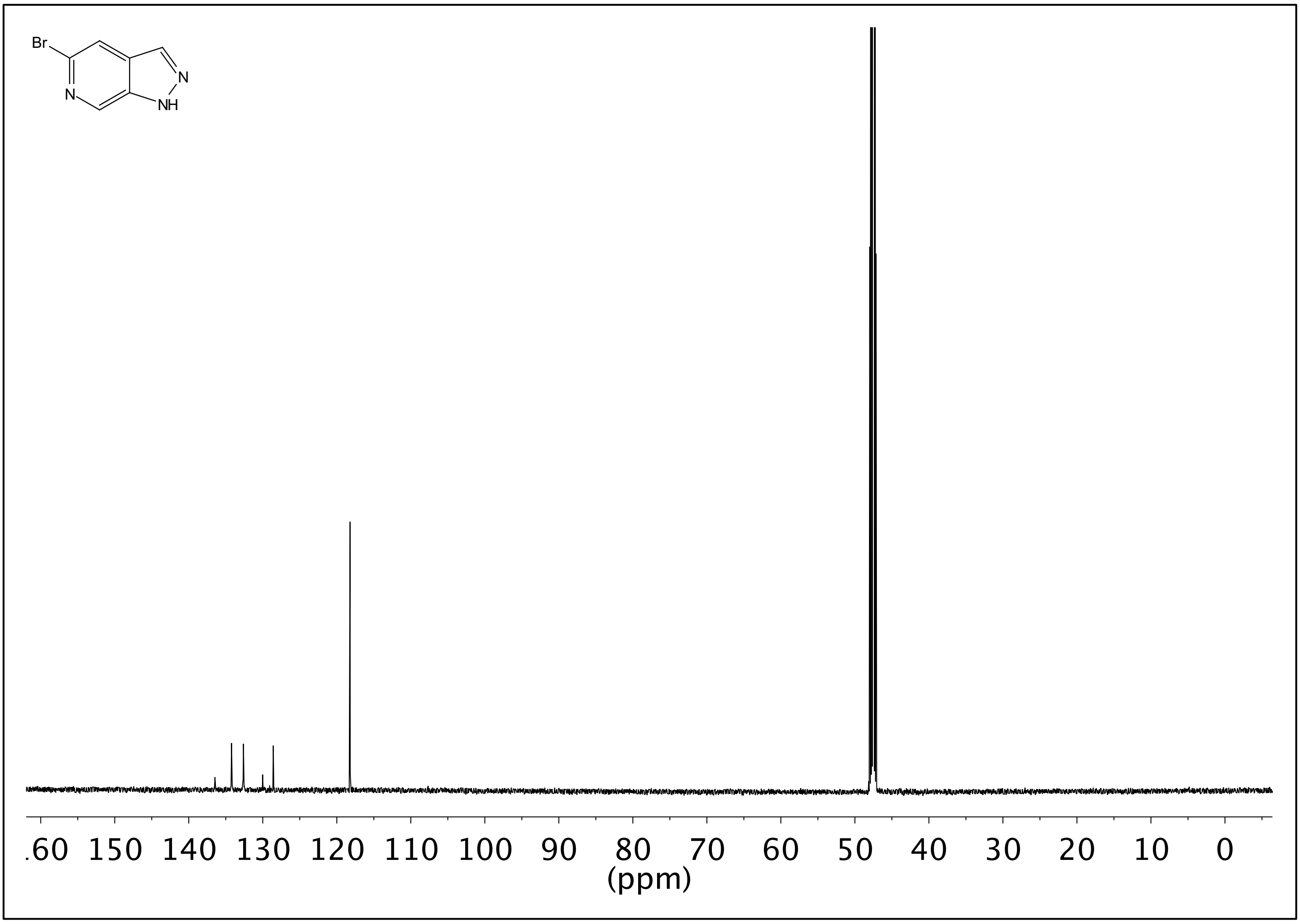

^{1}H NMR (600 MHz, Chloroform-*d*) and ^{13}C NMR (151 MHz, Chloroform-*d*) of compound **5a**

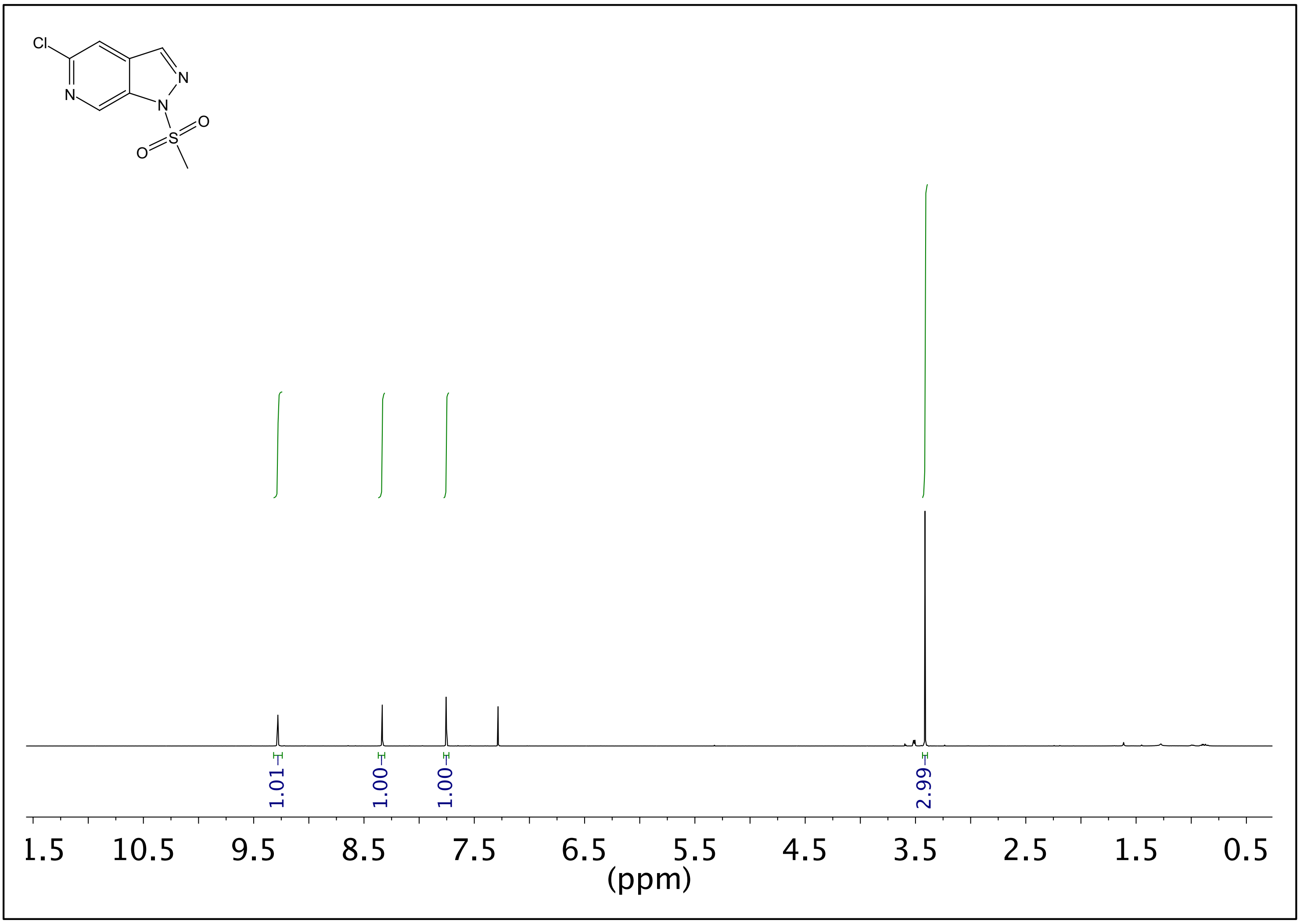

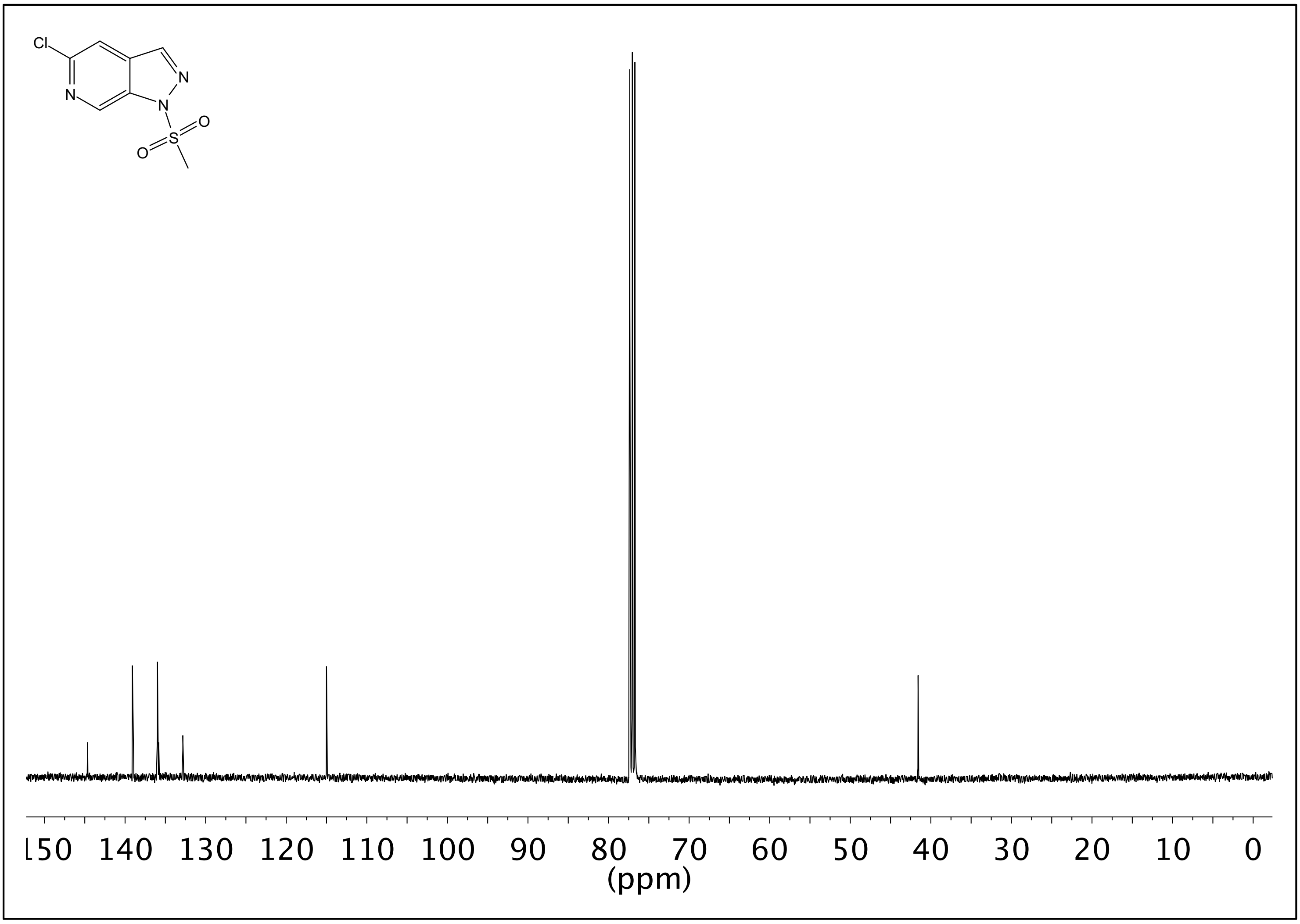

^{1}H NMR (600 MHz, Chloroform-*d*) and ^{13}C NMR (151 MHz, Chloroform-*d*) of compound **6a**

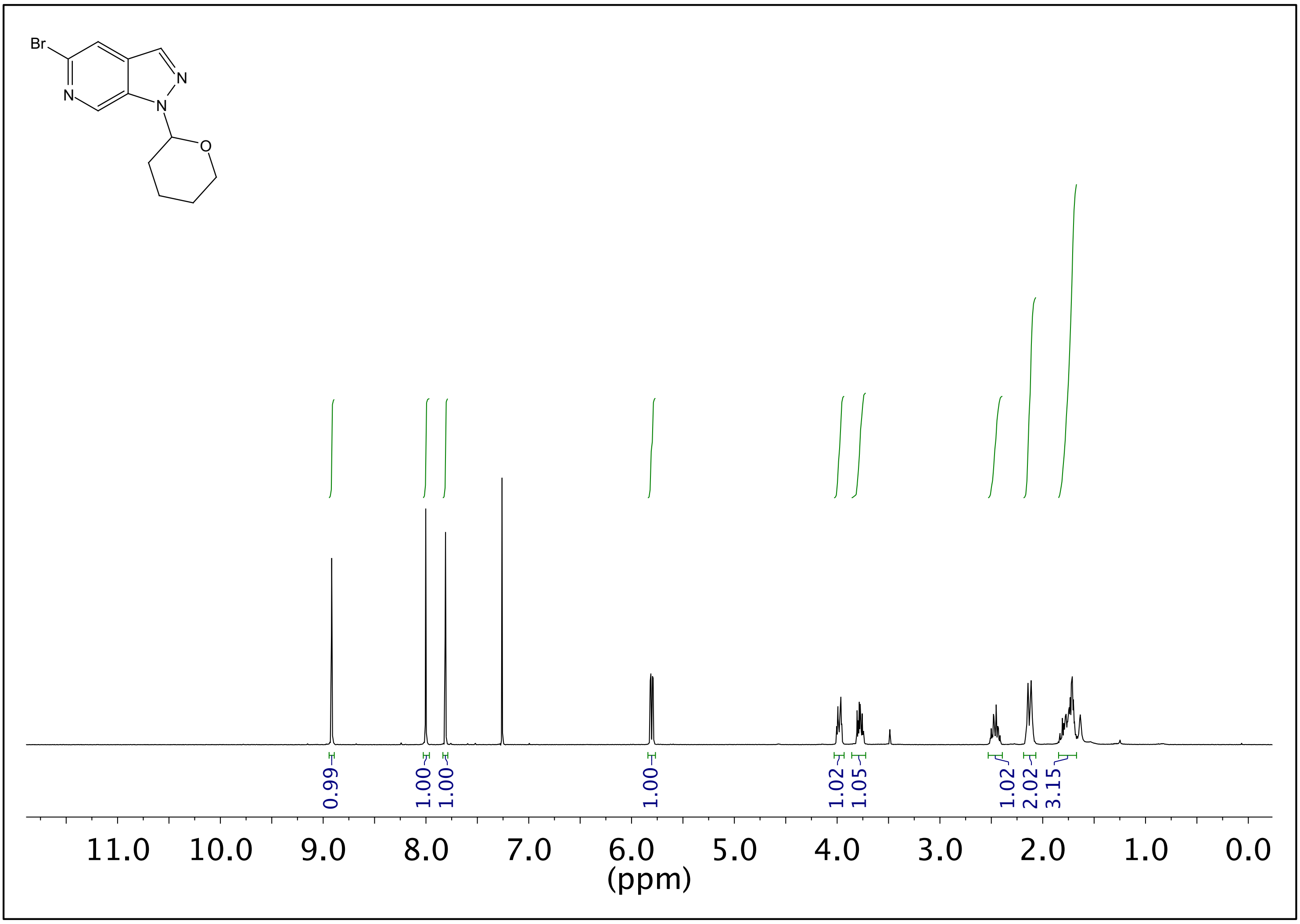

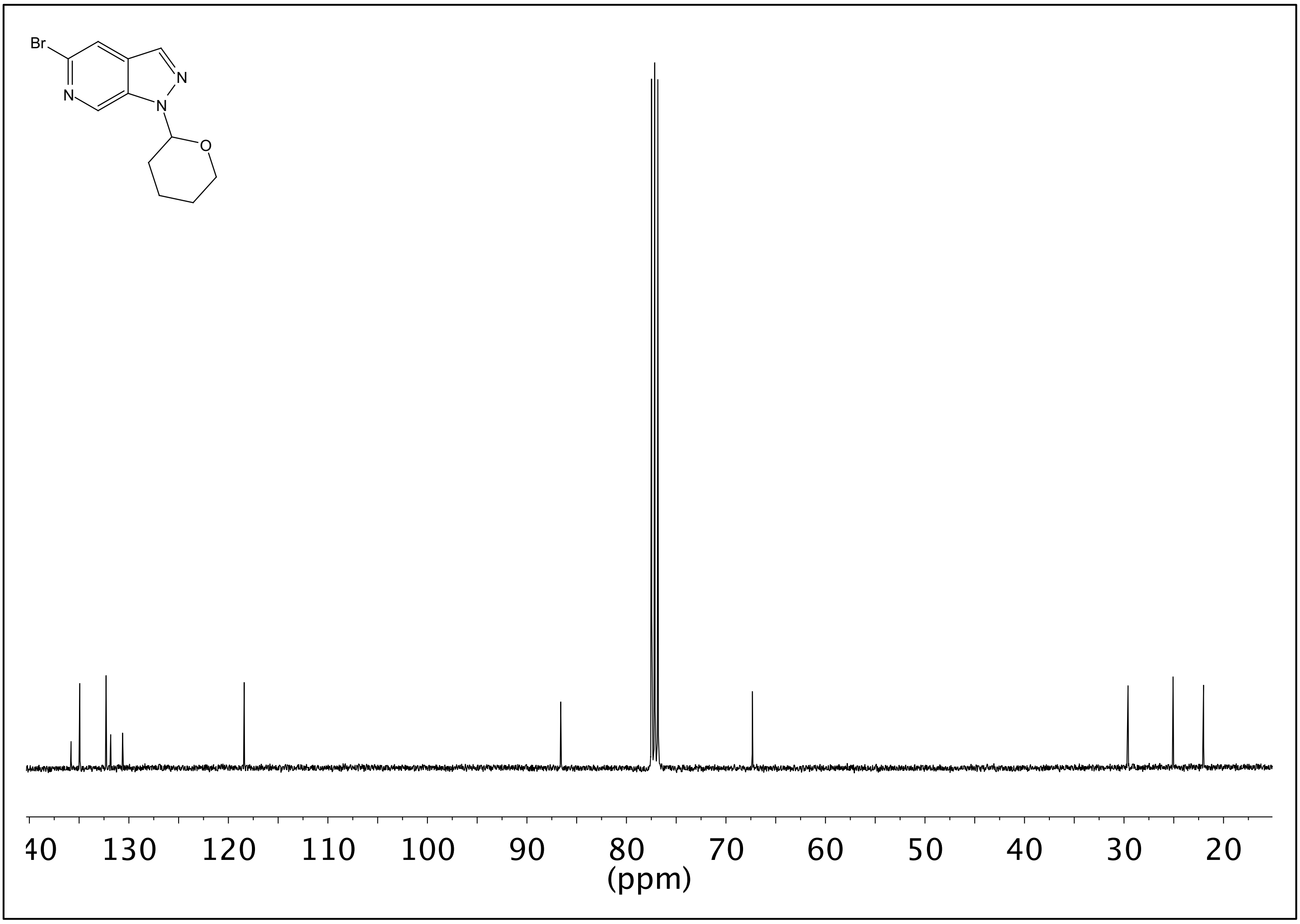

^{1}H NMR (600 MHz, Chloroform-*d*) and ^{13}C NMR (151 MHz, Chloroform-*d*) of compound **6b**

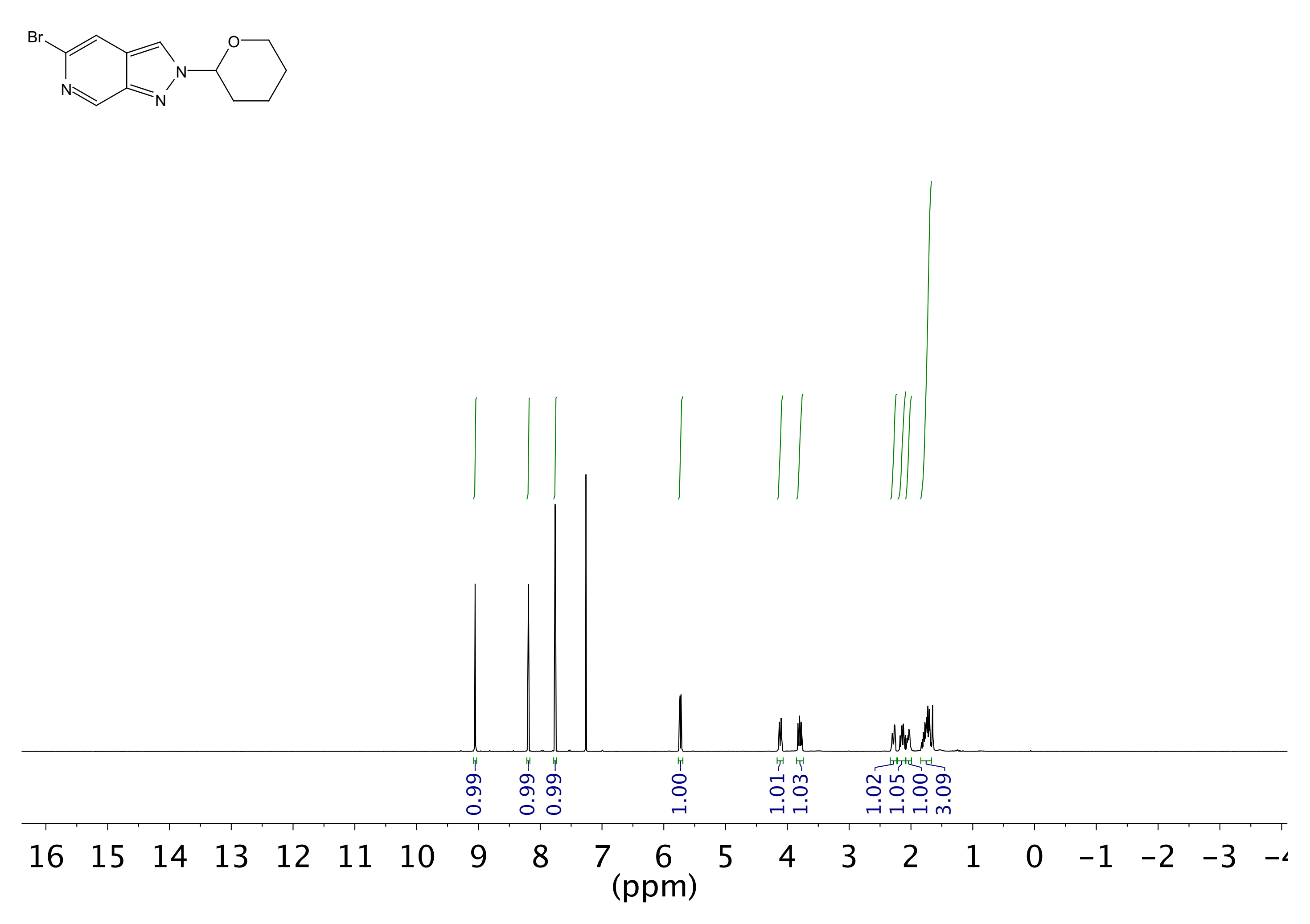

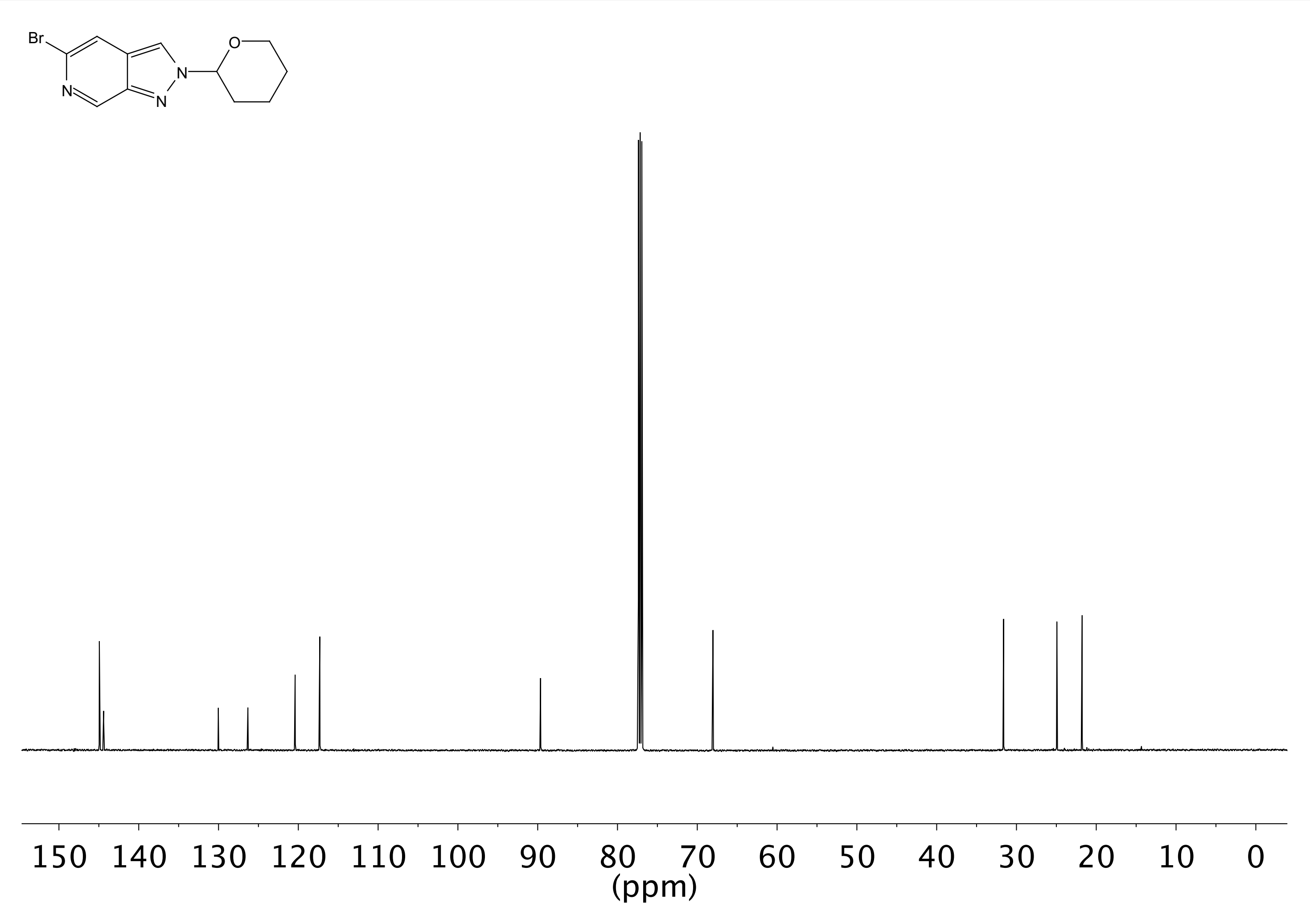

^{1}H NMR (600 MHz, Chloroform-*d*) and ^{13}C NMR (151 MHz, Chloroform-*d*) of compound **7a**

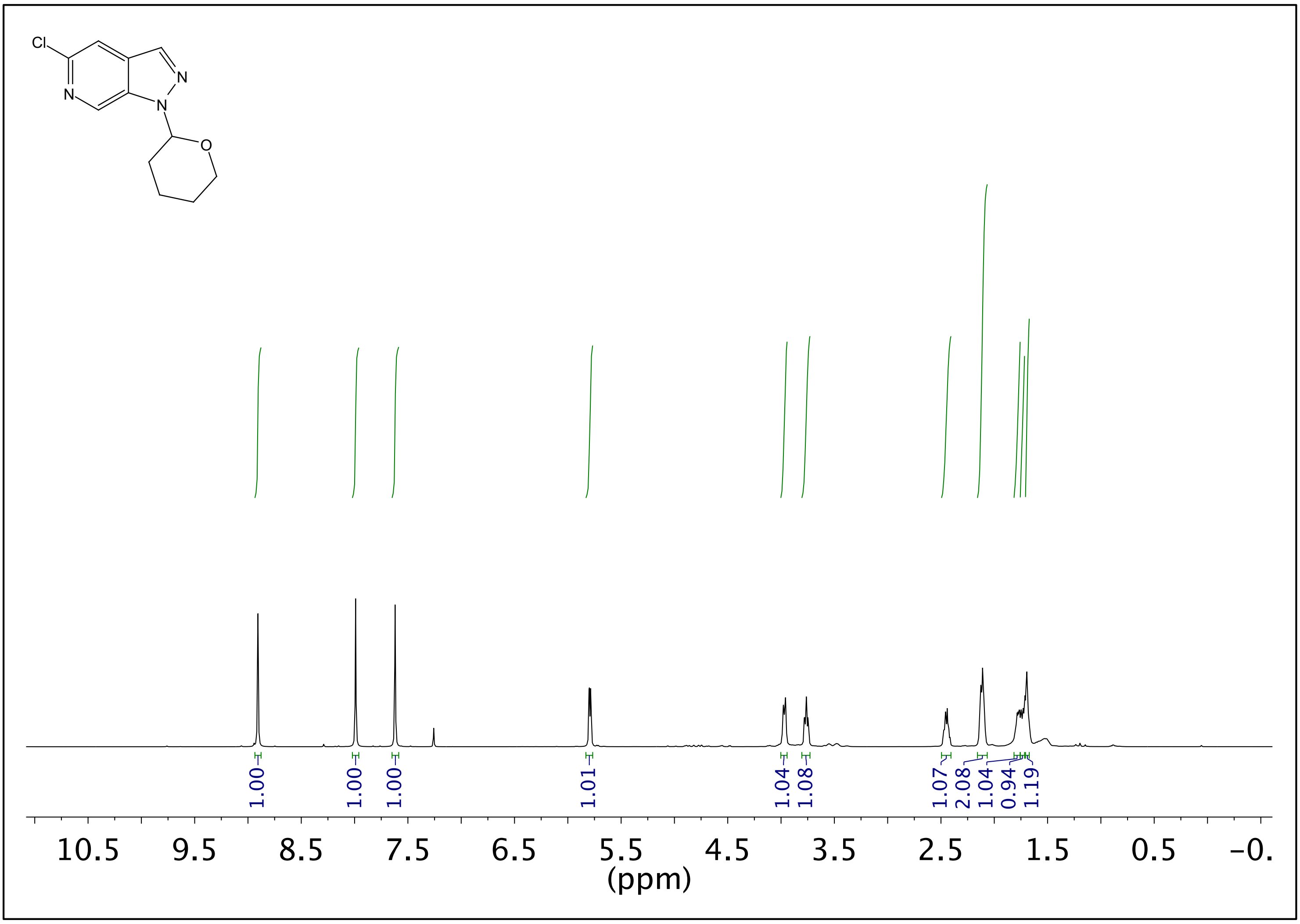

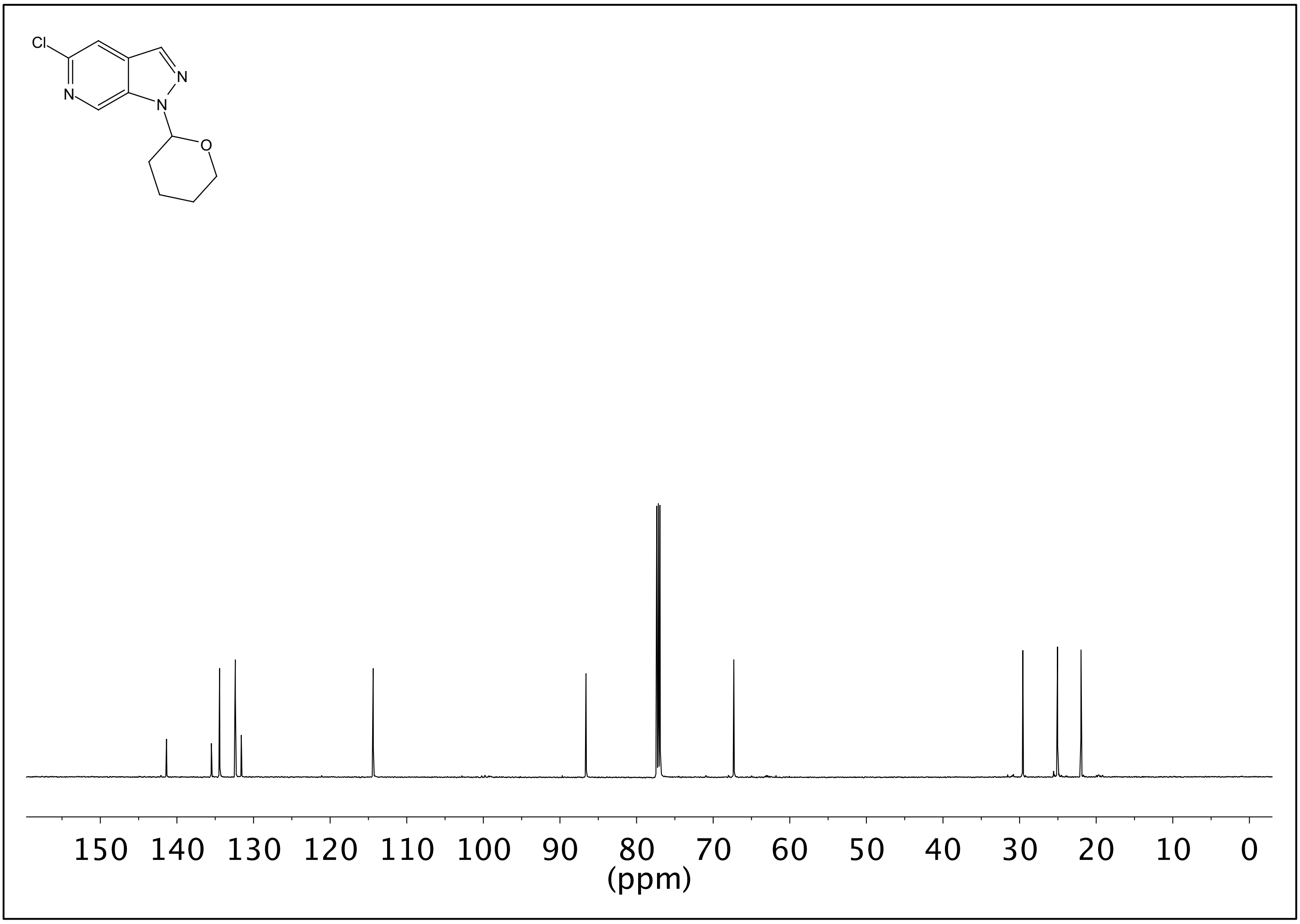

^{1}H NMR (600 MHz, Chloroform-*d*) and ^{13}C NMR (151 MHz, Chloroform-*d*) of compound **7b**

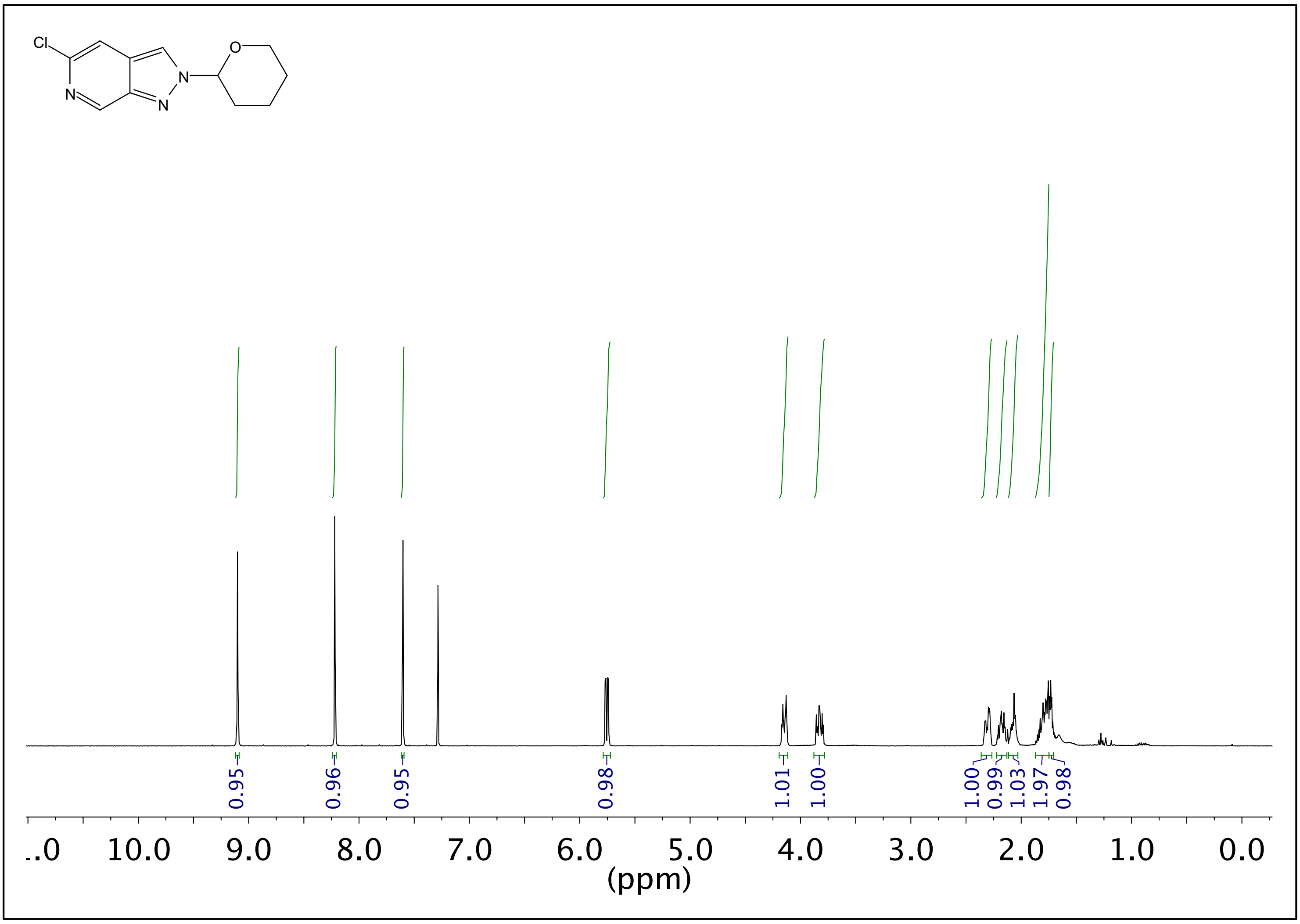

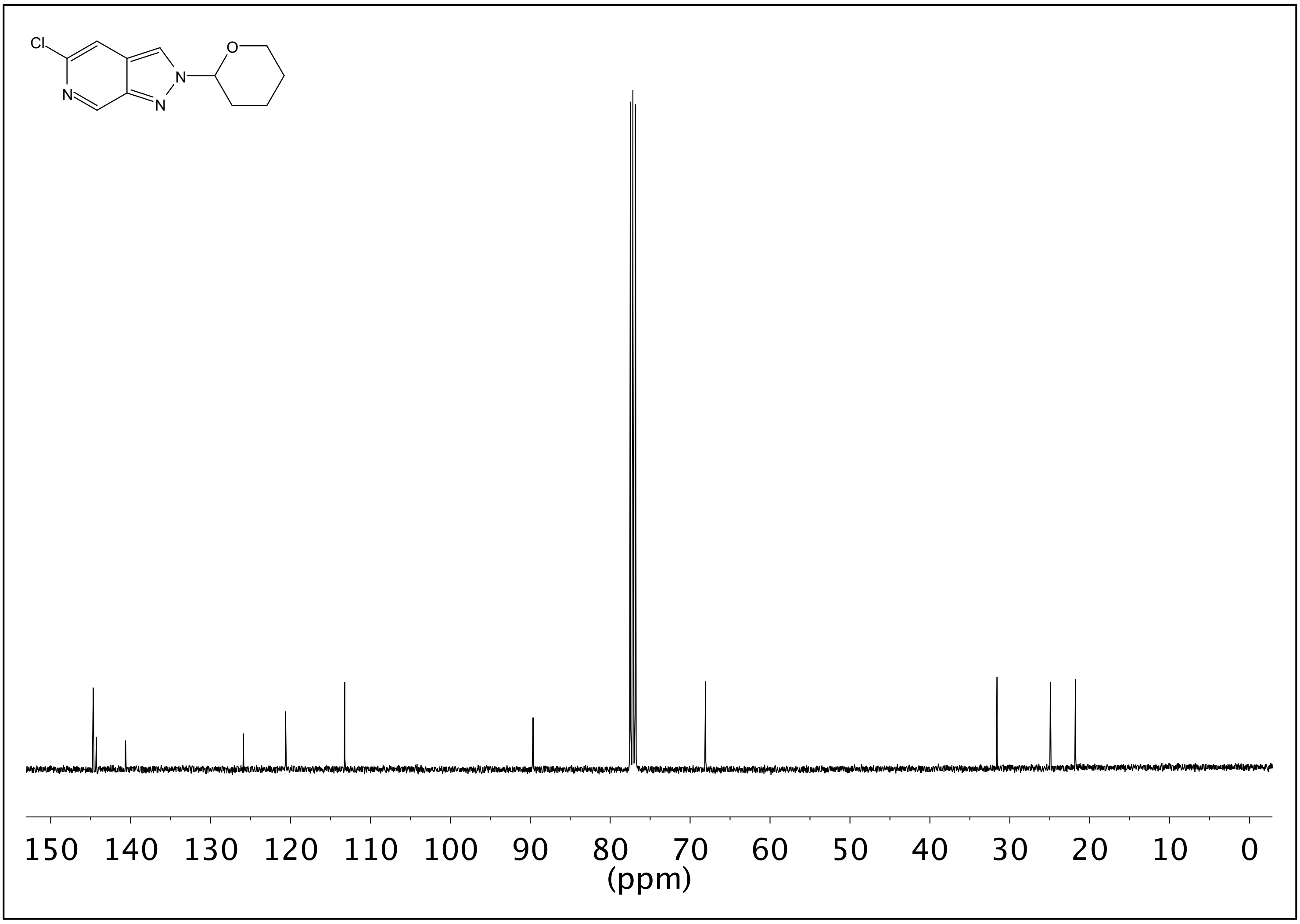

^{1}H NMR (600 MHz, Chloroform-*d*) and ^{13}C NMR (151 MHz, Chloroform-*d*) of compound **8a**

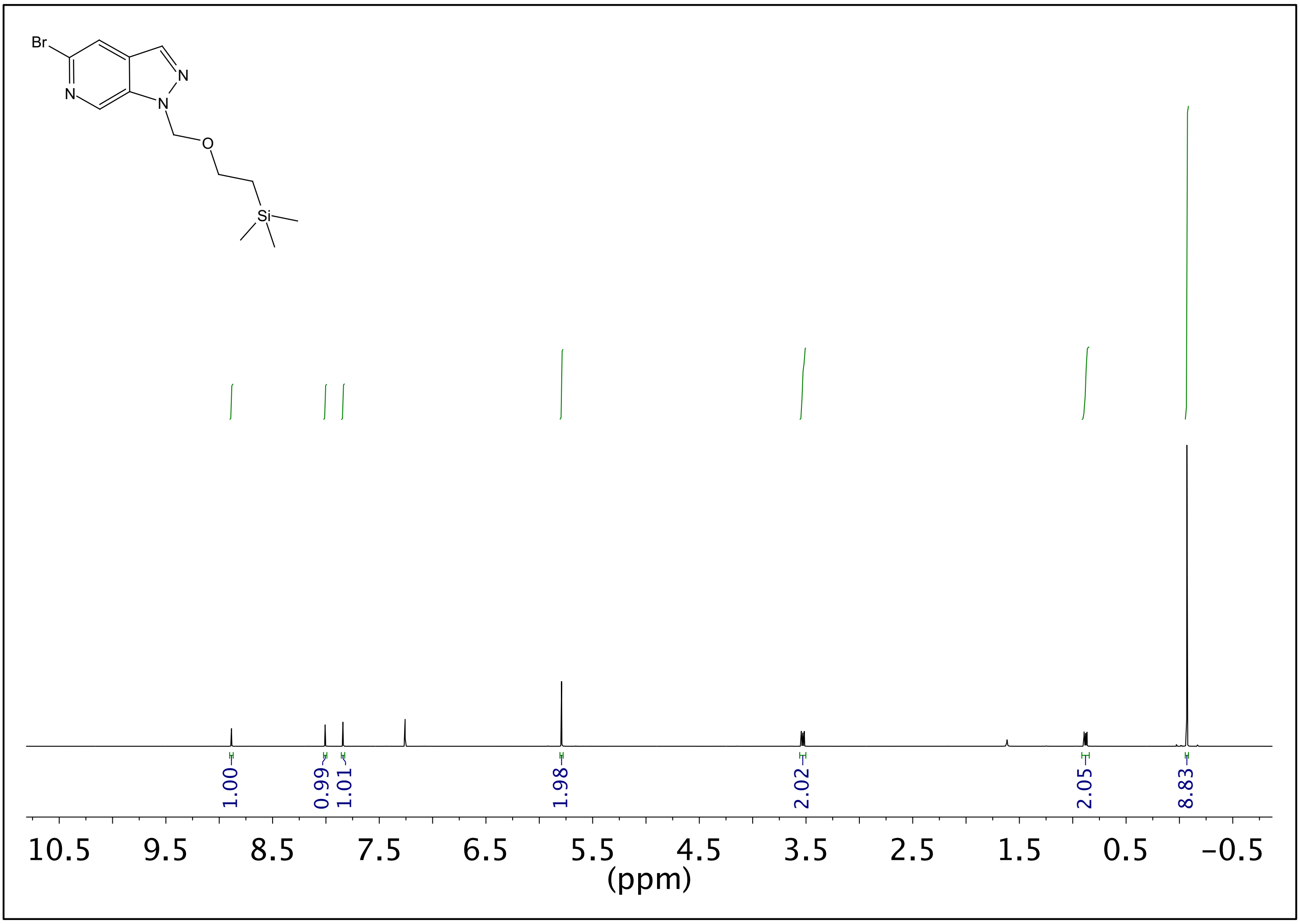

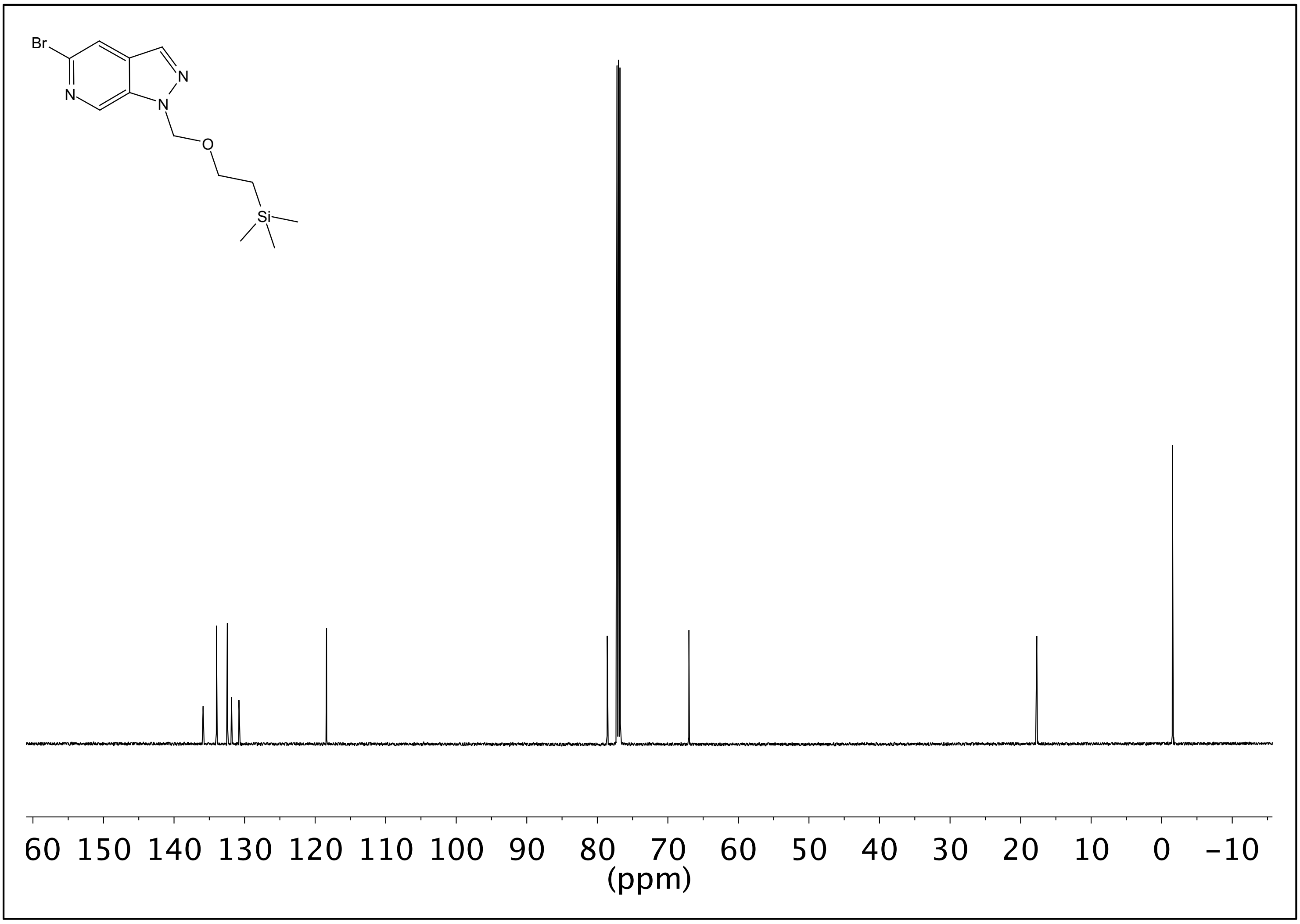

^{1}H NMR (600 MHz, Chloroform-*d*) and ^{13}C NMR (151 MHz, Chloroform-*d*) of compound **8b**

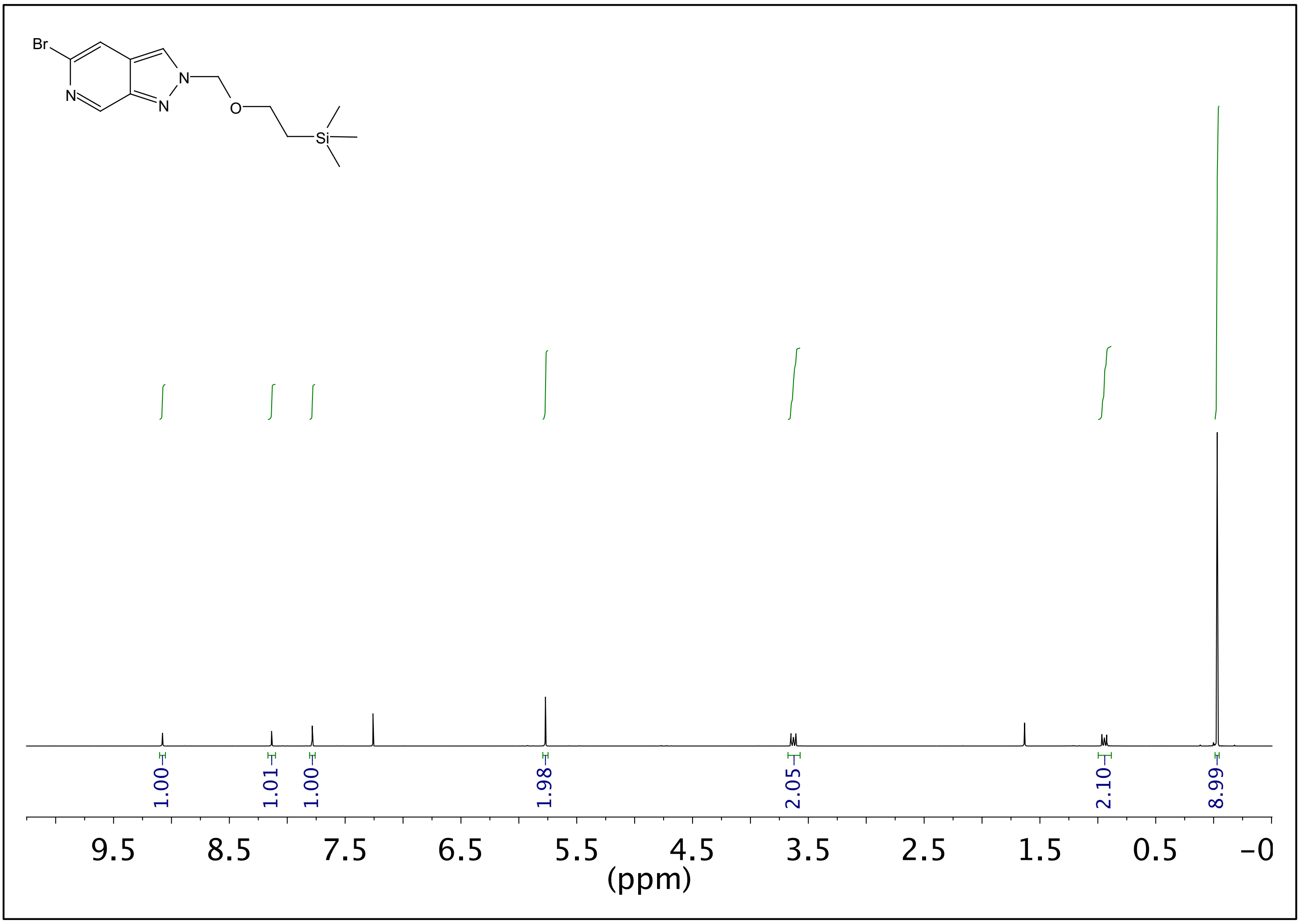

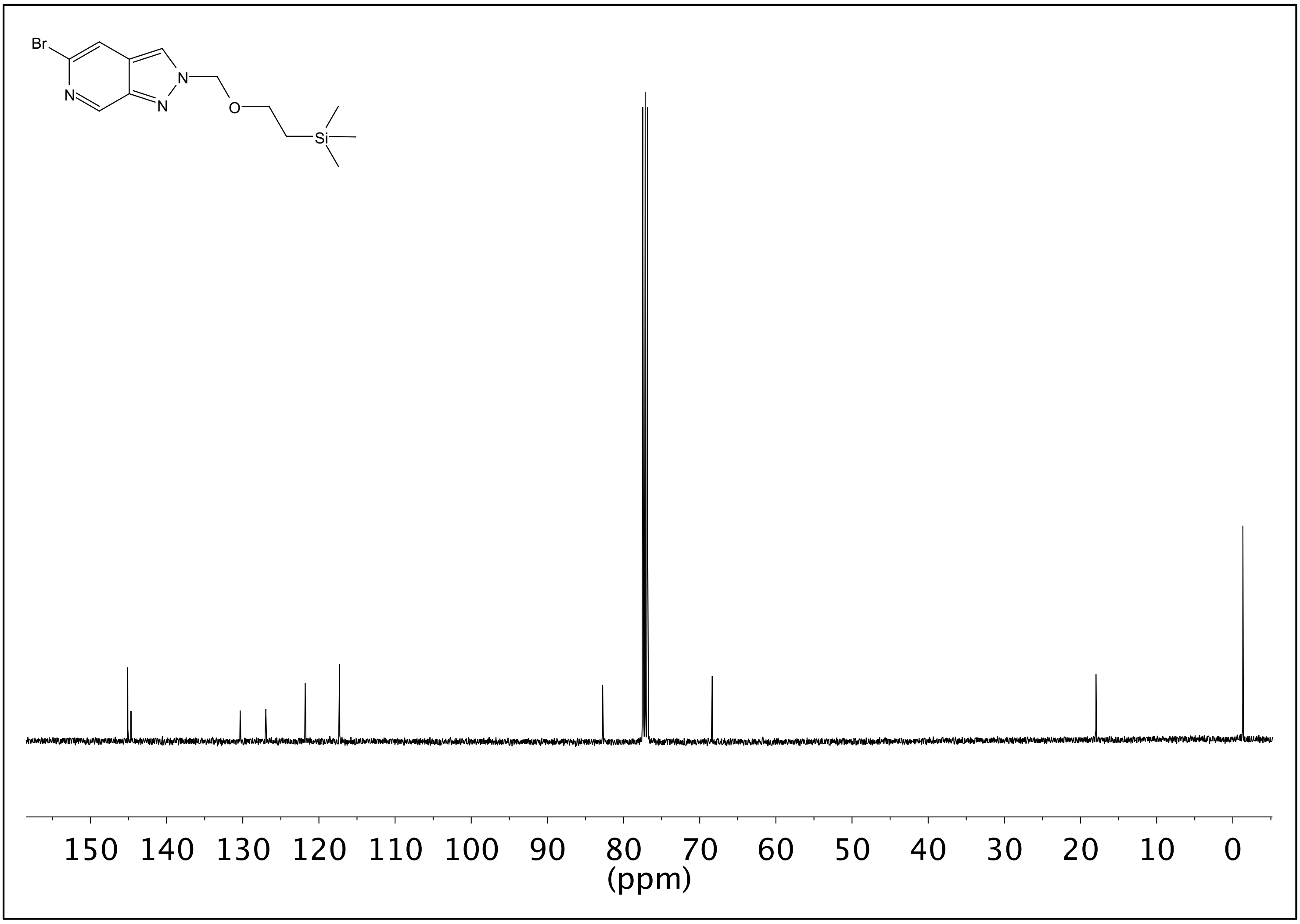

^{1}H NMR (400 MHz, Chloroform-*d*) and ^{13}C NMR (101 MHz, Chloroform-*d*) of compound **9a**

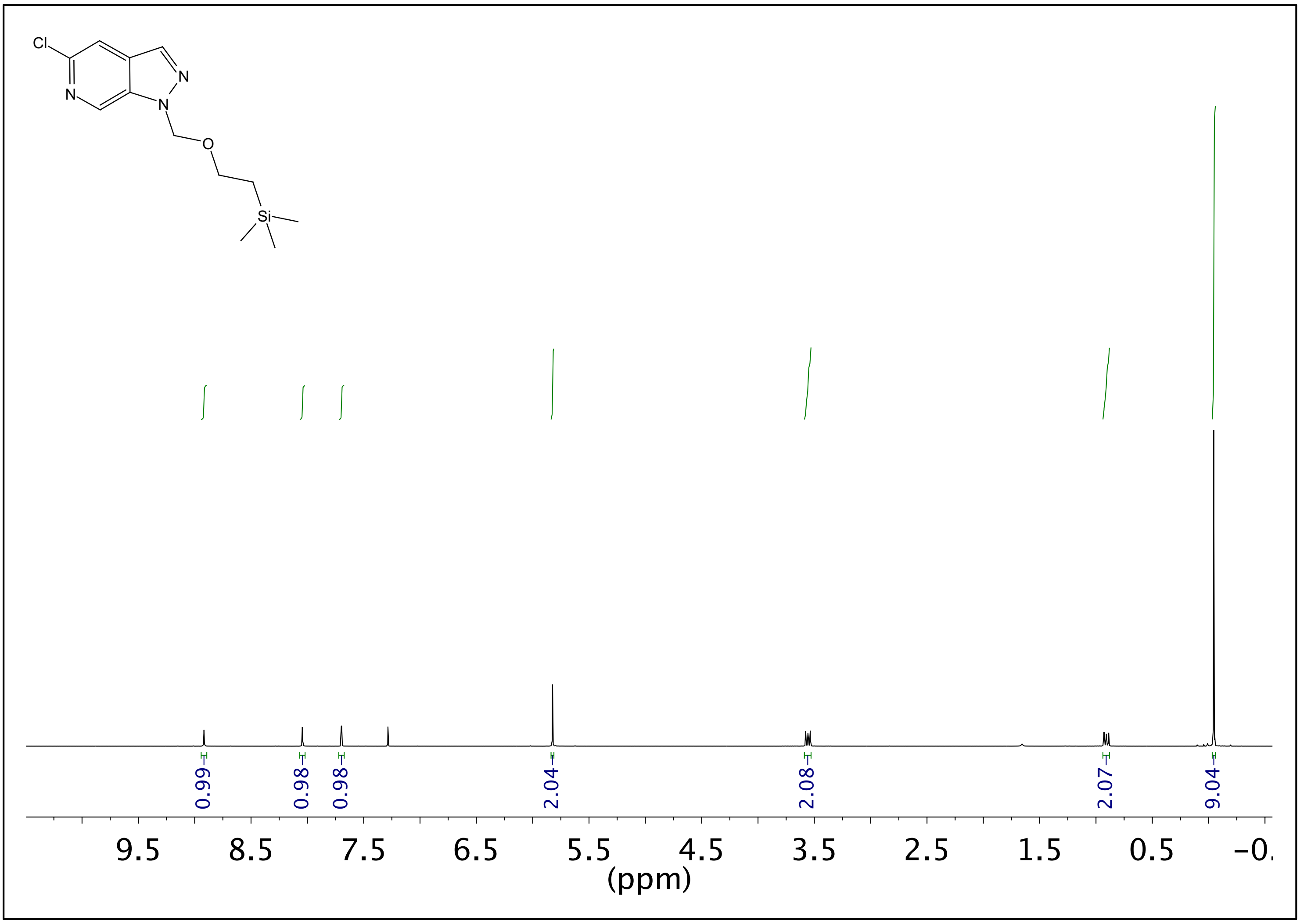

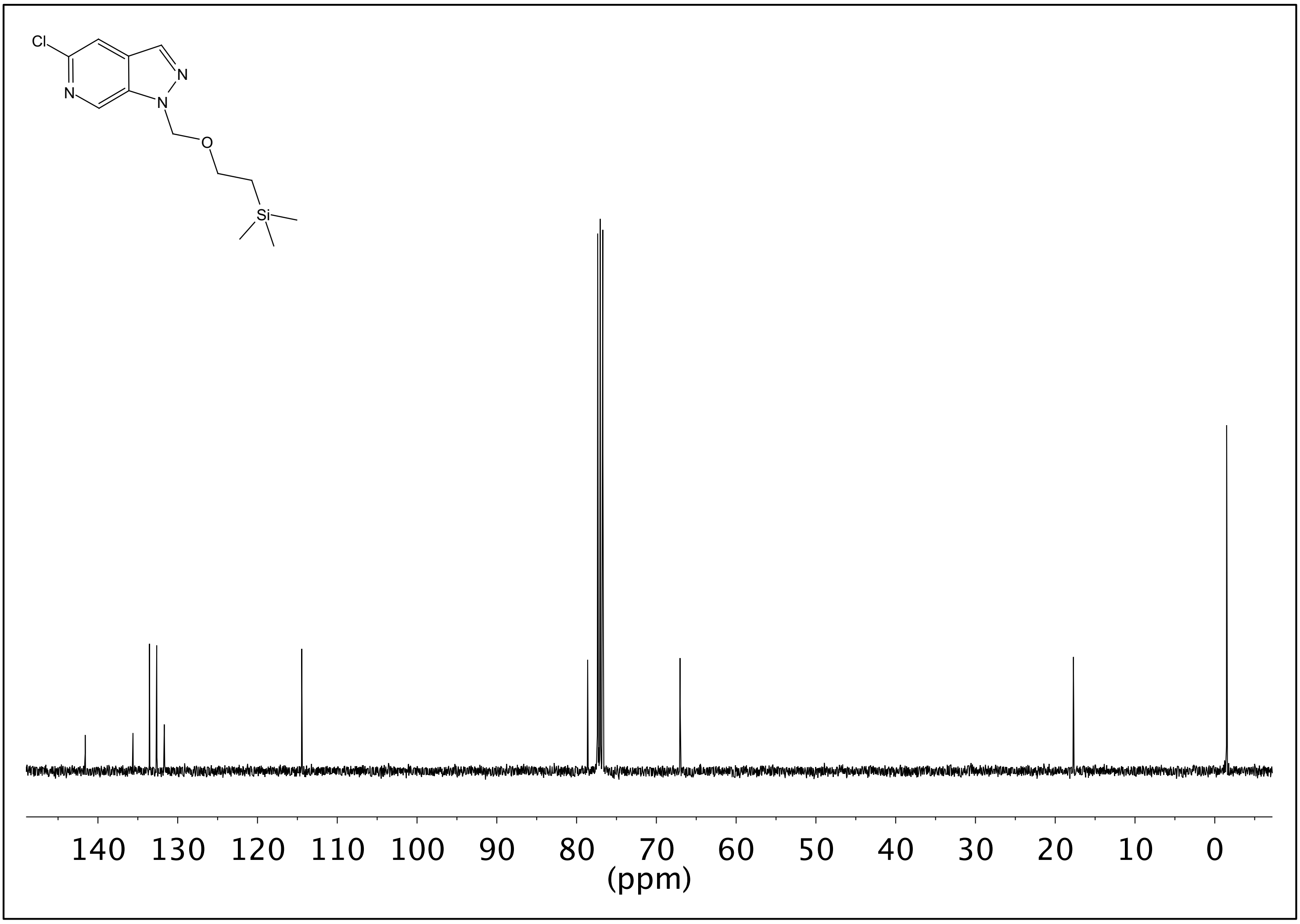

^{1}H NMR (400 MHz, Chloroform-*d*) and ^{13}C NMR (101 MHz, Chloroform-*d*) of compound **9b**

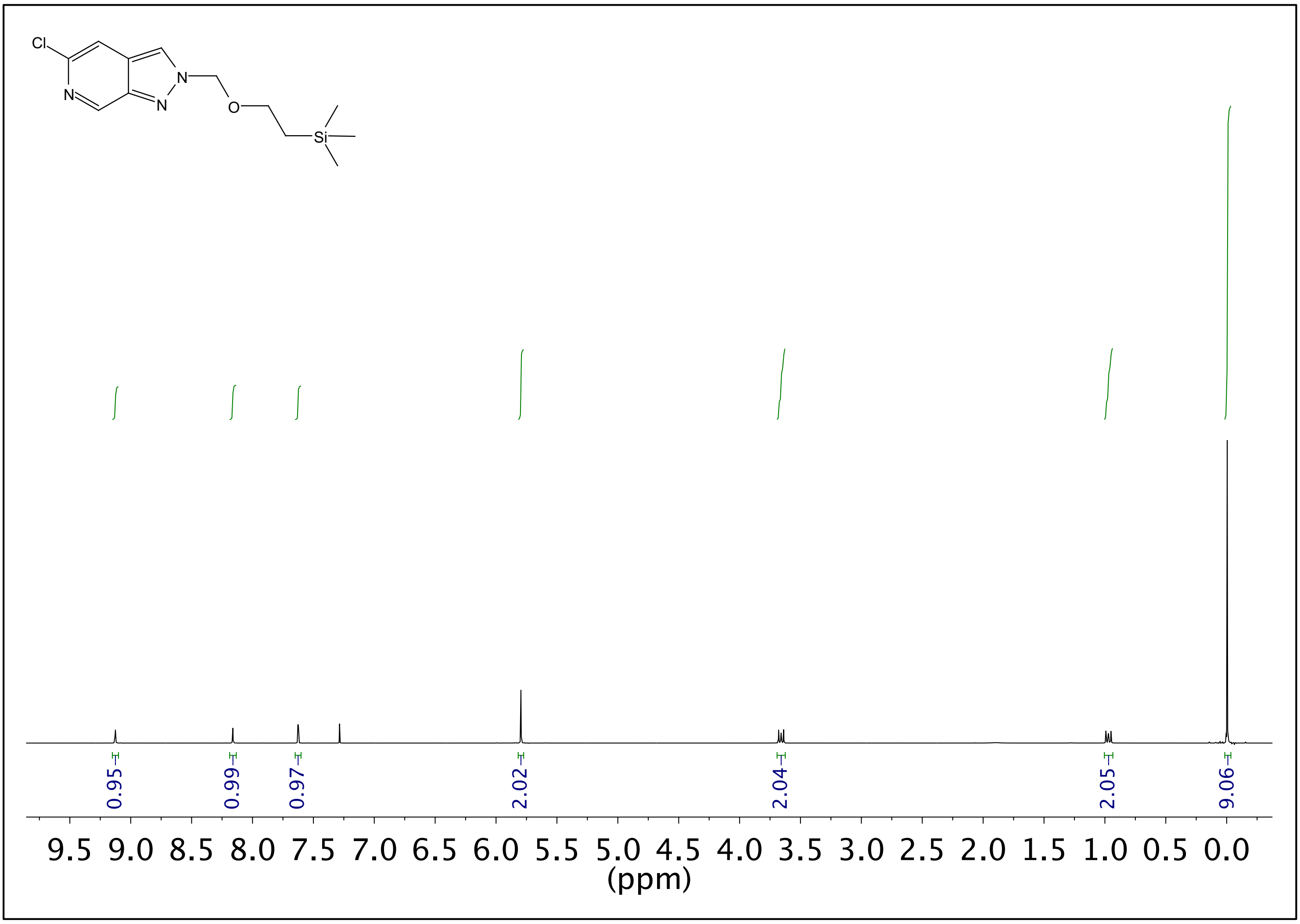

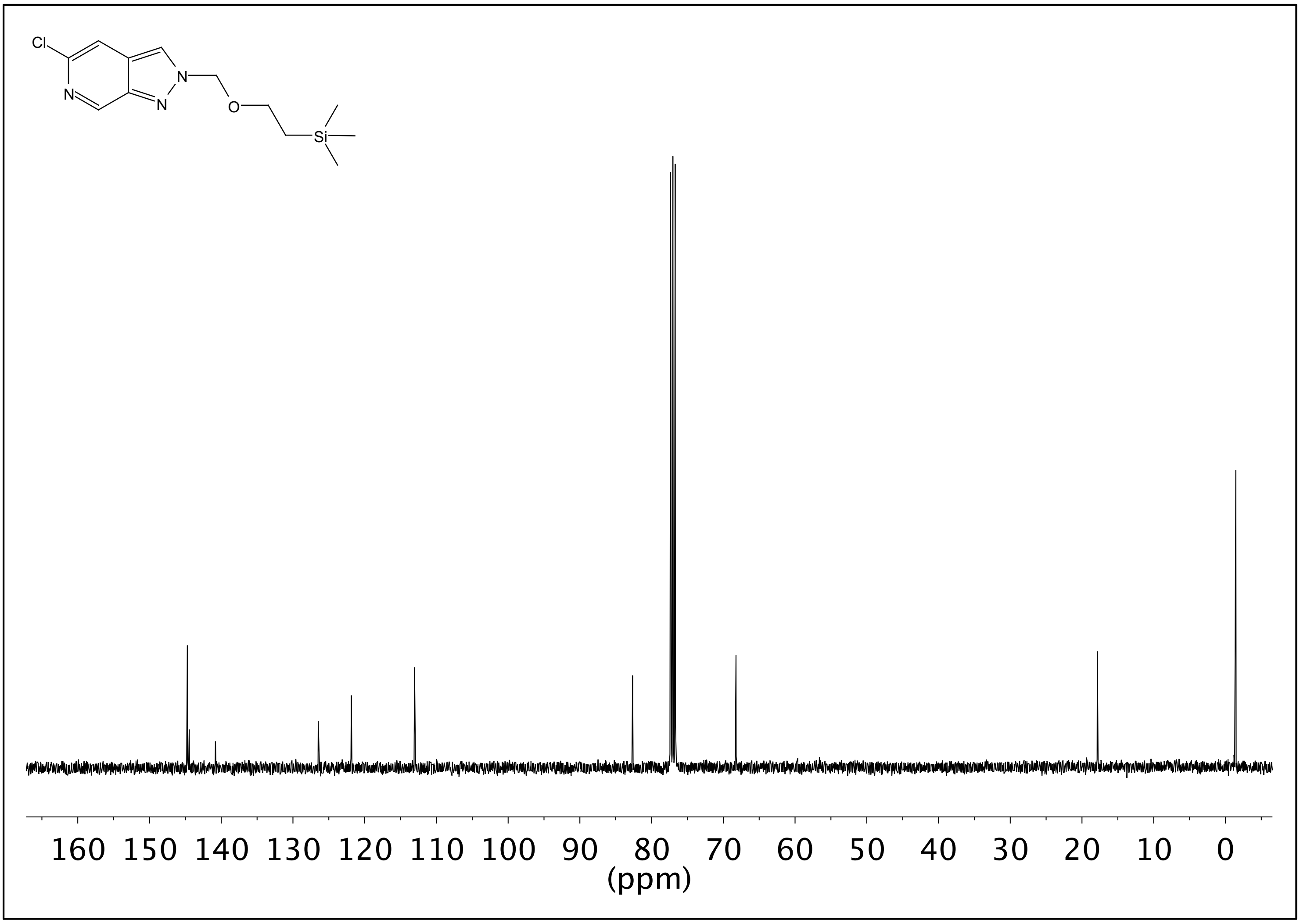

^{1}H NMR (700 MHz, Chloroform-*d*) and ^{13}C NMR (176 MHz, Chloroform-*d*) of compound **10a**

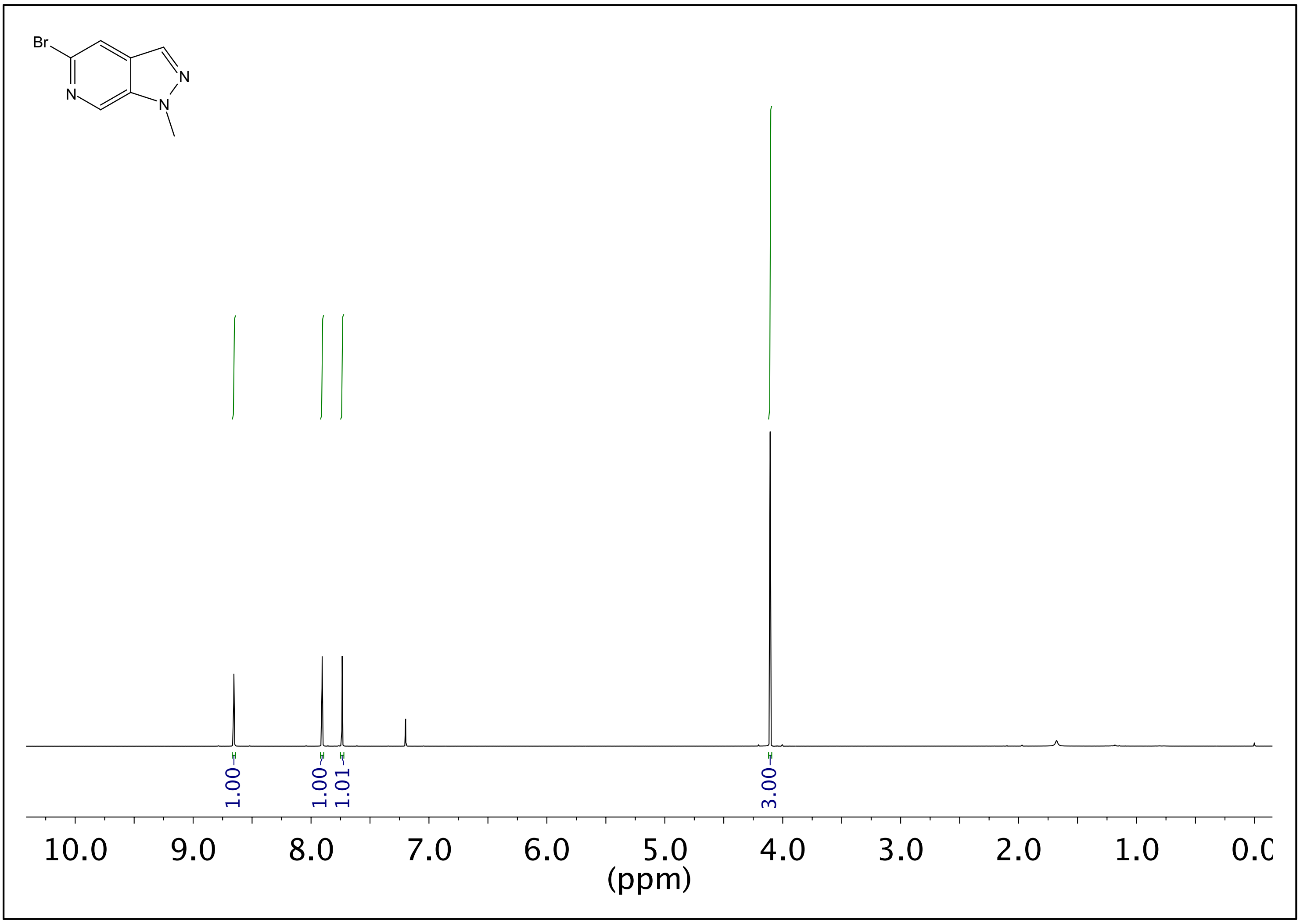

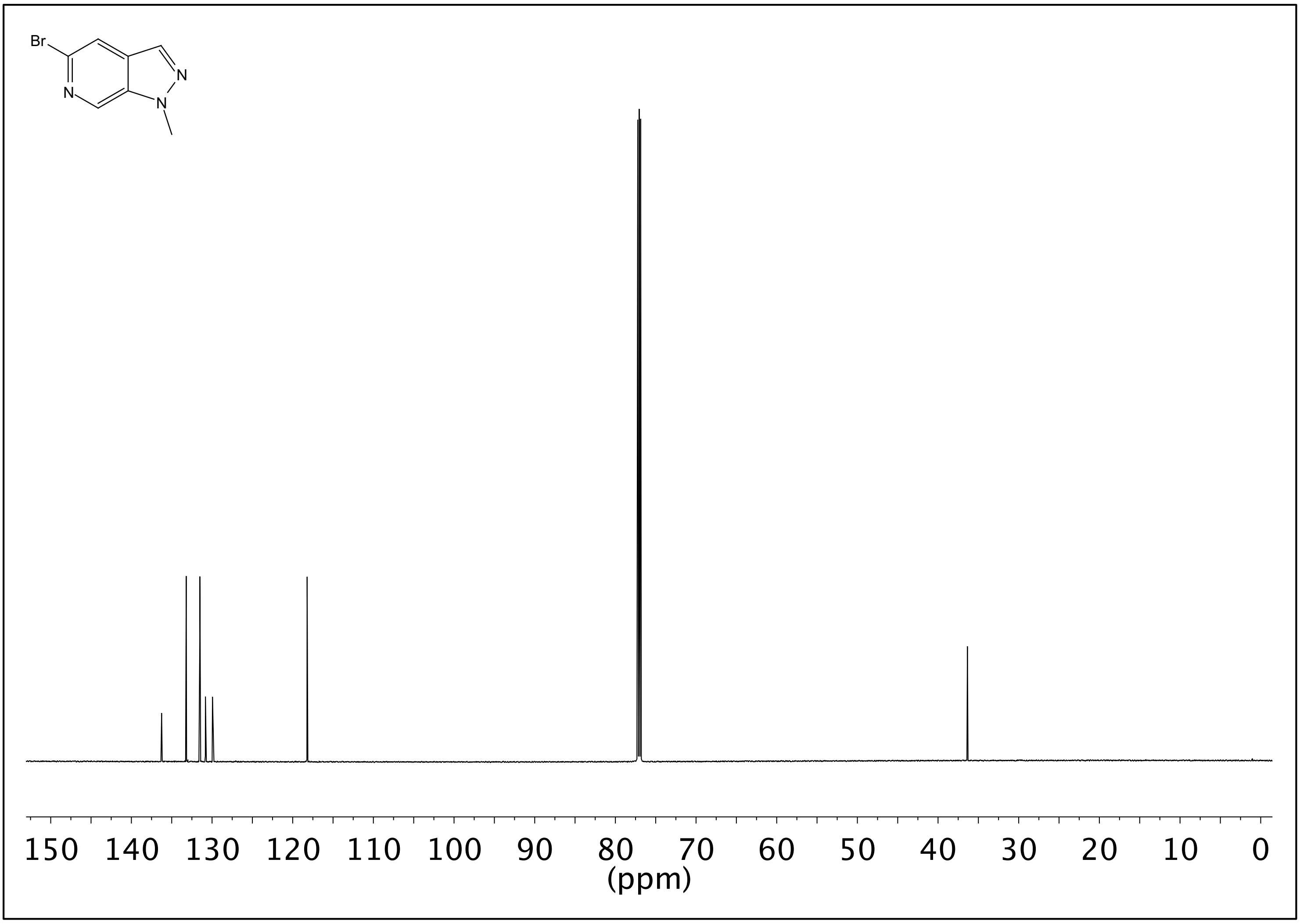

^{1}H NMR (700 MHz, Chloroform-*d*) and ^{13}C NMR (176 MHz, Chloroform-*d*) of compound **10b**

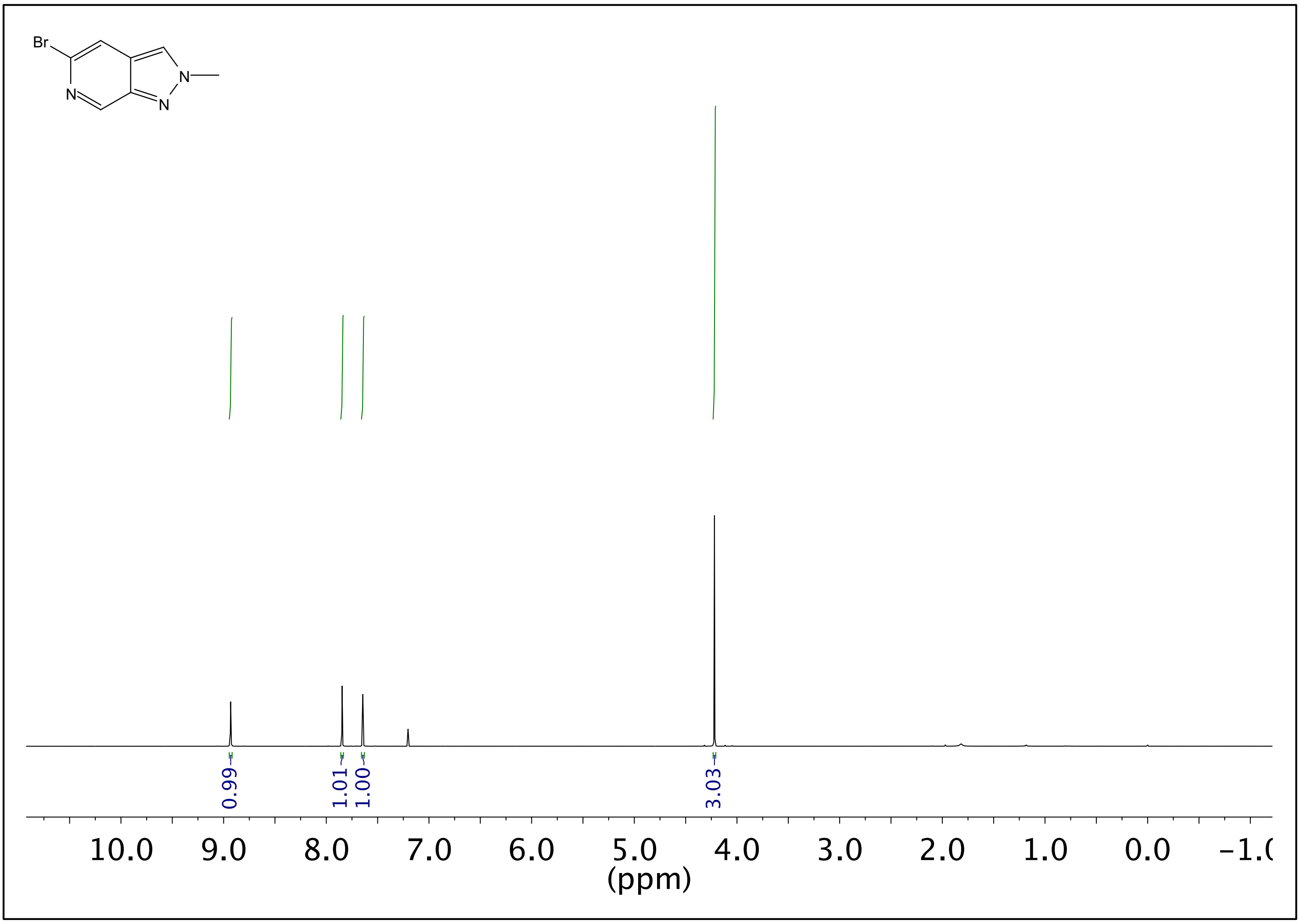

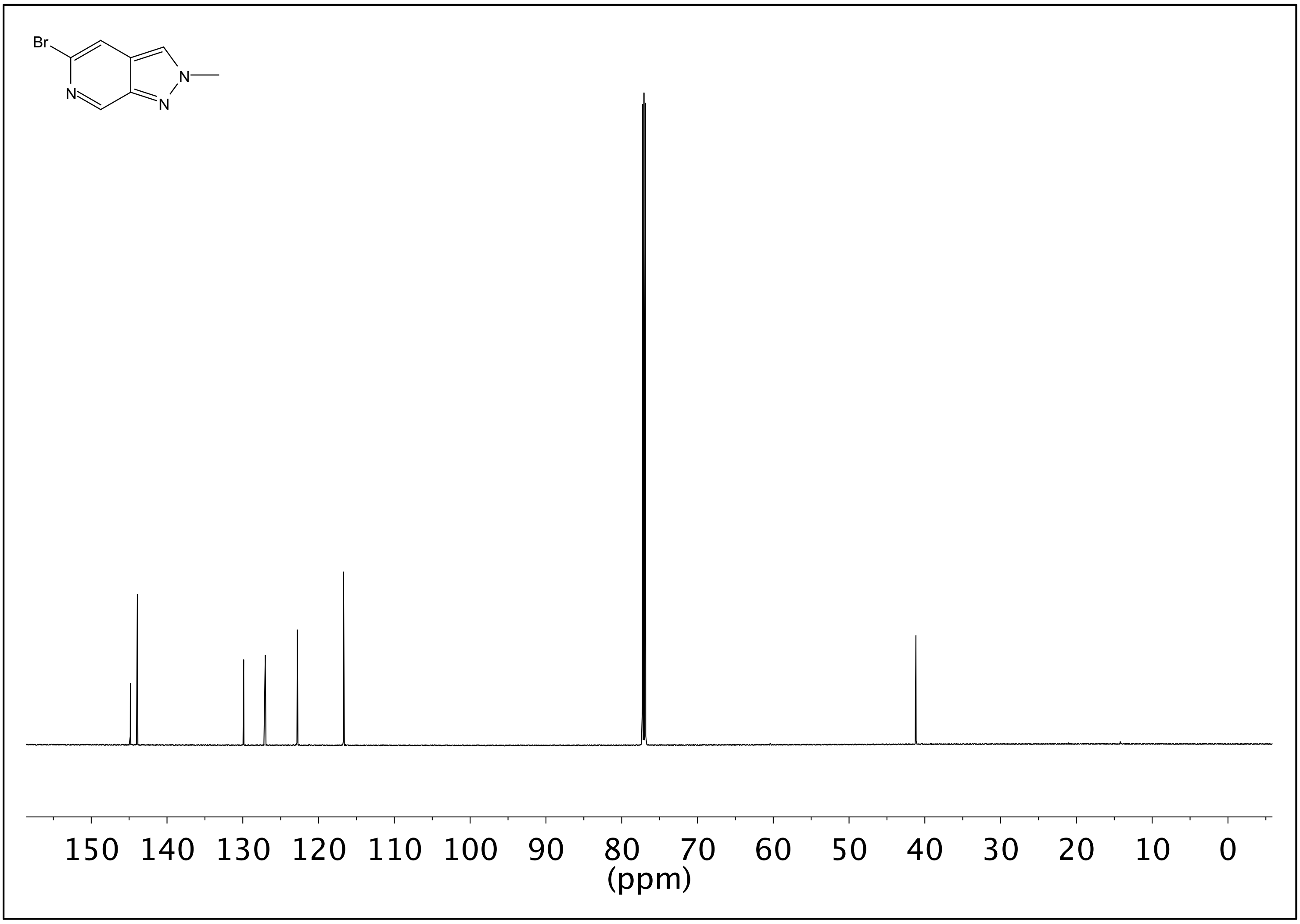

^{1}H NMR (600 MHz, Chloroform-*d*) and ^{13}C NMR (151 MHz, Chloroform-*d*) of compound **11a**

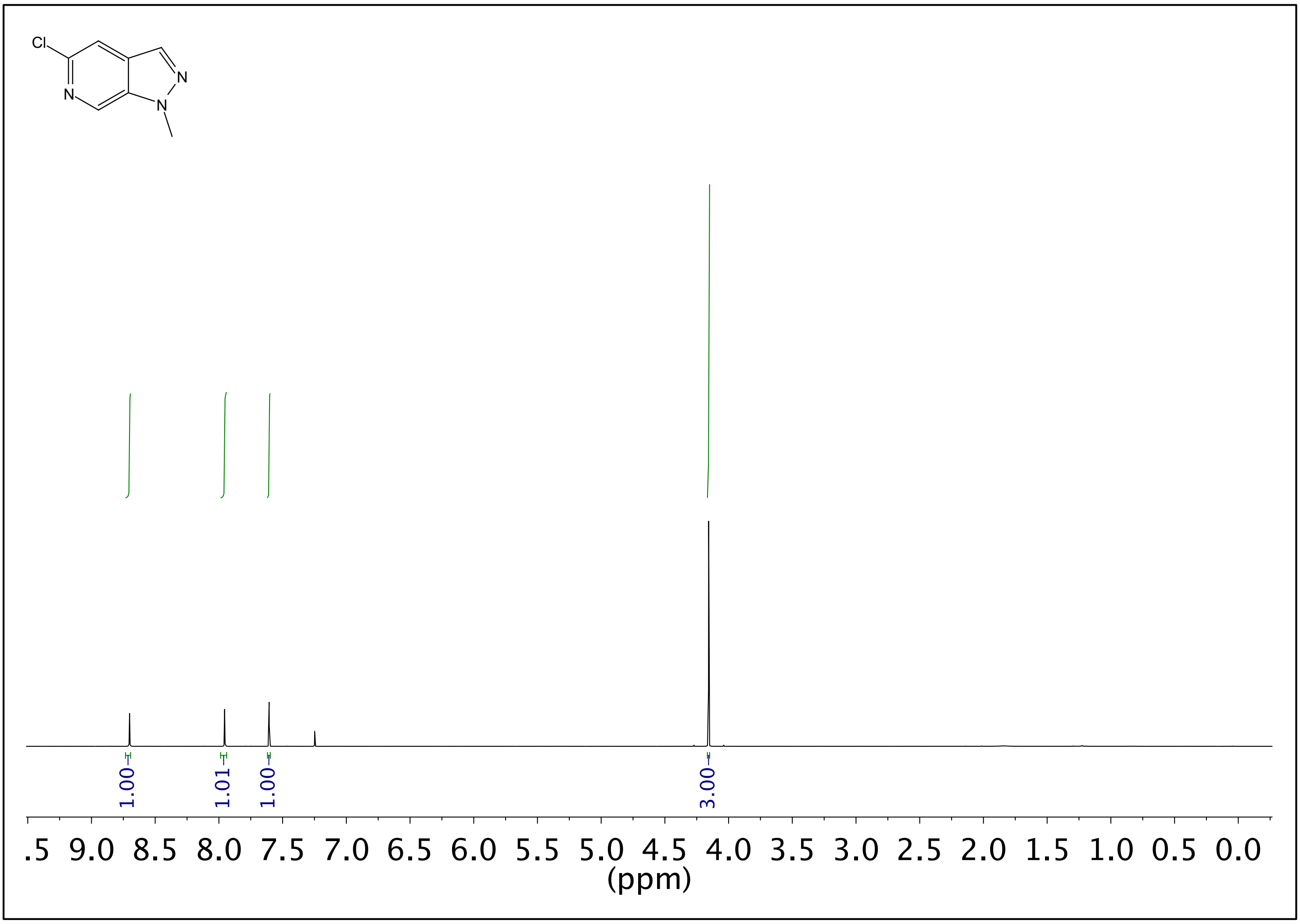

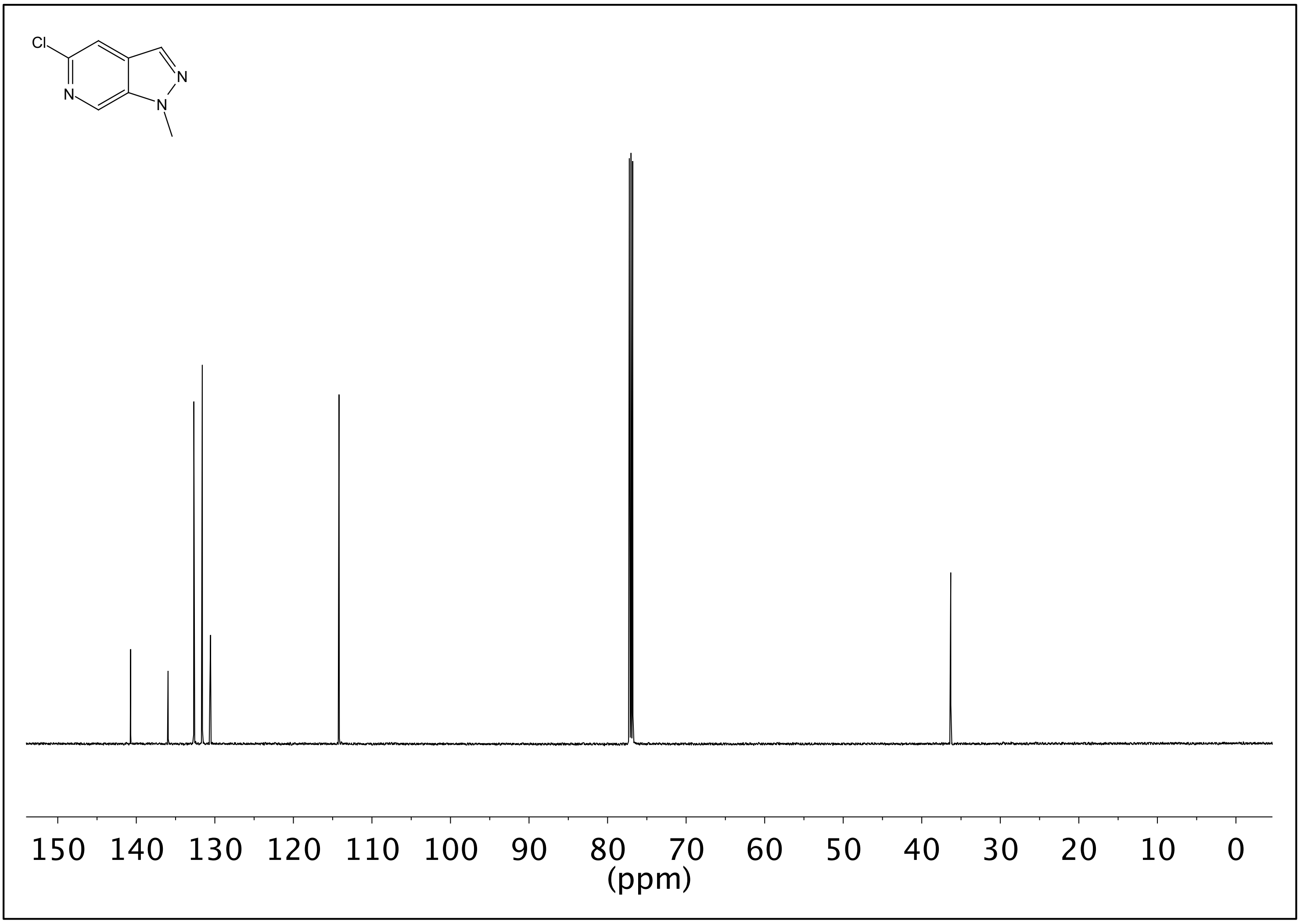

^{1}H NMR (600 MHz, Chloroform-*d*) and ^{13}C NMR (151 MHz, Chloroform-*d*) of compound **11b**

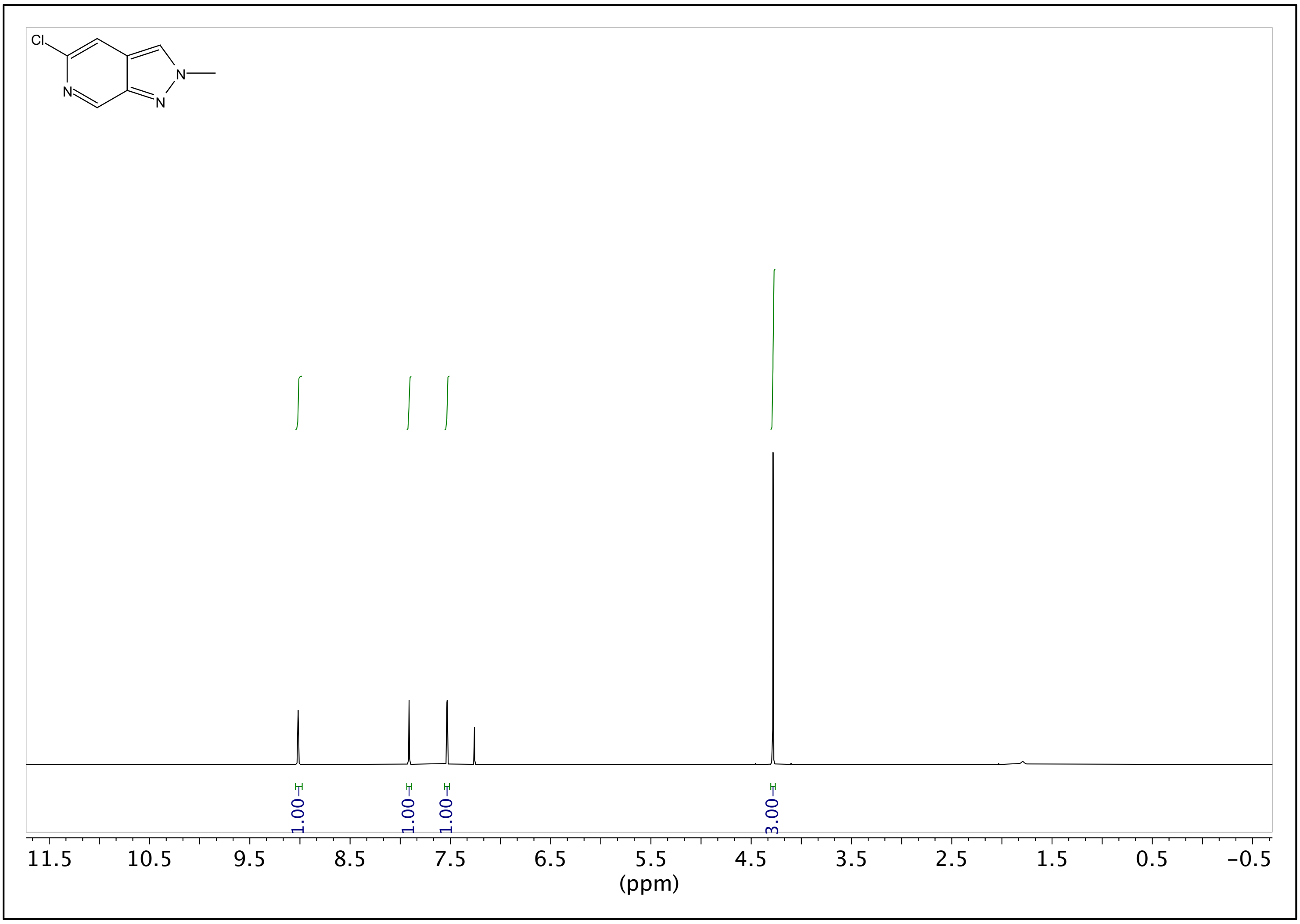

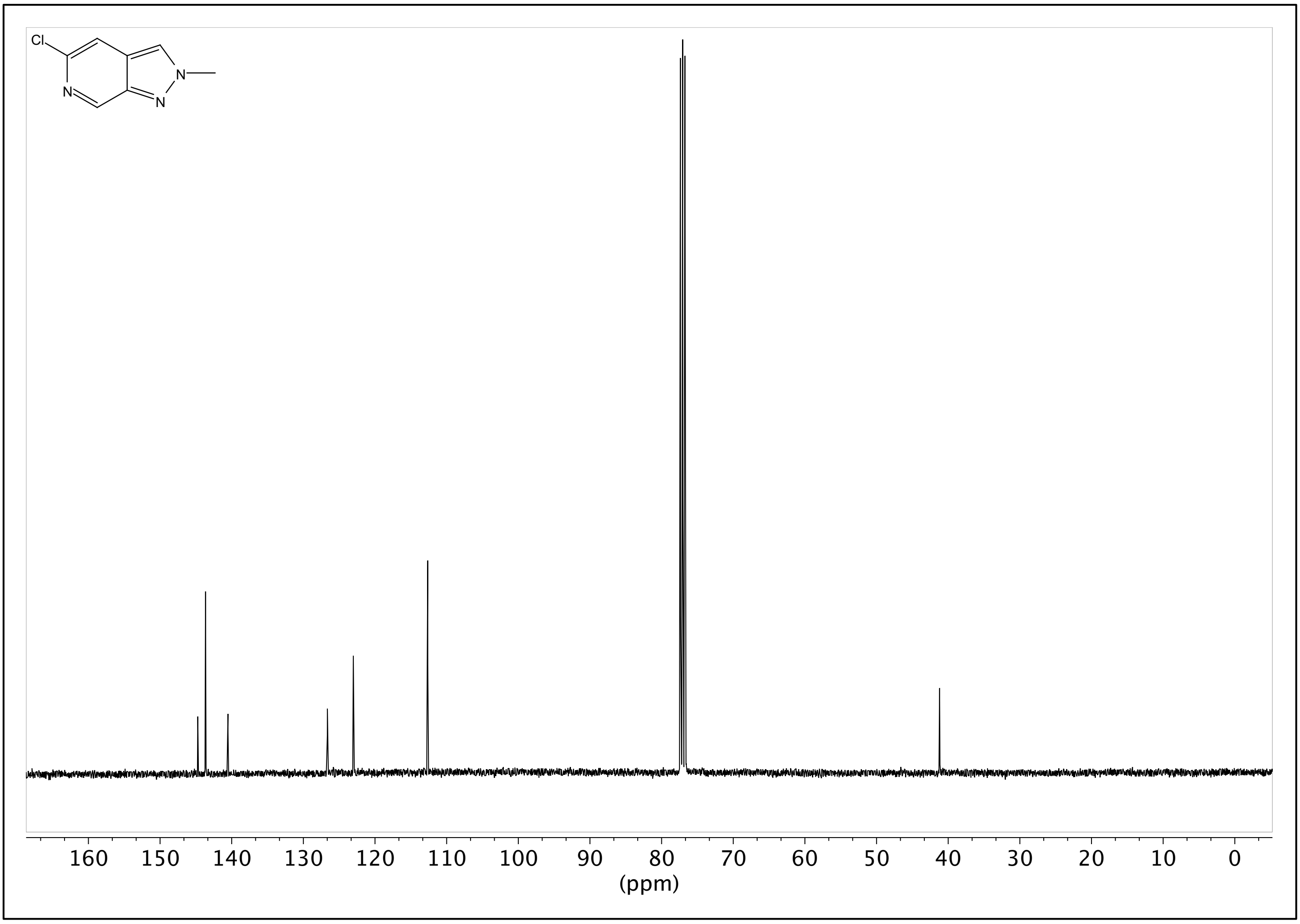

^{1}H NMR (400 MHz, Chloroform-*d*) and ^{13}C NMR (101 MHz, Chloroform-*d*) of compound **12a**

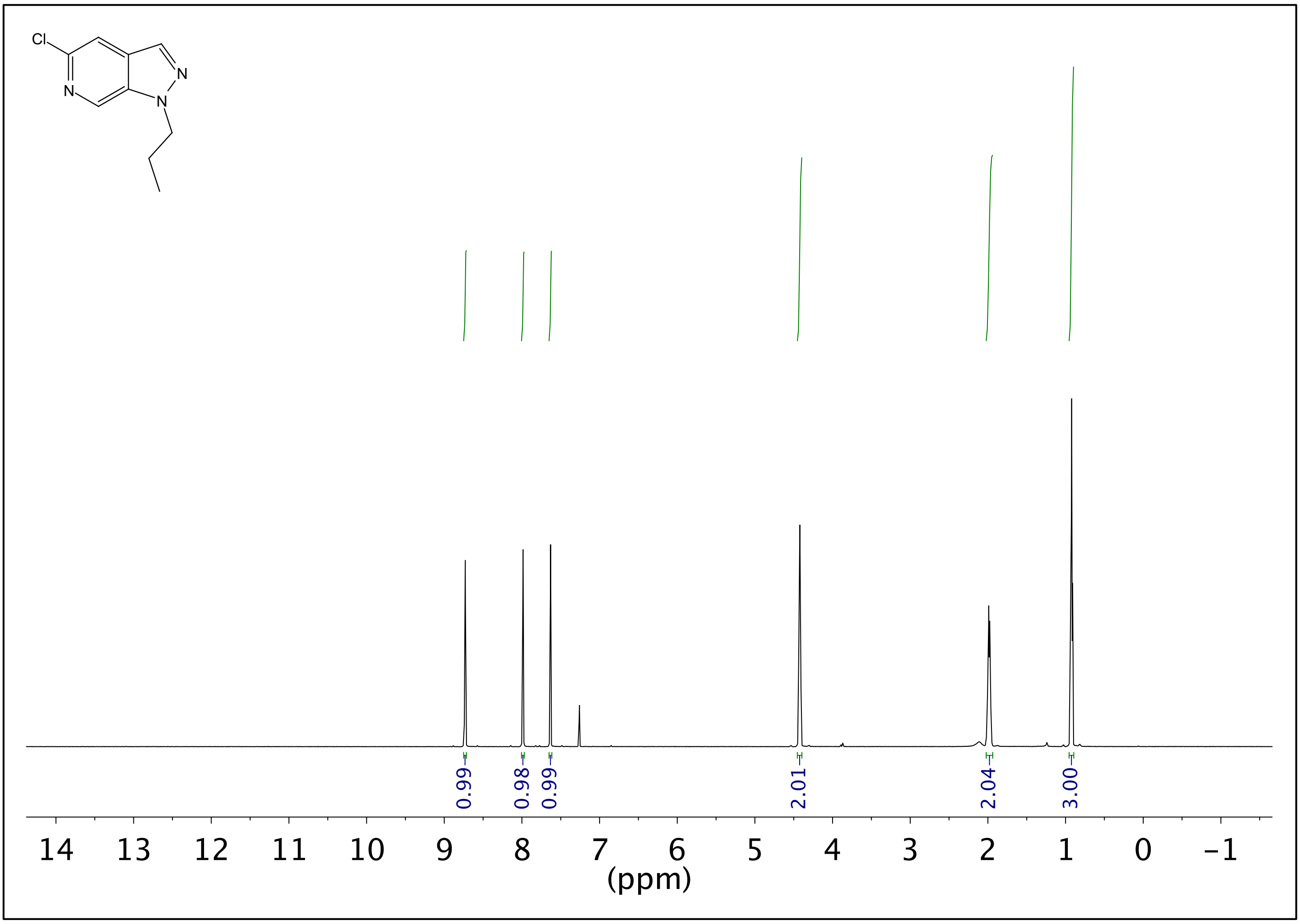

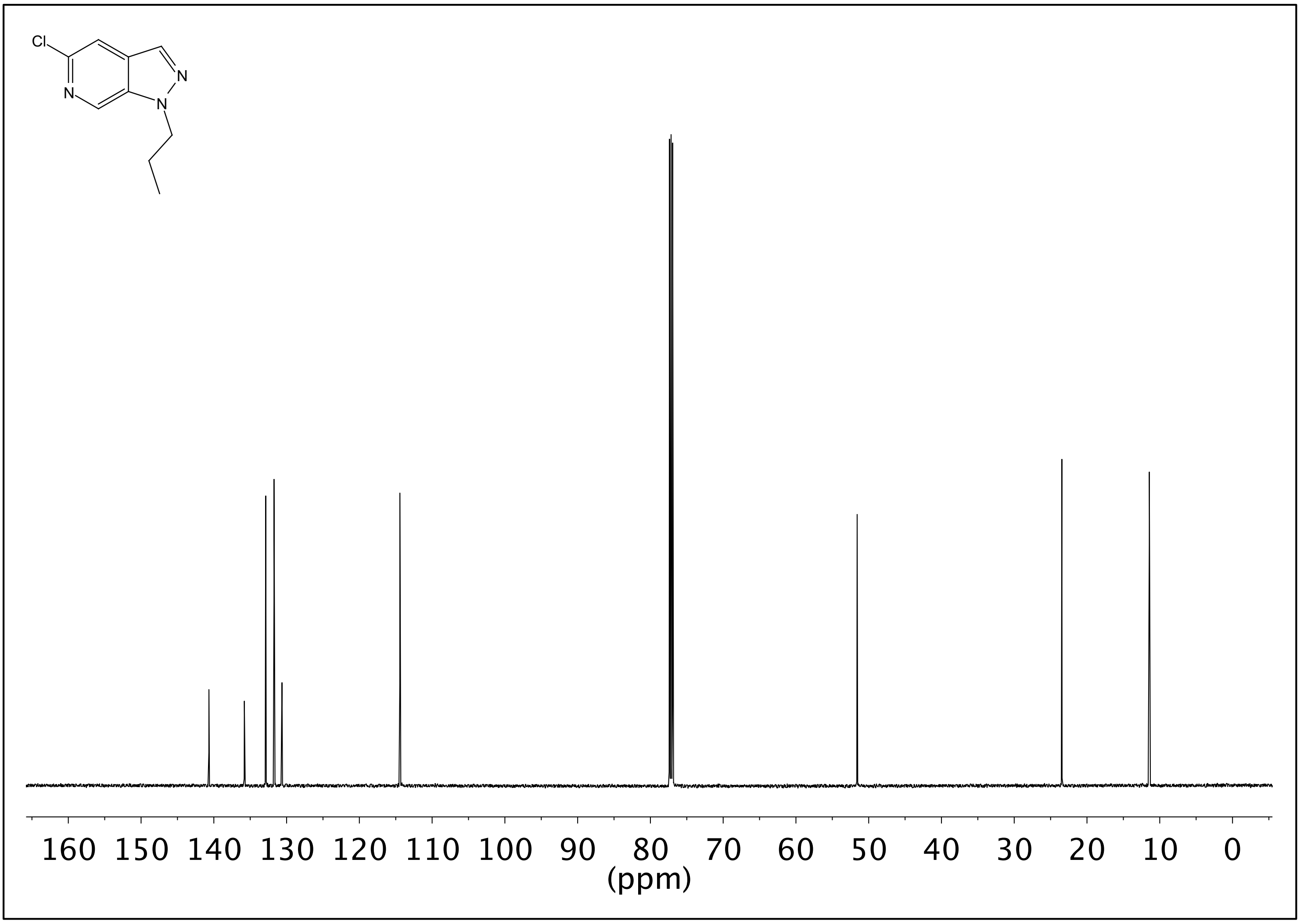

^1H NMR (400 MHz, Chloroform-*d*) and ^{13}C NMR (101 MHz, Chloroform-*d*) of compound **12b**

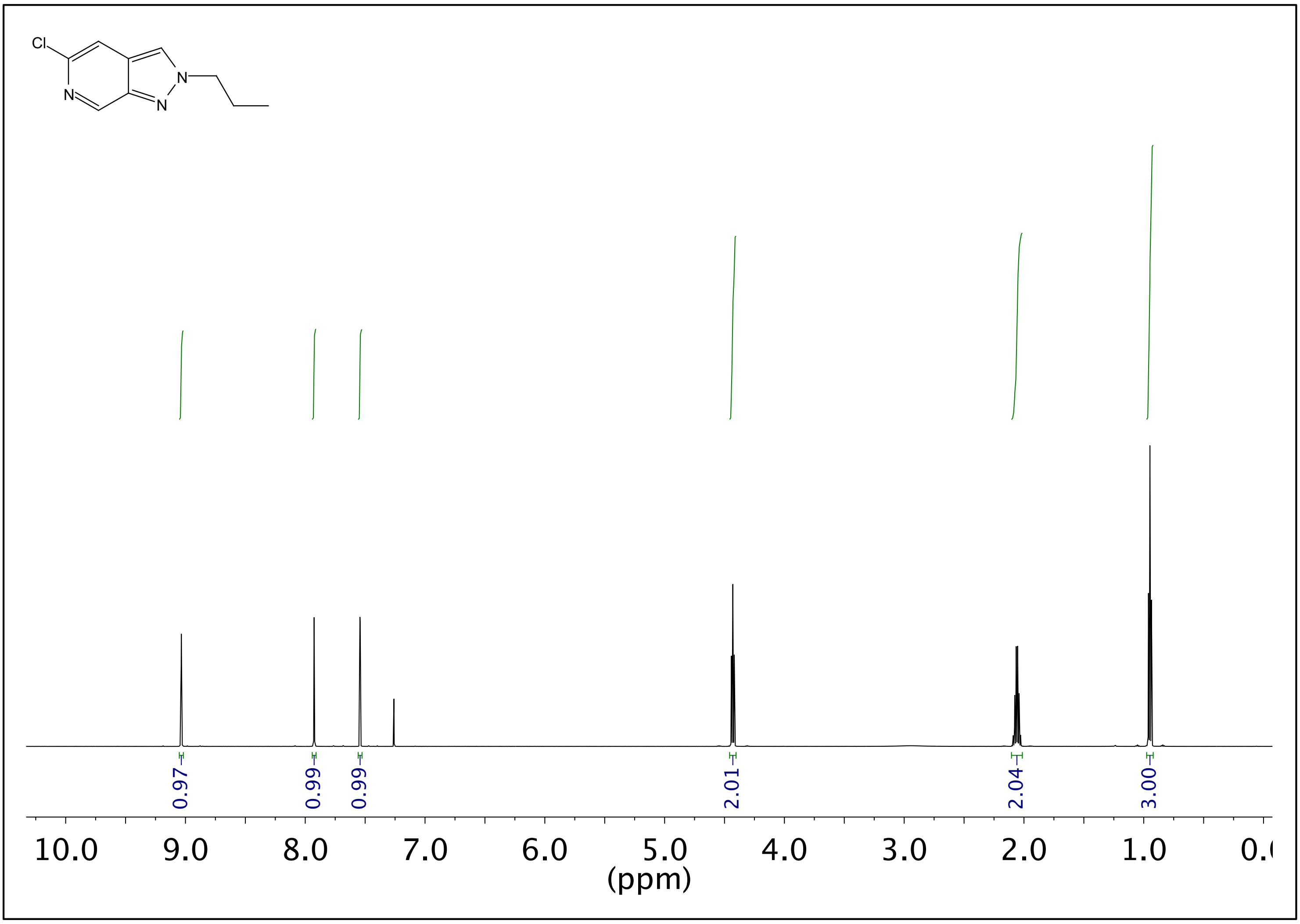

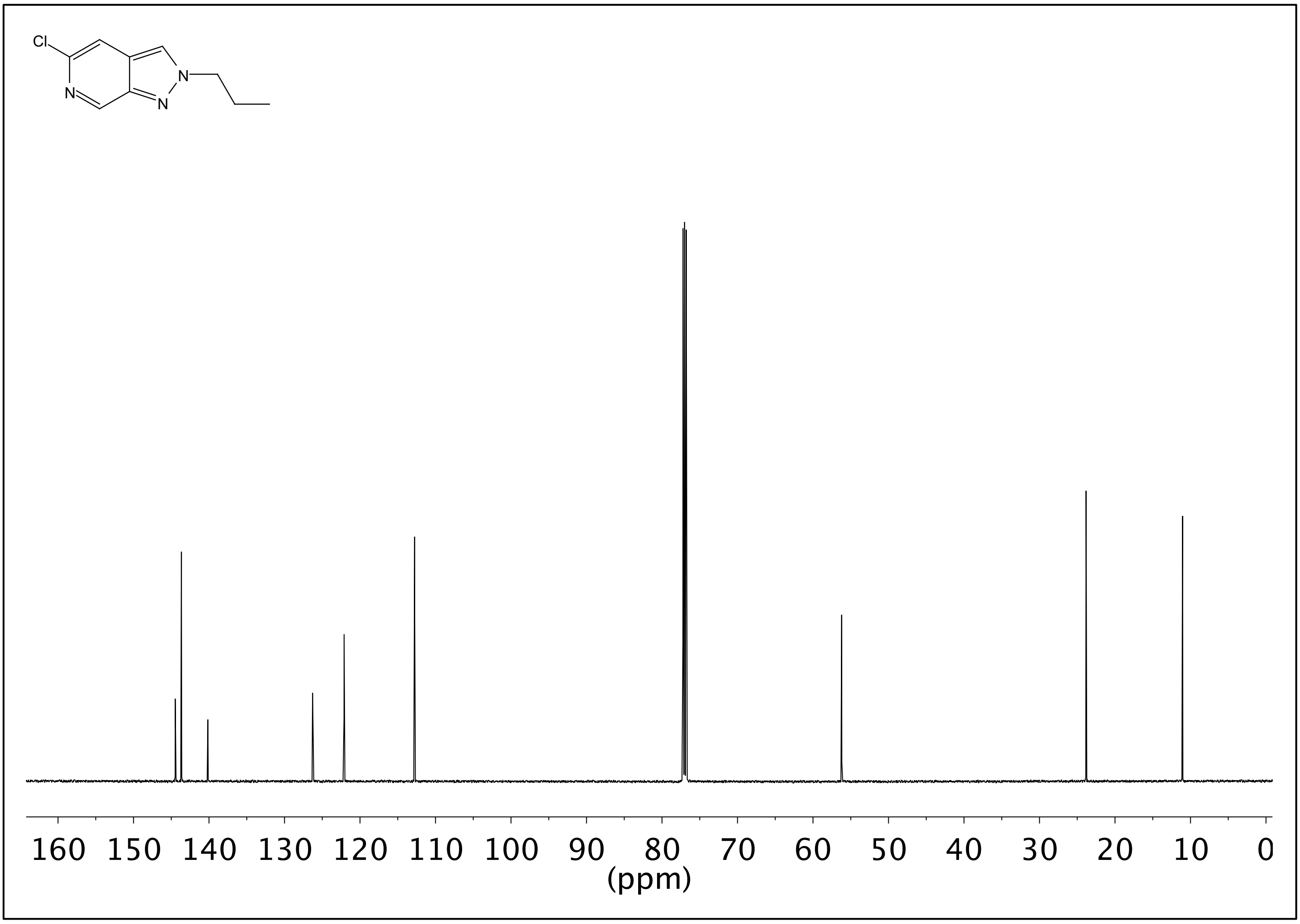

^{1}H NMR (600 MHz, Chloroform-*d*) and ^{13}C NMR (151 MHz, Chloroform-*d*) of compound **13**

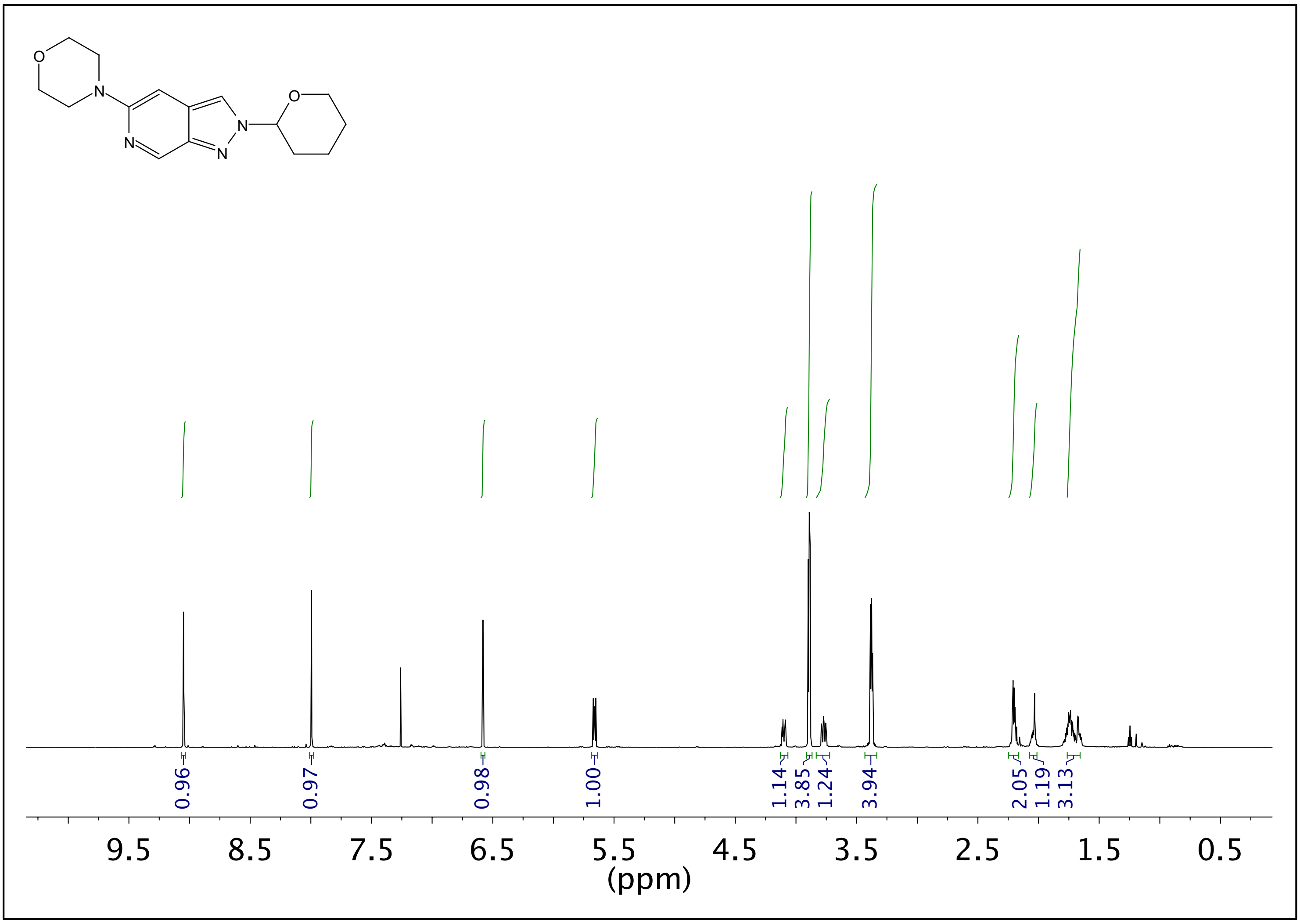

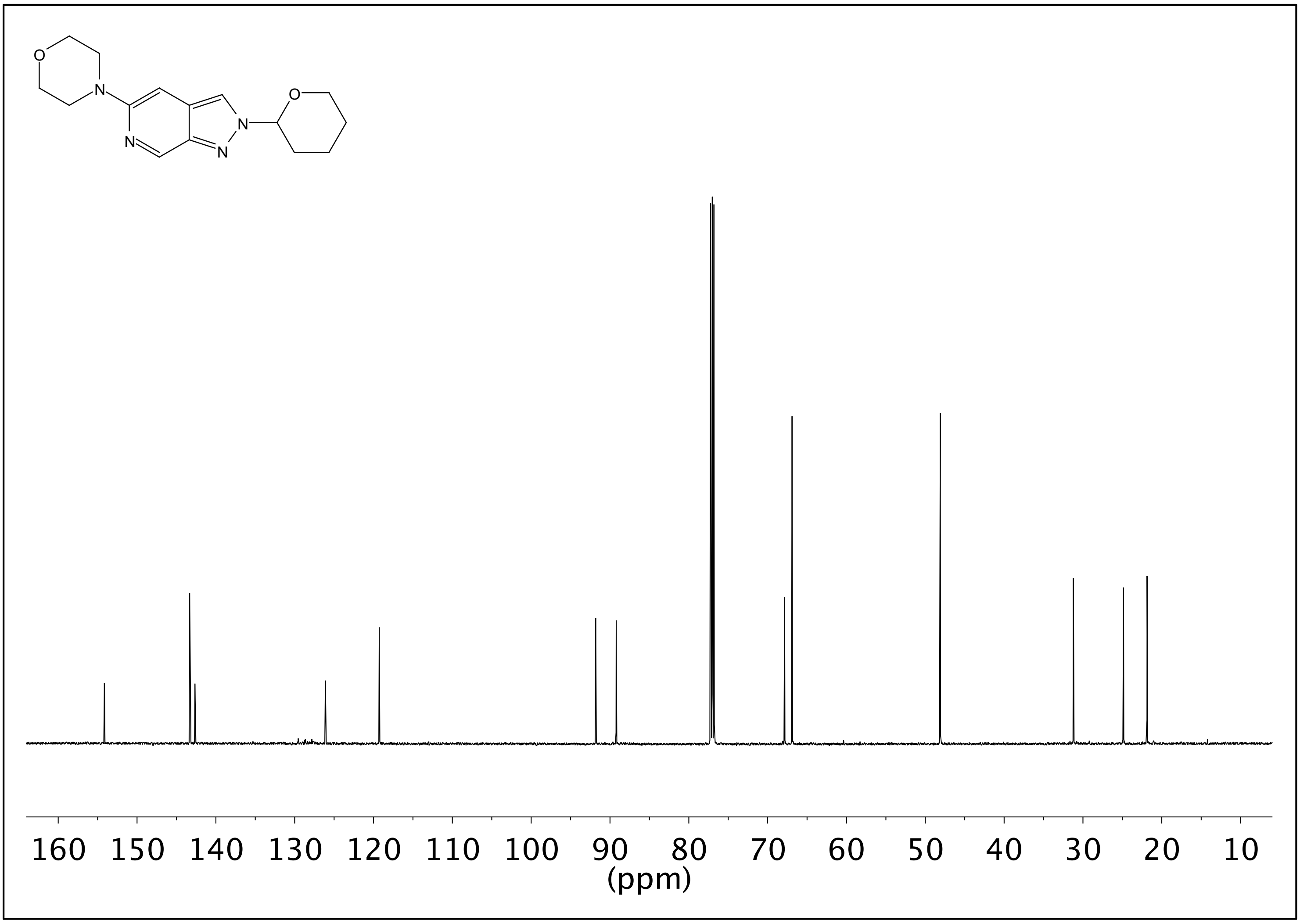

^{1}H NMR (600 MHz, Chloroform-*d*) and ^{13}C NMR (151 MHz, Chloroform-*d*) of compound **14**

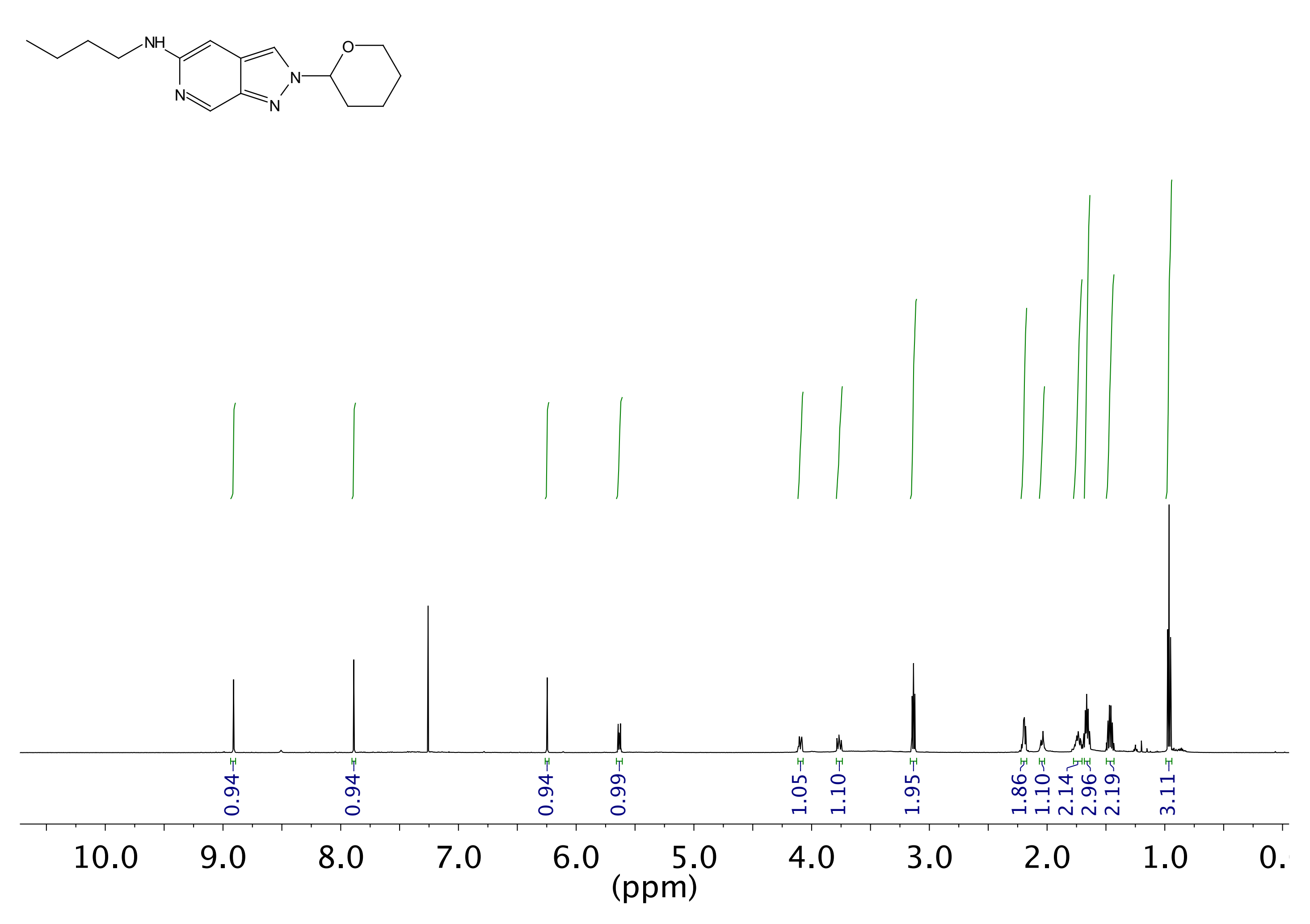

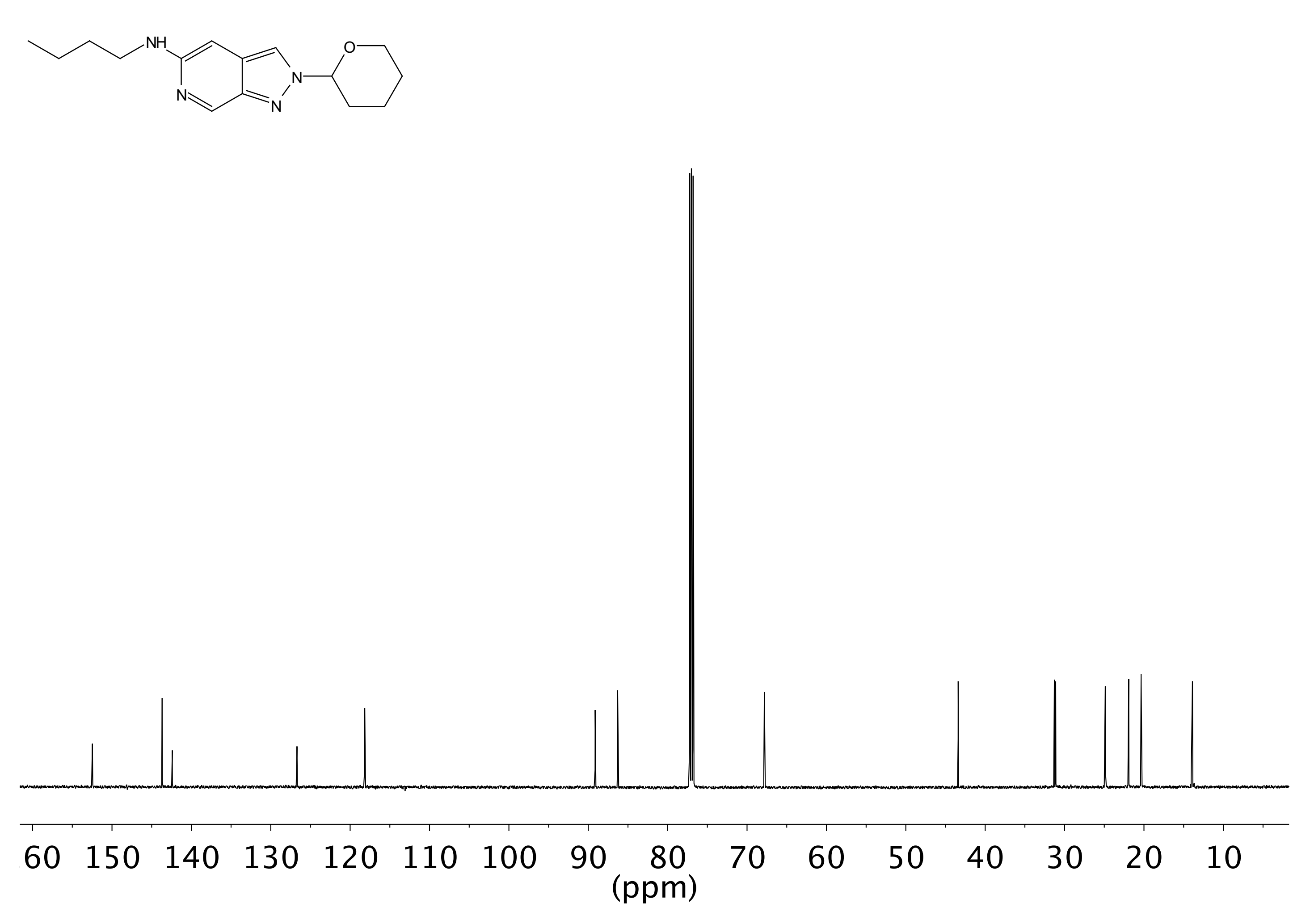

^{1}H NMR (600 MHz, Chloroform-*d*) and ^{13}C NMR (151 MHz, Chloroform-*d*) of compound **15**

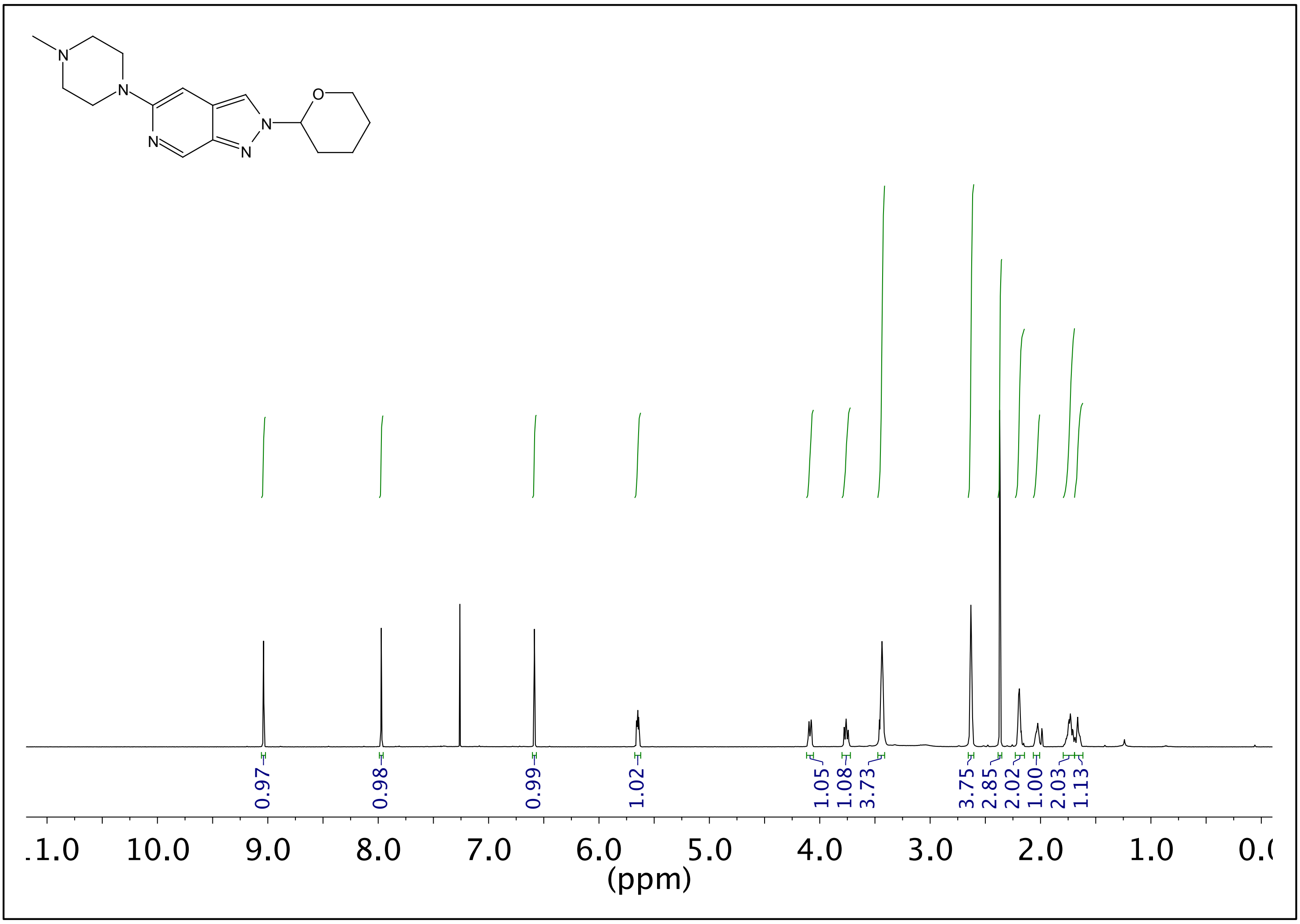

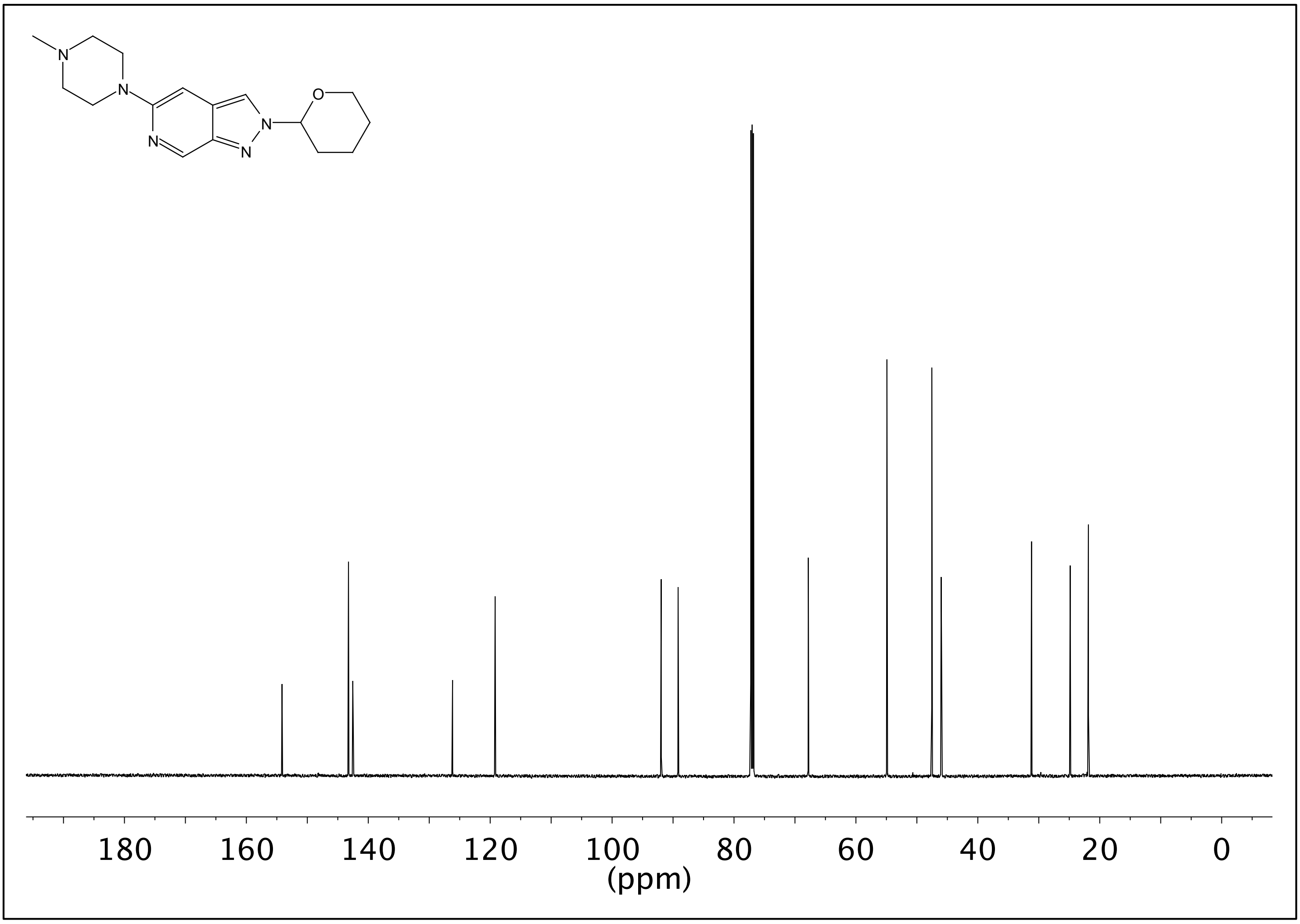

^{1}H NMR (600 MHz, Chloroform-*d*) and ^{13}C NMR (151 MHz, Chloroform-*d*) of compound **16**

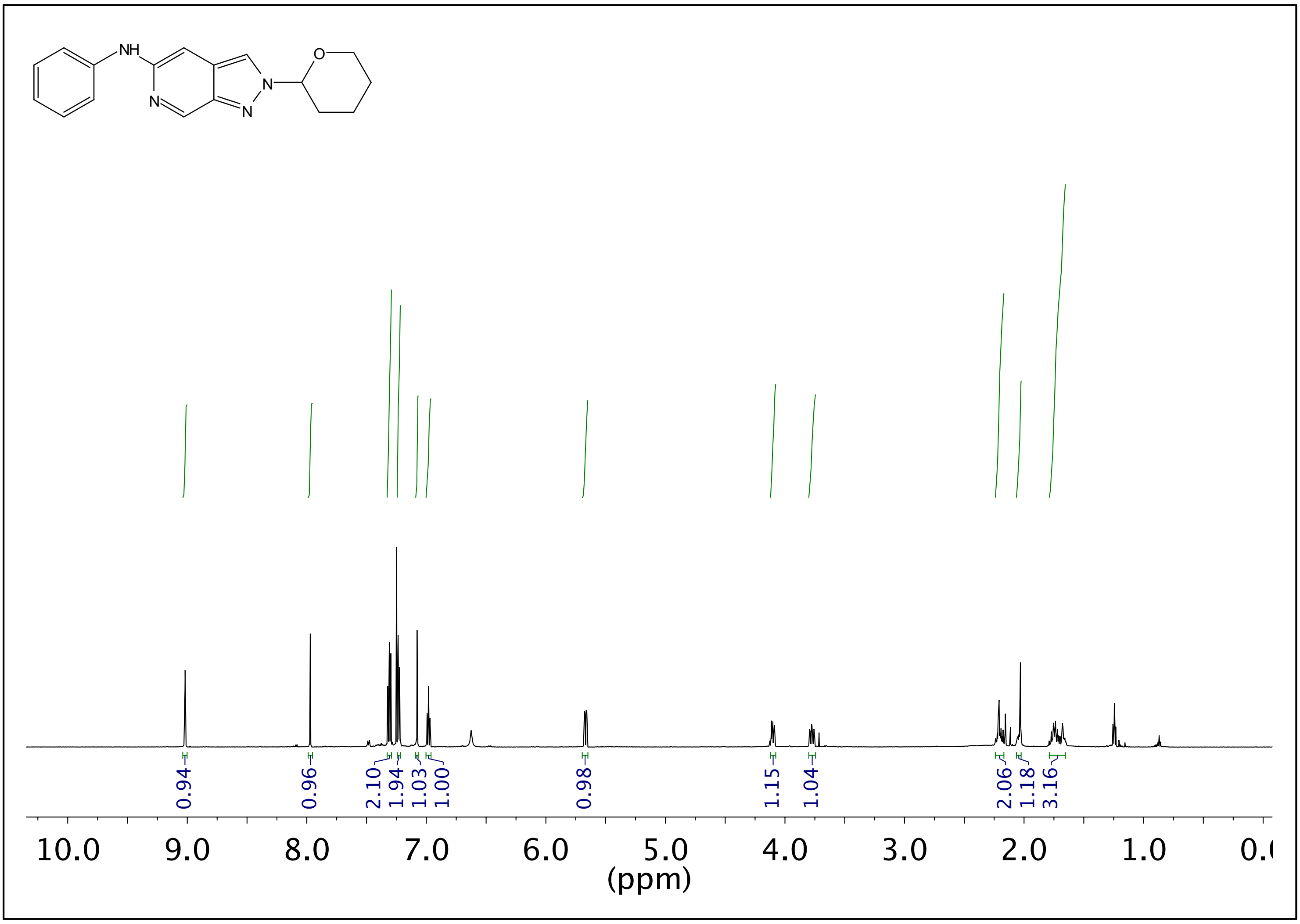

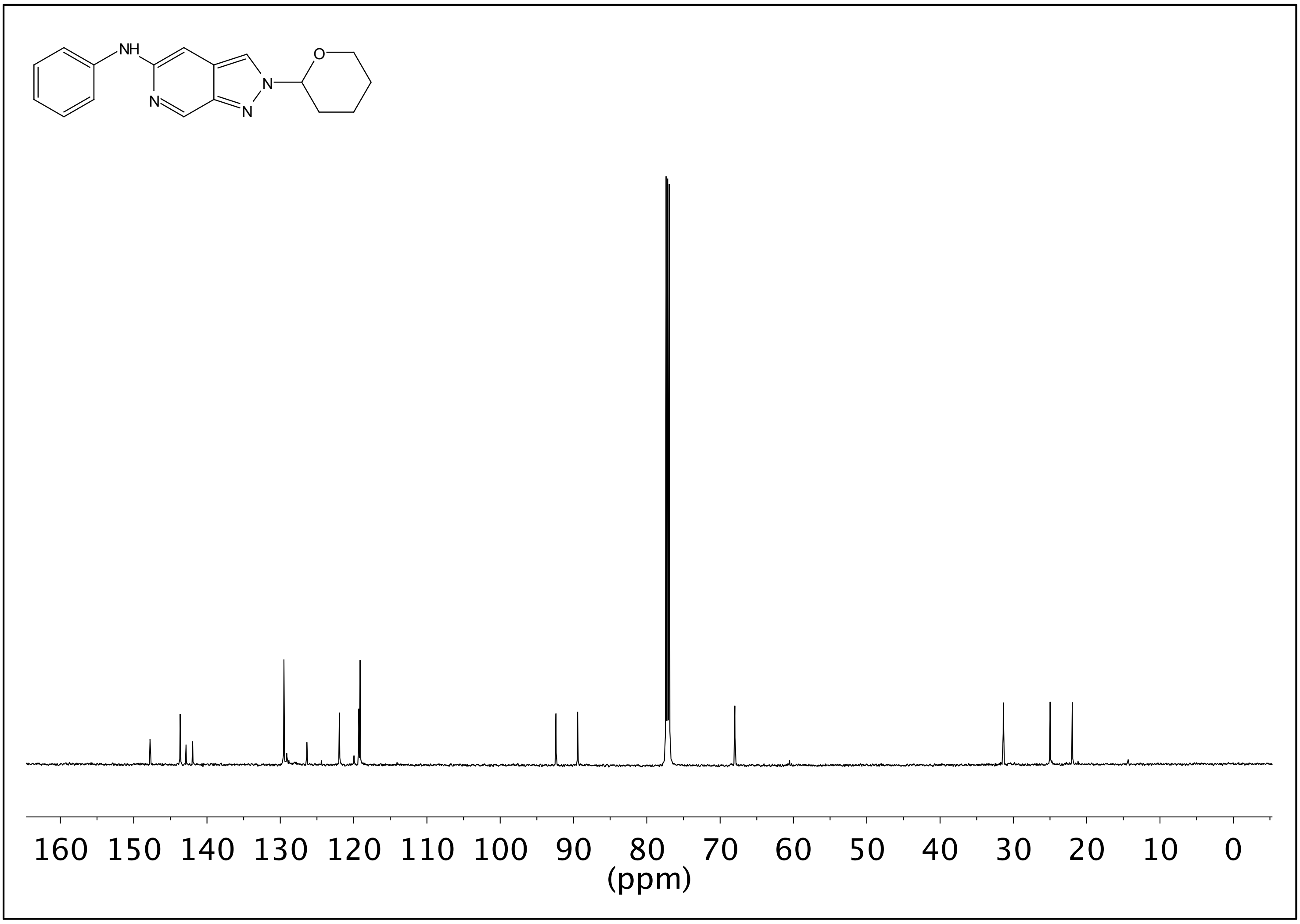

^{1}H NMR (400 MHz, Chloroform-*d*) and ^{13}C NMR (101 MHz, Chloroform-*d*) of compound **17**

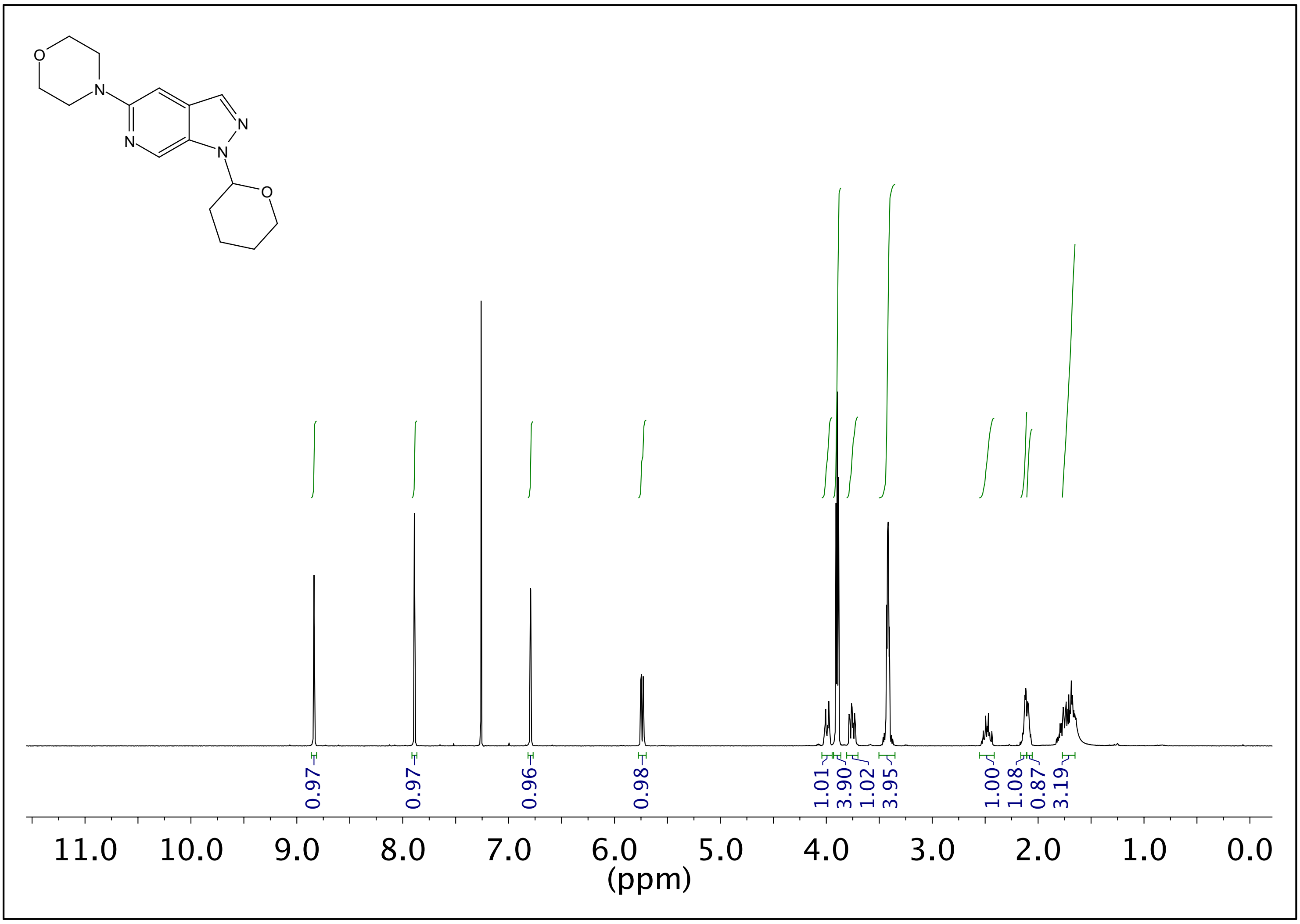

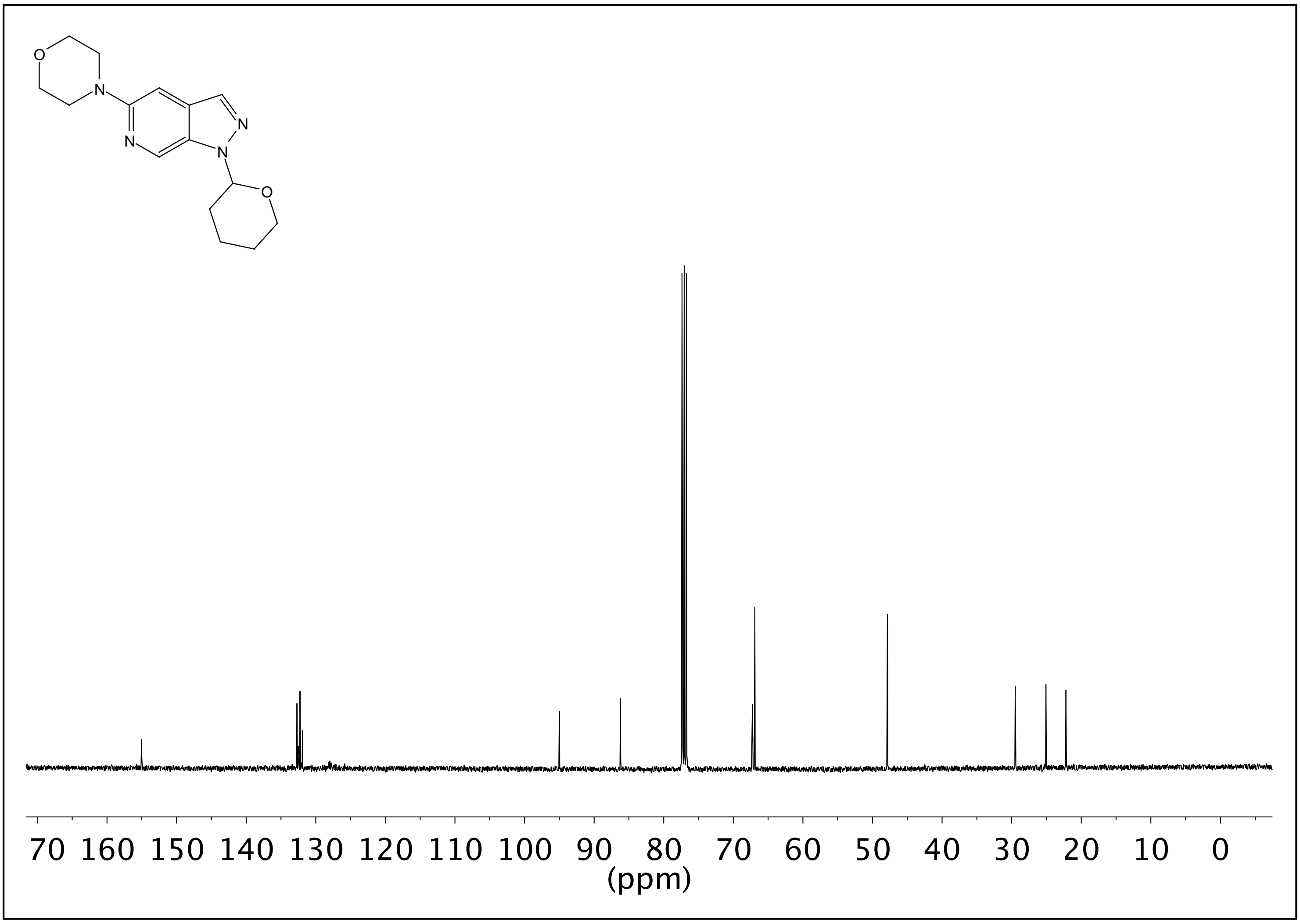

^{1}H NMR (600 MHz, Chloroform-*d*) and ^{13}C NMR (151 MHz, Chloroform-*d*) of compound **18**

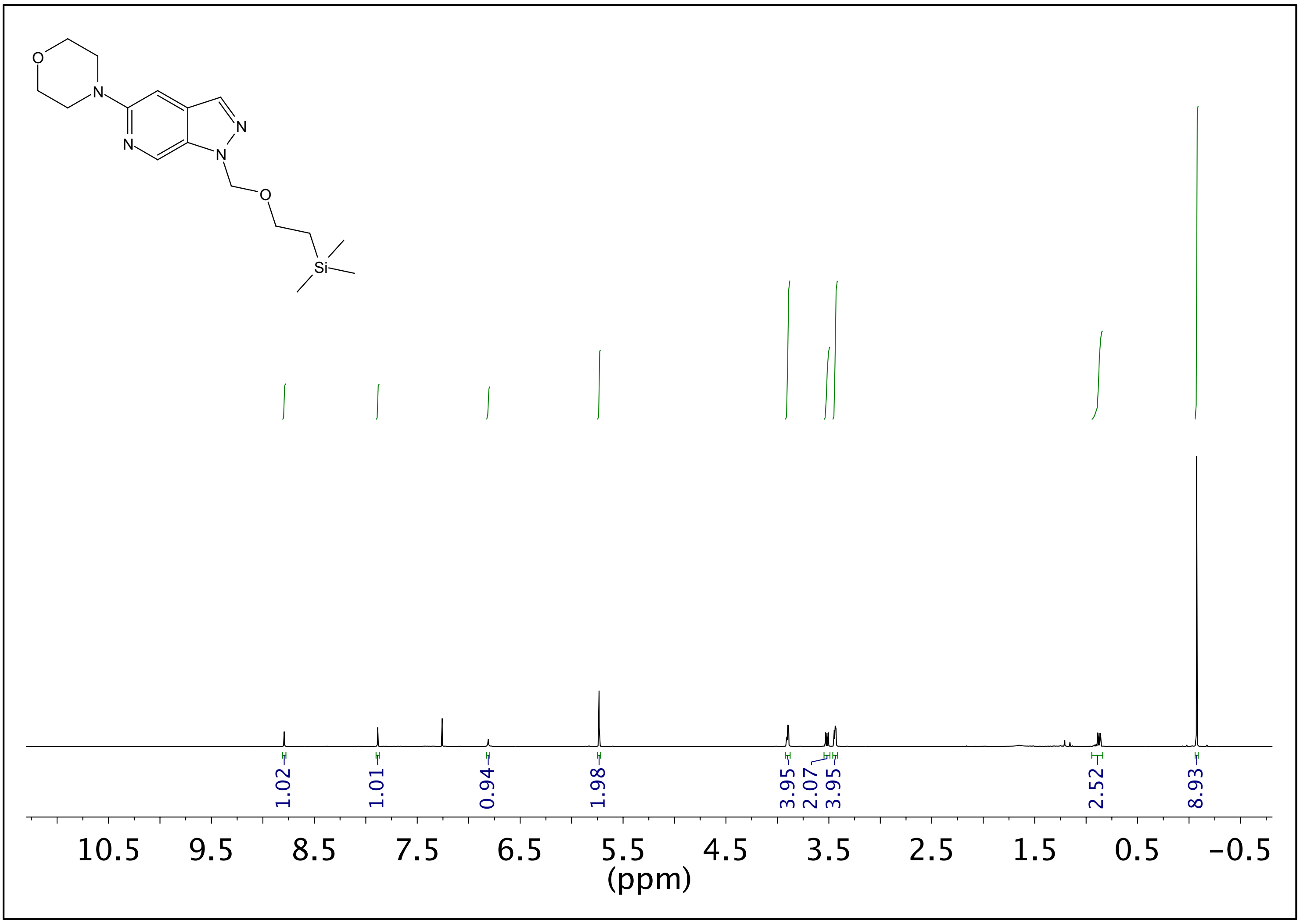

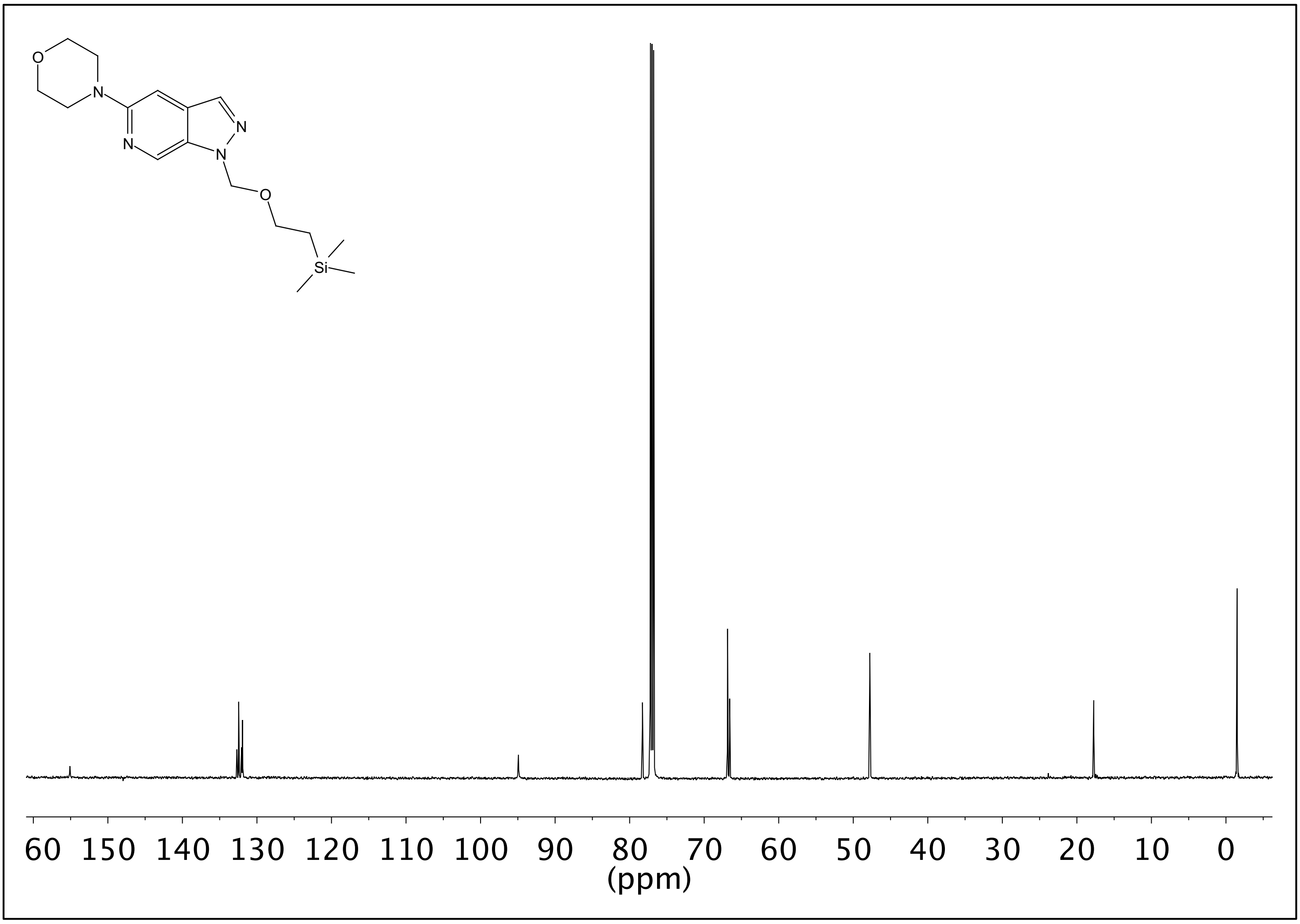

^{1}H NMR (600 MHz, Chloroform-*d*) and ^{13}C NMR (151 MHz, Chloroform-*d*) of compound **19**

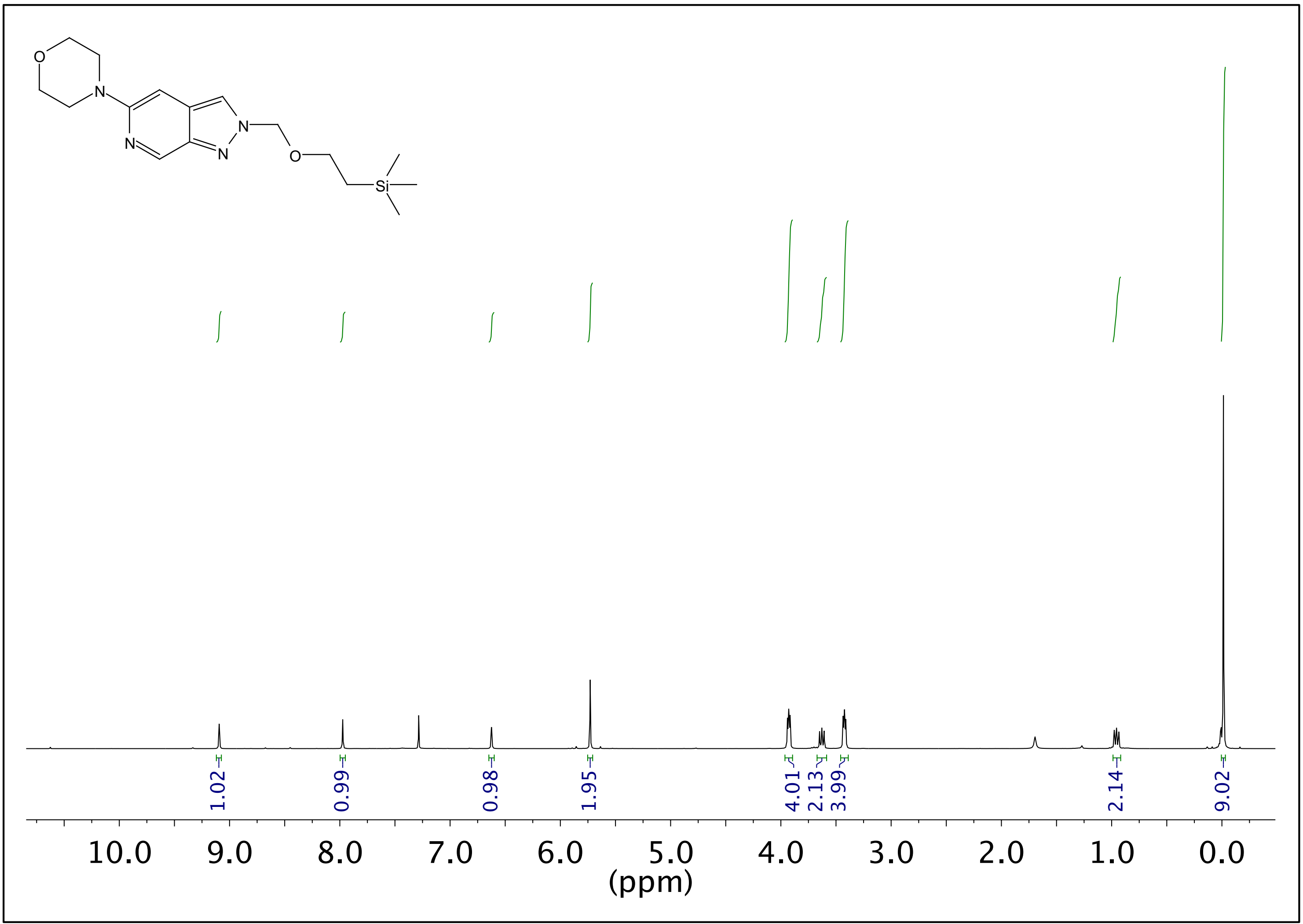

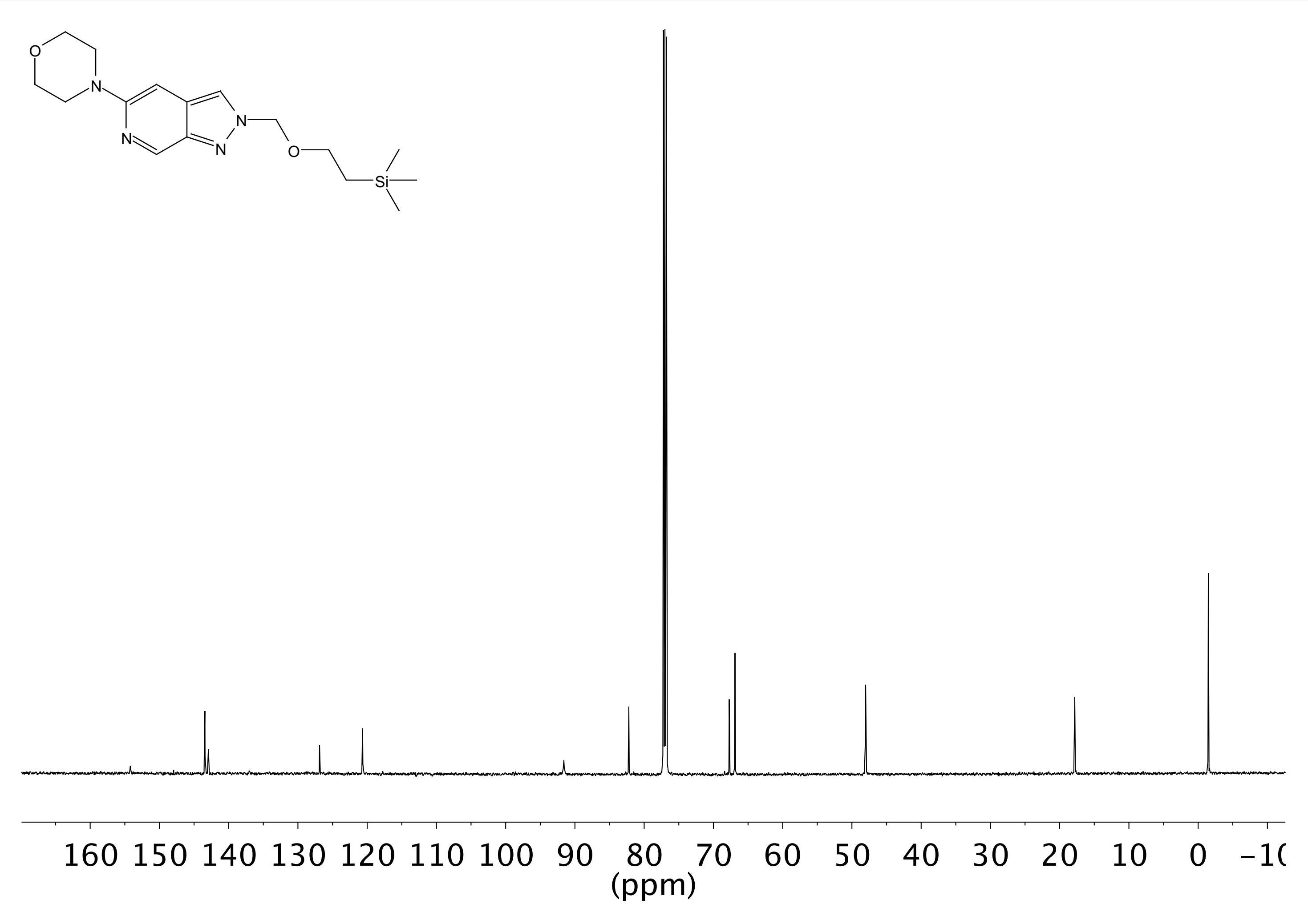

¹H NMR (600 MHz, Chloroform-*d*) and ¹³C NMR (151 MHz, Chloroform-*d*) of compound **20**

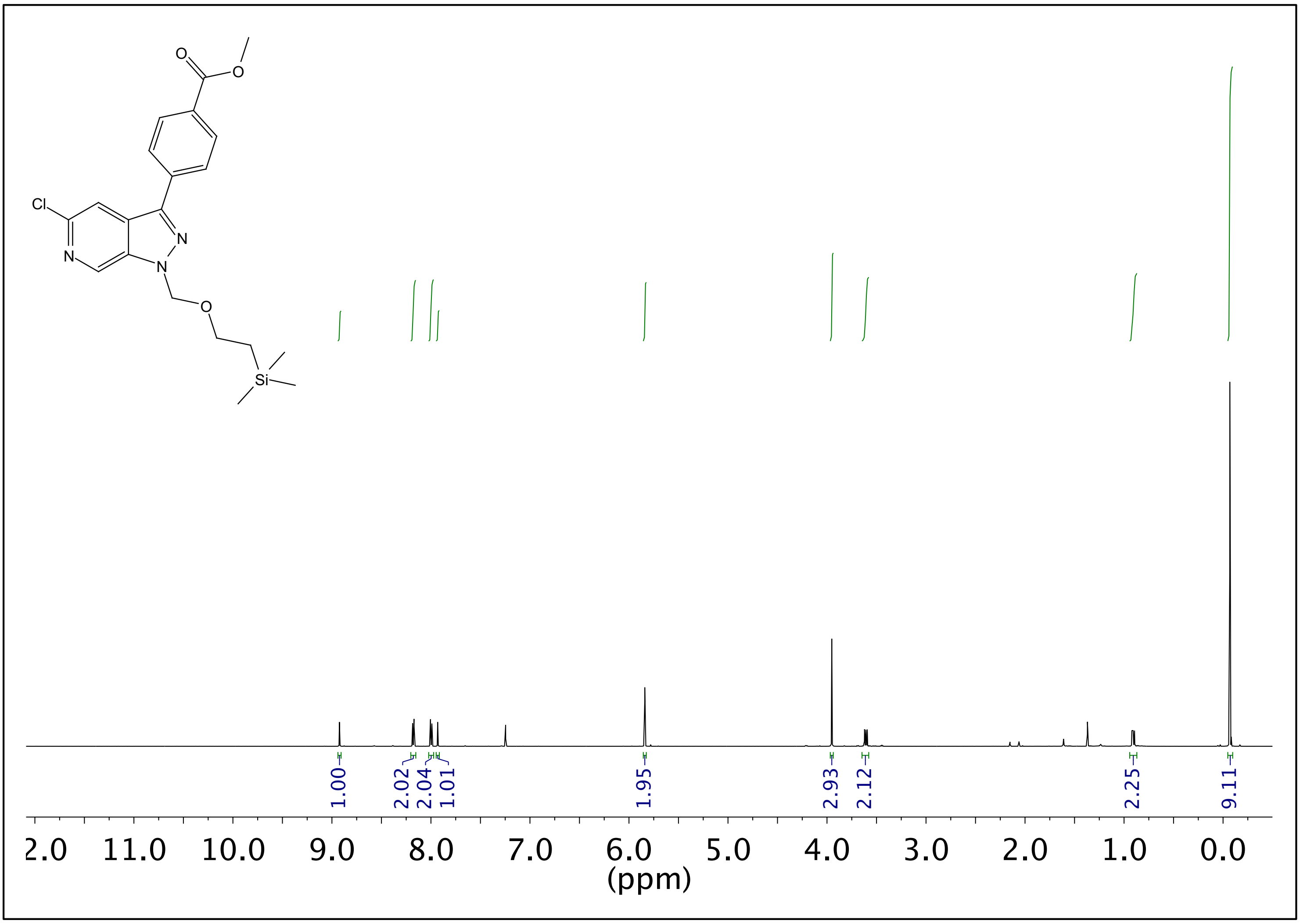

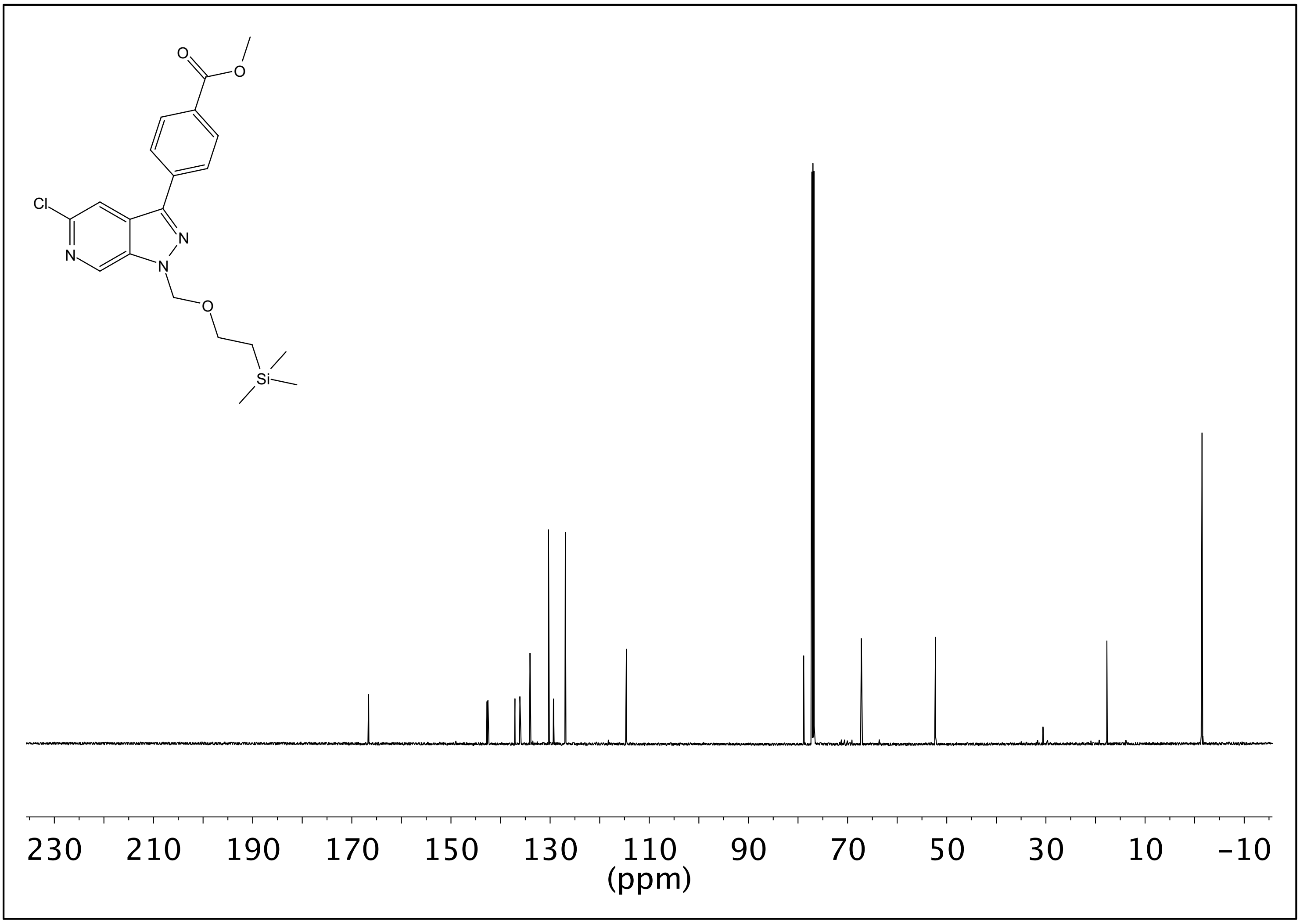

^{1}H NMR (700 MHz, Chloroform-*d*) and ^{13}C NMR (151 MHz, Chloroform-*d*) of compound **21**

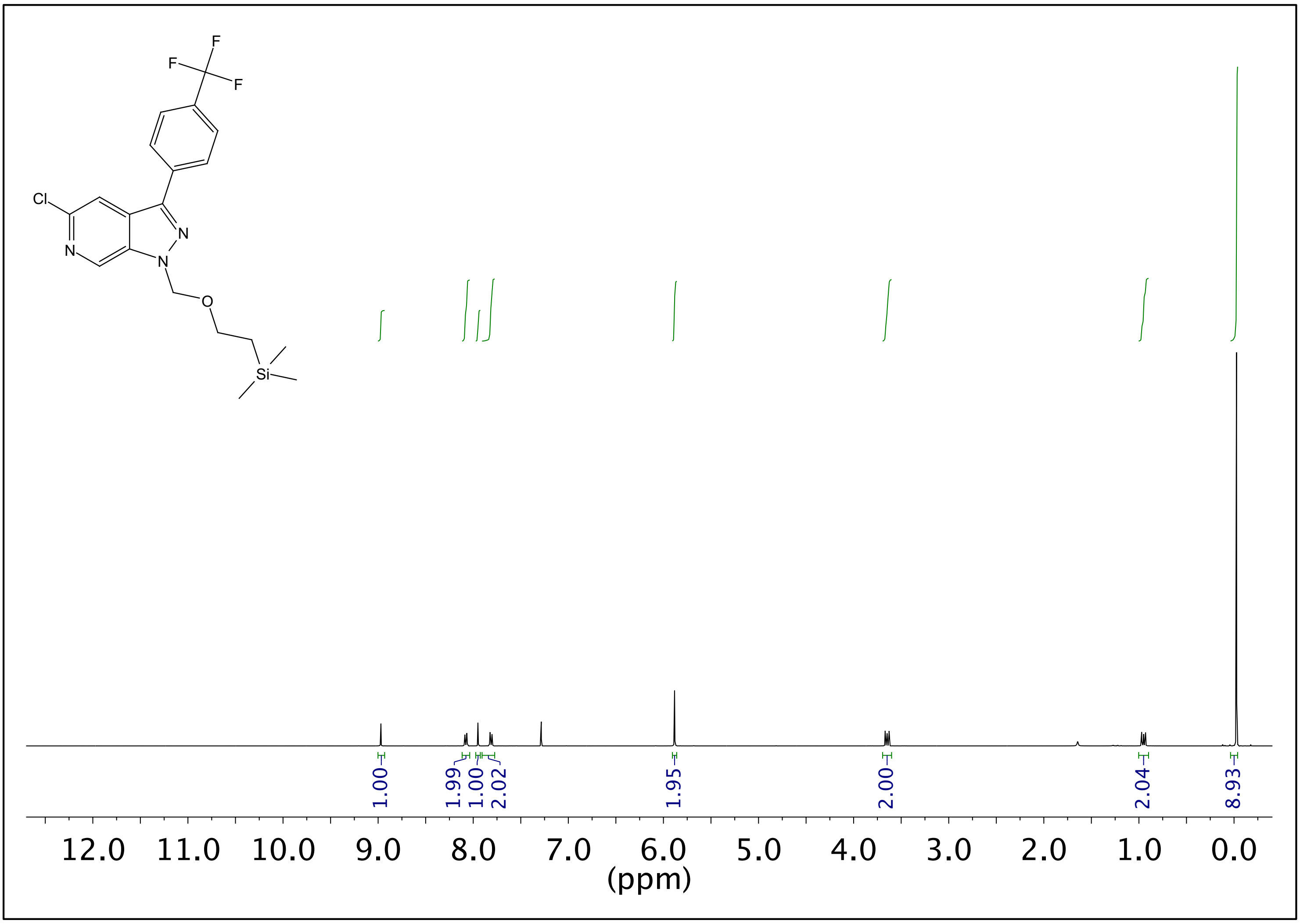

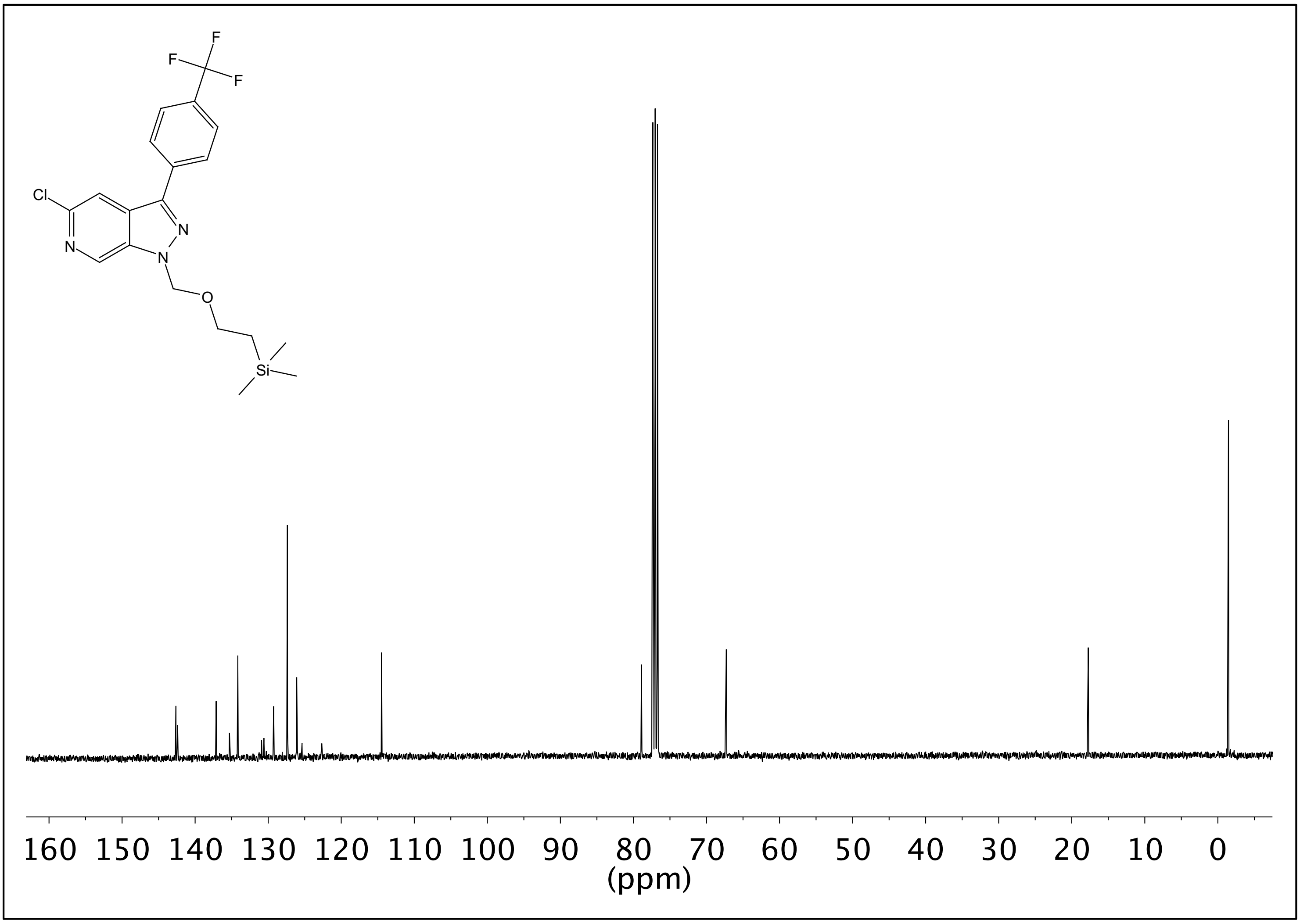

^{19}F NMR (376 MHz, Chloroform-*d*) of compound **21**

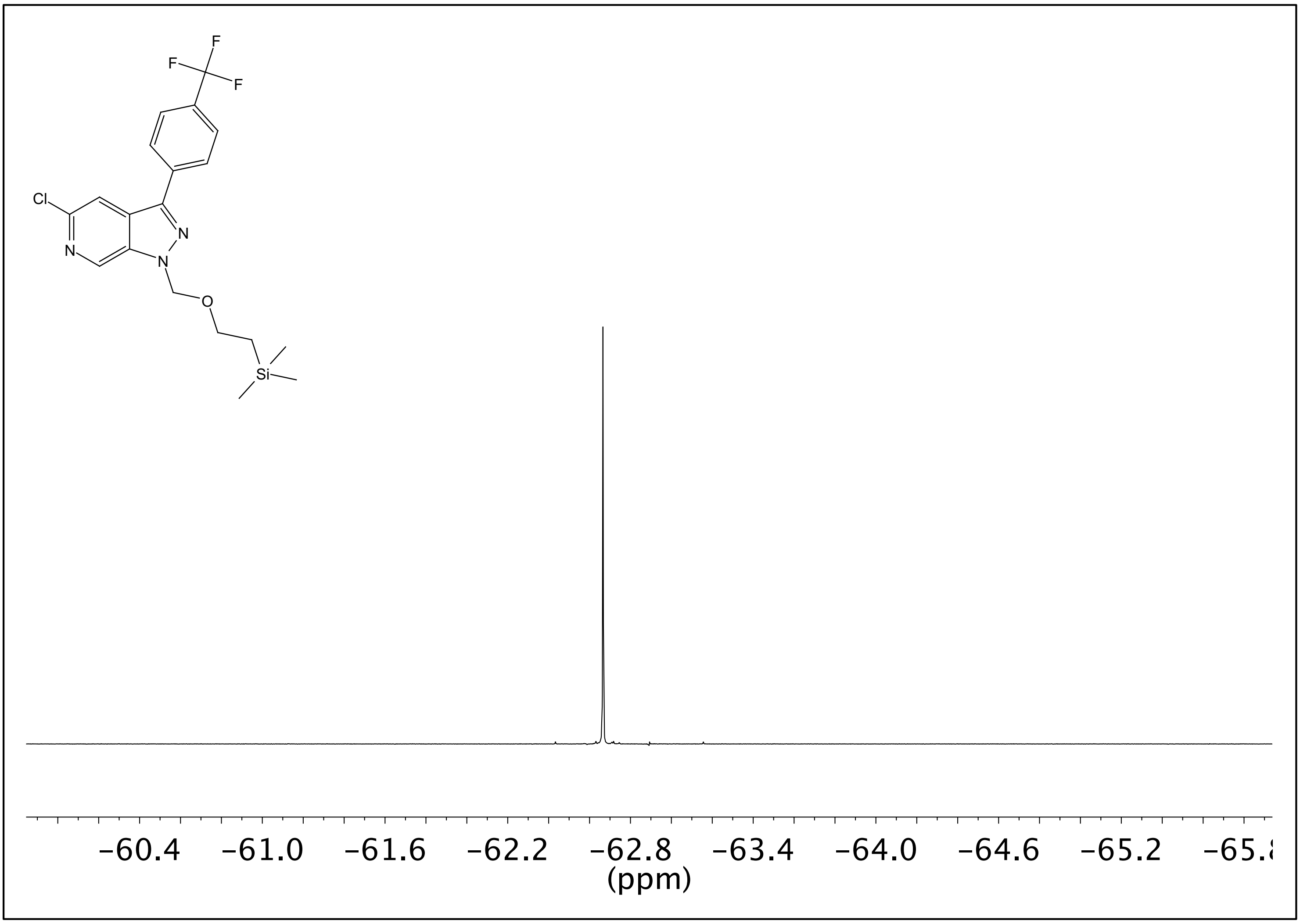

^{1}H NMR (600 MHz, Chloroform-*d*) and ^{13}C NMR (151 MHz, Chloroform-*d*) of compound **22**

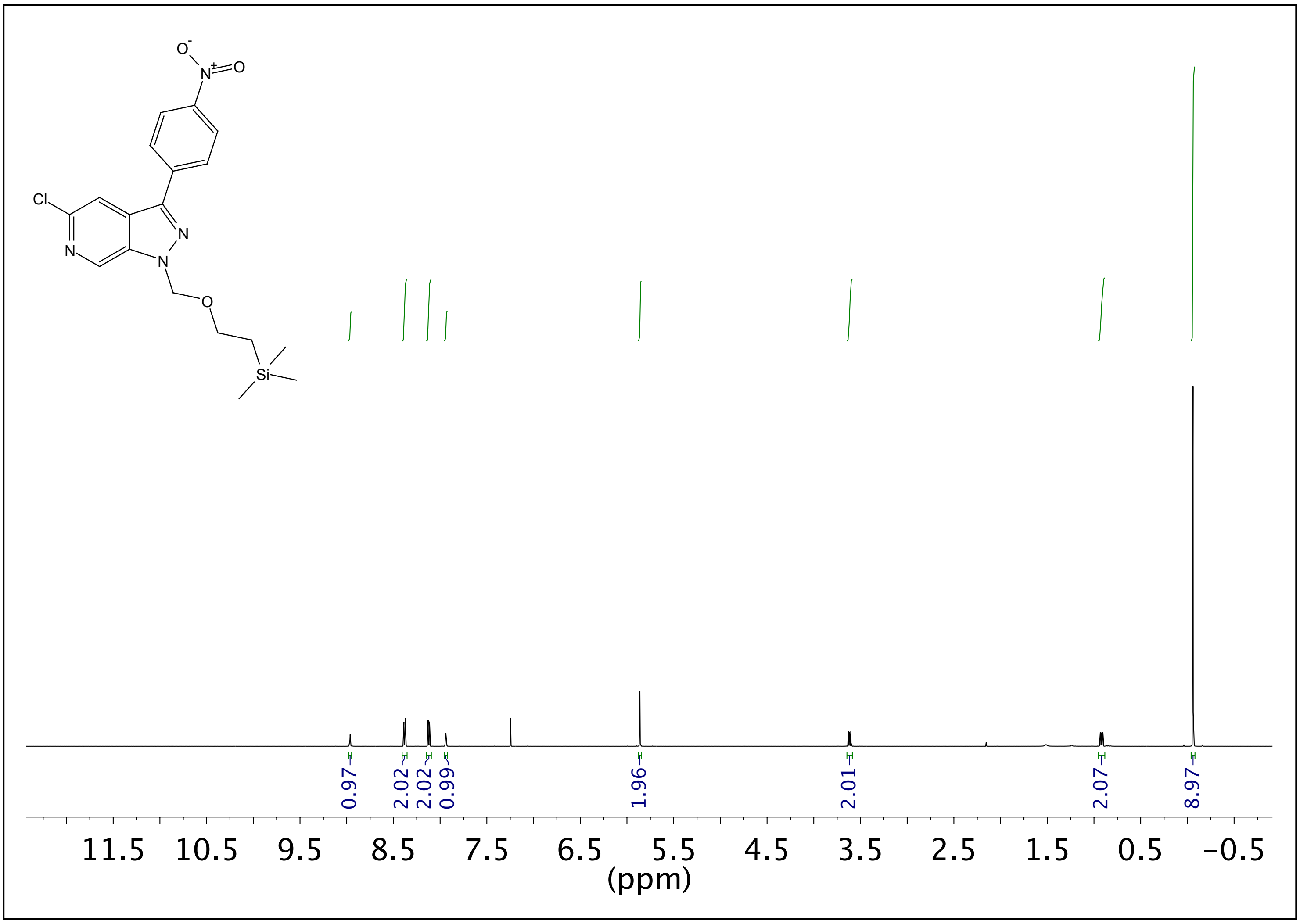

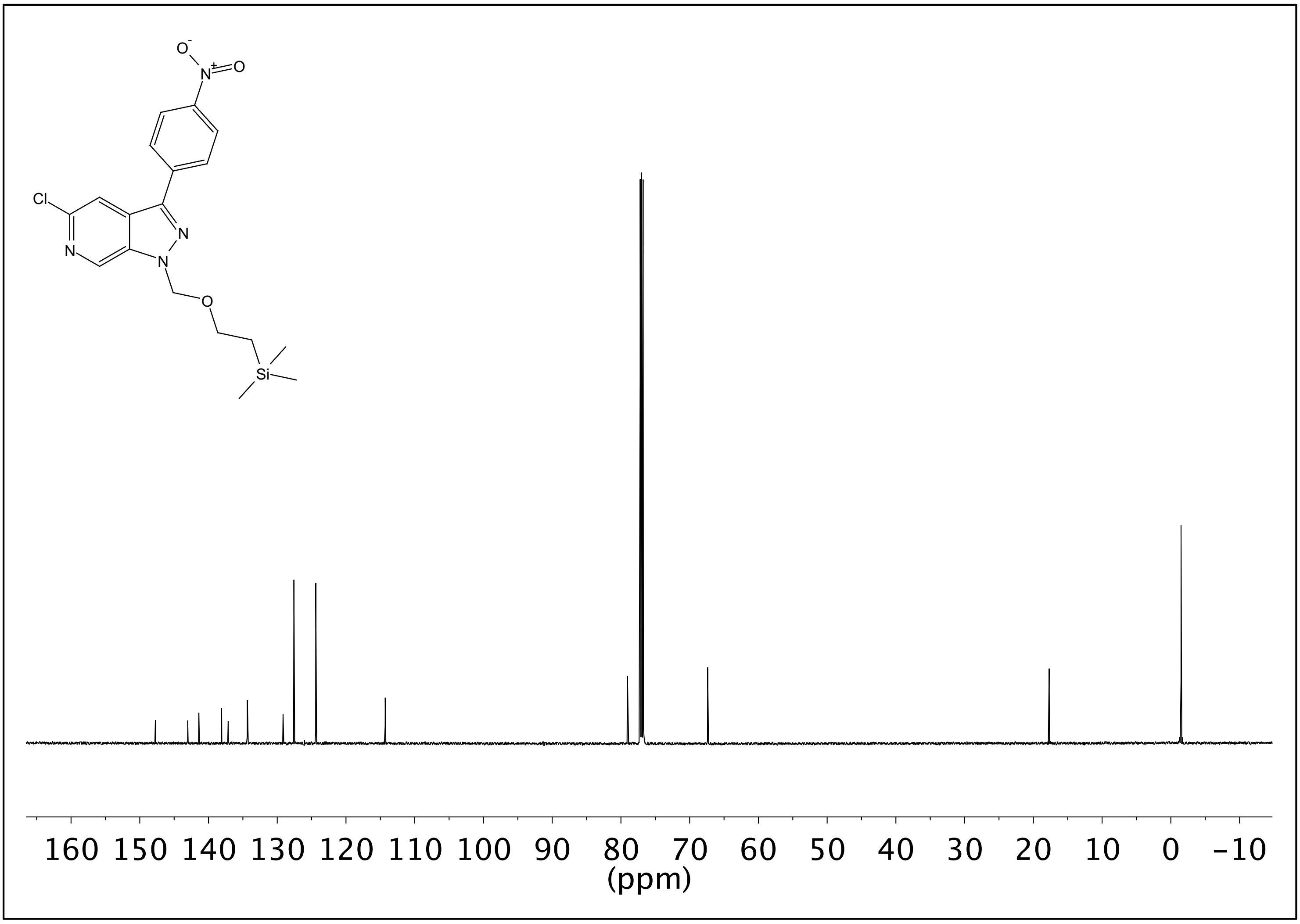

^{1}H NMR (600 MHz, Chloroform-*d*) and ^{13}C NMR (151 MHz, Chloroform-*d*) of compound **23**

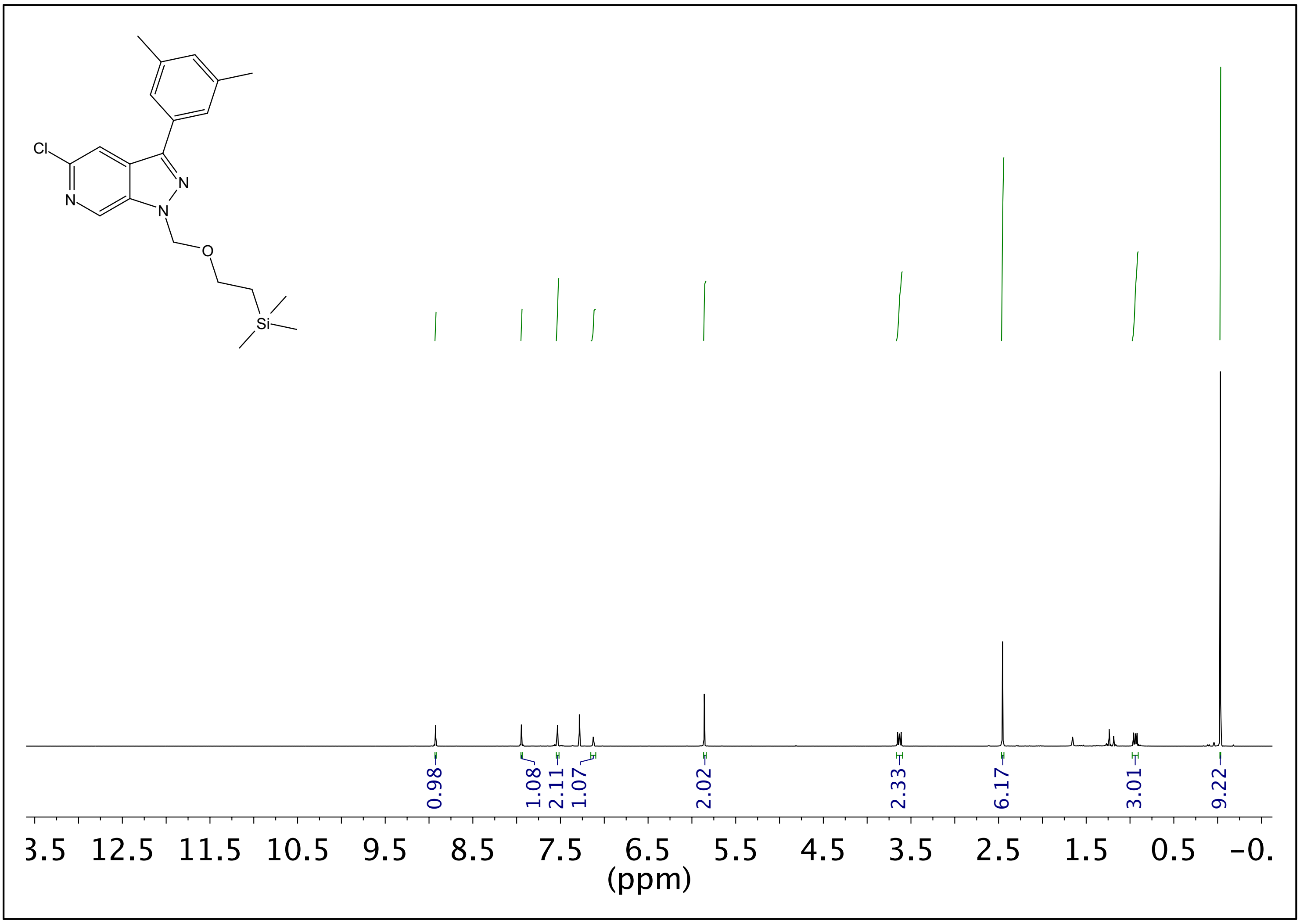

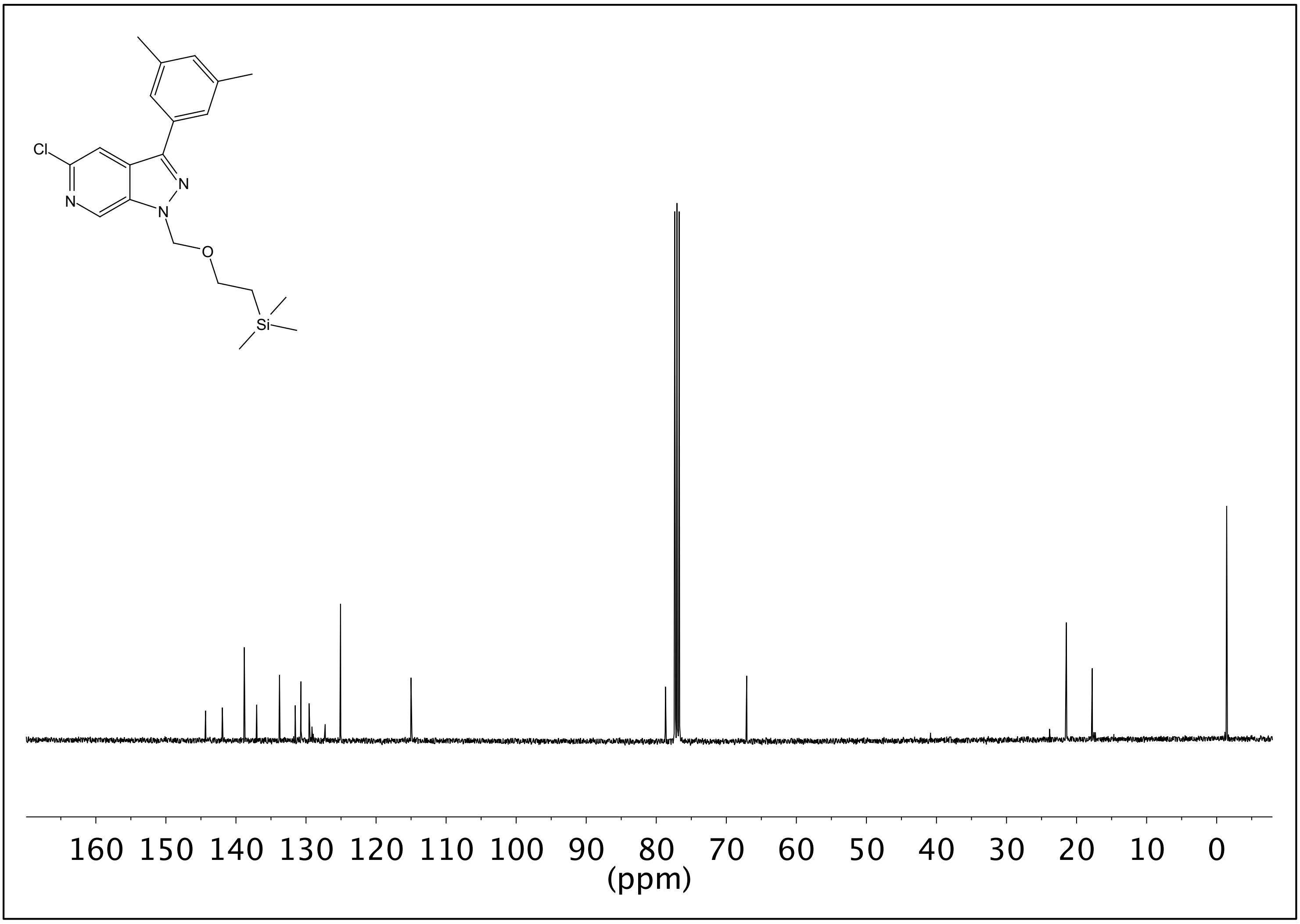

^{1}H NMR (600 MHz, Chloroform-*d*) and ^{13}C NMR (151 MHz, Chloroform-*d*) of compound **24**

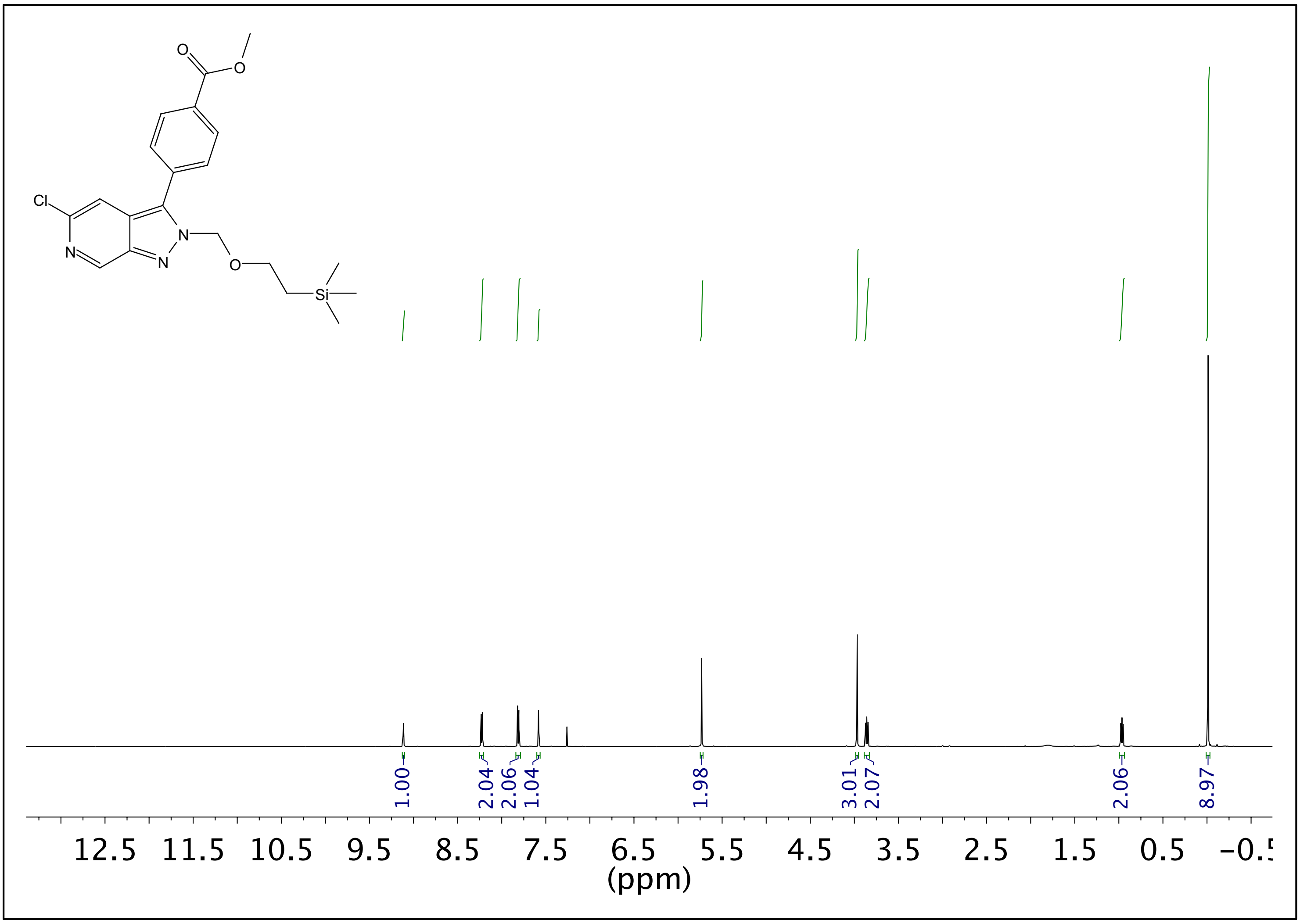

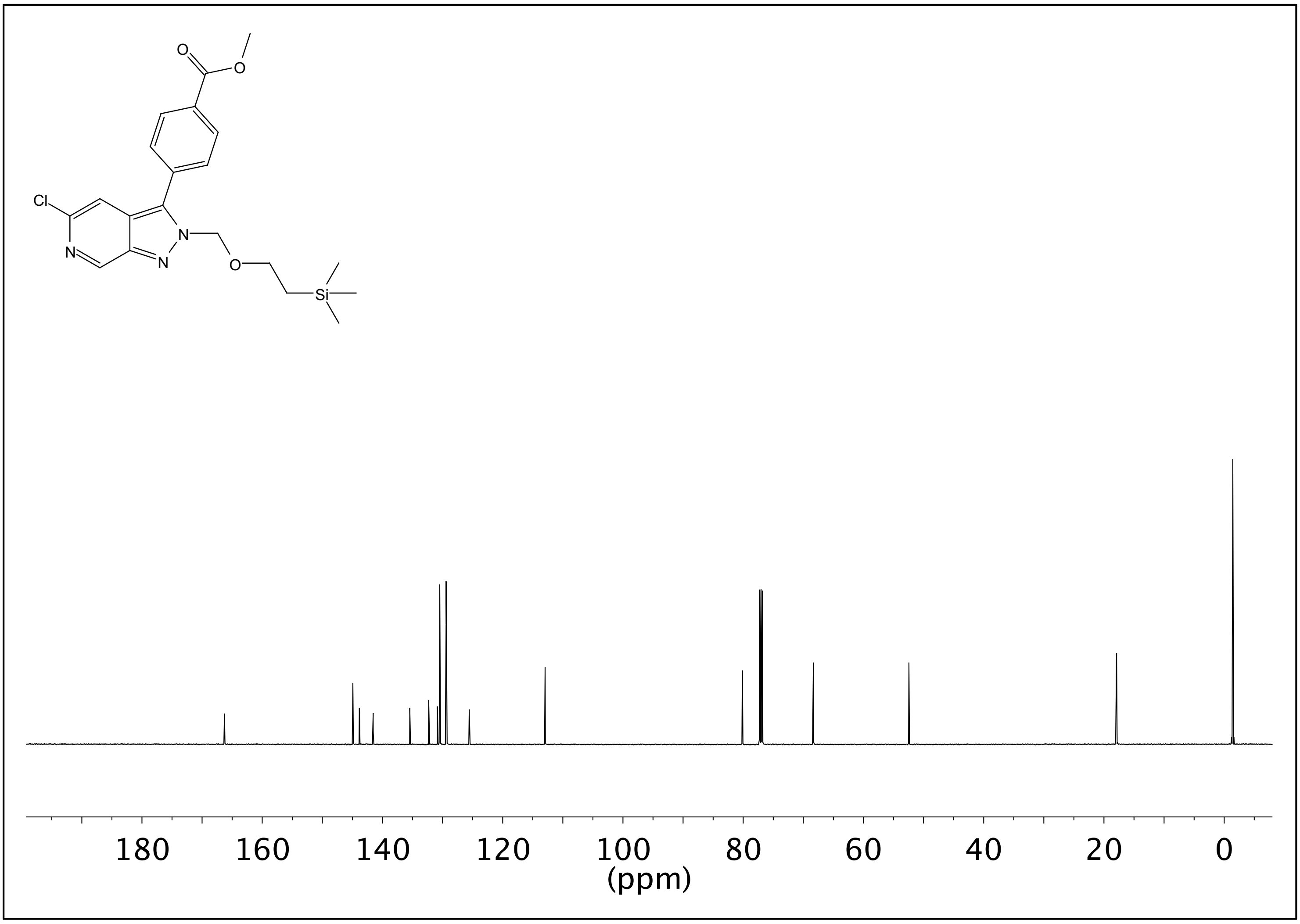

^1H NMR (600 MHz, Chloroform-*d*) and ^{13}C NMR (151 MHz, Chloroform-*d*) of compound **25**

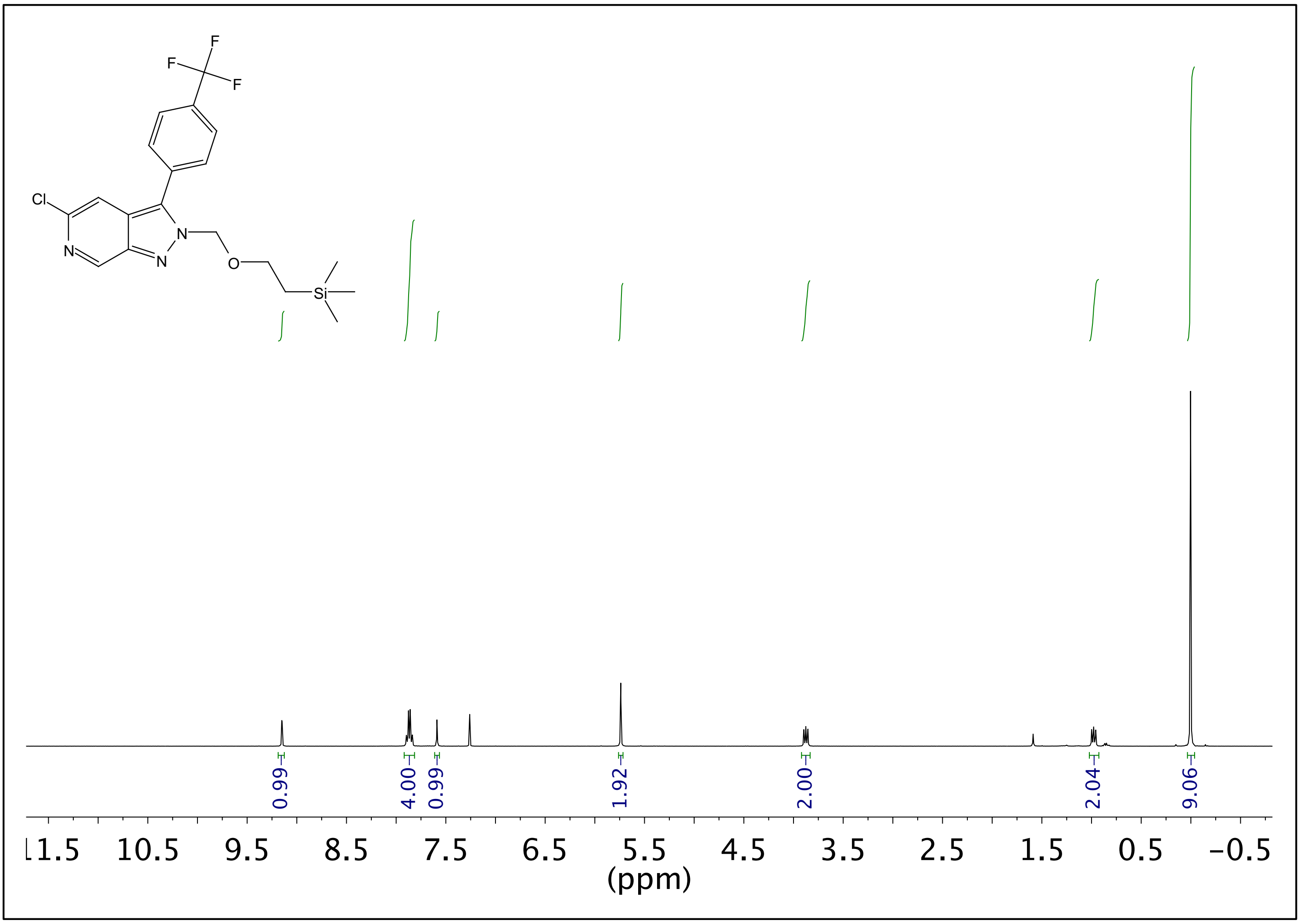

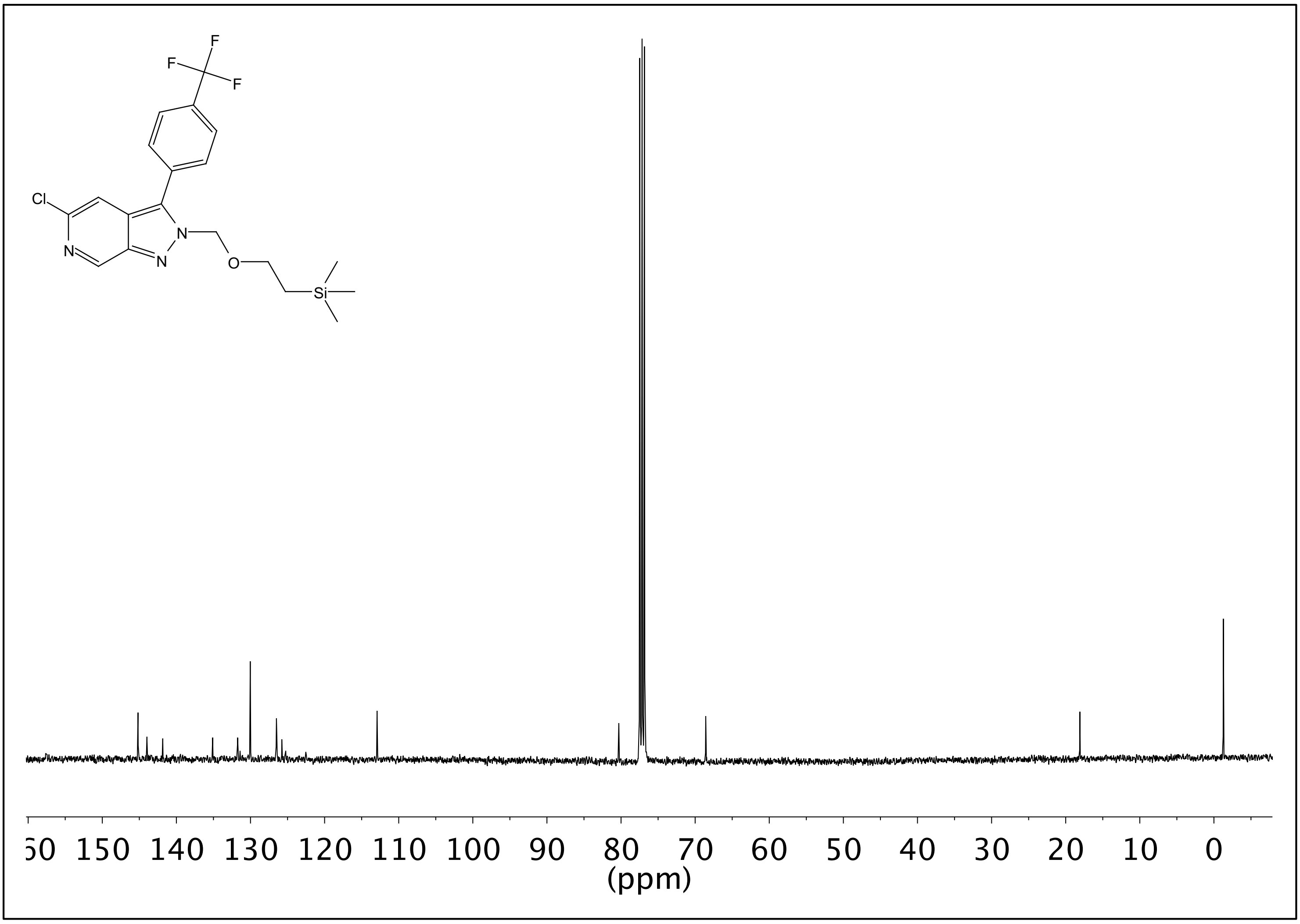

^{19}F NMR (376 MHz, Chloroform-*d*) of compound **25**

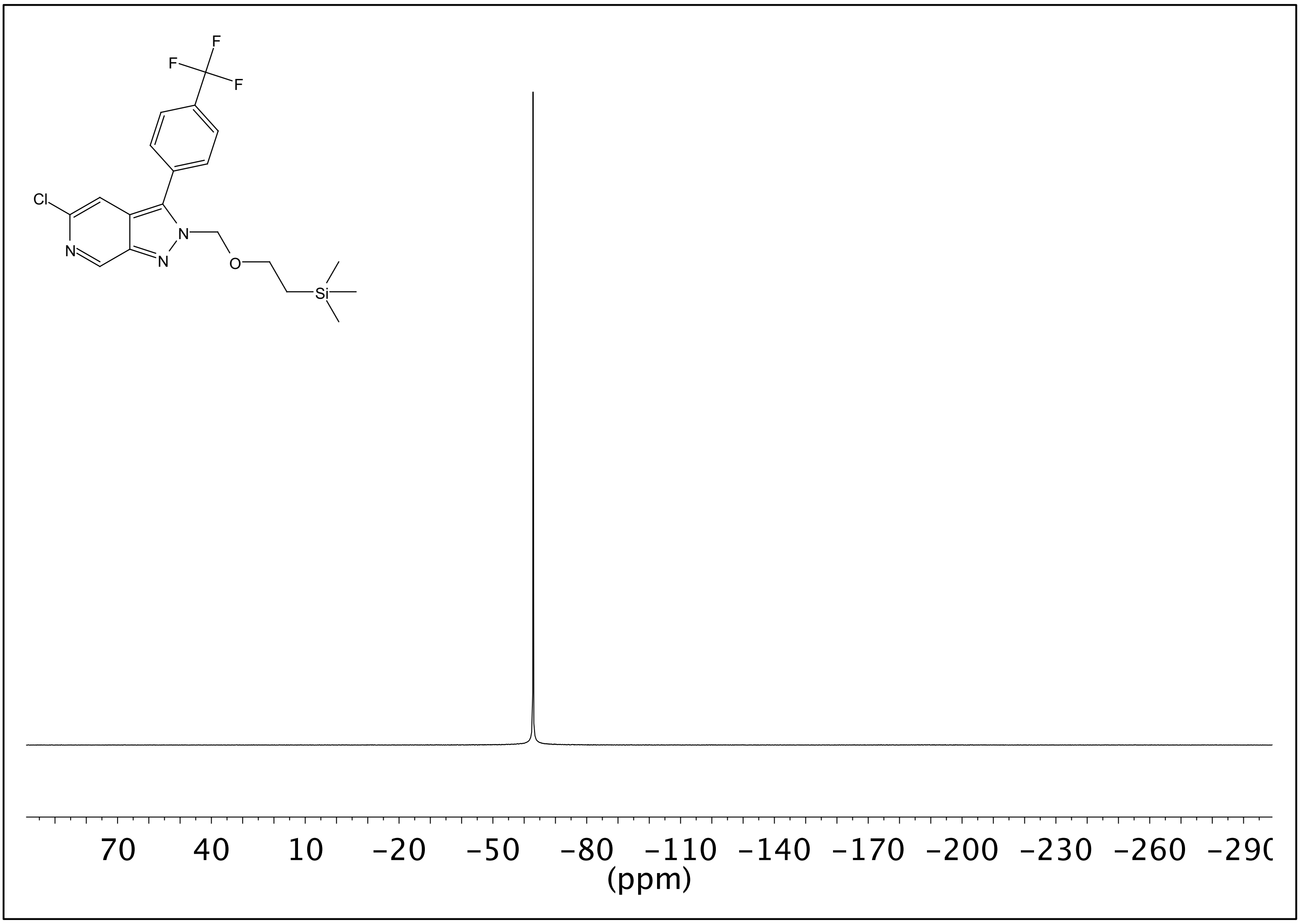

^{1}H NMR (600 MHz, Chloroform-*d*) and ^{13}C NMR (151 MHz, Chloroform-*d*) of compound **26**

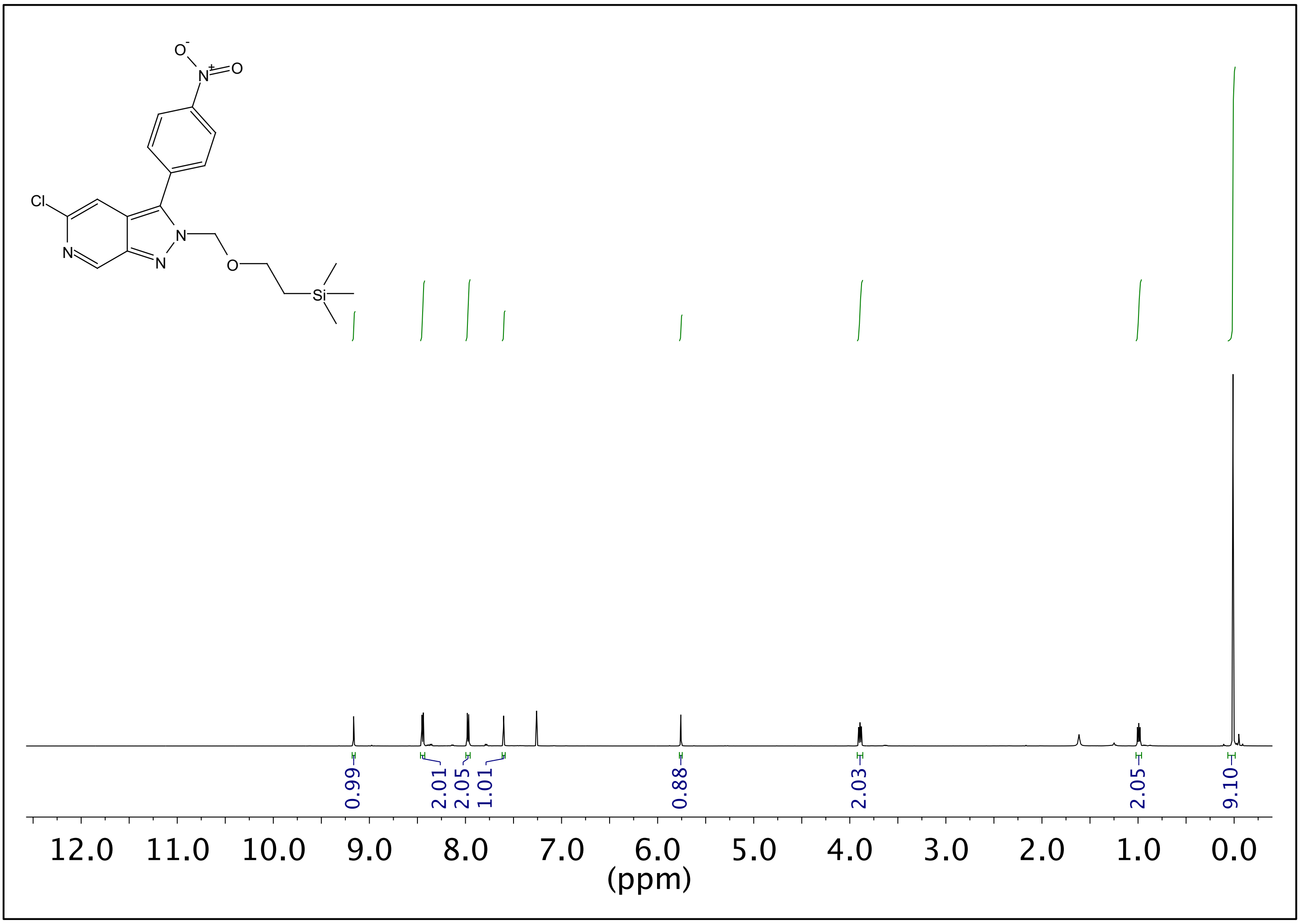

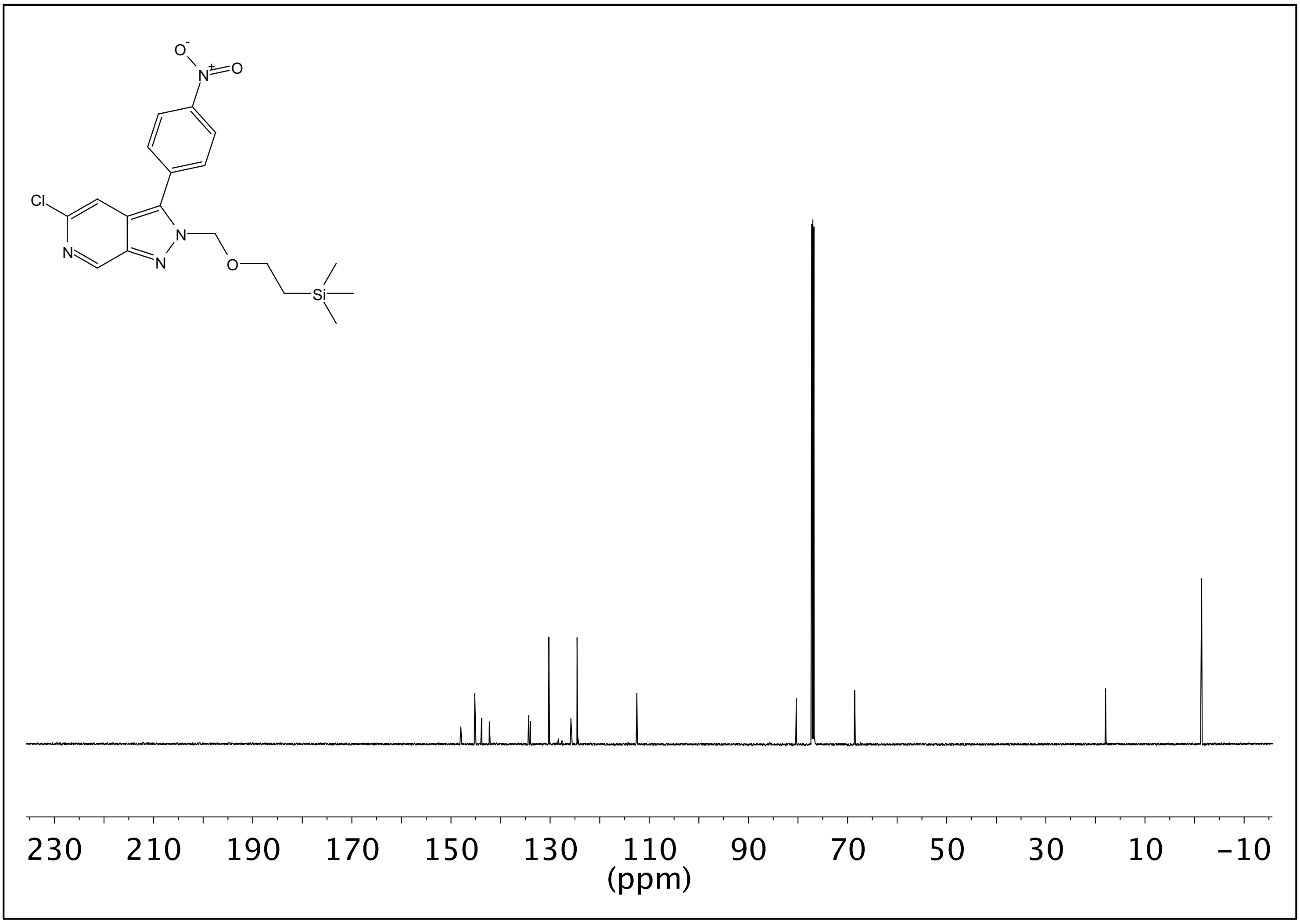

^{1}H NMR (600 MHz, Chloroform-*d*) and ^{13}C NMR (151 MHz, Chloroform-*d*) of compound **27**

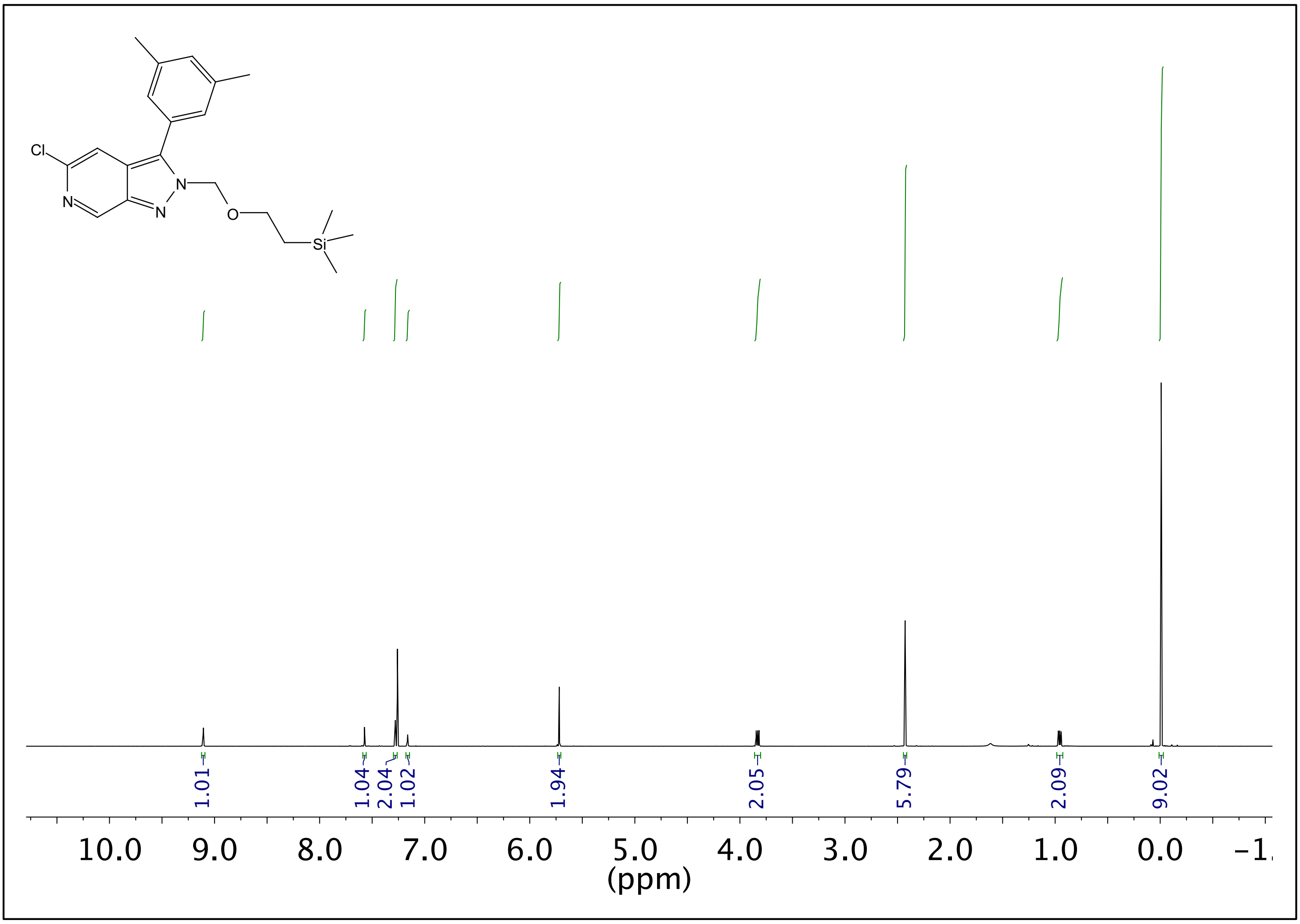

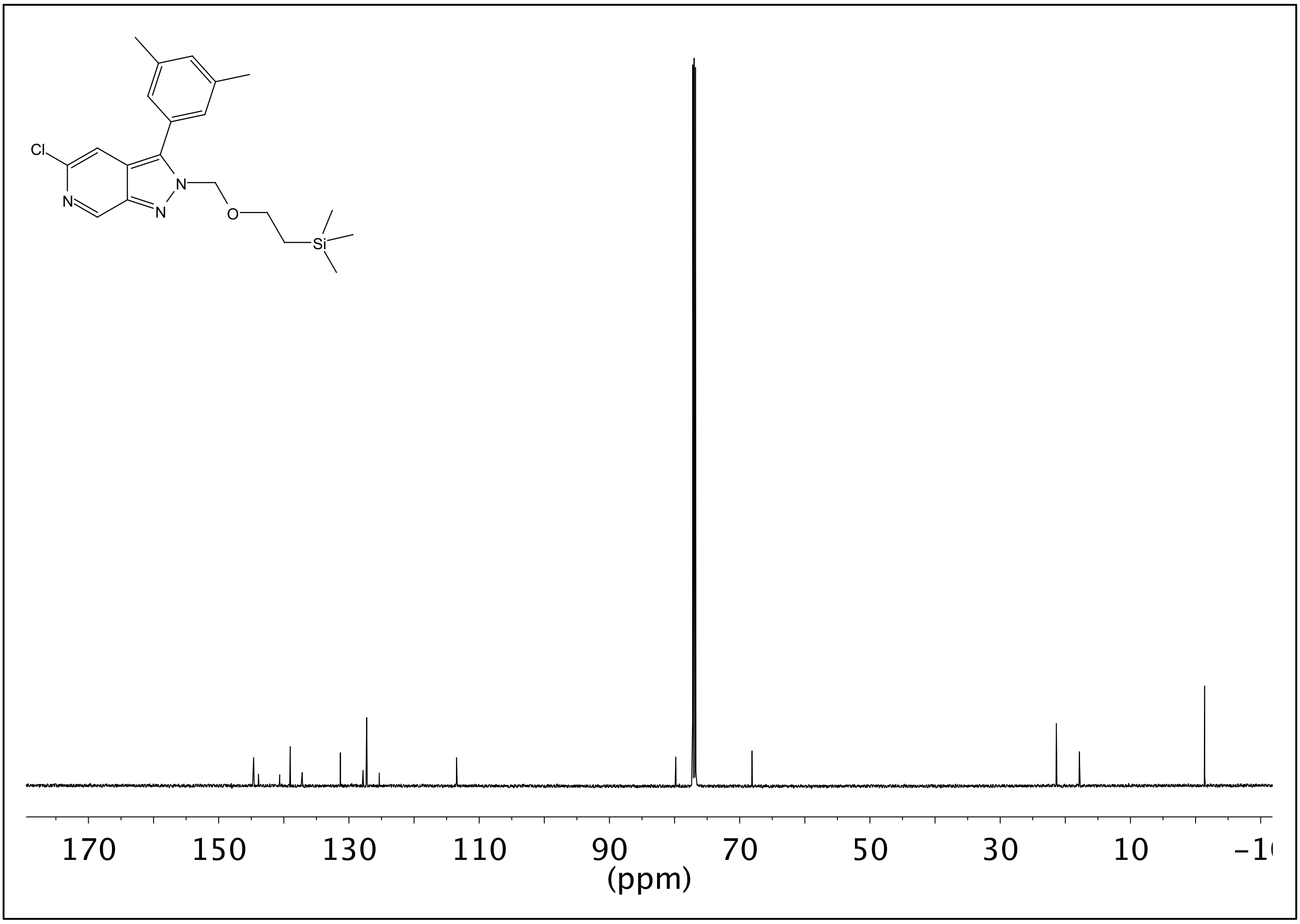

^{1}H NMR (700 MHz, Chloroform-*d*) and ^{13}C NMR (176 MHz, Chloroform-*d*) of compound **28**

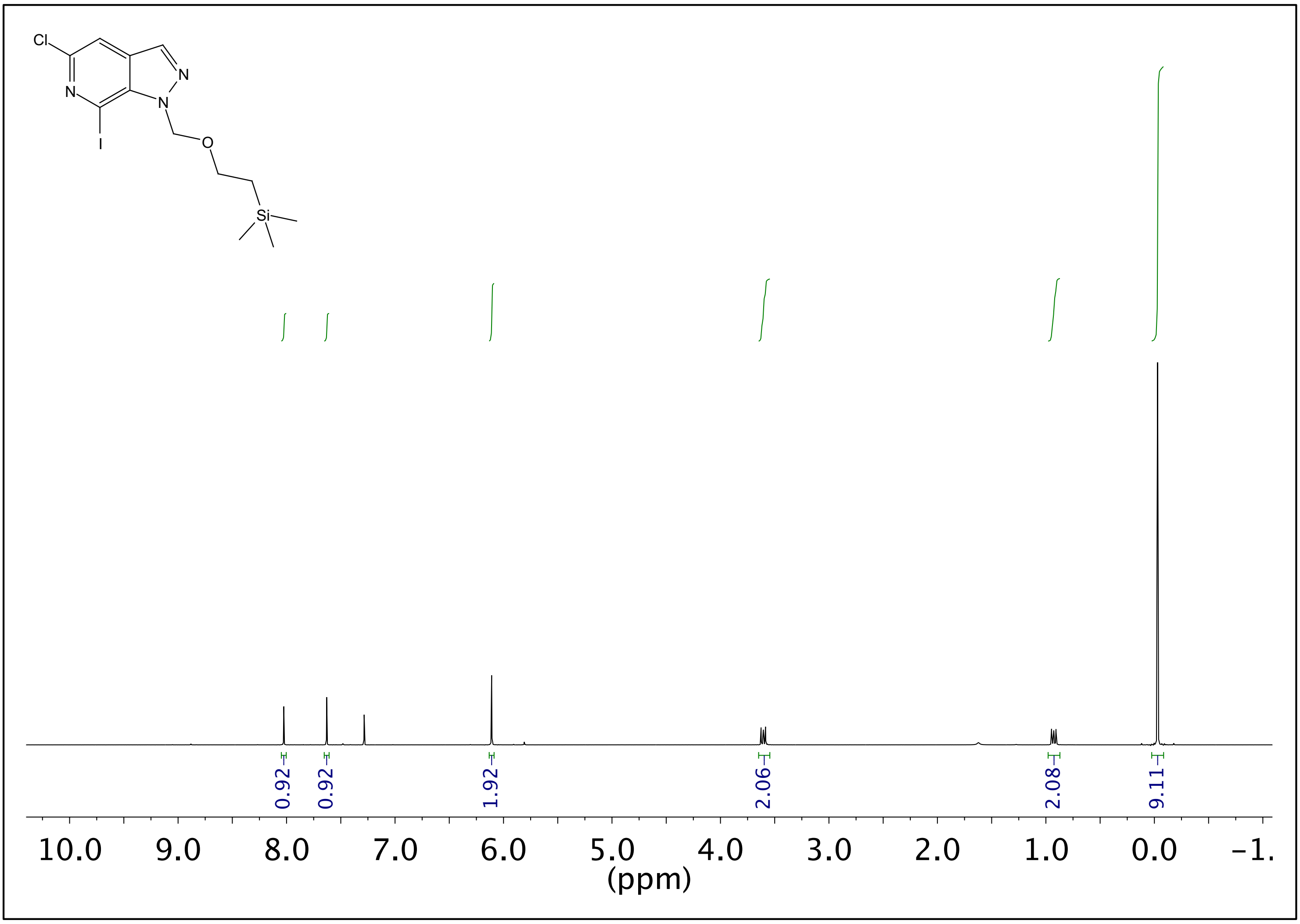

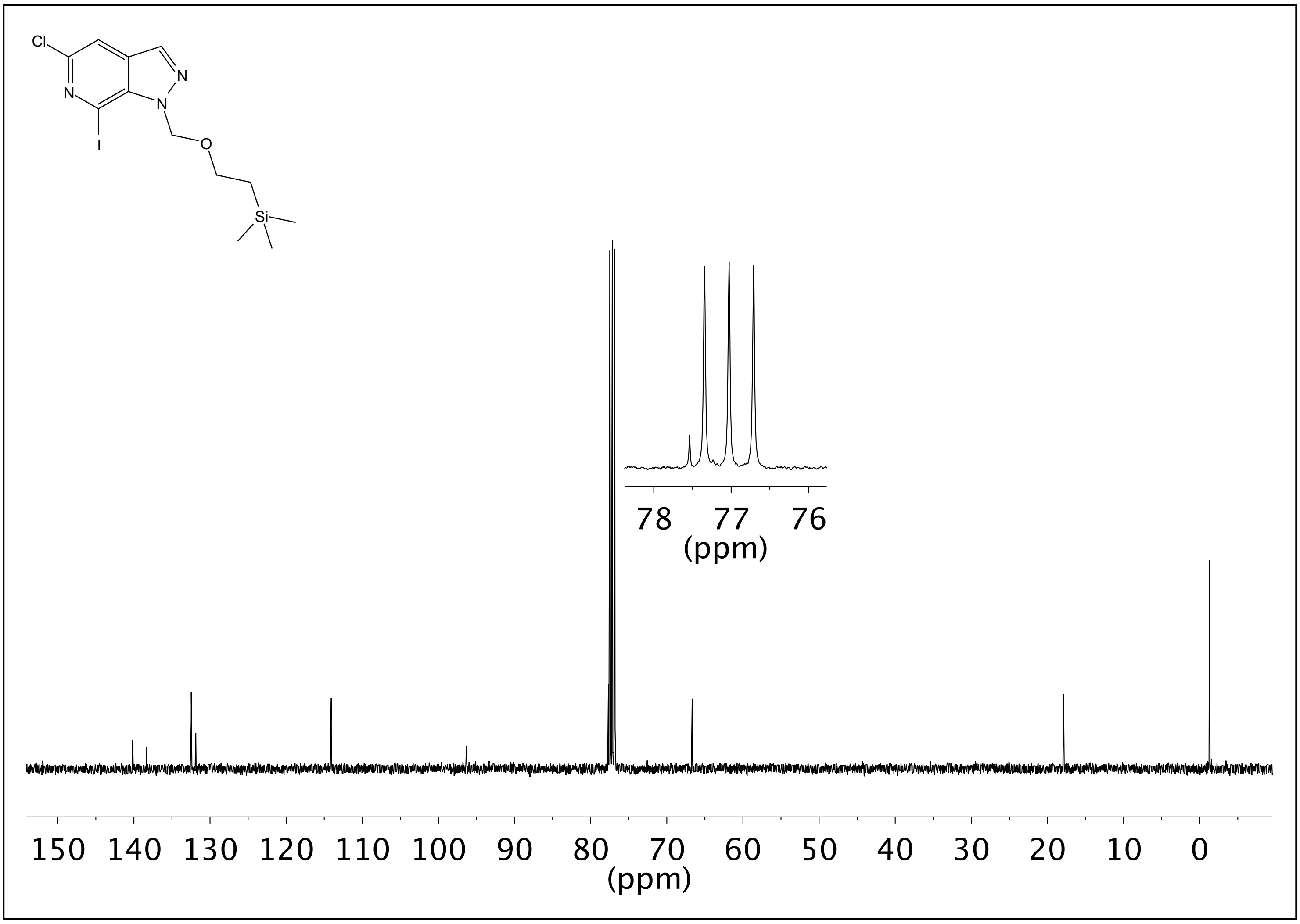

^{1}H NMR (700 MHz, Chloroform-*d*) and ^{13}C NMR (176 MHz, Chloroform-*d*) of compound **29**

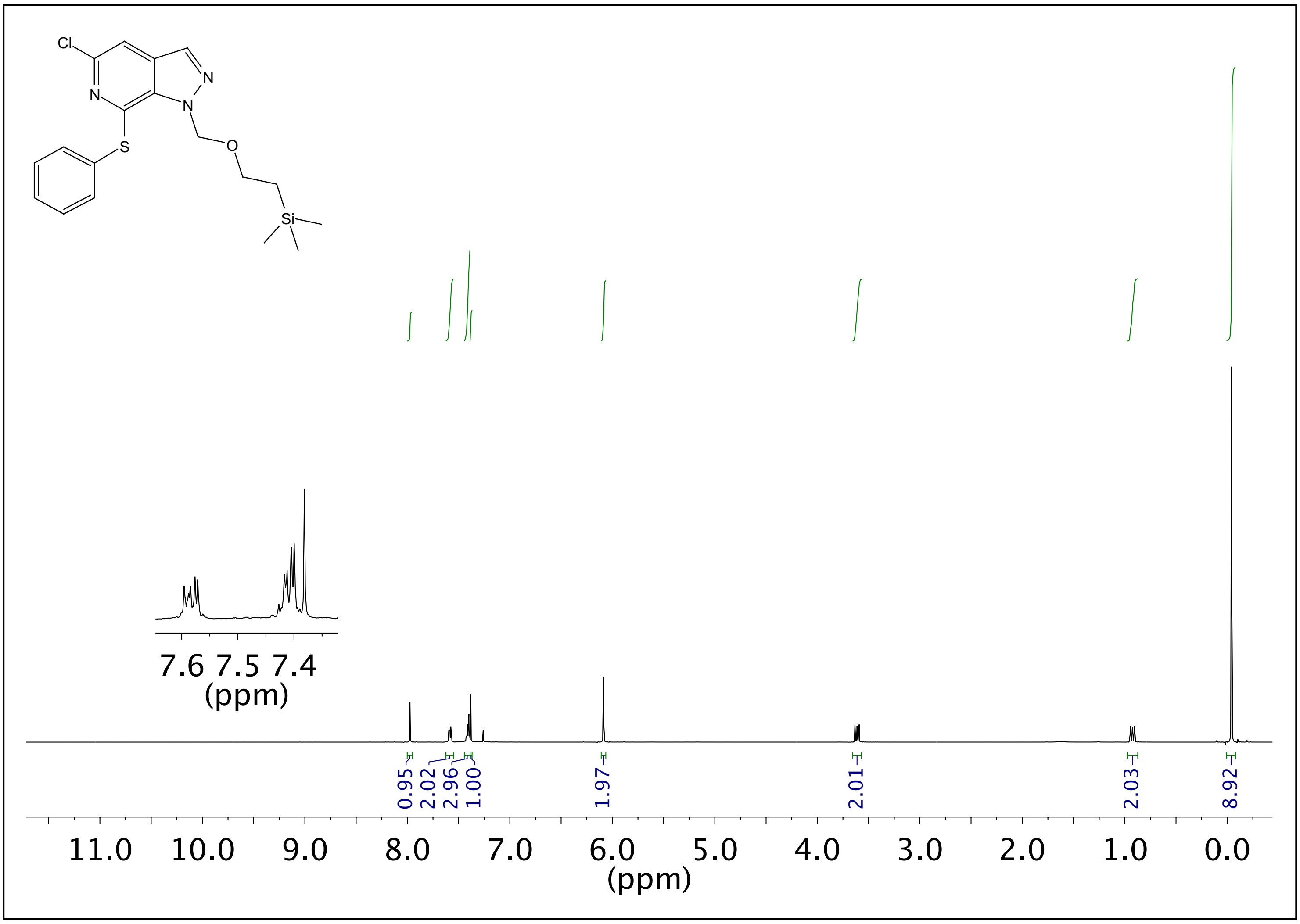

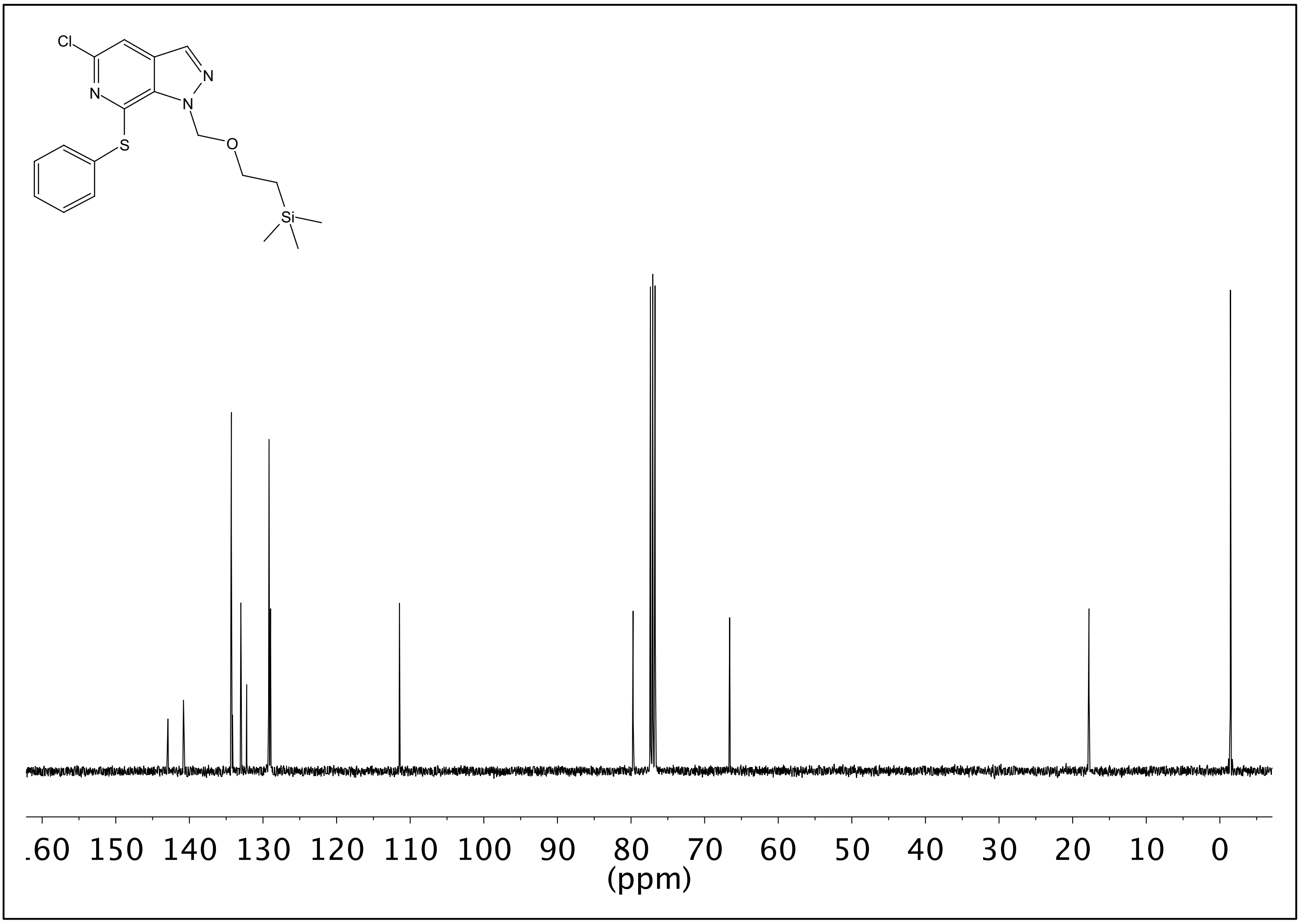

^{1}H NMR (600 MHz, Chloroform-*d*) and ^{13}C NMR (151 MHz, Chloroform-*d*) of compound **30**

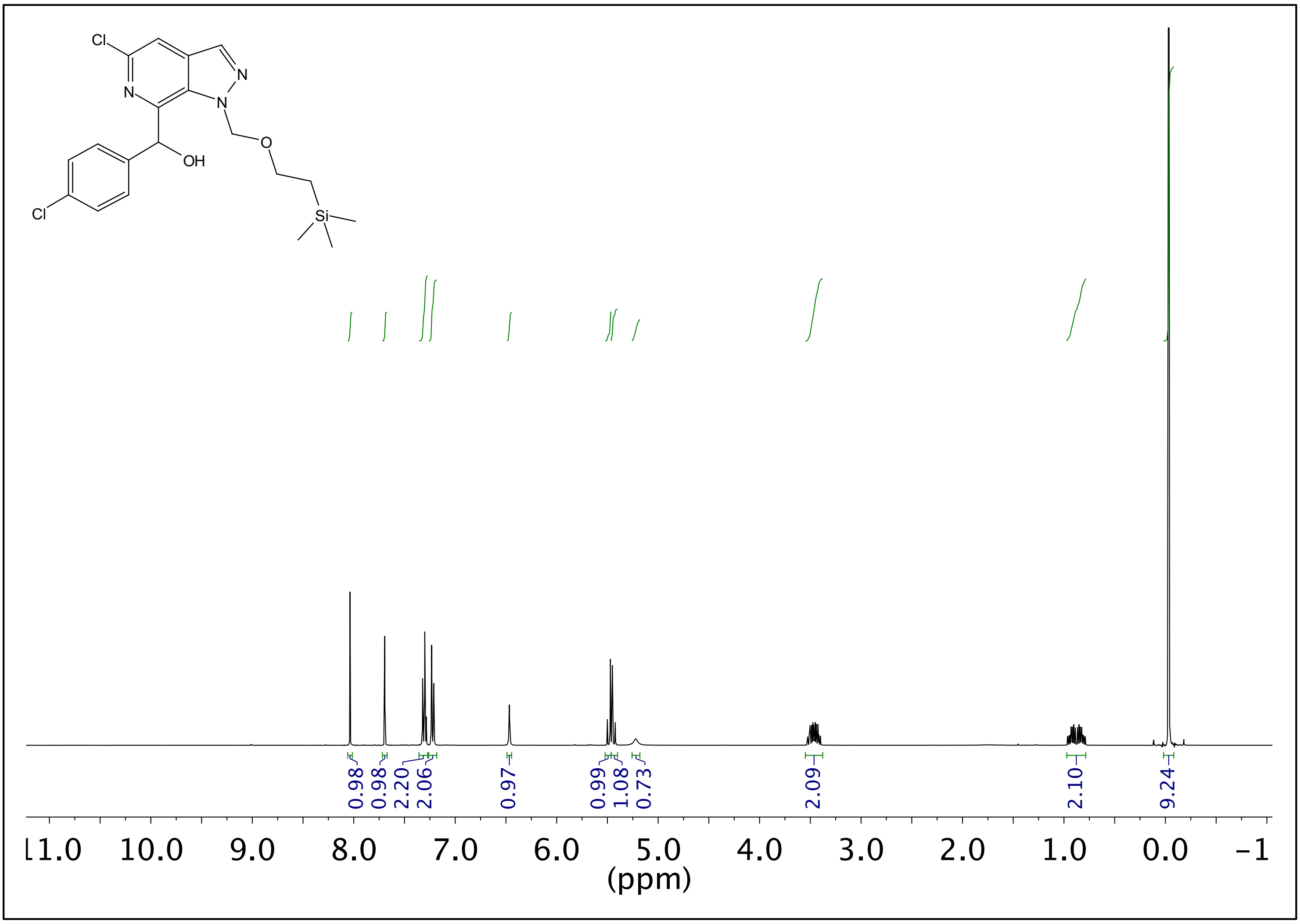

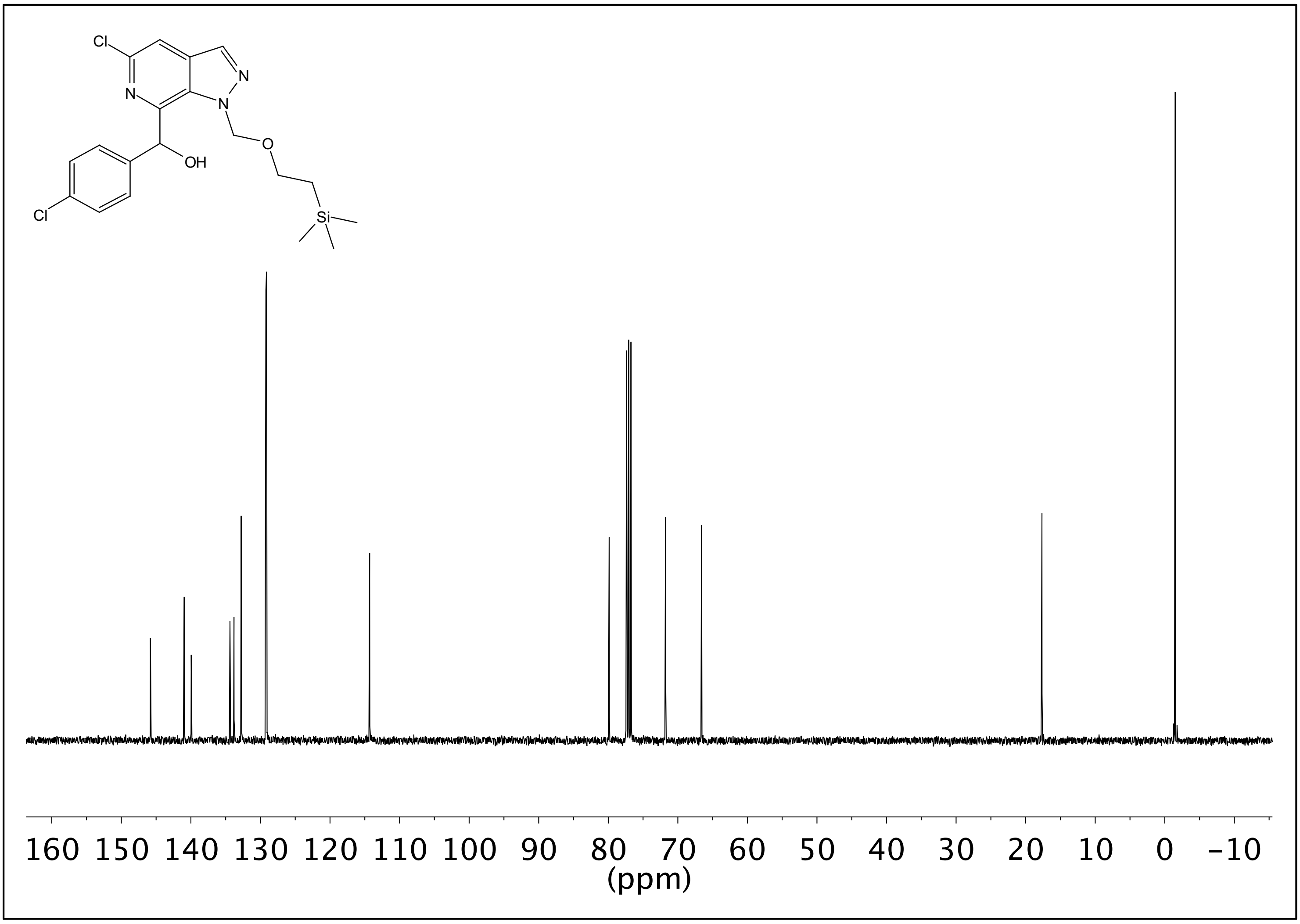

^{1}H NMR (600 MHz, Chloroform-*d*) and ^{13}C NMR (151 MHz, Chloroform-*d*) of compound **31**

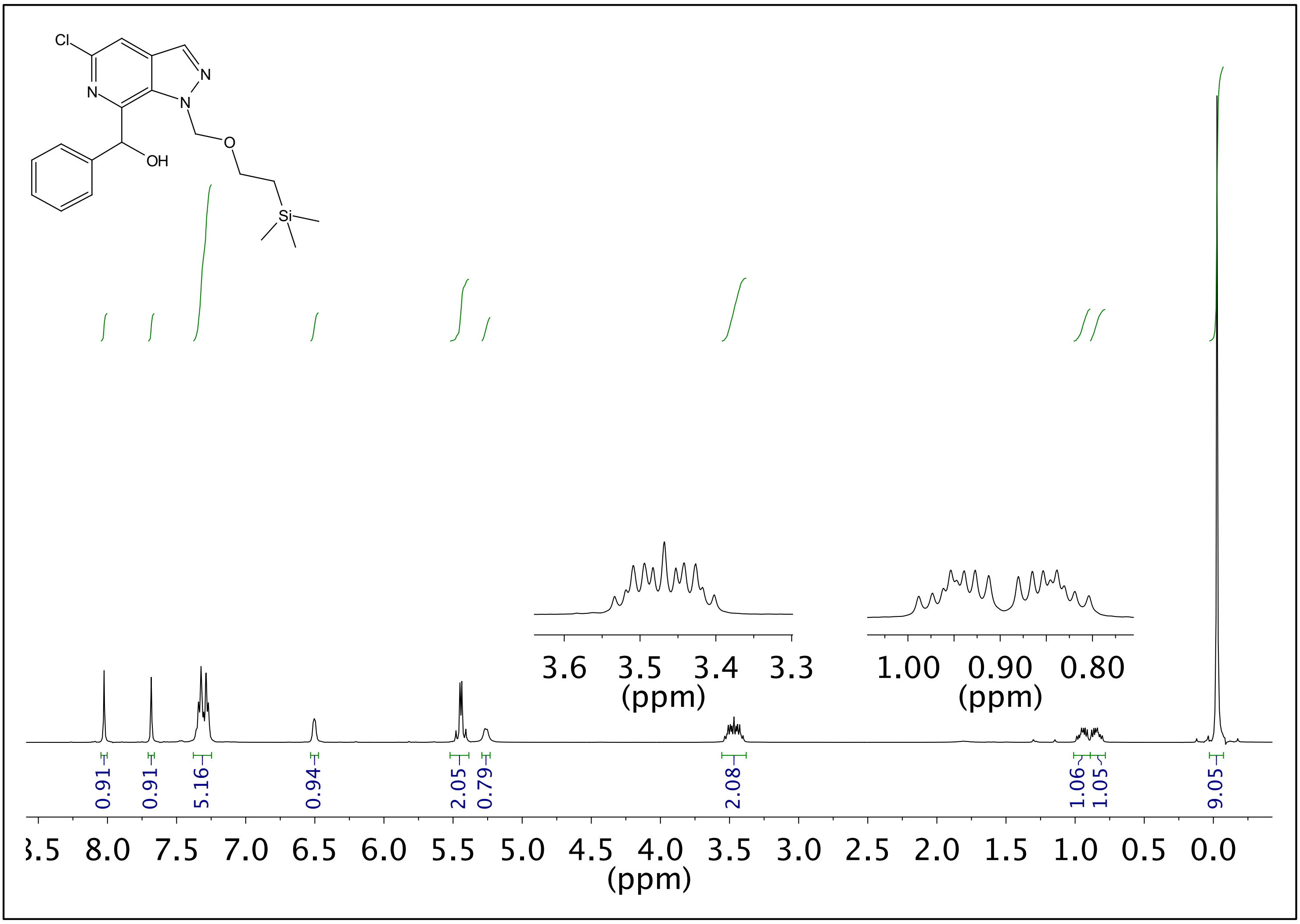

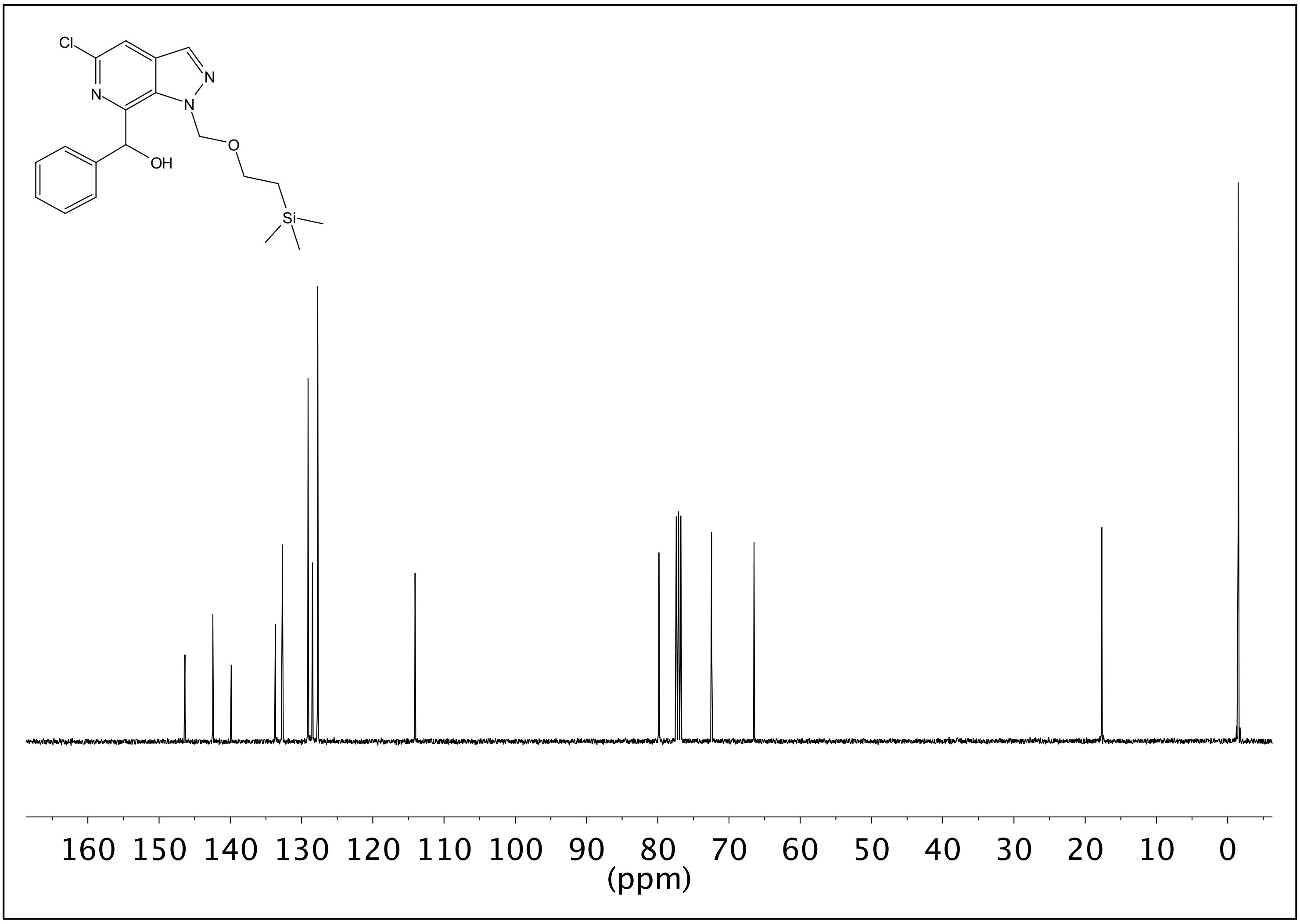

^{1}H NMR (600 MHz, Chloroform-*d*) and ^{13}C NMR (151 MHz, Chloroform-*d*) of compound **32**

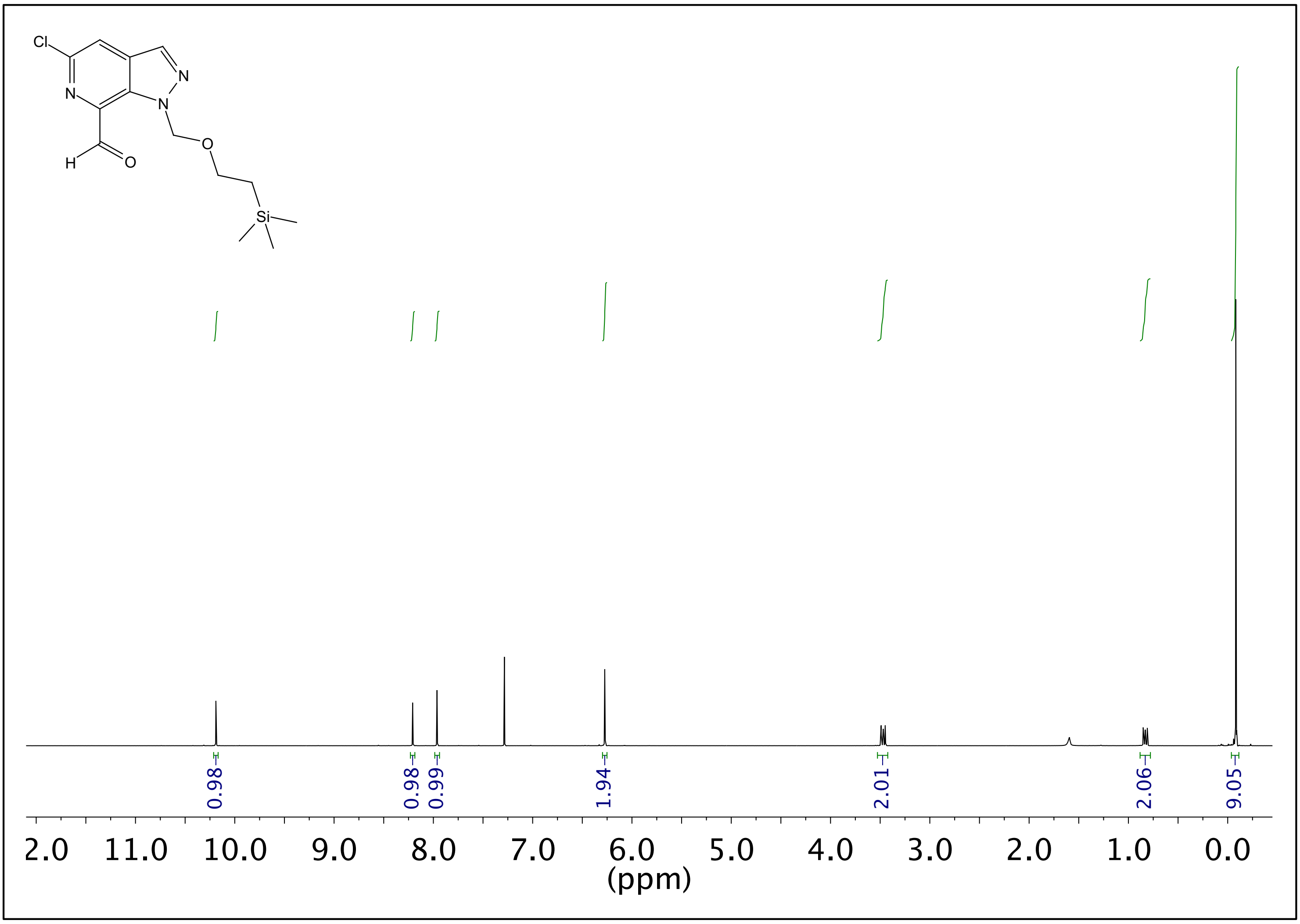

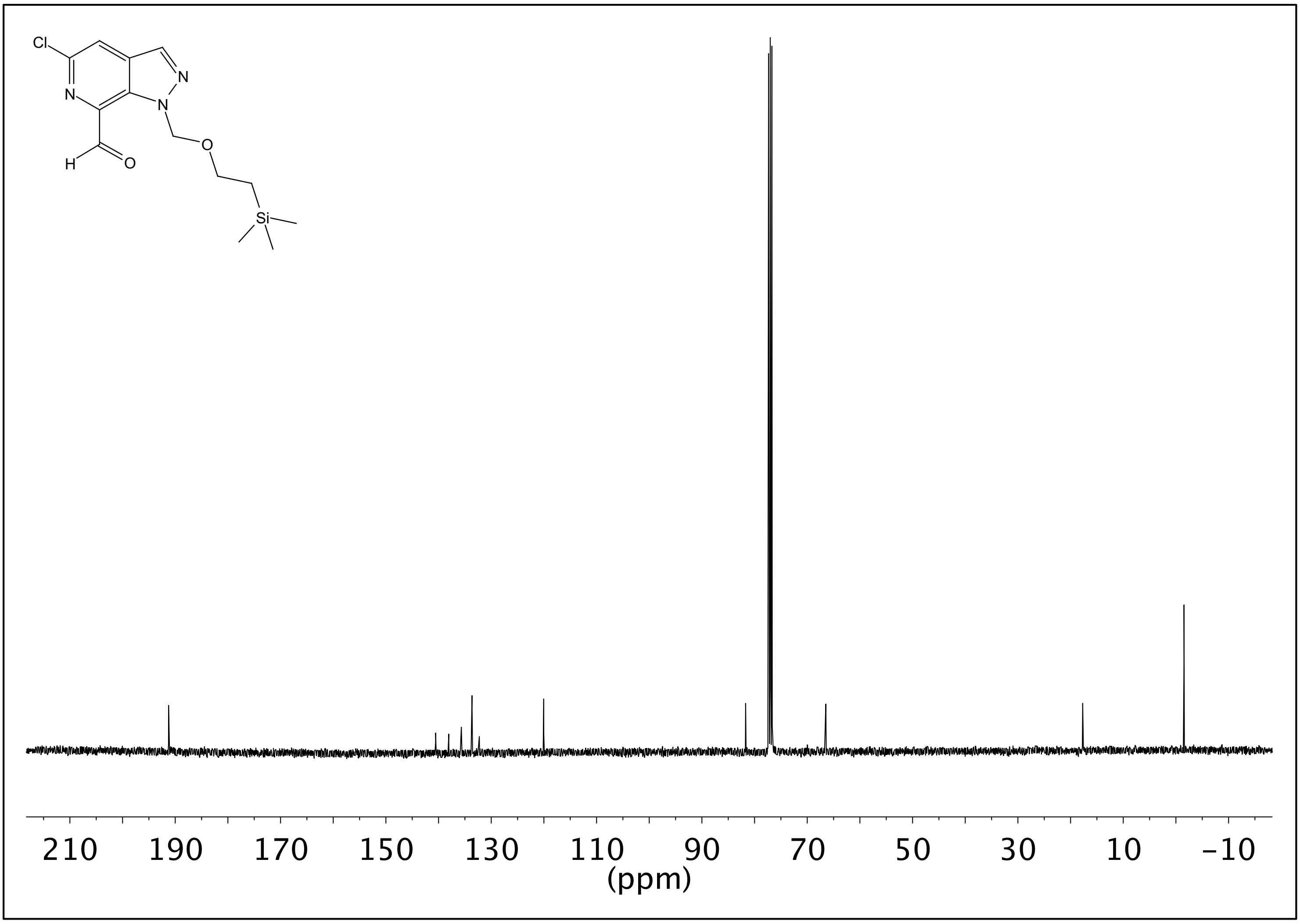

^{1}H NMR (600 MHz, Chloroform-*d*) and ^{13}C NMR (151 MHz, Chloroform-*d*) of compound **33**

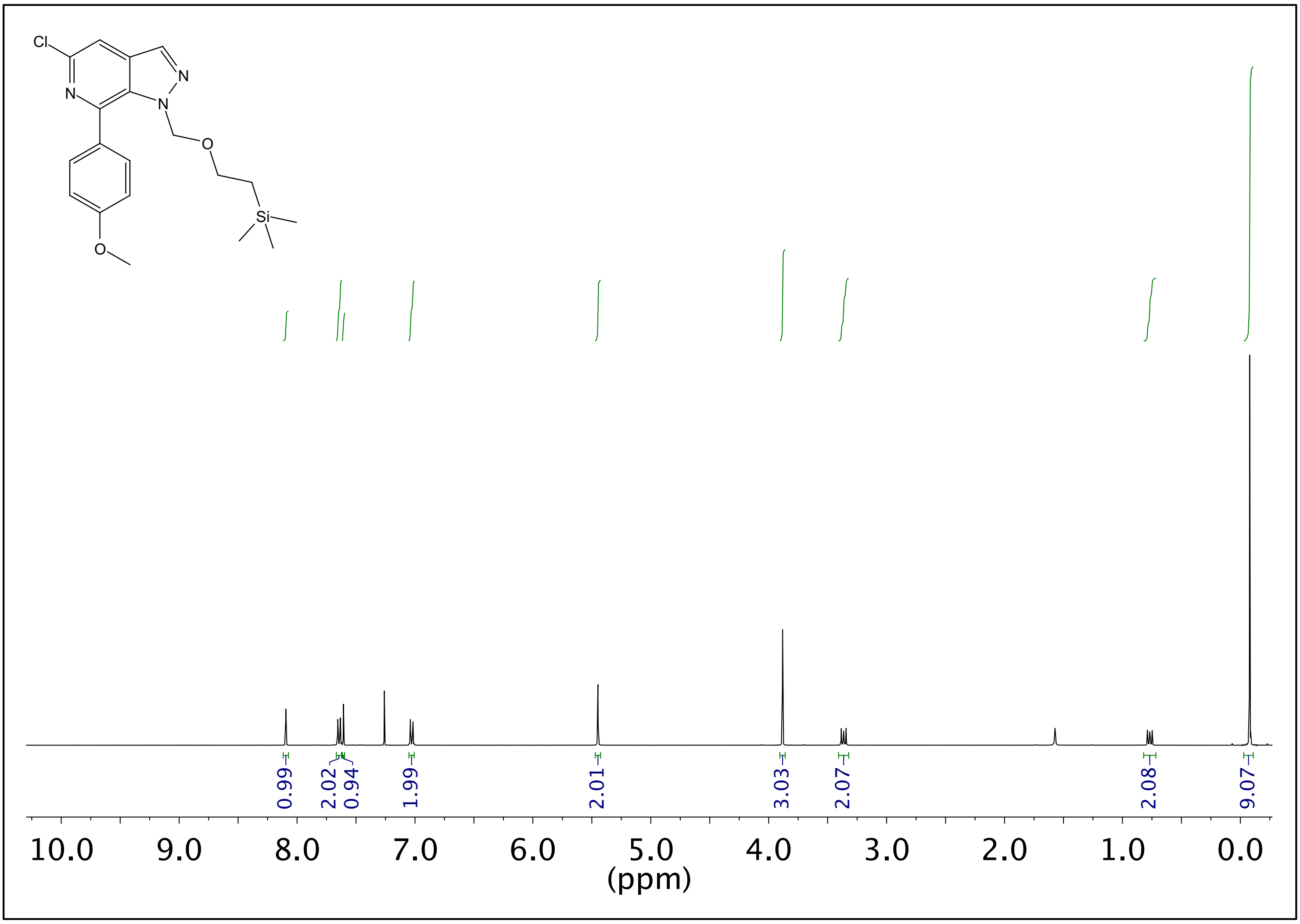

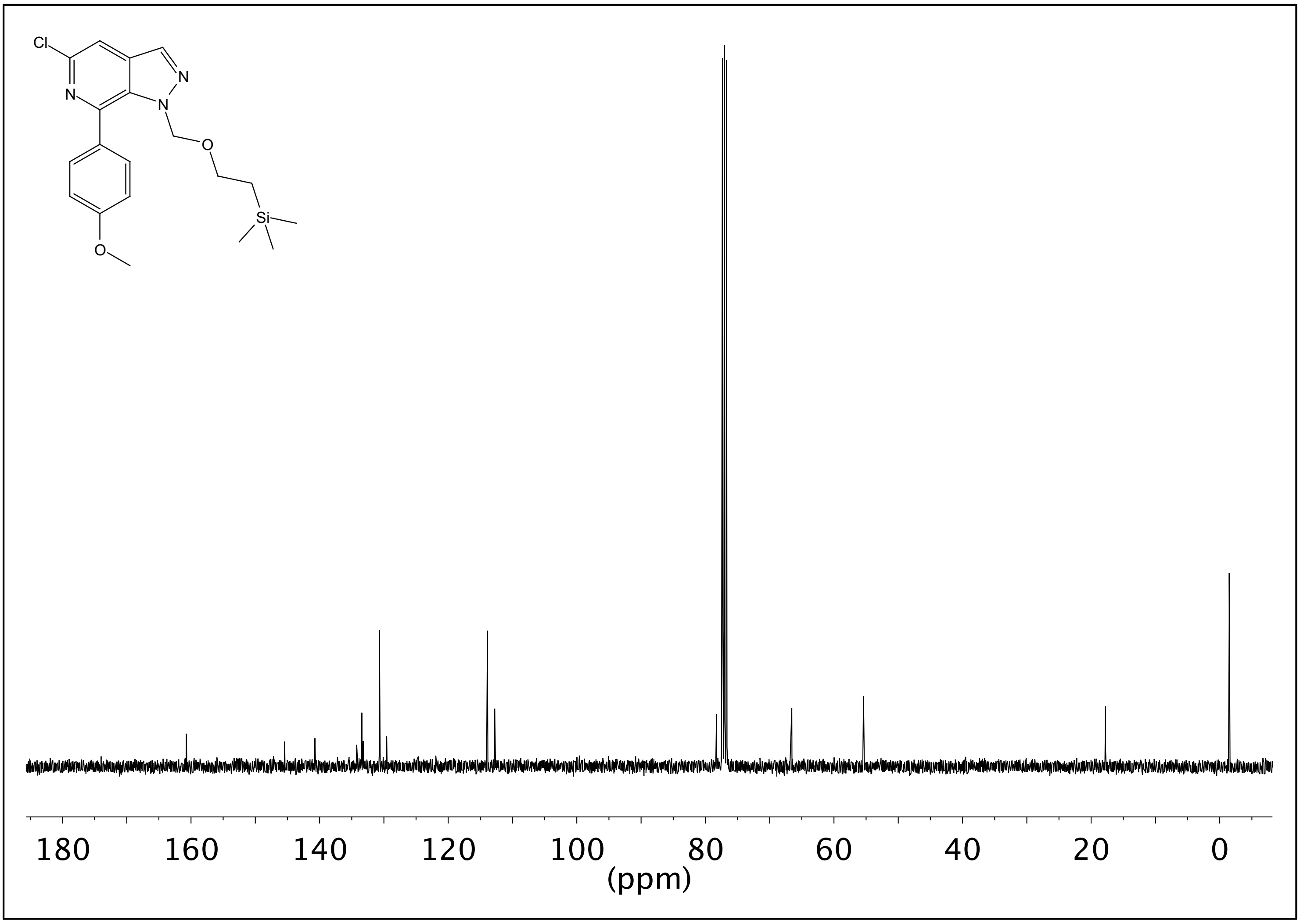

^{1}H NMR (600 MHz, Chloroform-*d*) and ^{13}C NMR (151 MHz, Chloroform-*d*) of compound **34**

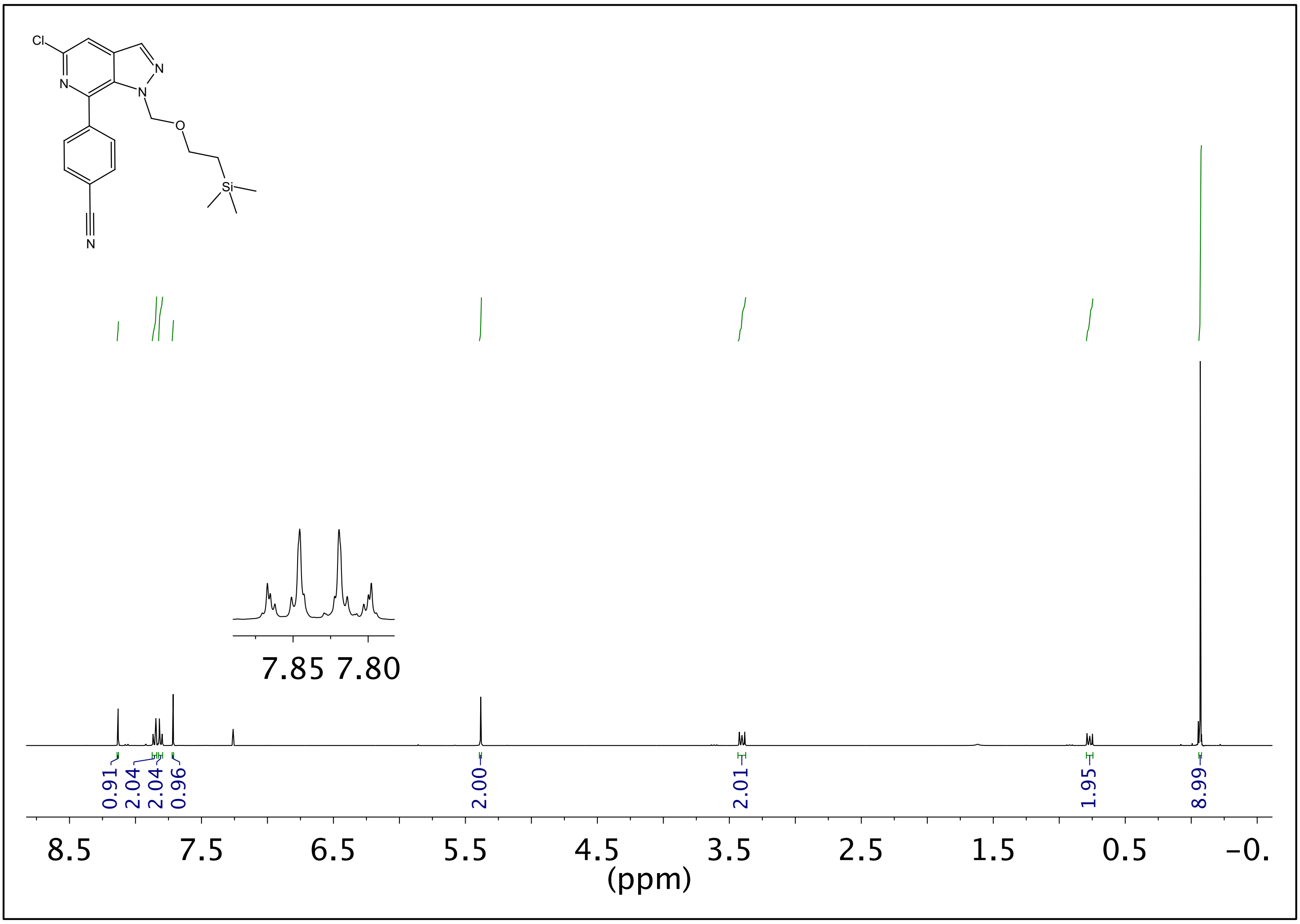

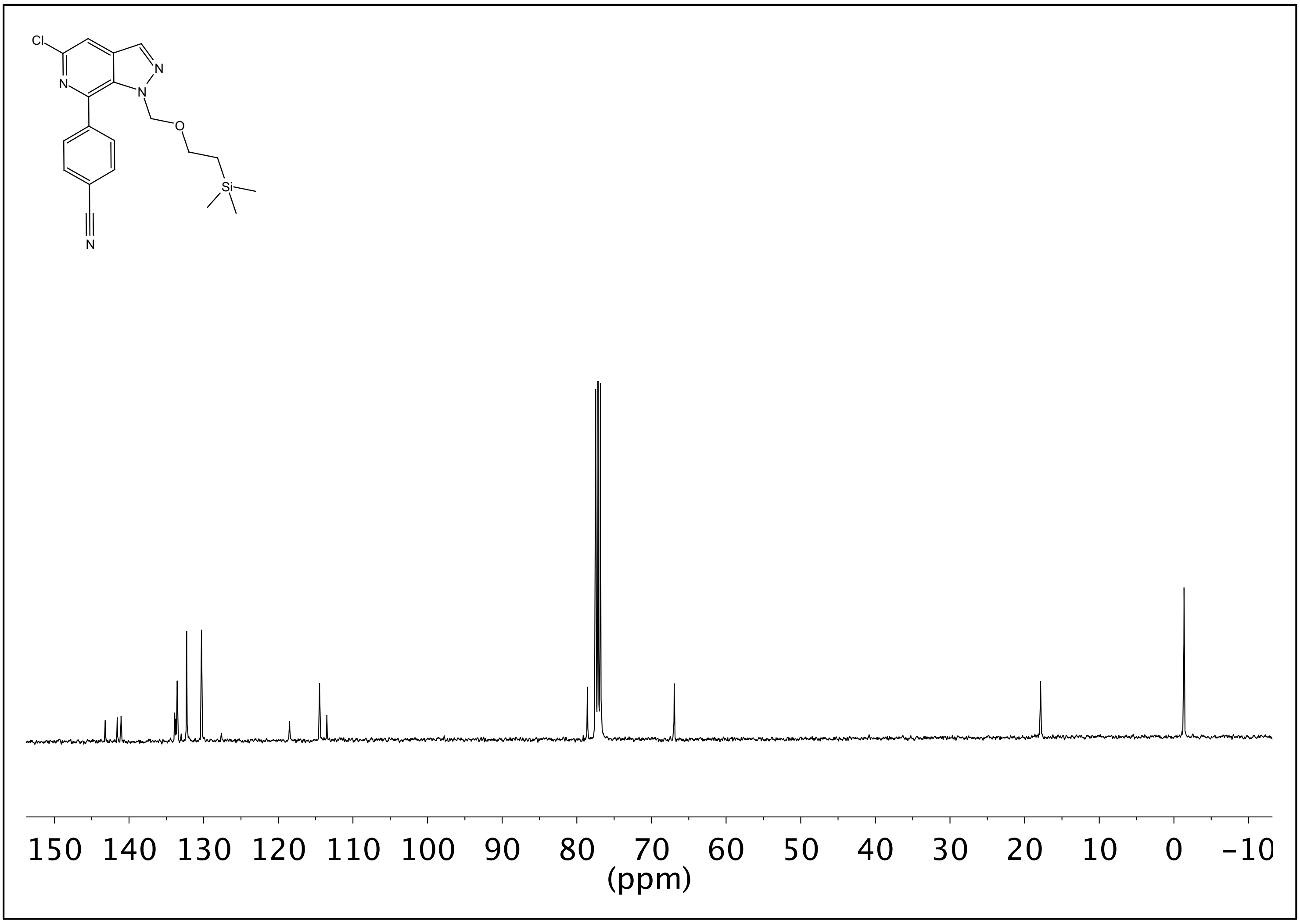

^{1}H NMR (700 MHz, Chloroform-*d*) and ^{13}C NMR (176 MHz, Chloroform-*d*) of compound **35**

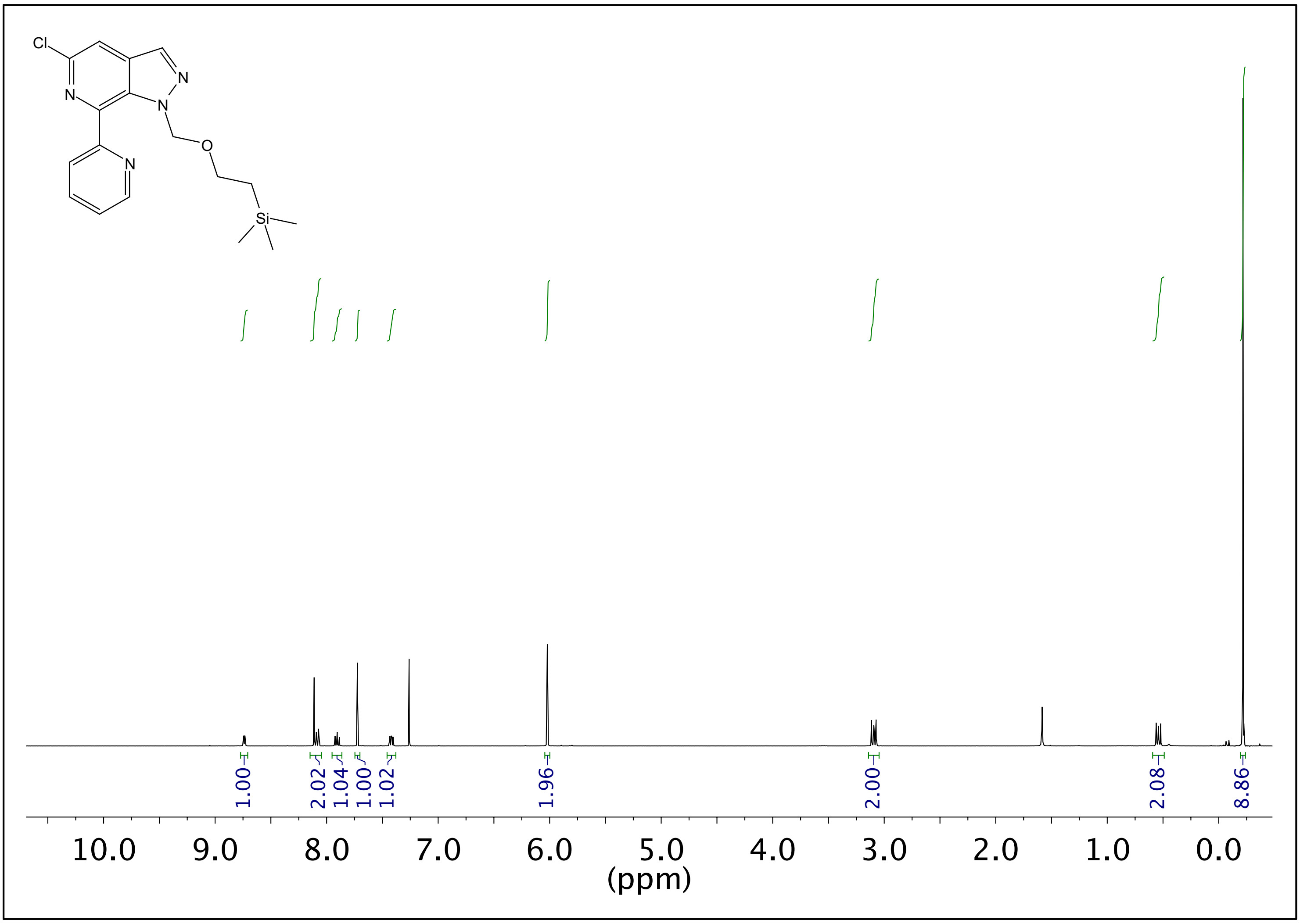

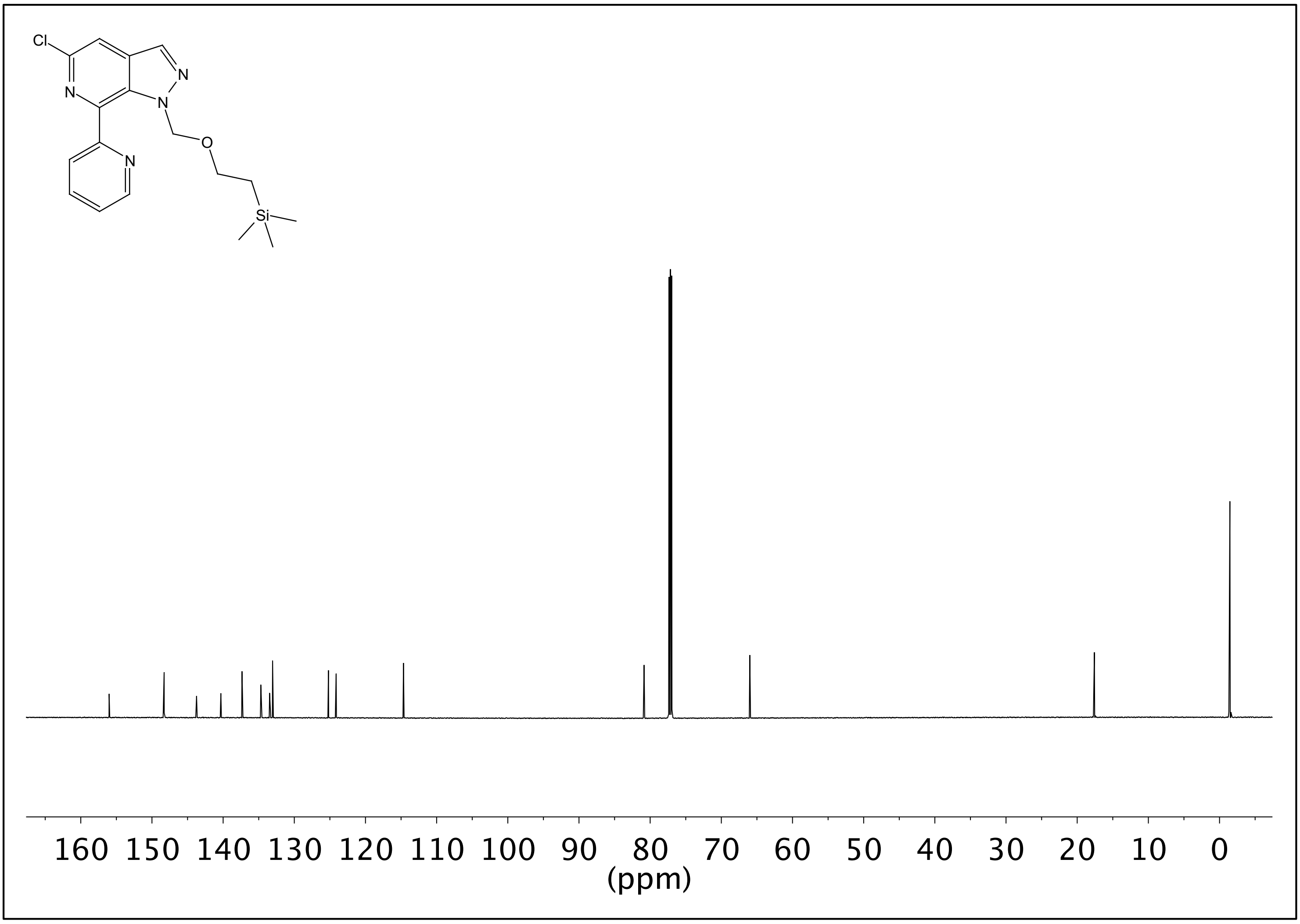

^{1}H NMR (600 MHz, Chloroform-*d*) and ^{13}C NMR (151 MHz, Chloroform-*d*) of compound **36**

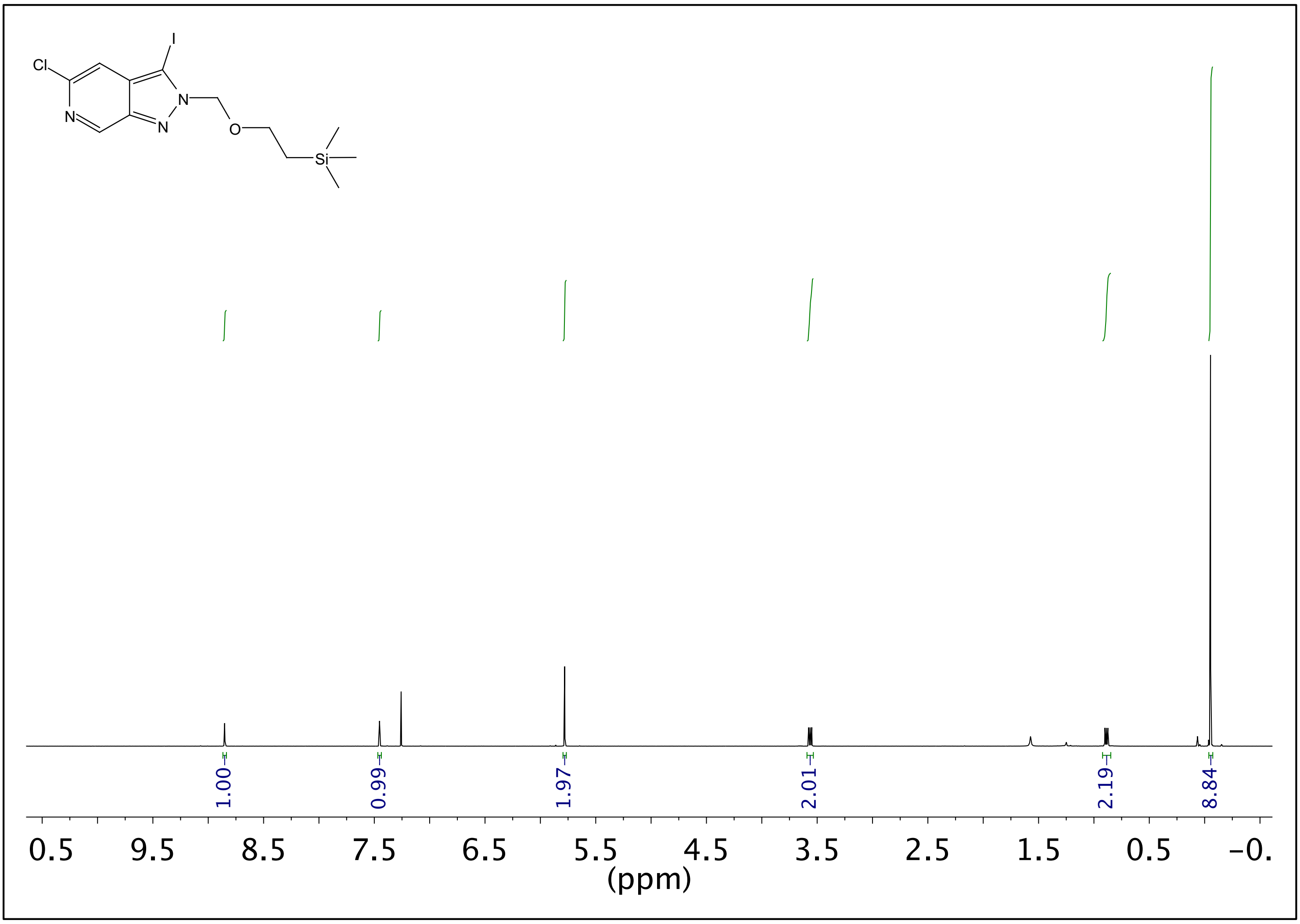

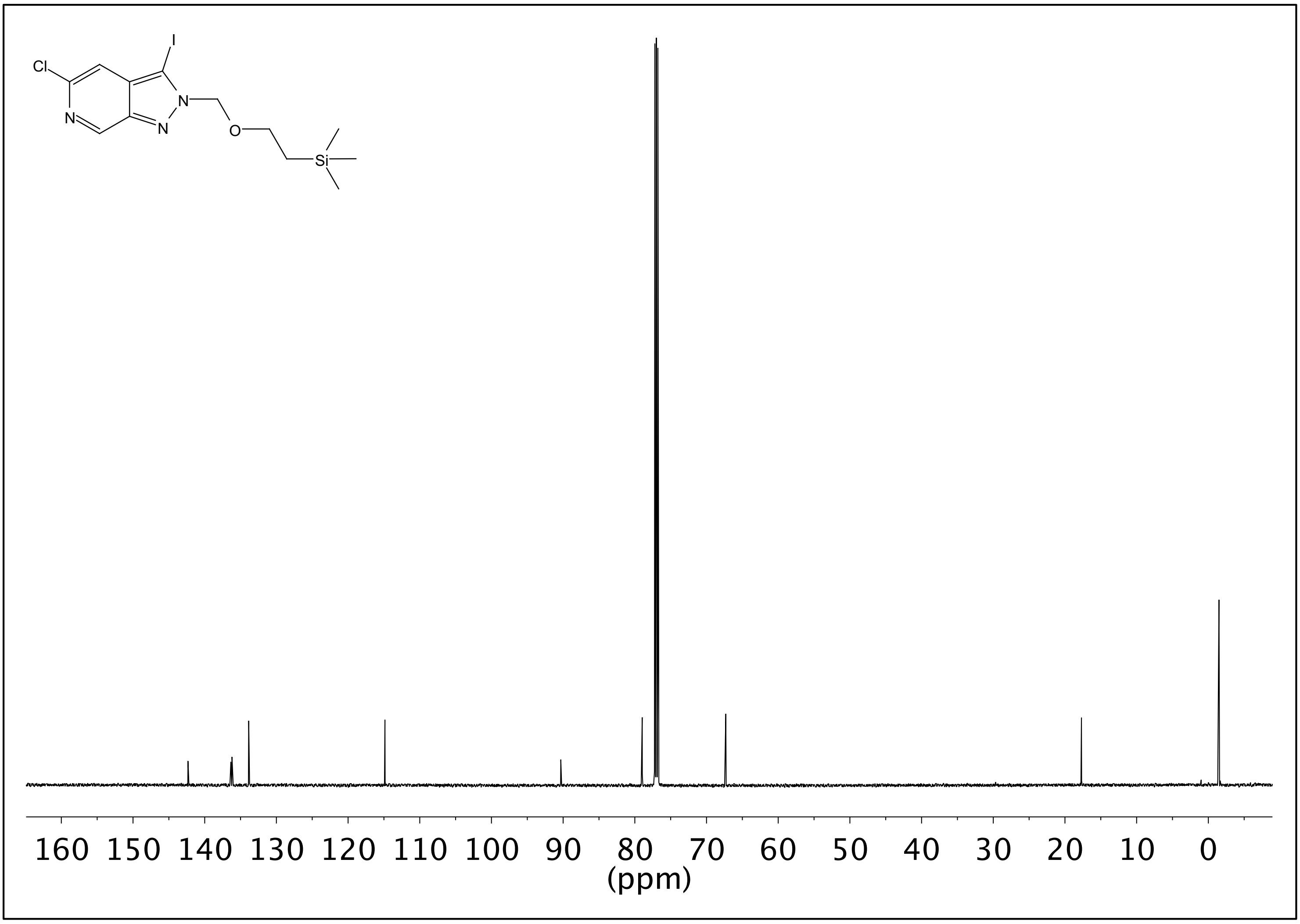

^{1}H NMR (600 MHz, DMSO) and ^{13}C NMR (151 MHz, DMSO) of compound **37**

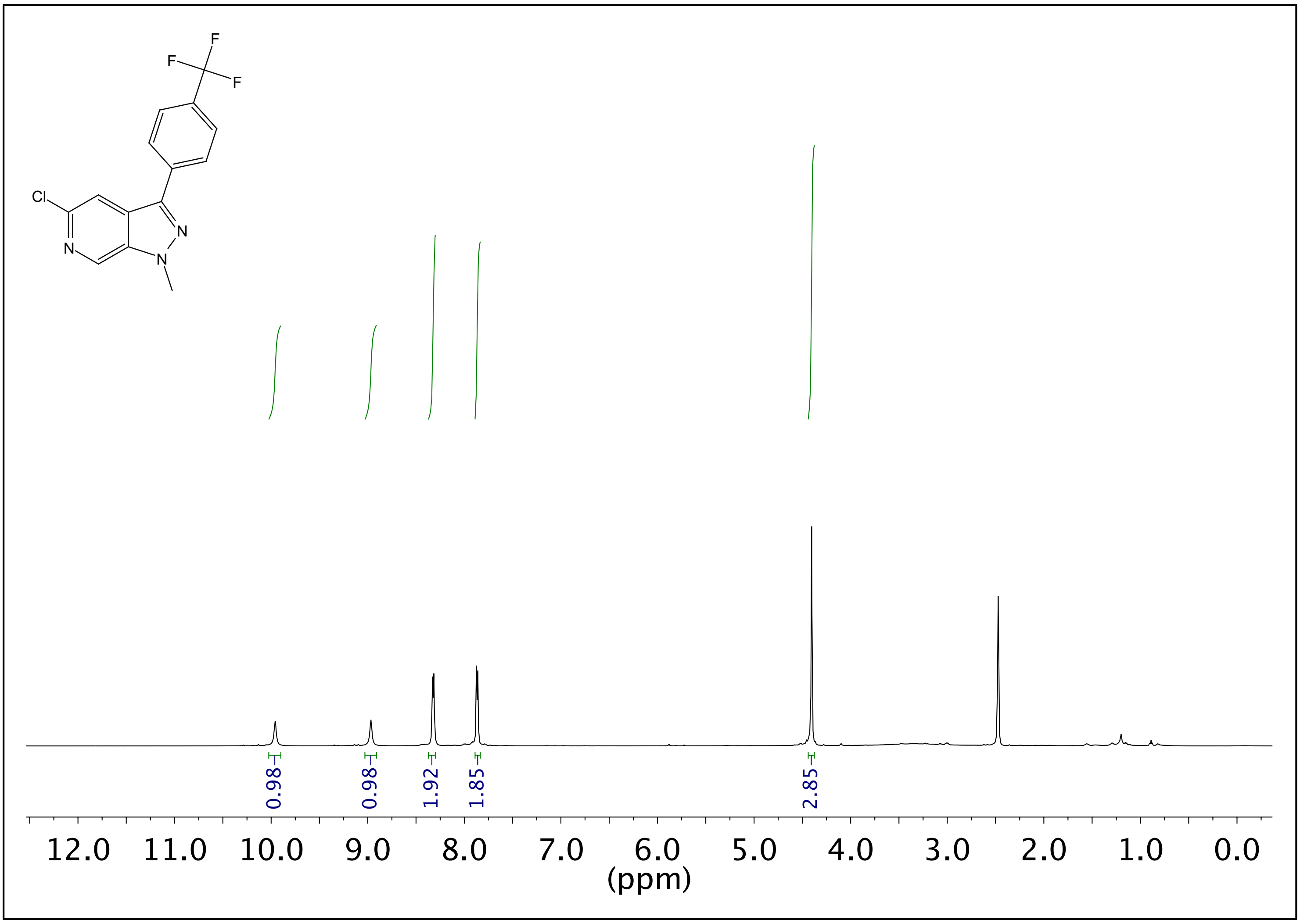

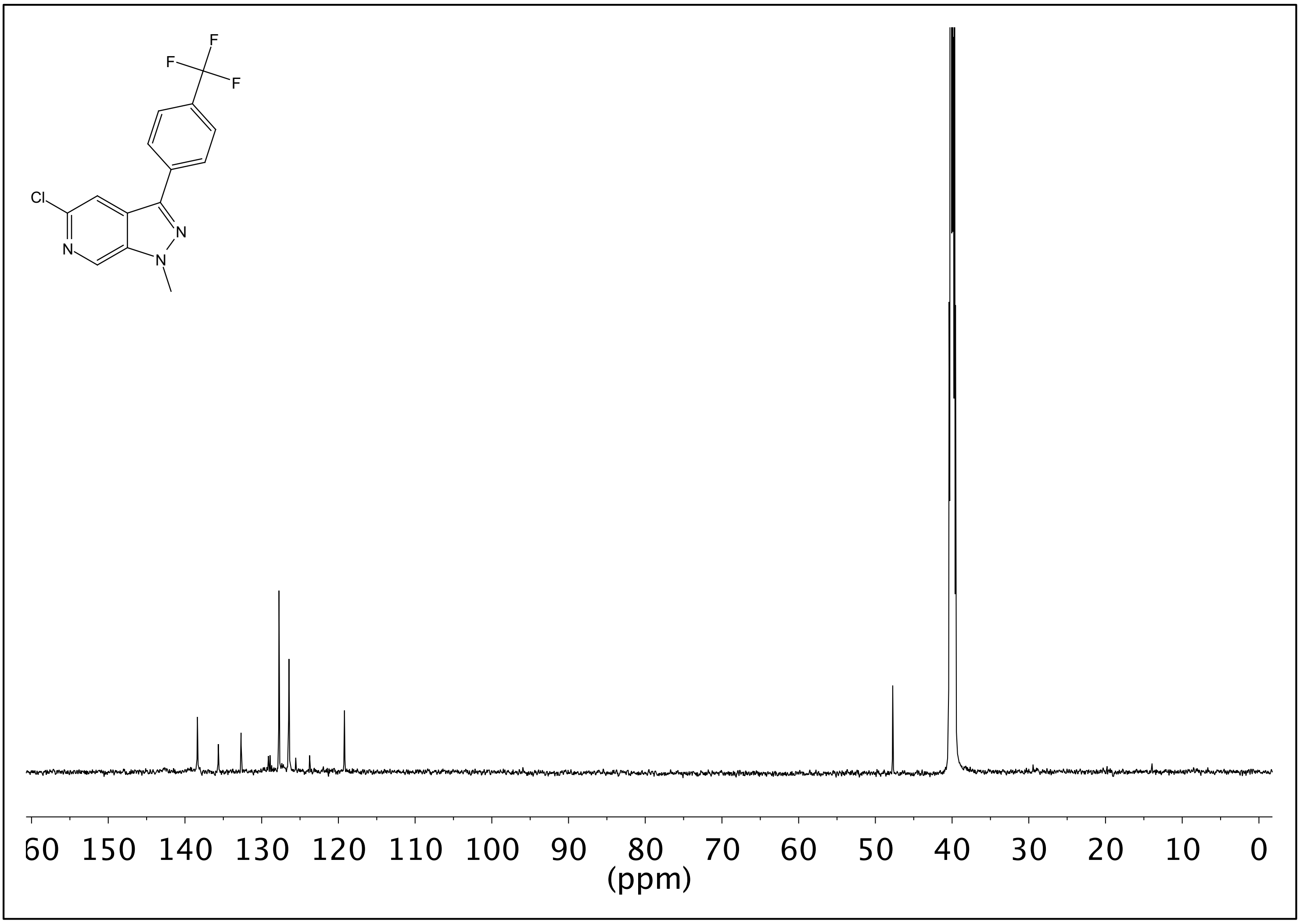

^{13}C NMR (151 MHz, DMSO) at 90 ˚C and ^{19}F NMR (376 MHz, DMSO) of compound **37**

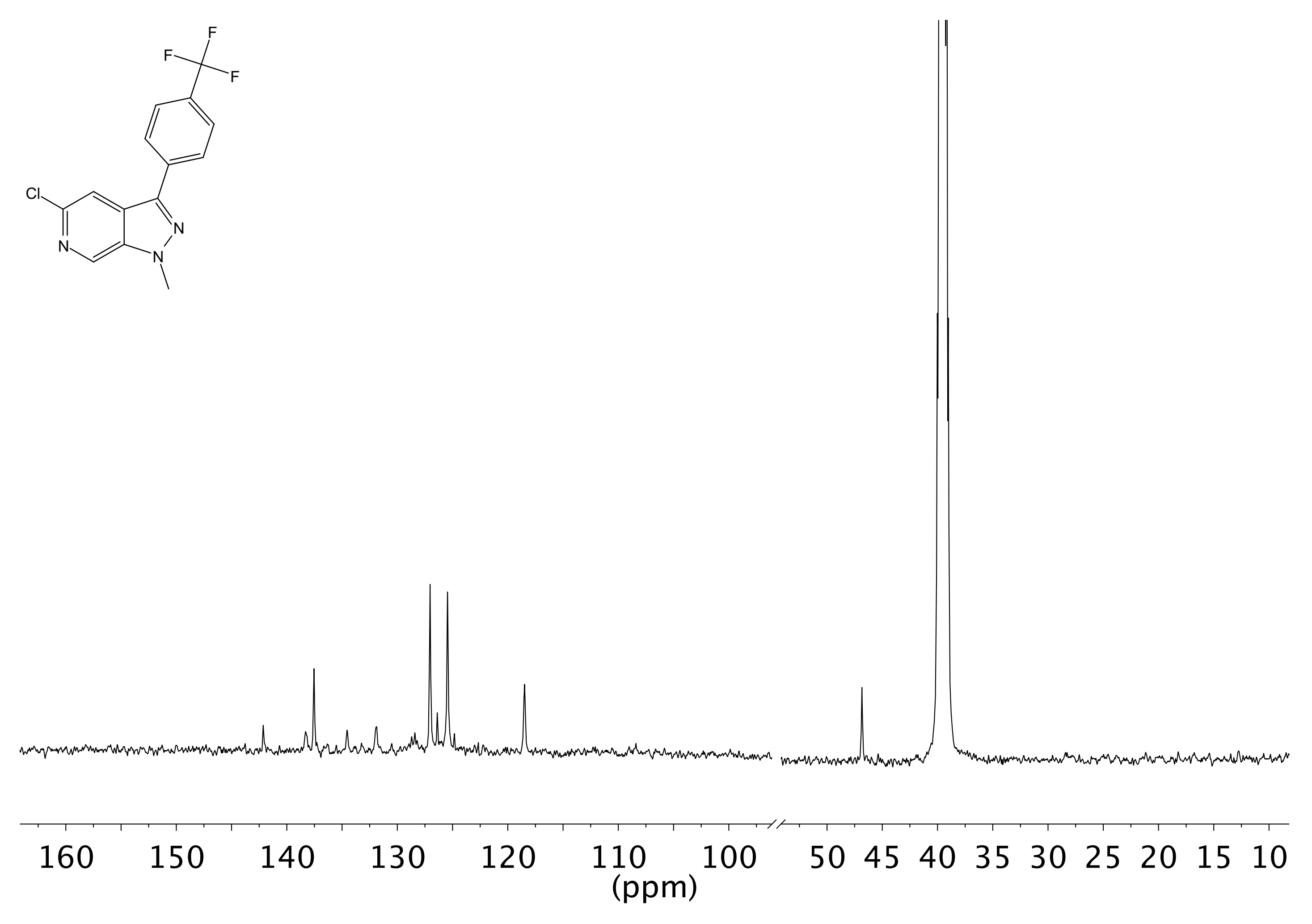

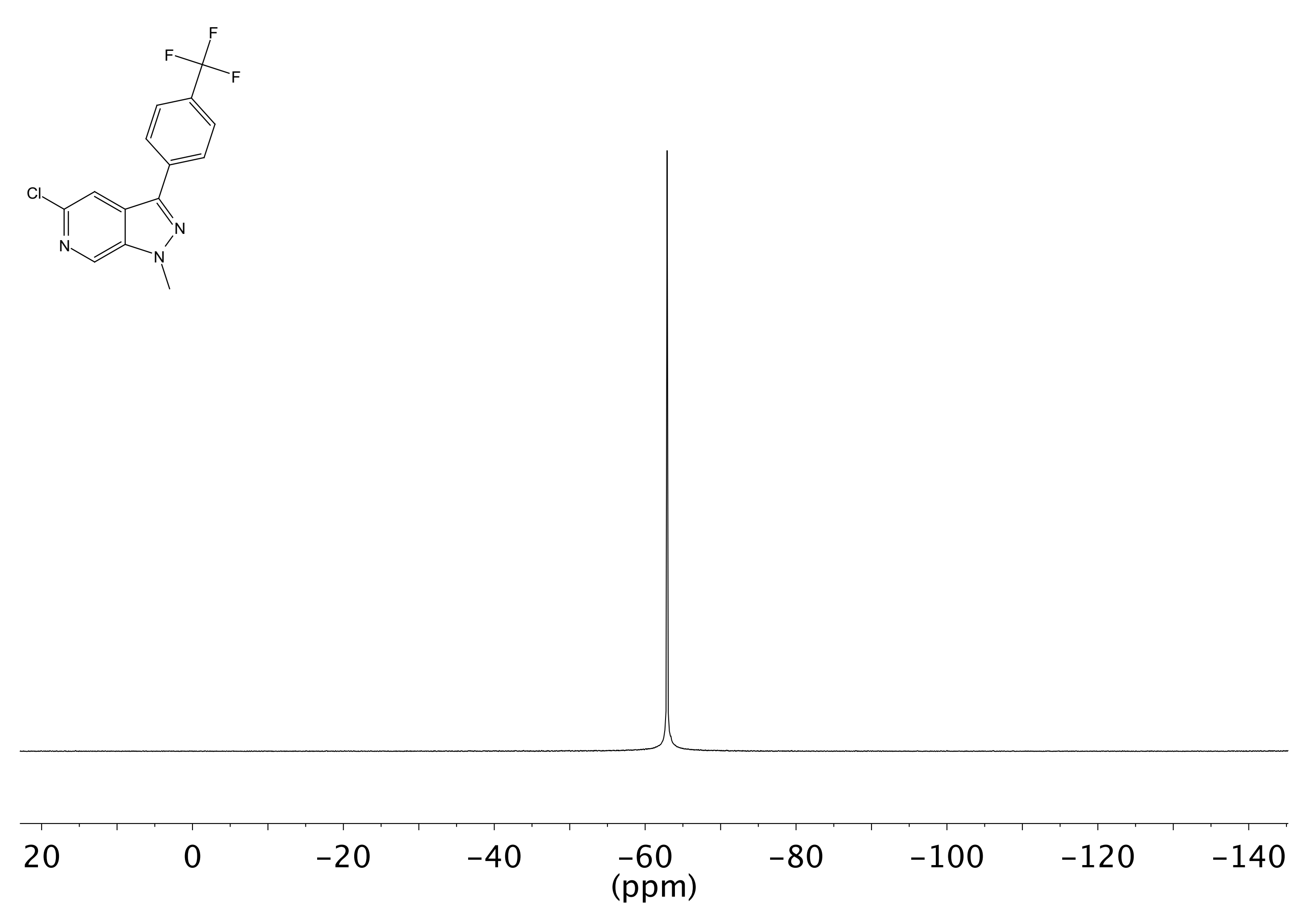

^1H NMR (400 MHz, DMSO) and ^1H NMR (600 MHz, DMSO + TFA) of compound **40**

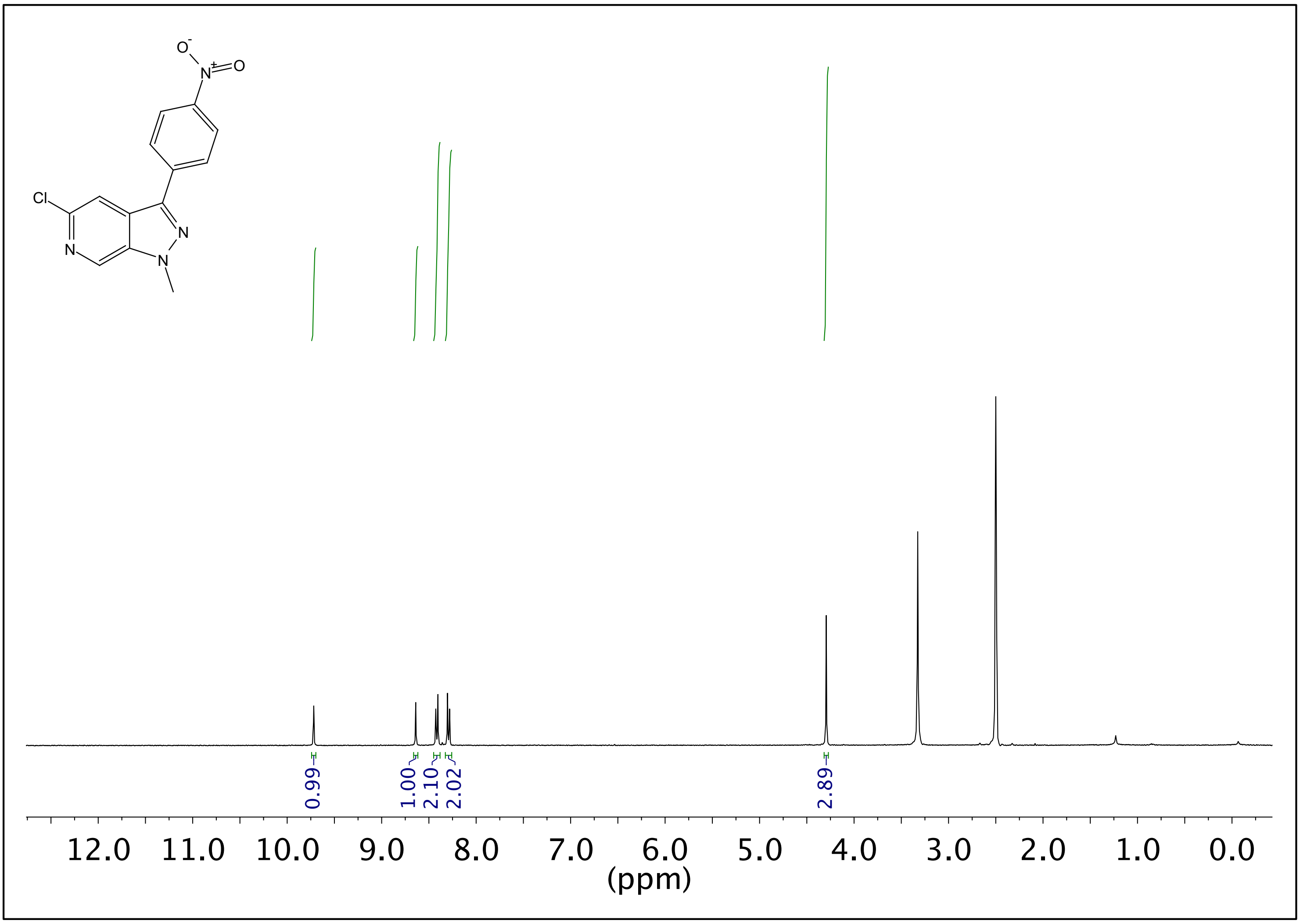

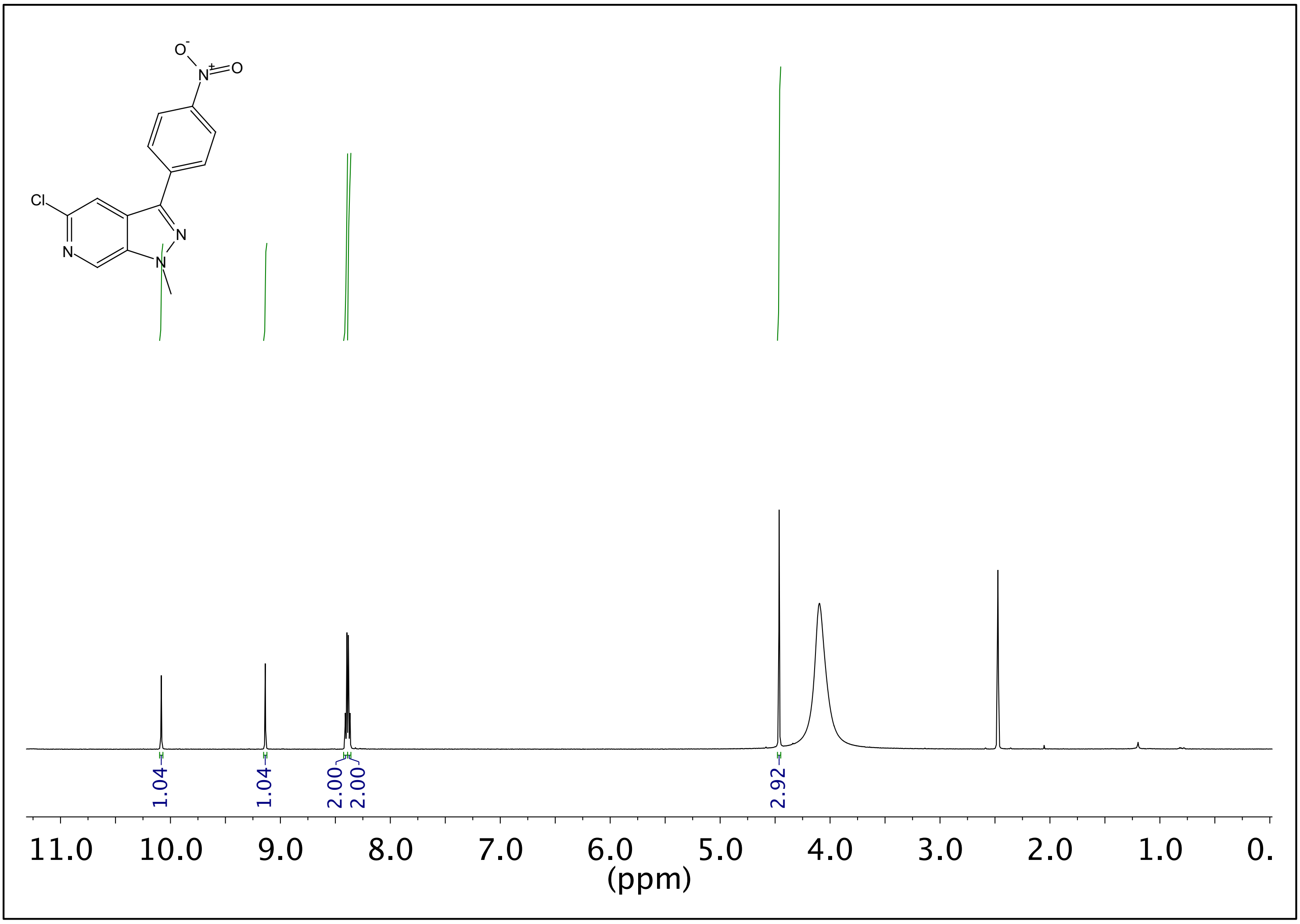

^{1}H NMR (600 MHz, DMSO + TFA) and ^{13}C NMR (151 MHz, DMSO + TFA) of compound **40**

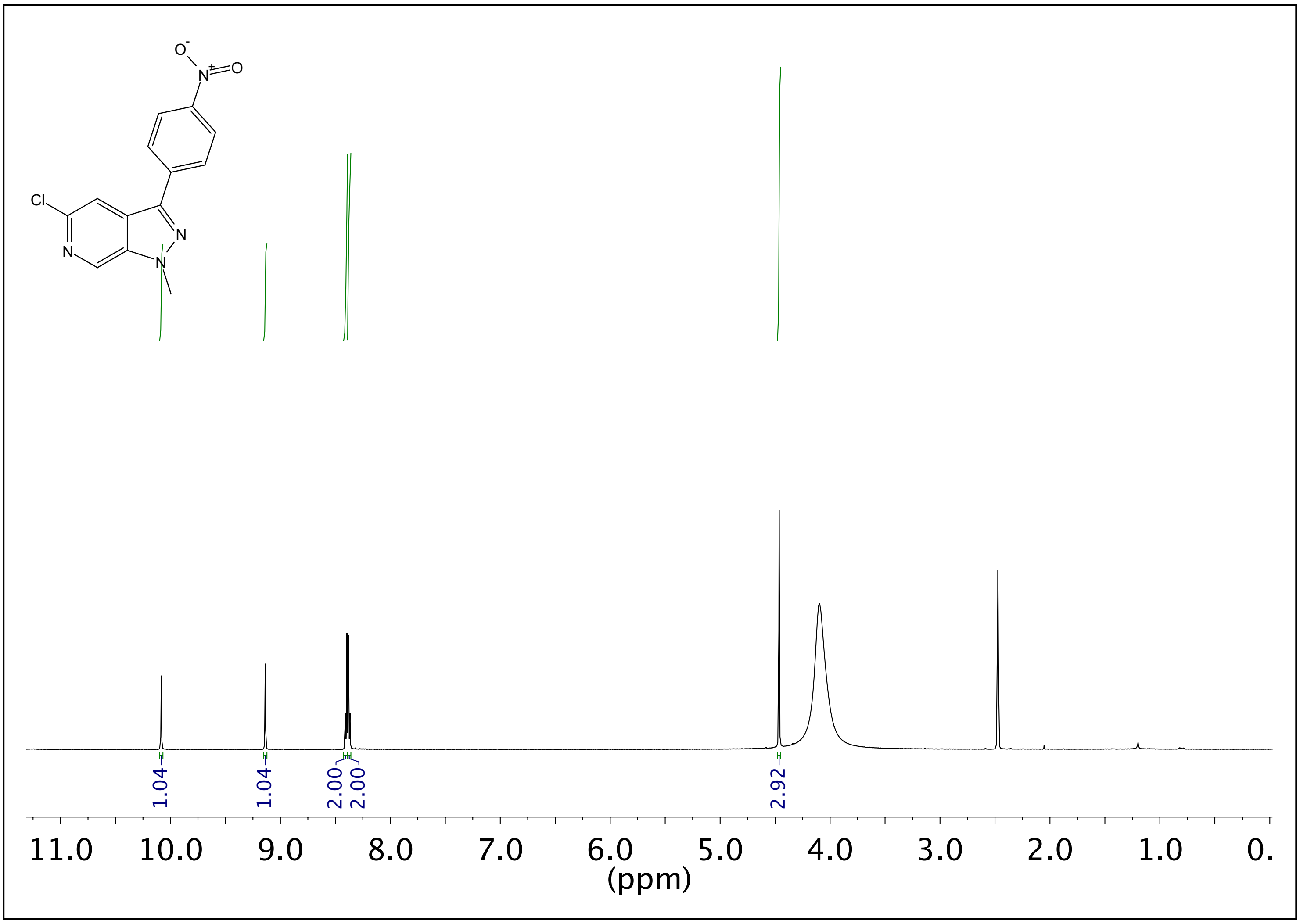

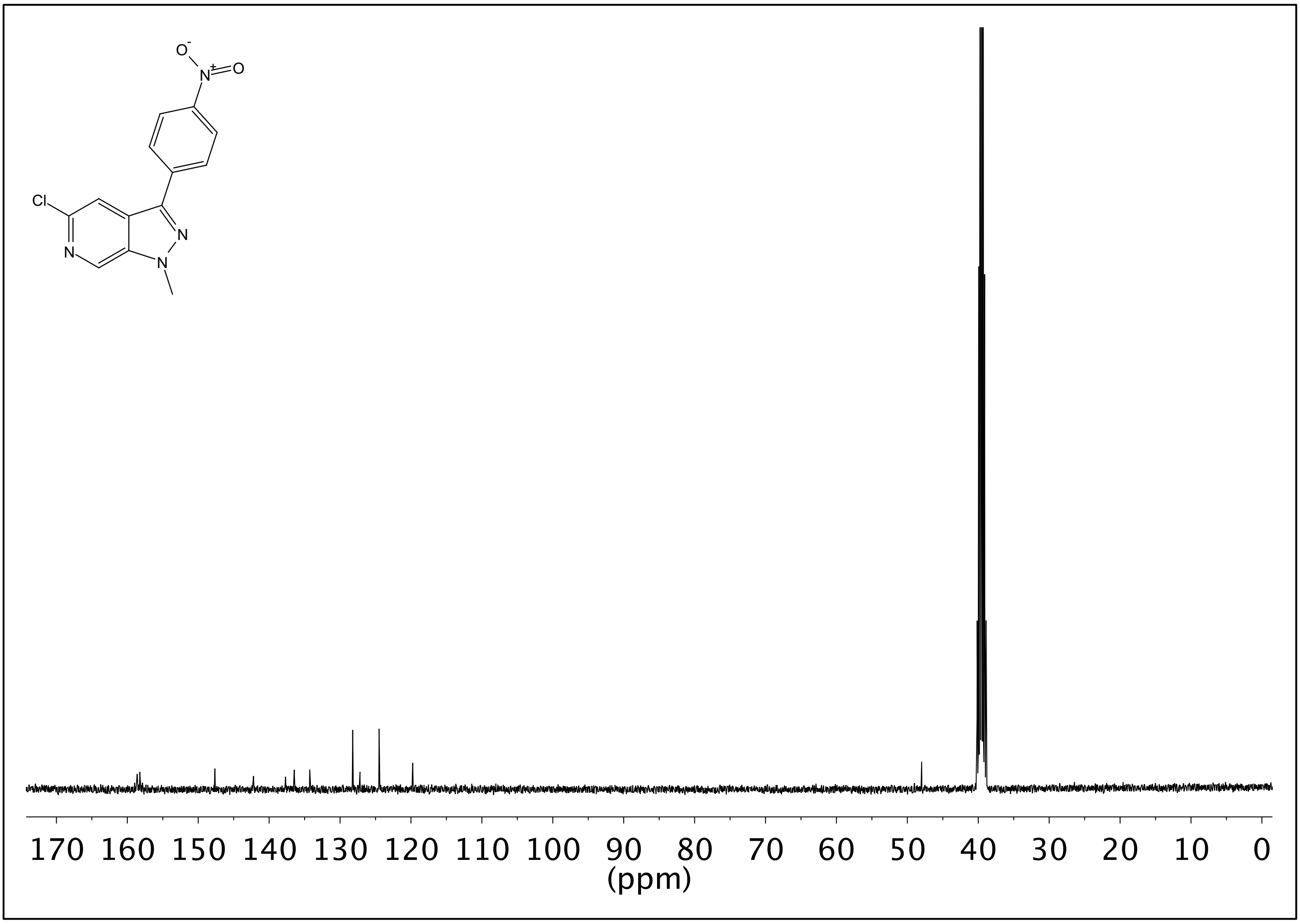

^{1}H NMR (700 MHz, Chloroform-*d*) and ^{13}C NMR (176 MHz, Chloroform-*d*) of compound **41**

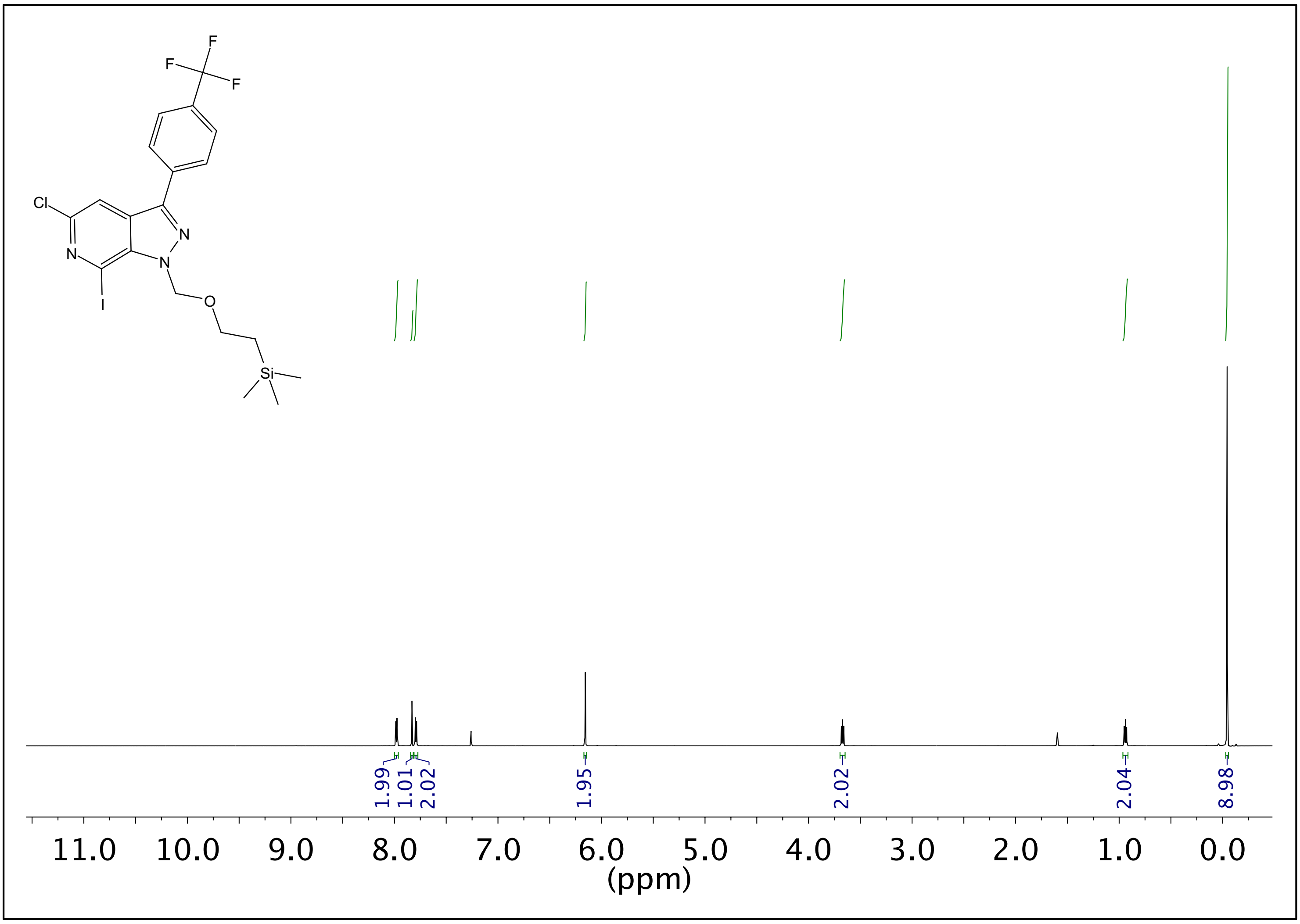

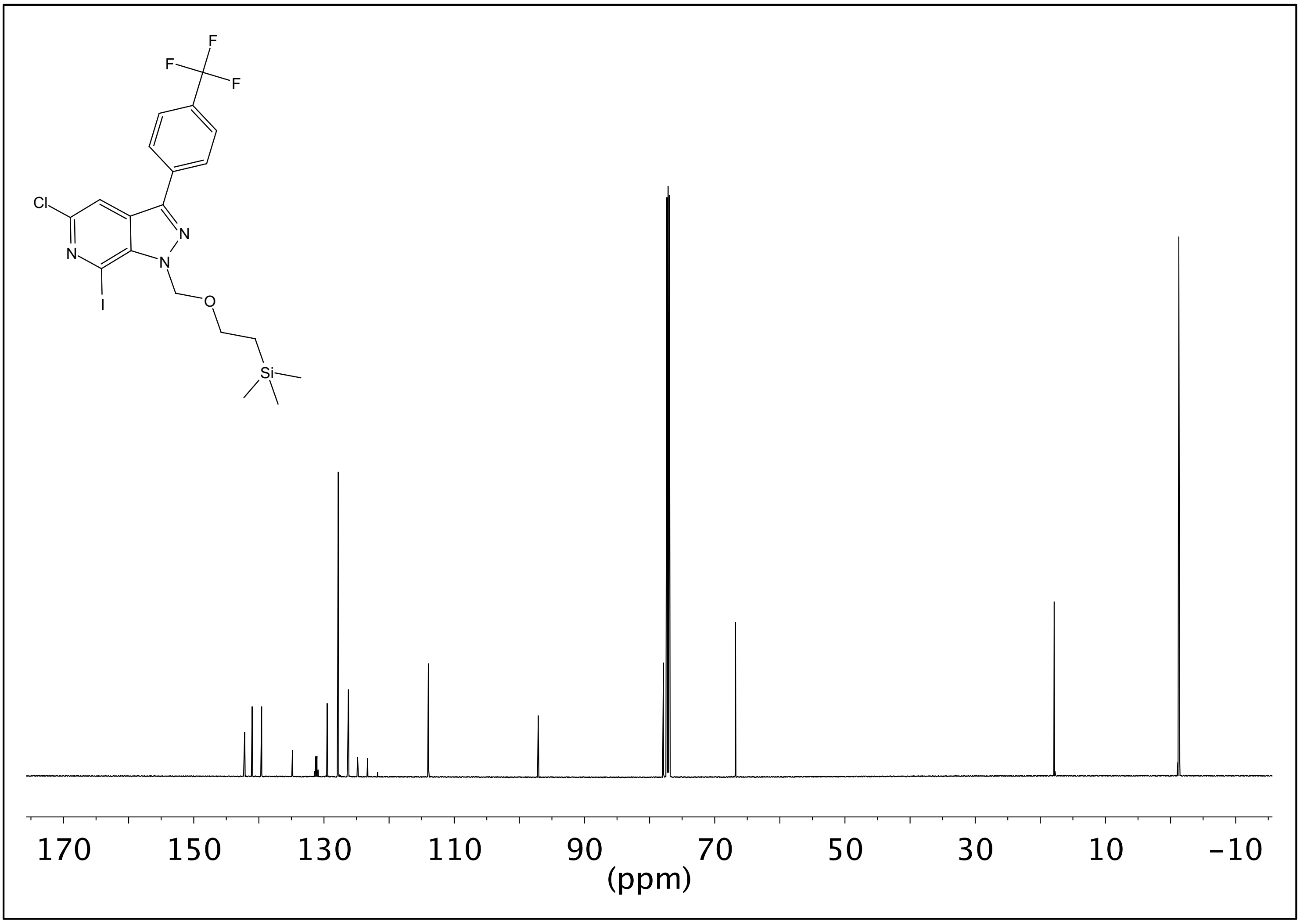

^{19}F NMR (376 MHz, Chloroform-*d*) of compound **41**

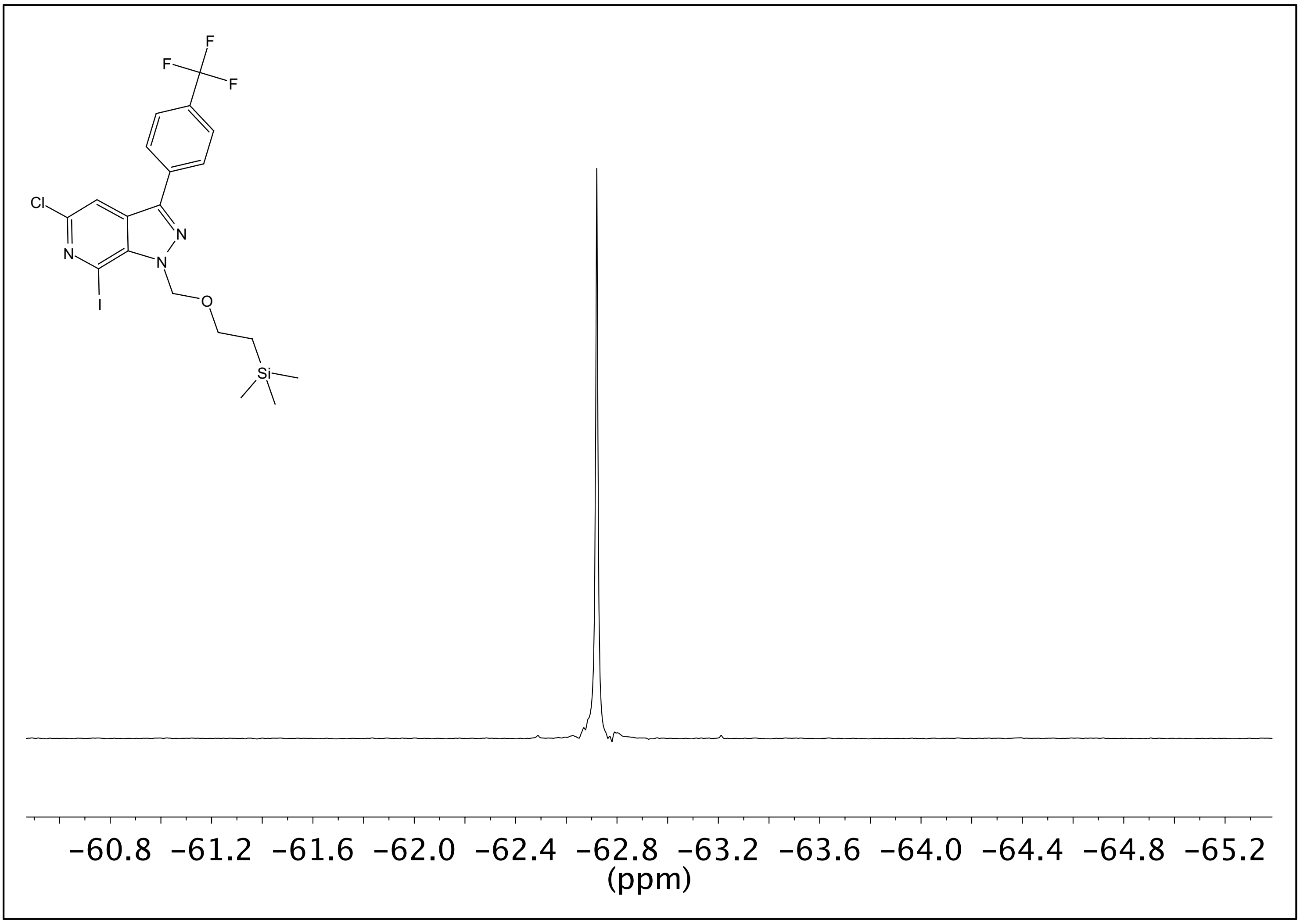

^{1}H NMR (600 MHz, Chloroform-*d*) and ^{13}C NMR (151 MHz, Chloroform-*d*) of compound **42**

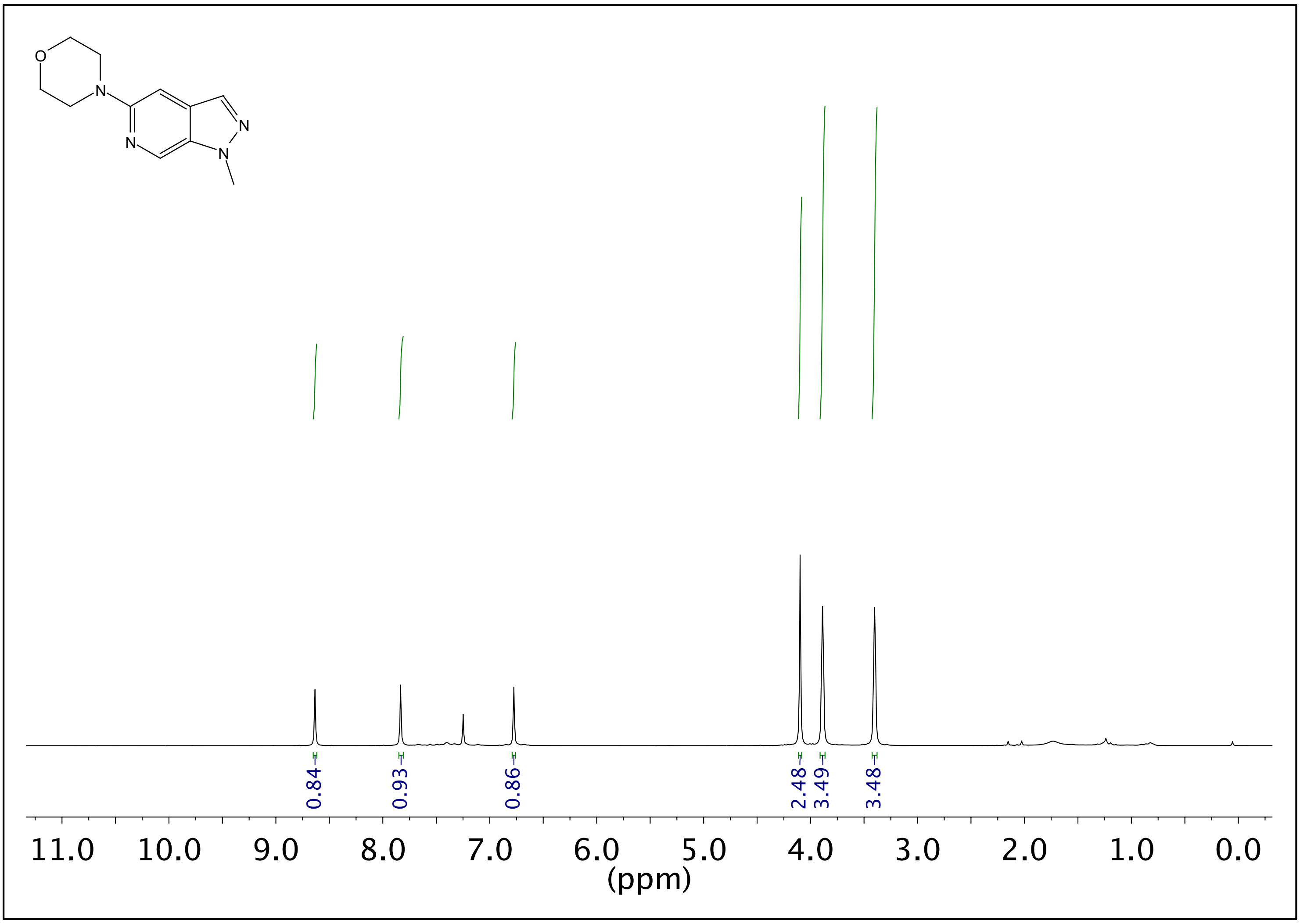

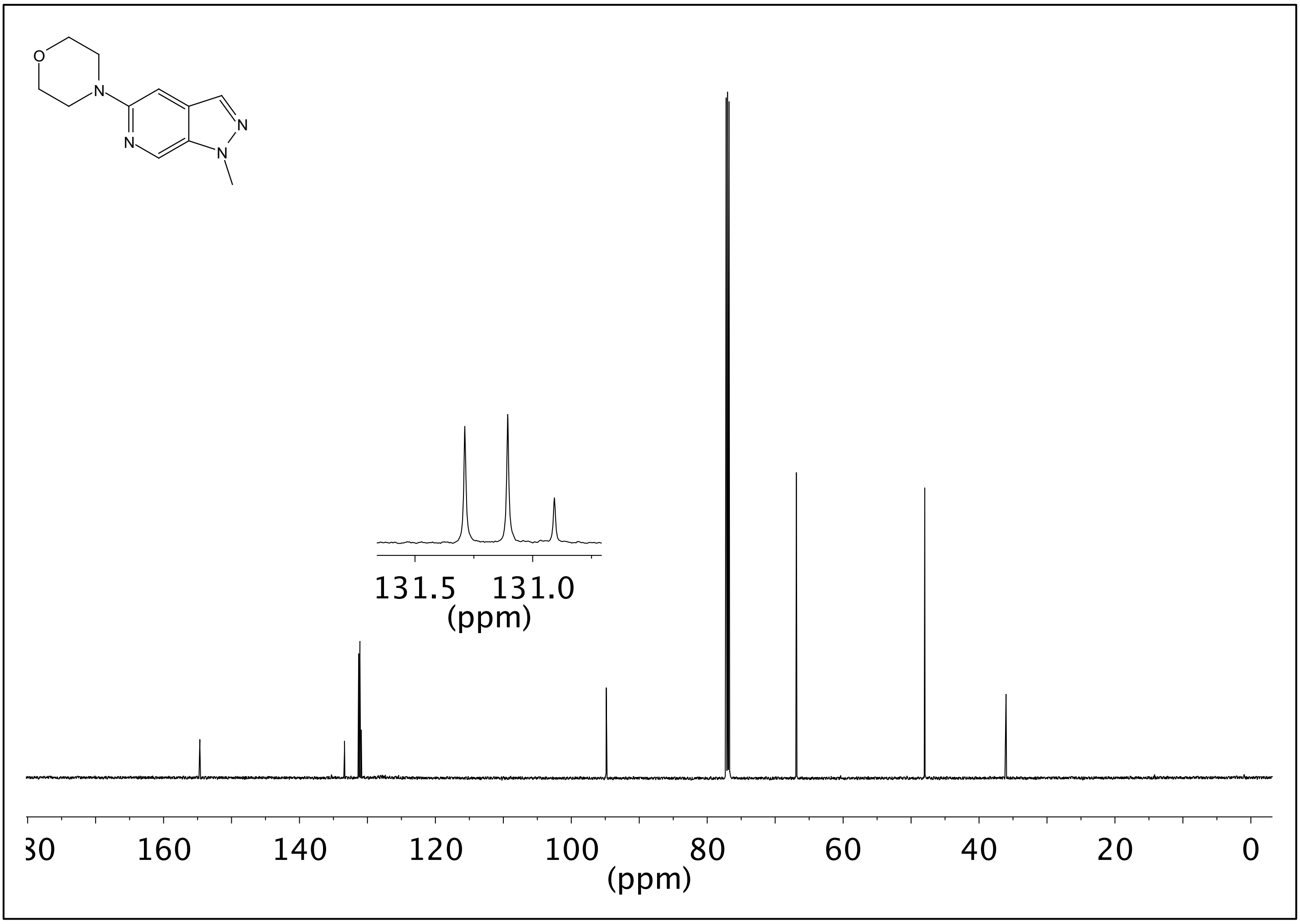

^{1}H NMR (700 MHz, Chloroform-*d*) and ^{13}C NMR (176 MHz, Chloroform-*d*) of compound **43**

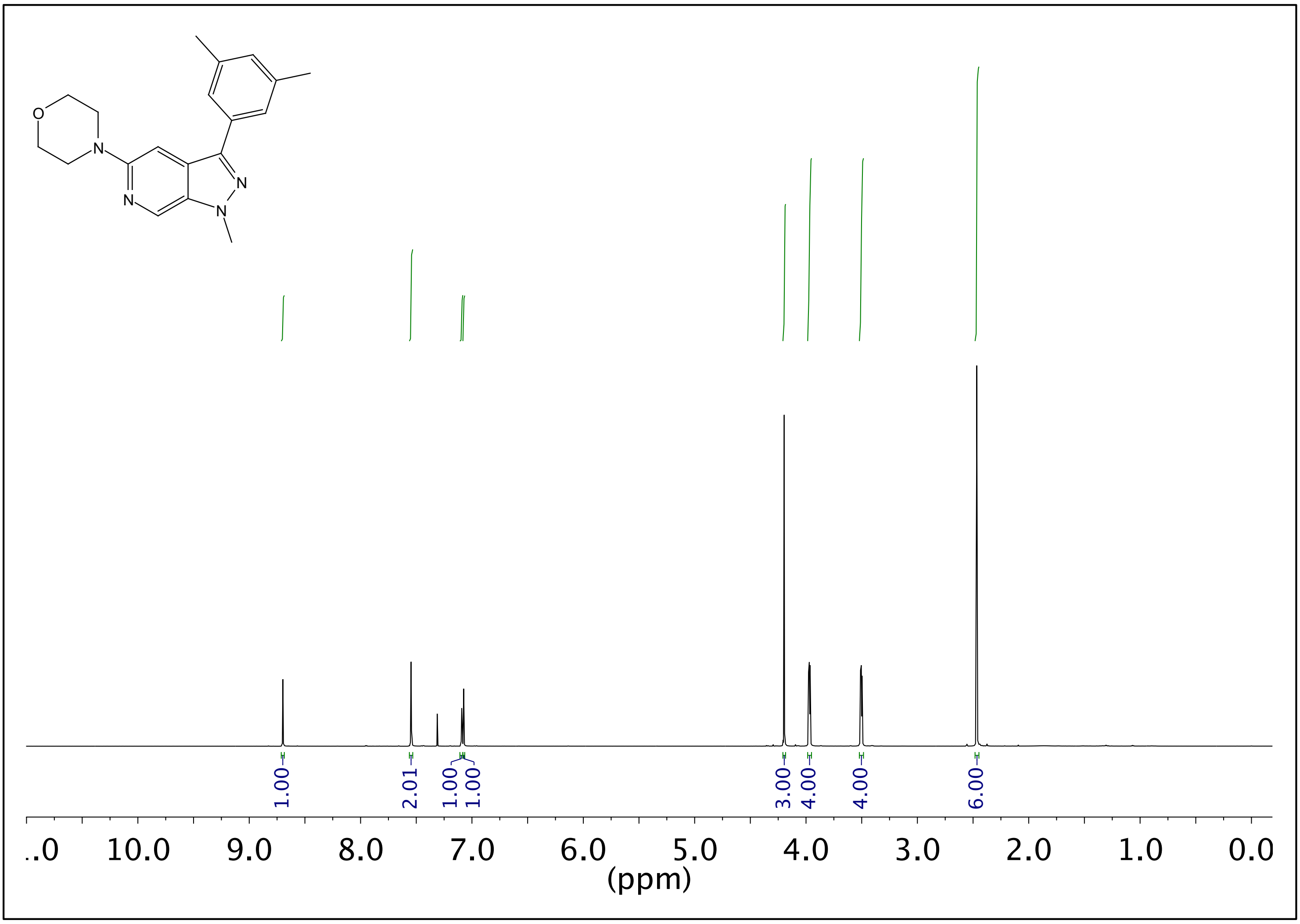

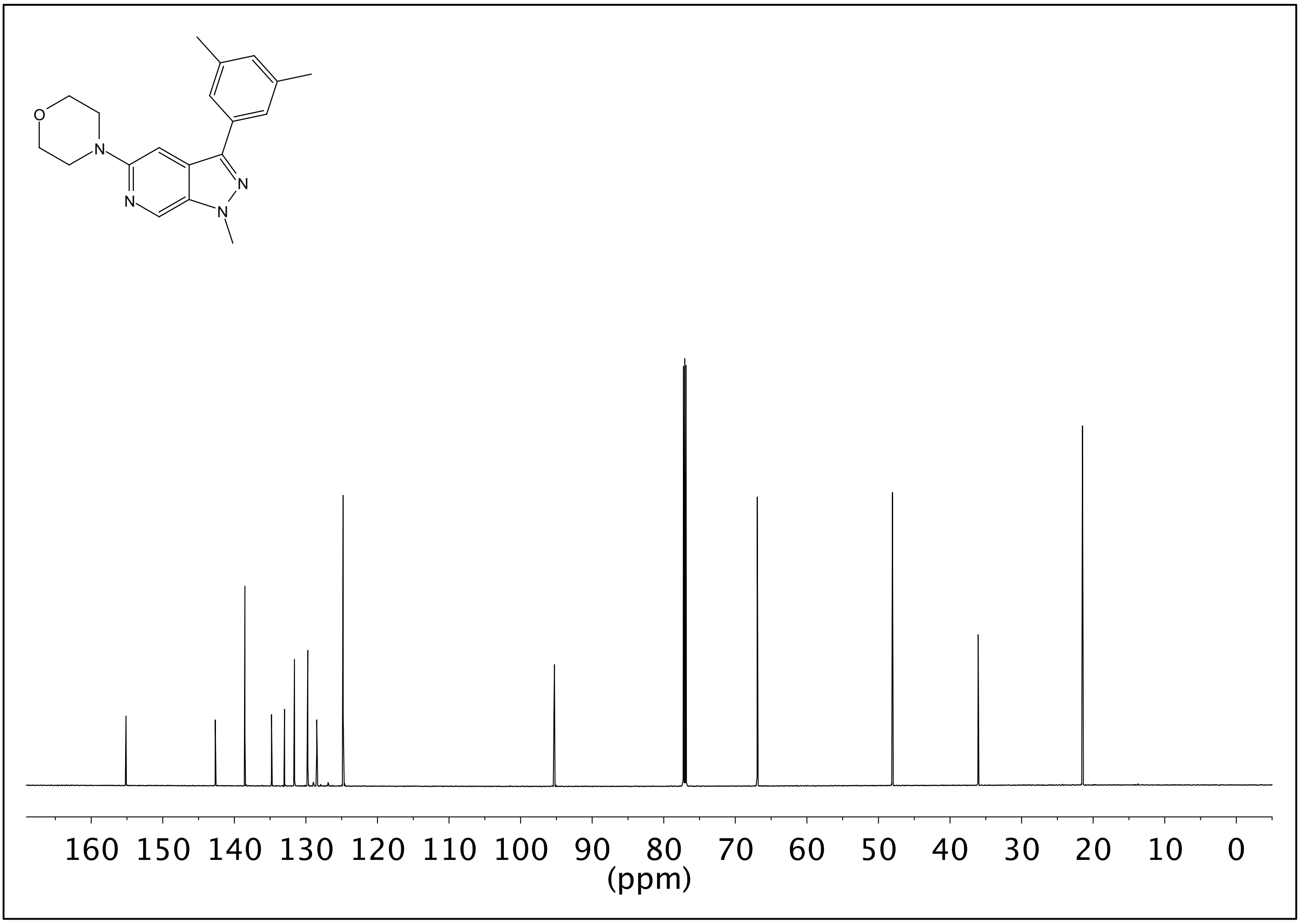

Supplement: RA-013-D3RA07458G-s001 [file RA-013-D3RA07458G-s001.pdf]
